# Prenatal antiseizure drug exposure and risk of neurodevelopmental disorders in children: population based cohort study

**DOI:** 10.1136/bmj-2025-085725

**Published:** 2026-03-11

**Authors:** Loreen Straub, Sonia Hernandez-Diaz, Brian T Bateman, Yanmin Zhu, Helen Mogun, Katherine L Wisner, Kathryn J Gray, Barry Lester, Christopher J McDougle, Page B Pennell, Krista F Huybrechts

**Affiliations:** 1 Division of Pharmacoepidemiology and Pharmacoeconomics, Department of Medicine, Brigham and Women's Hospital and Harvard Medical School, Boston, MA, USA; 2 Department of Epidemiology, Harvard T H Chan School of Public Health, Boston, MA, USA; 3 Department of Anesthesiology, Perioperative and Pain Medicine, Stanford University School of Medicine, Stanford, CA, USA; 4 Developing Brain Institute, Children’s National Hospital, Departments of Psychiatry, Pediatrics and Obstetrics and Gynecology, George Washington University School of Medicine, Washington, DC, USA; 5 Department of Obstetrics and Gynecology, University of Washington, Seattle, WA, USA; 6 Center for the Study of Children at Risk, Departments of Psychiatry and Pediatrics, Alpert Medical School of Brown University, and Women and Infants Hospital, Providence, RI, USA; 7 Department of Psychiatry, Harvard Medical School, Boston, MA, USA; 8 Lurie Center for Autism, Massachusetts General Hospital, Lexington, MA, USA; 9 Department of Neurology, University of Pittsburgh School of Medicine, Pittsburgh, PA, USA

## Abstract

**Objective:**

To evaluate whether prenatal exposure to specific antiseizure drugs increases the risk of neurodevelopmental disorders in children.

**Design:**

Population based cohort study.

**Setting:**

Healthcare use data from publicly and commercially insured beneficiaries in the United States, 2000-21.

**Participants:**

Pregnant patients with epilepsy linked to offspring.

**Interventions:**

Dispensing of the antiseizure drug of interest during the second half of pregnancy (synaptogenesis period): carbamazepine, lacosamide, lamotrigine, levetiracetam, oxcarbazepine, phenobarbital, phenytoin, topiramate, valproate, and zonisamide. The reference group consisted of pregnant patients with diagnosed epilepsy, but no antiseizure drug dispensation from three months before pregnancy until delivery.

**Main outcomes measures:**

Any neurodevelopmental disorder, attention deficit hyperactivity disorder, autism spectrum disorder, behavioral disorder, developmental coordination disorder, intellectual disability, learning difficulty, and speech or language disorder identified using validated algorithms. Hazard ratios were estimated using Cox proportional hazard models with propensity score overlap weighting to adjust for potential confounders.

**Results:**

The cohort included 8887 children who were prenatally unexposed. Exposed pregnancies ranged from 219 for lacosamide to 5261 for levetiracetam. Valproate and zonisamide showed associations with several outcomes (adjusted hazard ratio range 1.26-4.50), whereas levetiracetam and phenytoin were not associated with an increased risk of any outcome. Several drugs were associated with a two to fourfold risk increase for intellectual disability, but estimates were imprecise because of the small number of children with this disorder. Although no meaningful associations were found for topiramate and lamotrigine across most outcomes, there was a potential signal for intellectual disability (both drugs) and learning difficulty (topiramate only; hazard ratio 1.23 based on small numbers). Carbamazepine and oxcarbazepine showed a modest risk increase for attention deficit hyperactivity disorder and behavioral disorders (hazard ratio range 1.23-1.40). Results were robust across several sensitivity analyses, including using lamotrigine as an active comparator.

**Conclusions:**

The findings strengthen the evidence for increased neurodevelopmental risks among children with prenatal valproate exposure and suggest the need for further evaluation of zonisamide. Signals for other antiseizure drugs, observed in the context of several comparisons and rare outcomes, require confirmation as data accumulate.

## Introduction

Neurodevelopmental disorders (NDDs) such as attention deficit hyperactivity disorder (ADHD) and autism spectrum disorder (ASD) are prevalent conditions, affecting a large number of children globally.^1^ ^2^ ^3^ ^4^ In the United States, approximately seven million children (11.4%) aged 3-17 years have ever been diagnosed with ADHD based on a 2022 national parent survey,^5^ and about one in 31 (3.2%) has been diagnosed with ASD by age 8 according to estimates from the Centers for Disease Control and Prevention’s Autism and Developmental Disabilities Monitoring Network in 2022.^6^ The cause of NDDs is multifactorial, involving a complex interplay of genetic and environmental factors.^7^ ^8^ Among the various environmental components, prenatal exposure to drugs that act on the central nervous system has garnered considerable attention given the potential for these agents to influence fetal brain development during critical periods of neurogenesis and synaptogenesis.^9^

Antiseizure drugs, commonly prescribed for epilepsy and other indications (eg, bipolar disorders, migraine prophylaxis), are frequently and increasingly used by women of childbearing age.^10^ ^11^ ^12^ Maintaining maternal seizure control during pregnancy through continued antiseizure drug treatment is critical because uncontrolled seizures are associated with a heightened risk of maternal and fetal complications.^13^ Therefore, patients and their providers face a challenging balancing act because antiseizure drugs themselves may also create risks. The safety of antiseizure drugs during pregnancy, particularly relating to major congenital malformations,^14^ ^15^ ^16^ ^17^ ^18^ has been subject to extensive research, and more recently, the impact of these drugs on a child’s neurodevelopment has come under scrutiny.^19^ ^20^ ^21^ ^22^ ^23^ ^24^ Several studies have established a link between prenatal exposure to valproate and an increased risk of specific NDDs in children, including ADHD, ASD, and reduced cognitive abilities.^9^ ^19^ ^22^ ^23^ ^24^ ^25^ ^26^ ^27^ ^28^ ^29^ ^30^ ^31^ ^32^ ^33^ ^34^ ^35^ ^36^ ^37^ ^38^ ^39^ ^40^ ^41^ Recent findings from the Maternal Outcomes and Neurodevelopmental Effects of Antiepileptic Drug (MONEAD) study, a prospective cohort study examining maternal antiseizure drug blood concentrations during pregnancy and child neurodevelopment, suggest an inverse association between third trimester lamotrigine levels and behavioral functioning, and between levetiracetam levels and cognitive and behavioral functioning. However, sample sizes were small overall and functioning scores showed strong variability even among children with similar maternal antiseizure drug levels. The overall risk among children prenatally exposed to these drugs was still considered low and not substantially different from healthy controls.^42^ ^43^ Previous studies have typically not shown adverse neurodevelopmental effects for lamotrigine and levetiracetam, although comprehensive evaluations of specific NDDs are sparse.^9^ ^19^ ^22^ ^23^ ^24^ ^35^ ^36^ ^37^ ^38^ ^41^ ^44^ ^45^ ^46^ ^47^ ^48^ ^49^

For topiramate, regulatory agencies such as the European Medicines Agency and the UK Medicines and Healthcare products Regulatory Agency advise against its use during pregnancy because of a potential increased risk of ADHD, ASD, and intellectual disability reported in two Nordic studies,^23^ ^37^ ^50^ ^51^ although the risk of ASD was not confirmed in subsequent studies.^24^ ^52^ More uncertainty exists for other antiseizure drugs that have been in use for several decades like carbamazepine and phenobarbital, and the potential risks associated with newer drugs like lacosamide are poorly characterized.^31^ ^32^ ^42^ ^43^ ^44^ ^47^ Therefore, the current practice guidelines from the American Academy of Neurology advise against the use of valproate because of its link with major congenital malformations and NDDs. The guidelines recommend using lamotrigine, levetiracetam, or oxcarbazepine, when appropriate, given the current evidence supporting the lower risk of major congenital malformations for these drugs compared with other antiseizure drugs. However, the guidelines also emphasize that “[n]umerous ASMs [antiseizure medications] have limited available data on neurodevelopmental outcomes,”^53^ highlighting the importance of generating robust evidence to guide clinical decision making and ensure maternal and fetal safety.

In summary, despite the growing interest in potential neurodevelopmental sequelae of prenatal antiseizure drug exposure, the evidence base remains limited. This lack of evidence is partly because robust assessment of NDD risk requires very large cohorts with long term follow-up into childhood, which is often infeasible in prospective studies. Although such cohorts are well suited to document subtle manifestations of NDDs (eg, abnormal findings on neonatal magnetic resonance imaging, or cognitive or behavioral assessments), they are typically underpowered to evaluate the risk of debilitating conditions, such as ASD, ADHD, or specific developmental delays. Administrative claims data offer a unique opportunity in this context, yet historically have been used primarily to evaluate outcomes occurring during pregnancy or shortly after birth, such as congenital malformations. We have previously shown the feasibility of leveraging these data sources to identify and study NDDs, including after prenatal drug exposure.^4^ ^24^ ^54^ ^55^ ^56^ ^57^ This approach enables evaluation of common and rare NDDs across large, diverse populations, representing a new contribution to the evidence base on antiseizure drug safety in pregnancy. In this study, using two large US healthcare use databases, we aimed to assess the association between prenatal exposure to individual antiseizure drugs and the risk of specific NDDs in children.

## Methods

### Data source and study cohort

This study included cohorts of publicly and commercially insured pregnant women (including female people with other gender identities) and their liveborn children nested within the nationwide Medicaid Analytic eXtract/Transformed Medicaid Statistical Information System Analytic Files (MAX/TAF, 2000-18) and the Merative MarketScan Commercial Claims and Encounters Database (MarketScan, 2003-21). The development of these cohorts has been described before.^58^ ^59^ Both data sources include patient level information on demographics, insurance enrollment, outpatient drug dispensing, outpatient and emergency department visits and hospital admissions, and their accompanying diagnoses and procedures. Women aged 12-55 years were required to have continuous insurance coverage from at least three months before pregnancy to one month after delivery. Children were followed from birth until the end of continuous enrollment, diagnosis of the NDD of interest, the end of the study period, or death, whichever happened first. Children with a known chromosomal or genetic anomaly were excluded because the cause of NDDs in these children is unlikely to be related to prenatal antiseizure drug exposure (eFigures 1 and 2). For the main analyses, the cohort was further restricted to women with epilepsy, the main indication for the antiseizure drugs of interest (eAppendix 1 gives definition).

### Exposure to antiseizure drugs

Children were considered exposed if their mothers filled at least one antiseizure drug prescription (as monotherapy or polytherapy) during the second half of pregnancy, the period when neural connections in the fetal brain undergo major development,^60^ ^61^ and disruptions (eg, from drug exposure) can affect cognitive function, behavior, and overall brain structure. Although disruptions of processes during the first half of pregnancy (eg, neuronal progenitor cell proliferation, placental development) might also affect foundational aspects of neurodevelopment, too few pregnancies had antiseizure drug exposure only during the first half to isolate the effect of early pregnancy exposure. Our primary definition (at least one antiseizure drug prescription filled in the second half of pregnancy) does not isolate second half only exposure because most pregnancies with exposure in the second half also had exposure in the first half (eTable 2).

After requiring a minimum of 200 pregnancies with exposure to the individual antiseizure drug, the following antiseizure drugs were considered for analysis: carbamazepine, lacosamide, lamotrigine, levetiracetam, oxcarbazepine, phenobarbital, phenytoin, topiramate, valproate, and zonisamide (eTable 1, eTable 2). Valproate—given its well established association with NDDs^9^ ^19^ ^22^ ^23^ ^24^ ^25^ ^26^ ^27^ ^28^ ^29^ ^30^ ^31^ ^32^ ^33^ ^34^ ^35^ ^36^ ^37^ ^38^ ^39^ ^40^ ^41^—served as a positive control exposure, whereas lamotrigine—generally not linked to substantial NDD risk increases^9^ ^19^ ^22^ ^23^ ^24^ ^35^ ^36^ ^37^ ^38^ ^41^ ^42^ ^43^ ^44^ ^45^ ^46^ ^47^ ^48^ ^49^—served as a negative control. Gabapentin and pregabalin were not evaluated because these drugs are commonly used for neuropathic pain rather than primarily for epilepsy treatment.^62^ Children who were unexposed prenatally (the reference group) were those born to mothers with no antiseizure drug dispensation from three months before pregnancy until birth (ie, women with presumed inactive or pharmacologically untreated epilepsy).

### Neurodevelopmental disorders

We focused on the most commonly diagnosed NDDs in the US,^63^ ^64^ ^65^ which we defined using validated algorithms with high positive predictive values^54^: ADHD, ASD, behavioral disorder, developmental coordination disorder, intellectual disability, learning disability, speech or language disorder, and any NDD, defined as the presence of any specific NDD considered (see eTable 3 for a description of the algorithms, corresponding diagnostic codes, and positive predictive values).

### Covariates

We considered a broad range of potential confounders, including personal characteristics (ie, maternal age, race or ethnicity (available for MAX/TAF only), US region, calendar year), maternal mental health or developmental conditions (eg, anxiety, bipolar disorder, depression, migraine or headache, ADHD, intellectual disability), healthcare use markers (eg, number of outpatient visits), substance use (eg, alcohol use disorder), other prescription drug exposure (eg, antidepressants, antipsychotics, opioids), and other comorbidities (eg, pregestational diabetes, hypertension; eTable 4).

### Analyses

We compared patient characteristics between the respective exposure and reference groups, using standardized mean differences. An absolute standardized mean difference <10% was considered well balanced.^66^ Distribution of characteristics and balance were assessed separately for each cohort.

Propensity scores^67^were estimated using logistic regression including all prespecified covariates. Propensity score overlap weights were used for confounding control.^68^ Crude and propensity score overlap weighted cumulative incidences for each NDD of interest stratified by exposure were assessed using Kaplan-Meier analyses, and crude and weighted hazard ratios were calculated using Cox proportional hazard models. For the calculation of cumulative incidences and hazard ratios, data from both cohorts were combined by pooling at the individual pregnancy level, and the respective cohort was included as a binary indicator variable in the logistic regression model used to estimate the propensity score.

Several sensitivity analyses were conducted. We stratified analyses by monotherapy versus polytherapy, with monotherapy defined as exposure to only one individual antiseizure drug during the second half of pregnancy, and polytherapy defined as concomitant use of more than one antiseizure drug during this period (excluding women additionally using valproate given its well known neurotoxic effect). Because women taking only one antiseizure drug during the second half of pregnancy might have been using a different antiseizure drug during the first half—which may also represent a window of neurodevelopmental vulnerability—we also required no exposure to any antiseizure drug other than the antiseizure drug of interest at any time in pregnancy (strict monotherapy). To address potential exposure misclassification, we required at least two dispensations of the antiseizure drug of interest during the second half under the assumption that women who refill their prescription might be more likely to take the drug as prescribed. Because women with untreated epilepsy during pregnancy may differ substantially from those who receive antiseizure drugs, we conducted a comparative safety analysis using lamotrigine monotherapy as the reference group given its common use and previous literature suggesting no strong neurotoxicity. To preserve cohort size, and therefore precision, we accounted for epilepsy through inclusion in the propensity score rather than restricting the cohort to those with a recorded diagnosis.

For each pregnancy exposed to a specific antiseizure drug during the second half, we identified the prescription with the highest daily dose. We then calculated the distribution of these maximum daily doses across all such pregnancies and used the median as the threshold to classify exposures as low or high dose. We also explored whether an observed risk increase could be partially driven by hereditary epilepsy. Children of mothers with certain epilepsy types may inherit a predisposition to epilepsy and NDDs (eg, intellectual disability), which share biological pathways.^69^ ^70^ ^71^ If genetic predispositions differ by epilepsy treatment (eg, owing to variations in maternal epilepsy type and severity), residual confounding by hereditary epilepsy is possible. Therefore, we conducted an analysis where NDDs were only considered present if the child did not receive an epilepsy diagnosis during follow-up. Finally, given the potential for selection bias owing to informative censoring, we conducted a sensitivity analysis in which we combined the propensity score overlap weights with stabilized inverse probability of censoring weights, calculated using the same baseline covariates as the propensity score model, to jointly address confounding and informative censoring.^72^ ^73^ ^74^

In exploratory analyses, we evaluated alternative exposure windows and definitions. Given that the first half of pregnancy might also affect certain neurodevelopmental processes, we examined exposure during the first half of pregnancy (irrespective of exposure thereafter), and any time in pregnancy. Additionally, for each exposure window, we restricted the analysis to strict monotherapy, which was defined as no exposure to any other antiseizure drug at any time in pregnancy.

All analyses were conducted from October 2023 to August 2025 using SAS 9.4 (SAS Institute). No adjustments were made for multiple testing.^75^ When interpreting the results, we focused on the magnitude and precision of the effect estimates and the consistency of results across main and sensitivity analyses, rather than dichotomizing P values into statistically significant and non-significant.^76^ The study was approved by the institutional review board of Brigham and Women’s Hospital, which waived the need for informed consent. This study followed the Strengthening the Reporting of Observational Studies in Epidemiology (STROBE) reporting guidelines for observational studies (eAppendix 2).

### Patient and public involvement

No patients were involved in setting the research question or the outcome measures, nor were they involved in developing plans for study design or implementation. No patients were asked to advise on interpretation or writing up of results. 

## Results

### Cohort characteristics

The full cohorts (before epilepsy restriction) included 2 454 550 publicly (MAX/TAF) and 1 854 201 commercially (MarketScan) insured mother-child linked pregnancies. After restricting the cohorts to those with recorded epilepsy, there were 7245 unexposed pregnancies and 10 345 pregnancies with at least one antiseizure drug dispensation in the second half of pregnancy in MAX/TAF. Respective numbers were 1642 and 4648 in MarketScan. Among individual antiseizure drugs (monotherapy or polytherapy), exposure counts ranged from 150 (lacosamide) to 3728 (levetiracetam) in MAX/TAF, and from 69 (lacosamide) to 1976 (lamotrigine) in MarketScan (eFigure 1).

**Fig 1:**
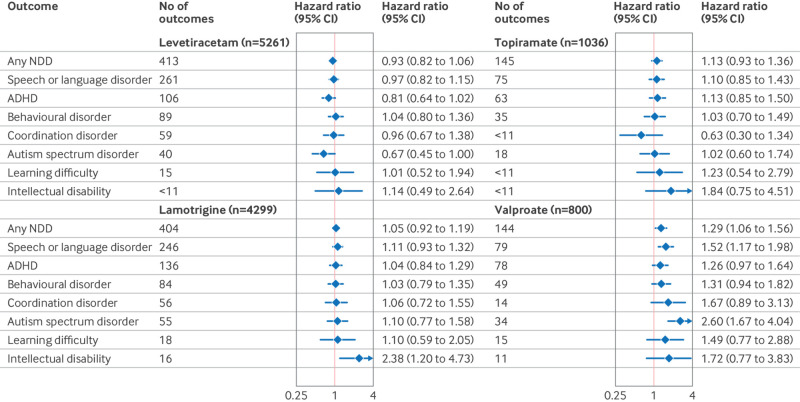
Selected patient characteristics among publicly (MAX/TAF) insured pregnant women with exposure to specific antiseizure drug of interest (based on at least one dispensation in second half of pregnancy, monotherapy or polytherapy) compared with unexposed pregnancies within the epilepsy restricted cohort. Individual drugs are sorted by number of exposed pregnancies in both cohorts combined (from left (highest) to right (lowest)). Patient characteristics are presented as column % unless stated otherwise. Standardized mean difference (SMD) was used to assess balance of covariates between respective exposure group and unexposed reference. For continuous variables, SMD is calculated as (x̄_treatment − x̄_control) / √((s_treatment² + s_control²)/2), where x̄ denotes sample mean and s denotes standard deviation of covariate. For binary or categorical variables, standardized mean difference is calculated as (p̂_treatment − p̂_control) / √((p̂_treatment(1 − p̂_treatment) + p̂_control(1 − p̂_control)) / 2), where p̂ denotes prevalence of variable in treatment and control groups, respectively.^66^ Absolute SMDs <0.1 were considered well balanced. *Race or ethnicity information was only available for publicly (MAX/TAF) insured pregnant women but not for commercially (MarketScan) insured study cohort. Information based on data submitted to Centers for Medicare & Medicaid Services by each state from collected and coded Medicaid applications. Category unknown or other included American Indian/Alaska Native, Native Hawaiian/other Pacific Islander, Hispanic/Latino, more than one race, and unknown; category Hispanic or Latino included this ethnicity with missing race information, whereas unknown or other included Hispanic or Latino with one or more races. ADHD=attention deficit hyperactivity disorder; MAX/TAF=Medicaid Analytic eXtract/Transformed Medicaid Statistical Information System Analytic Files; SD=standard deviation

**Fig 2:**
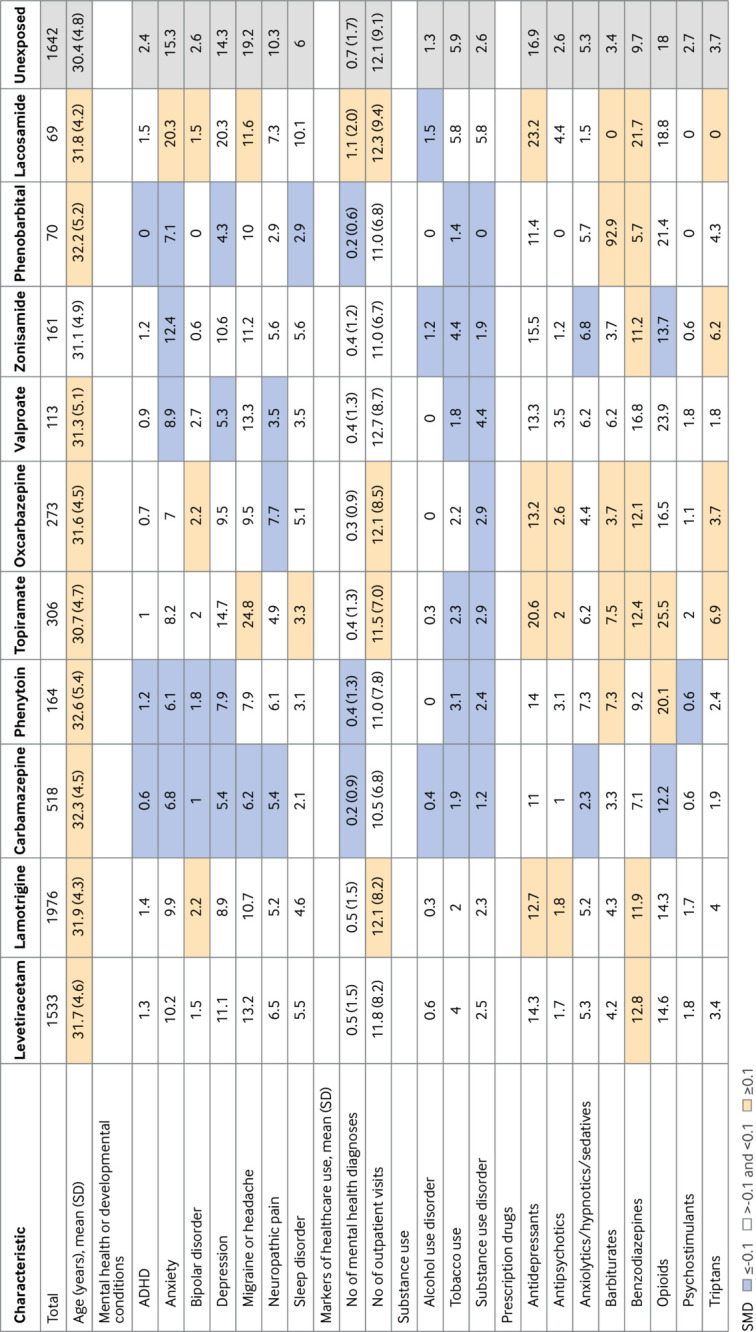
Selected patient characteristics among commercially (MarketScan) insured pregnant women with exposure to specific antiseizure drug of interest (based on at least one dispensation in second half of pregnancy, monotherapy or polytherapy) compared with unexposed pregnancies within the epilepsy restricted cohort. Individual drugs are sorted by number of exposed pregnancies in both cohorts combined (from left (highest) to right (lowest)). Patient characteristics are presented as column % unless stated otherwise. Standardized mean difference (SMD) was used to assess balance of covariates between respective exposure group and unexposed reference. For continuous variables, SMD is calculated as (x̄_treatment − x̄_control) / √((s_treatment² + s_control²)/2), where x̄ denotes sample mean and s denotes standard deviation of covariate. For binary or categorical variables, SMD is calculated as (p̂_treatment − p̂_control) / √((p̂_treatment(1 − p̂_treatment) + p̂_control(1 − p̂_control)) / 2), where p̂ denotes prevalence of variable in treatment and control groups, respectively.^66^ Absolute SMDs <0.1 were considered well balanced. ADHD=attention deficit hyperactivity disorder; MarketScan=Merative MarketScan Commercial Claims and Encounters Database; SD=standard deviation

Patient characteristics among those taking levetiracetam or lamotrigine (the most common antiseizure drugs in both cohorts) were similar to the unexposed group. More imbalances were observed for other antiseizure drugs, with women who had exposed pregnancies generally more likely to be older and white (MAX/TAF), to have more outpatient visits and more concomitant drug use, and less likely to use tobacco and to have substance use disorders. Propensity score overlap weighting resulted in perfect balance (with standardized mean differences equal to zero, as per the definition^68^) for all covariates included in the propensity score model (fig 1, fig 2, eTable 5). Average length of follow-up of children was 3.4 years; of the 23 880 children eligible at birth (born to mothers with epilepsy), 5505 were followed for at least five years and 2516 for at least eight years after birth (the distribution of follow-up by database and antiseizure drug of interest is shown in eTable 6).

### Cumulative incidence and hazard ratio of NDDs

**Fig 3:**
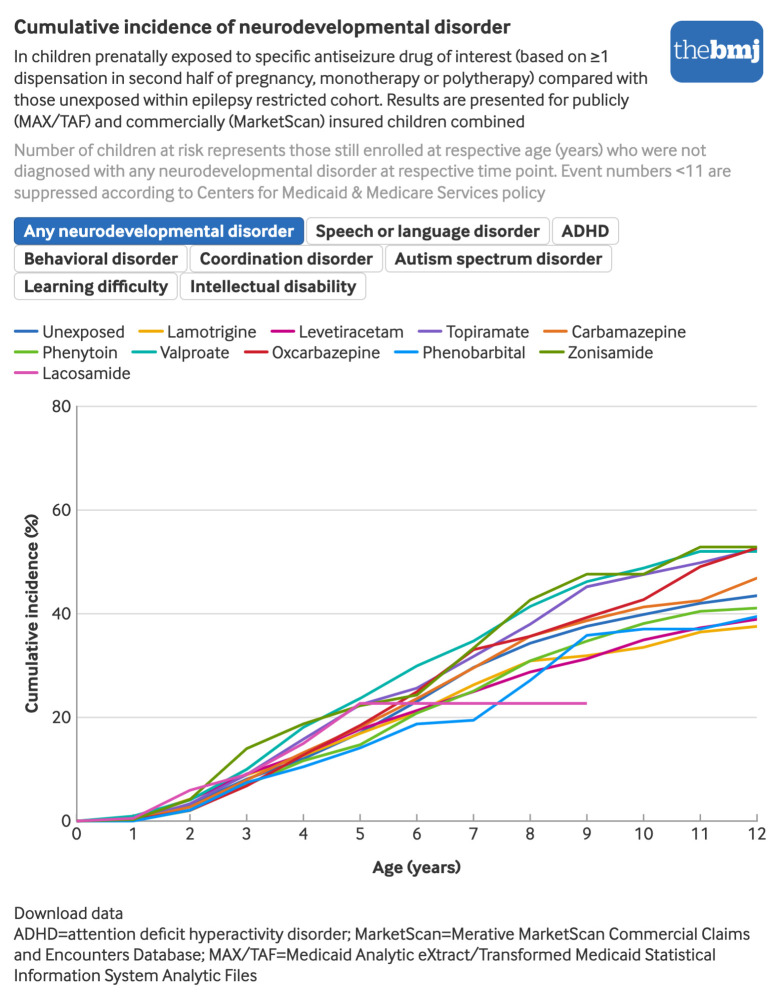
Cumulative incidence of neurodevelopmental disorder in children prenatally exposed to specific antiseizure drug of interest (based on at least one dispensation in second half of pregnancy, monotherapy or polytherapy) compared with those unexposed within epilepsy restricted cohort. An interactive version of this graphic and downloadable data are available at https://public.flourish.studio/visualisation/27664020/

After combining both cohorts, cumulative incidence of any NDD by 8 years—when most NDDs are expected to have been diagnosed—was 34.3% (95% confidence interval (CI) 32.0% to 36.7%) among children unexposed prenatally and ranged from 22.7% (12.2% to 39.8%) for those exposed to lacosamide to 42.6% (31.9% to 55.3%) for those exposed to zonisamide. Estimates did not change substantially after propensity score weighting (fig 3, eTable 6).

For any NDD, weighted hazard ratios indicated a potential risk increase for valproate (hazard ratio 1.29, 95% CI 1.06 to 1.56) and zonisamide (1.41, 1.06 to 1.88), while no meaningful risk increase was observed for the other drugs.

Several antiseizure drugs were associated with an increased risk of intellectual disability: hazard ratio of 2.38 (95% CI 1.20 to 4.73) for lamotrigine, 1.84 (0.75 to 4.51) for topiramate, 3.30 (1.29 to 8.41) for oxcarbazepine, 1.72 (0.77 to 3.83) for valproate, 4.50 (1.43 to 14.18) for zonisamide, and 3.70 (0.85 to 16.11) for phenobarbital.

Valproate was associated with an increased risk of all specific NDDs, whereas lamotrigine was associated with an increased risk of intellectual disability only, with no evidence of increased risk of other NDDs.

**Fig 4:**
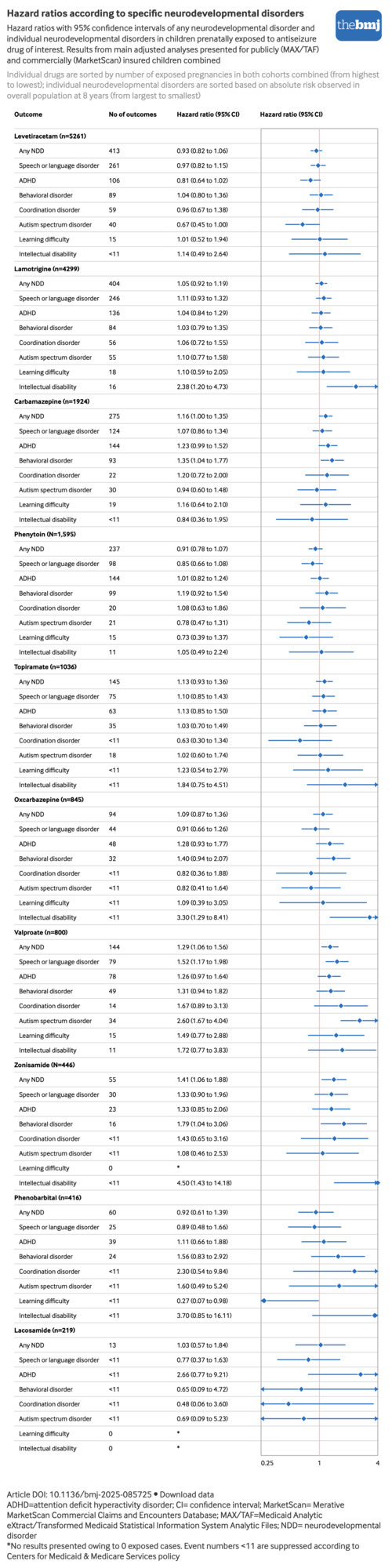
Hazard ratios (95% confidence intervals) of any neurodevelopmental disorder and individual neurodevelopmental disorders in children prenatally exposed to specific antiseizure drug of interest. An interactive version of this graphic and downloadable data are available at https://public.flourish.studio/visualisation/27665062/

Levetiracetam and phenytoin were not associated with any outcome. Topiramate only showed an association with intellectual disability, and potentially learning difficulty, yet with a wide confidence interval (hazard ratio 1.23, 95% CI 0.54 to 2.79). Carbamazepine and oxcarbazepine showed a moderate increased risk of ADHD and behavioral disorder (hazard ratio range 1.23-1.40). Zonisamide was associated with an increased risk of most individual NDDs except for ASD (fig 4), but associations were imprecise owing to the relatively small number of exposed pregnancies (n=446). No children prenatally exposed to zonisamide were found to have learning difficulties, which is not unexpected given that these conditions are diagnosed at older ages when very few of those exposed to zonisamide were still under observation. Data for specific NDDs were too sparse for phenobarbital and lacosamide to be informative, but no consistent patterns emerged (fig 4, eTable 6).

### Sensitivity analyses

Because women tended to only take one individual antiseizure drug (frequency of monotherapy exposure ranged from 50% for zonisamide to 76% for carbamazepine; eTable 1), the results for those receiving monotherapy were generally consistent with the main analyses. For example, for carbamazepine, the hazard ratio for ADHD was 1.23 (95% CI 0.99 to 1.52) in the main analysis, 1.27 (1.00 to 1.60) for monotherapy, and 1.26 (0.98 to 1.62) for strict monotherapy. For some comparisons (eg, when monotherapy was less common), risk estimates attenuated, although not consistently and with wider confidence intervals given the smaller number of exposed pregnancies. For instance, for zonisamide, the hazard ratio for any NDD was 1.41 (1.06 to 1.88) in the main analysis, 1.31 (0.88 to 1.95) for monotherapy, and 1.15 (0.73 to 1.81) for strict monotherapy. In contrast, when restricting the analysis to those exposed to polytherapy and requiring at least two dispensations, the risk increases observed in the main analyses tended to slightly strengthen, but also with considerably less precision (eg, hazard ratio for any NDD after zonisamide exposure was 1.56 (1.05 to 2.30) for polytherapy and 1.55 (1.14 to 2.11) for at least two dispensations).

**Fig 5:**
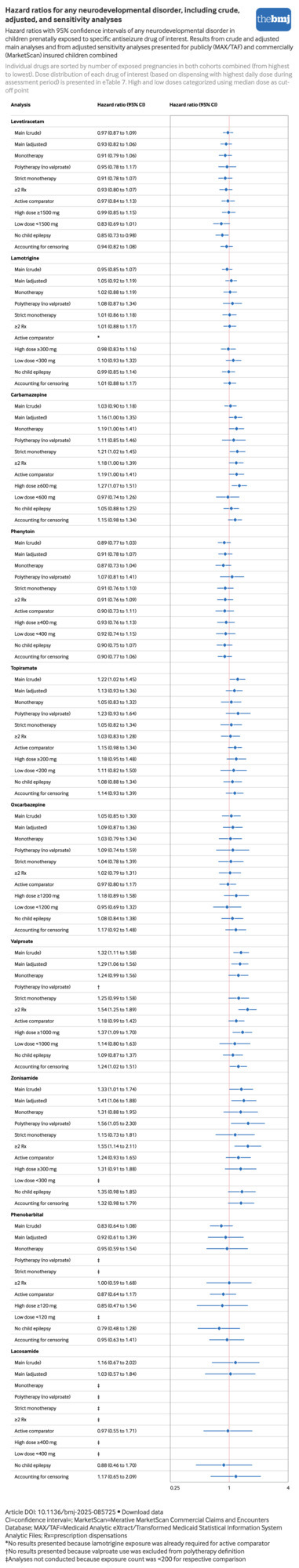
Hazard ratios (95% confidence intervals) of any neurodevelopmental disorder in children prenatally exposed to specific antiseizure drug of interest, including crude, adjusted, and sensitivity analyses. An interactive version of this graphic and downloadable data are available at https://public.flourish.studio/visualisation/27665466/

When we used lamotrigine monotherapy as the active comparator, risks shifted only marginally closer to the null. Findings further pointed towards a dose-response relation for those associations observed in the main analysis. For intellectual disability, estimates were too imprecise to draw any conclusion about a dose response. Restricting the analysis to NDDs without co-occurrence of childhood epilepsy only marginally changed the relative risk estimates, with the potential exception of valproate, when estimates were attenuated, but with considerably more imprecision. Accounting for censoring weights yielded similar results to the main analyses (fig 5, eTable 6)

### Exploratory analyses

When using alternative exposure windows, point estimates were generally similar to those for second half exposure but tended to be slightly closer to the null. Results were also consistent when restricting the analysis to monotherapy during these alternative exposure windows, albeit with wide confidence intervals (eFigure 3).

## Discussion

### Principal findings

With many women requiring antiseizure drug treatment during pregnancy, concerns exist about the potential neurodevelopmental impact on the offspring, yet data for many antiseizure drugs remain sparse. This large cohort study contributes evidence on the neurodevelopmental risks after prenatal antiseizure drug exposure. The observed risk increase for all NDDs after valproate exposure aligns with previously described fetal neurotoxic effects of the drug. For levetiracetam, lamotrigine, phenytoin, and topiramate, our findings indicate no substantial risk increase. One potential exception is intellectual disability, for which a risk increase was observed for lamotrigine and topiramate, as well as for oxcarbazepine, valproate, zonisamide, and phenobarbital. However, findings were based on few exposed events because intellectual disability is the least common of the NDDs studied, and residual confounding owing to genetic factors cannot be completely ruled out (given the shared cause of some epilepsy types and intellectual disability, and the impact of epilepsy on cognitive function). Nevertheless, continued monitoring is warranted as more data accumulate given the severity and lifelong functional implications of intellectual disability.

We observed moderate risk increases for ADHD and behavioral disorder after exposure to carbamazepine and its structural analogue oxcarbazepine, which were consistent across sensitivity analyses. This finding is important because current guidelines recommend oxcarbazepine (as well as lamotrigine and levetiracetam) owing to its generally low risk of major congenital malformations, whereas NDD safety data for oxcarbazepine are currently lacking.^53^ Continued monitoring of this drug is therefore needed. Our findings raise potential concerns for zonisamide, which was associated with several NDDs in the main analysis. However, these estimates are based on small numbers and should be interpreted with caution.

For some comparisons, including zonisamide, we observed attenuation of risk when restricting the analysis to monotherapy. Because polytherapy regimens most often involved lamotrigine or levetiracetam—both generally considered safe in pregnancy and also showing no substantial increased risks in our study—the excess risk seen with polytherapy may reflect not only the additional drug burden but also underlying differences in epilepsy severity or type that we could not fully explore. Future work is warranted to disentangle these mechanisms and better characterize the impact of polytherapy on NDD risk. Data for phenobarbital and lacosamide were too sparse to draw firm conclusions.

### Comparison with other studies

A recent exploratory Nordic cohort study assessed the association between commonly used antiseizure drugs and a spectrum of 13 psychiatric disorders in children born to mothers with epilepsy.^23^ This large study was conducted in a pregnant population with epilepsy and focused on the safety of individual antiseizure drugs used as monotherapy with specific neurodevelopmental and psychiatric outcomes. Exposure was defined based on maternal prescription fills from 30 days before the last menstrual period until birth. Although many outcomes considered differed from those evaluated in the present study, the Nordic study did include ADHD, ASD, and intellectual disability. For these outcomes, findings from the Nordic cohort are consistent with ours for valproate (increased risks) and lamotrigine (no substantial risk increase), reinforcing the established evidence. The potential signals for ADHD after levetiracetam and topiramate exposure observed in the Nordic study were not confirmed in our analyses. Similarly, an increased risk of ASD after topiramate as seen in the Nordic study was not found in our study, as we previously reported in a study focused on topiramate and ASD using the same US data source.^24^ By contrast, other than for valproate and potentially topiramate, no increased risk of intellectual disability was observed in the Nordic study. Similarly, unlike our study, the Nordic study did not find increased risk of ADHD, oppositional defiant disorder, and conduct disorder after carbamazepine and oxcarbazepine exposure.

Another recently published study from the UK and Sweden (with Swedish data overlapping those included in the Nordic study) also focused on the risk of ADHD, ASD, and intellectual disability after prenatal exposure to individual antiseizure drugs.^52^ The findings for the epilepsy restricted cohort were consistent with ours with regard to risk increases for all outcomes after valproate and for intellectual disability after topiramate exposure. By contrast, the authors observed risk increases for ASD after topiramate exposure and intellectual disability after carbamazepine exposure, which were not seen in our study.

Several important differences exist between these recent studies and our study, making a direct comparison of findings challenging. For example, in the Nordic study, exposure to individual antiseizure drugs was assessed based on monotherapy at any time between 30 days before pregnancy and delivery, in contrast to our focus on any use (irrespective of exposure to other antiseizure drugs) during the period of synaptogenesis. Overall, however, our results were consistent across alternative exposure definitions, suggesting that the choice of definition is unlikely to account for the differences in findings. We generally required the presence of at least two NDD diagnosis records (with an additional age criterion for most outcomes) informed by our validation study,^54^ whereas the previous studies focused on at least one diagnosis at any age. Study heterogeneity was also noted as a challenge in a recently published narrative synthesis covering evidence through 2023.^77^ Finally, only a few individual drugs—carbamazepine, lamotrigine, levetiracetam, topiramate, and valproate—were evaluated in all three studies, whereas the two more recently approved drugs, zonisamide and lacosamide, as well as phenytoin (an older drug still used in the US but not in Europe^10^) were not included in the European studies.

Based on the findings from the Nordic study and a previous study on prenatal antiseizure drug exposure and risk of ASD and intellectual disability^37^ using the same initial Nordic cohort with a slightly different exposure window, regulatory agencies (like the European Medicines Agency and the UK Medicines and Healthcare products Regulatory Agency) now advise against using topiramate given its potential increased risk of ADHD, ASD, and intellectual disability. In a recent study from our group, no increased risk of ASD could be confirmed for topiramate.^24^ The findings from the current study and the recent study using UK and Swedish data also do not support the hypothesis of an increased risk of ADHD. The potential signal for intellectual disability after topiramate observed in the European studies and the current study, although based on small numbers of cases in all cohorts, requires confirmation in follow-up studies as more data accumulate.

### Potential biological mechanisms

The teratogenic and neurotoxic effects of valproate may arise from shared biological mechanisms, including disruptions in folate metabolism, oxidative stress pathways, dysregulation of neurotransmitter signaling, and neuronal apoptosis.^61^ ^78^ ^79^ ^80^ ^81^ Other antiseizure drugs, such as zonisamide, may act through similar pathways,^82^ ^83^ ^84^ whereas lamotrigine and levetiracetam might lead to less systemic disruption.^85^ ^86^ ^87^ Topiramate has previously not shown an association with neuronal apoptosis in rodents.^88^ Despite such plausible mechanisms, we observed a risk attenuation for valproate—although with greater imprecision—when only including NDDs in children not diagnosed with epilepsy. This finding raises the possibility that some of the risk may be attributable to genetic predisposition or maternal epilepsy type and severity. No similar attenuation was seen for other antiseizure drugs. However, clinical studies, where information on maternal IQ and epilepsy type was available and controlled for, still found lower IQ score and more autistic traits in offspring prenatally exposed to valproate than for those who were unexposed.^19^ ^37^ ^40^ ^44^ ^45^

### Strengths and limitations of this study

Strengths of our study include the use of two large, nationwide databases of publicly and commercially insured pregnant women linked to their children, enhancing the generalizability of our findings and enabling us to assess the risk of specific NDDs associated with individual antiseizure drugs. The study benefited from independent ascertainment of exposure and outcome, a focus on the second half of pregnancy as the period of robust synaptogenesis (we did not restrict analysis to second half only exposure because very few women were exposed exclusively during this period), use of validated outcome measures, and adjustment for a broad range of potential confounders, strengthening the internal validity of our results.

Despite these strengths, our study has several limitations. The reliance on insurance claims data necessitates continuous enrollment of the children, resulting in loss to follow-up and potential bias if censoring is informative. However, despite the substantial loss, the absolute number of children still enrolled several years after birth remained large, particularly for more common antiseizure drugs, and estimates were consistent when accounting for censoring weights, making selection bias unlikely. Exposure misclassification is possible as prescription fills do not guarantee drug ingestion. However, results were consistent when requiring several dispensations. Despite the large cohorts, data for some antiseizure drugs and NDDs (eg, lacosamide, intellectual disability), and some subgroup analyses, remained sparse, resulting in wide confidence intervals for these specific comparisons; such estimates should be interpreted with caution and confirmed in future studies. While we started with a large birth cohort, a substantial number of children were censored over time because the end of the study period was reached or insurance was changed. Although this loss to follow-up does not appear to be informative—as supported by sensitivity analyses accounting for censoring—this reduced sample size at later ages increases imprecision and may limit the ability to detect later onset NDDs, particularly for less common exposures or outcomes.

Although we explored the possibility that observed risk increases could be partially driven by hereditary epilepsy, we did not have sufficient detail in claims data to adequately control for underlying epilepsy type, syndrome, and severity. Therefore, potential residual confounding by such unmeasured maternal epilepsy characteristics cannot be completely ruled out. Finally, we had to rely on dispensed doses to evaluate dose-response relations. Given that drug clearance varies considerably during pregnancy and across women, such doses may not accurately reflect blood concentrations.^89^ Moreover, the dichotomization into high versus low dose might obscure substantial variability within each of these groups. Therefore, the dose-response findings from our study cannot be directly compared with those from the MONEAD study, which evaluated maternal blood concentrations.^42^ ^43^

### Conclusions

Our study reinforces the substantial risks of NDDs associated with prenatal valproate exposure and suggests the need to further evaluate the safety of zonisamide during pregnancy. We did not confirm the previously suggested increase in risk of ADHD and ASD after prenatal topiramate. Data for lacosamide were too limited to draw definitive conclusions, but no consistent pattern has emerged so far. Continued monitoring of newer antiseizure drugs and the few potential signals that emerged (ie, the moderate increased risk of ADHD and behavioral disorder after carbamazepine and oxcarbazepine exposure, and the association of several antiseizure drugs with intellectual disability) will be important.

**What is already known on this topic**

Neurodevelopmental disorders are prevalent conditions affecting a large number of children globally
Antiseizure drugs are frequently and increasingly used by women of childbearing age
Valproate use during pregnancy has been linked to impaired neurodevelopment in children, but information on other antiseizure drugs is limited

**What this study adds**

The findings support previous evidence of increased neurodevelopmental risks among children with prenatal valproate exposure and suggest the need for further evaluation of zonisamide
Continued monitoring of newer antiseizure drugs and the few potential signals that emerged will be important

## Data Availability

No additional data available.

## Web extra

Extra material supplied by authors

Web appendix: Supplementary online content

## Notes

Contributors: LS, SH-D, BTB, and KFH contributed to all the aspects of this study. HM was involved in preparation of analytic datasets and designing the study. YZ, KLW, KJG, BL, CJM, and PBP were involved in designing the study and preparation of the final manuscript. LS was involved in preparation of analytic datasets, designing the study, and preparation of the final manuscript. LS and KFH had full access to all of the data (including statistical reports and tables) in the study and take responsibility for the integrity of the data and the accuracy of the data analysis. LS and KFH are the guarantors. The corresponding author attests that all listed authors meet authorship criteria and that no others meeting the criteria have been omitted.

Transparency: The lead author affirms that the manuscript is an honest, accurate, and transparent account of the study being reported; that no important aspects of the study have been omitted; and that any discrepancies from the study as planned (and, if relevant, registered) have been explained.

Dissemination to participants and related patient and public communities: Because the datasets are anonymized, it is not possible to disseminate the results of the research to study participants.

Provenance and peer review: Provenance and peer review: Not commissioned; externally peer reviewed.
