# Supplementary material for: Prenatal antiseizure drug exposure and risk of neurodevelopmental disorders in children: population based cohort study
Source: BMJ. 2026 Mar 11;392:e085725. doi: 10.1136/bmj-2025-085725 (PMC12978236; doi:10.1136/bmj-2025-085725)
Supplement: Supplementary file 1 — Web appendix: Supplementary online content [file strl085725.ww.pdf]

SUPPLEMENTARY ONLINE CONTENT

Prenatal Antiseizure Drug Exposure and Risk of Neurodevelopmental Disorders in Children: Population Based Cohort Study

Loreen Straub, MD MS; Sonia Hernandez-Diaz MD DrPH; Brian T. Bateman MD MS; Yanmin Zhu, MS PhD; Helen Mogun, MS; Katherine L. Wisner, MD; Kathryn J. Gray, MD PhD; Barry Lester, PhD; Christopher J. McDougle, MD; Page B. Pennell, MD; Krista F. Huybrechts, MS PhD

Supplementary Figures

eFigure 1. Cohort Selection for Pregnancies in the Medicaid Analytic eXtract/Transformed Medicaid Statistical Information System Analytic Files (MAX/TAF, 2000-2018) and the MarketScan Research Database (MarketScan, 2003-2021)........ 2
eFigure 2. Design Diagram for In-Utero Exposure to Antiseizure Medications of Interest During the Second Half of Pregnancy and Risk of Neurodevelopmental Disorders. 4
eFigure 3. Cumulative Incidence in % at Age 8 Years and Hazard Ratios (95% Confidence Intervals) of Any Neurodevelopmental Disorder and Individual Neurodevelopmental Disorders in Children Prenatally Exposed to the Specific Antiseizure Medication of Interest. Results from Adjusted Analyses are Presented for Publicly and Commercially Insured Children Combined. Exposure is Defined Based on (a) Any Use (>=1 Dispensing, Irrespective of Exposure to Other Antiseizure Medications) and (b) Strict Monotherapy (>=1 Dispensing, But no Exposure to Other Antiseizure Medications from LMP-3 Months to Delivery-1 Day), Respectively, Separately for 3 Assessment Periods (i) 2nd Half of Pregnancy, (ii) 1st Half of Pregnancy, and (iii) Any Time in Pregnancy........ 5

Supplementary Tables

eTable 1. Counts of Exposure and Reference Groups for Main and Sensitivity Analyses. 15
eTable 2. Pregnancy Counts with Antiseizure Medication Exposure of Interest by Timing of Exposure During Pregnancy. 16
eTable 3. Definition of Neurodevelopmental Disorders.[1] 17
eTable 4. Full List of Covariates and Corresponding Assessment Periods Included in Propensity Score Models. 18
eTable 5. Patient Characteristics Among Publicly (MAX/TAF) and Commercially (MarketScan) Insured Women Exposed to the Specific Antiseizure Medication of Interest (Based on >=1 Dispensation in the Second Half of Pregnancy) Compared with Unexposed Pregnancies within the Epilepsy-Restricted Cohort. 21
eTable 6. Length of Follow-Up in Years Since Birth in Children Born to Mothers with Epilepsy, who were Prenatally Exposed to the Specific Antiseizure Medication of Interest Compared to Those Unexposed........ 53
eTable 7. Cumulative Incidence in % at Age 8 Years and Hazard Ratios (95% Confidence Intervals) of Any Neurodevelopmental Disorder and Individual Neurodevelopmental Disorders in Children Prenatally Exposed to the Specific Antiseizure Medication of Interest. Results from Crude and Adjusted Main Analyses and from Adjusted Sensitivity Analyses are Presented for Publicly and Commercially Insured Children Combined. 55
eTable 8. Dose Distribution (in mg) of Each Medication of Interest Based on the Dispensing with the Highest Daily Dose During the Second Half of Pregnancy. 74

Appendix

eAppendix 1. Internal Validation of Algorithm to Identify Epilepsy. 75
eAppendix 2. STROBE Statement—Checklist Of Items That Should be Included in Reports of Cohort Studies........ 78

eFigure 1. Cohort Selection for Pregnancies in the Medicaid Analytic eXtract/Transformed Medicaid Statistical Information System Analytic Files (MAX/TAF, 2000-2018) and the MarketScan Research Database (MarketScan, 2003-2021).

MAX/TAF 2000-2018

2,522,490 mother-child linked pregnancies with maternal enrollment between 3 months before LMP to 1 month after delivery

2,504,900 pregnancies were excluded (in hierarchical order):
- 5,188 with chromosomal anomalies[a]
- 13,994 without linkage to county-level SES variables[b]
- 48,758 with ≥1 ASM[c] dispensing within 3 months before LMP to end of first half of pregnancy, but not during second half of pregnancy
- 2,436,960 without epilepsy[d]

17,590 pregnancies were evaluated:
- 7,245 without any ASM exposure within 3 months before LMP and throughout pregnancy
- 10,345 with ≥1 ASM dispensing during second half of pregnancy; N by individual medication (not mutually exclusive):
  - 3,728: levetiracetam
  - 2,323: lamotrigine
  - 1,406: carbamazepine
  - 1,431: phenytoin
  - 730: topiramate
  - 572: oxcarbazepine
  - 687: valproate
  - 285: zonisamide
  - 346: phenobarbital
  - 150: lacosamide

MarketScan 2003-2021

1,875,849 mother-child linked pregnancies with maternal enrollment between 3 months before LMP to 1 month after delivery

1,869,559 pregnancies were excluded (in hierarchical order):
- 6,107 with chromosomal anomalies[a]
- 15,541 with ≥1 ASM[c] dispensing within 3 months before LMP to end of first half of pregnancy, but not during second half of pregnancy
- 1,847,911 without epilepsy[d]

6,290 pregnancies were evaluated:
- 1,642 without any ASM exposure within 3 months before LMP and throughout pregnancy
- 4,648 with ≥1 ASM dispensing during second half of pregnancy; N by individual medication (not mutually exclusive):
  - 1,533: levetiracetam
  - 1,976: lamotrigine
  - 518: carbamazepine
  - 164: phenytoin
  - 306: topiramate
  - 273: oxcarbazepine
  - 113: valproate
  - 161: zonisamide
  - 70: phenobarbital
  - 69: lacosamide

Abbreviations: ASM, antiseizure medication; LMP, last menstrual period; MarketScan, Merative MarketScan Commercial Claims and Encounters Database; MAX/TAF, Medicaid Analytic eXtract/Transformed Medicaid Statistical Information System Analytic Files; N, number.

Note: While pregnancies with ASM exposure only during the first half of pregnancy were excluded from the main analysis, they were included in exploratory analyses where alternative exposure windows (i.e., exposure during the first half of pregnancy, and at any time of pregnancy) were evaluated. Similarly, while patients without recorded epilepsy were

excluded from the main analysis, they were included in a sensitivity analysis where patients exposed to lamotrigine monotherapy were considered as the reference group.

[a] Chromosomal anomalies are based on diagnostic coding out to 90 days from child birth.

[b] The MAX/TAF pregnancy cohort was merged with county-level data on various socioeconomic status measures provided by the US Department of Agriculture, after linkage of maternal zip codes with corresponding Federal Information Processing System county codes using crosswalk files provided by the US Department of Housing and Urban Development. A small proportion of pregnancies could not get linked to county-level data due to missing/incomplete zip code information.

Source:

US Department of Agriculture's Economic Research Service. County-level data sets. Accessed March 18, 2020. https://www.ers.usda.gov/dataproducts/county-level-data-sets/download-data/

US Department of Housing and Urban Development's Office of Policy Development and Research. HUD-USPS ZIP Crosswalk Files. Accessed March 18, 2020. https://www.huduser.gov/portal/datasets/usps_crosswalk.html#data

[c] Antiseizure medications included levetiracetam, lamotrigine, carbamazepine, phenytoin, topiramate, oxcarbazepine, valproate, gabapentin, zonisamide, phenobarbital, lacosamide, pregabalin, ethosuximide, acetazolamide, primidone, felbamate, tiagabine, eslicarbazepine, brivaracetam, perampanel, fosphenytoin, mephobarbital, methsuximide, cannabidiol, cenobamate, ethotoin, ezogabine, fenfluramine, mephenytoin, rufinamide, stiripentol, vigabatrin.

[d] For algorithm used to identify individuals with epilepsy, see eAppendix.

eFigure 2. Design Diagram for In-Utero Exposure to Antiseizure Medications of Interest During the Second Half of Pregnancy and Risk of Neurodevelopmental Disorders.

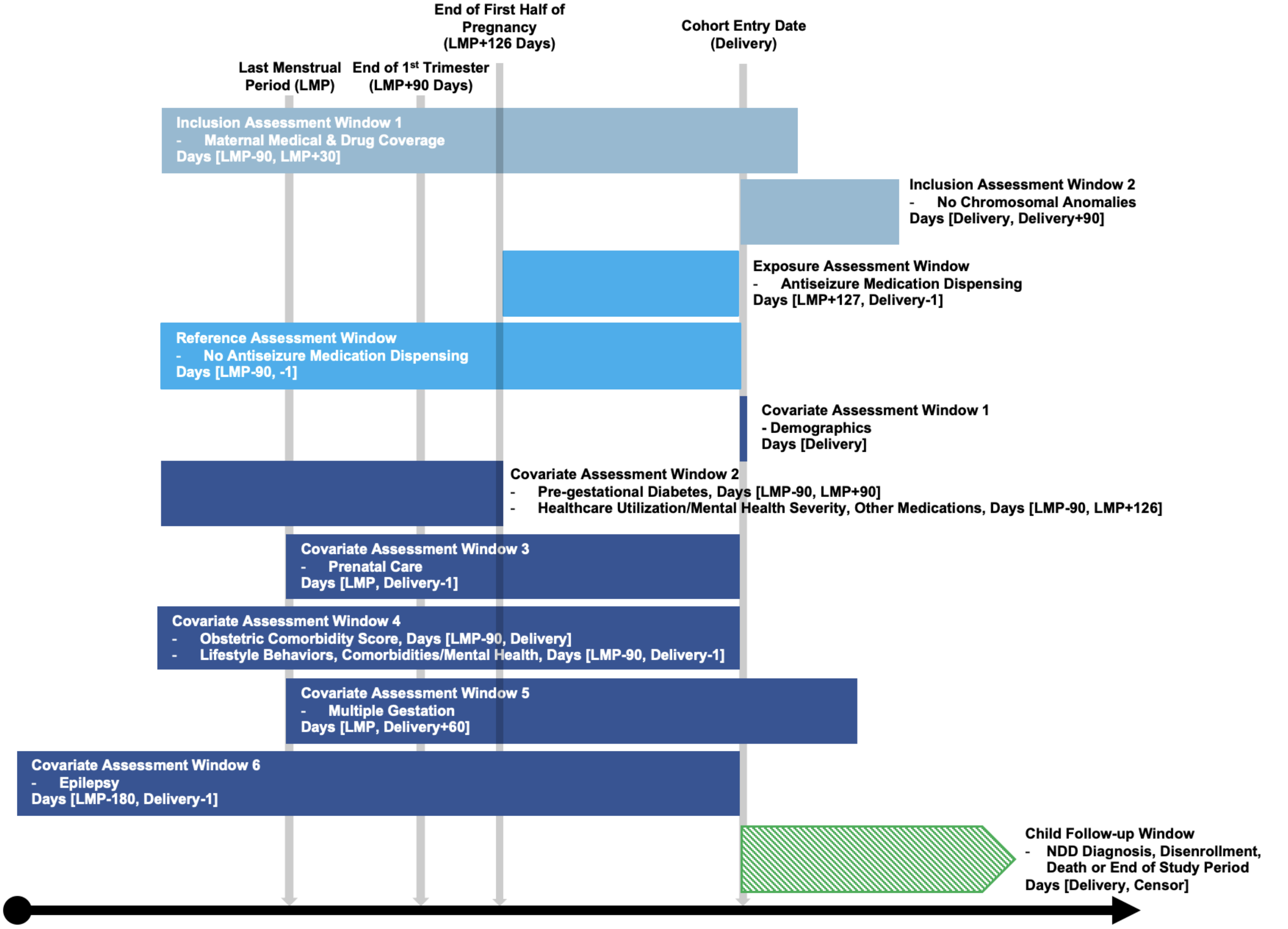

Abbreviations: LMP, last menstrual period; NDD, neurodevelopmental disorder.

eFigure 3. Cumulative Incidence in % at Age 8 Years and Hazard Ratios (95% Confidence Intervals) of Any Neurodevelopmental Disorder and Individual Neurodevelopmental Disorders in Children Prenatally Exposed to the Specific Antiseizure Medication of Interest. Results from Adjusted Analyses are Presented for Publicly and Commercially Insured Children Combined. Exposure is Defined Based on (a) Any Use (>=1 Dispensing, Irrespective of Exposure to Other Antiseizure Medications) and (b) Strict Monotherapy (>=1 Dispensing, But no Exposure to Other Antiseizure Medications from LMP-3 Months to Delivery-1 Day), Respectively, Separately for 3 Assessment Periods (i) 2nd Half of Pregnancy, (ii) 1st Half of Pregnancy, and (iii) Any Time in Pregnancy.

A) Levetiracetam

Outcome	Exposure	Analysis	N Cases / Total		Cumulative Incidence in % (95% CI) at Age 8 Years		HR (95% CI)
			Among Exposed	Among Unexposed	Among Exposed	Among Unexposed	
Any NDD	levetiracetam	2nd Pregnancy Half (Any Use)	413 / 5261	872 / 8887	29.46 (26.14 - 33.09)	33.78 (31.09 - 36.62)	0.93 (0.82 - 1.06)
Any NDD	levetiracetam	1st Pregnancy Half (Any Use)	394 / 4890	872 / 8887	31.11 (27.55 - 35.00)	34.29 (31.56 - 37.18)	0.93 (0.82 - 1.06)
Any NDD	levetiracetam	Any Time in Pregnancy (Any Use)	480 / 6162	872 / 8887	30.43 (27.24 - 33.90)	34.12 (31.49 - 36.90)	0.91 (0.81 - 1.03)
Any NDD	levetiracetam	2nd Pregnancy Half (Strict Monotherapy)	232 / 3360	872 / 8887	27.65 (23.44 - 32.44)	32.84 (29.96 - 35.92)	0.91 (0.78 - 1.07)
Any NDD	levetiracetam	1st Pregnancy Half (Strict Monotherapy)	216 / 3045	872 / 8887	29.91 (25.36 - 35.06)	33.25 (30.31 - 36.40)	0.92 (0.78 - 1.08)
Any NDD	levetiracetam	Any Time in Pregnancy (Strict Monotherap	271 / 3929	872 / 8887	29.18 (25.10 - 33.77)	33.22 (30.43 - 36.20)	0.91 (0.79 - 1.06)
Speech/Language Disorder	levetiracetam	2nd Pregnancy Half (Any Use)	261 / 5261	456 / 8887	14.99 (12.83 - 17.48)	15.80 (14.07 - 17.71)	0.97 (0.82 - 1.15)
Speech/Language Disorder	levetiracetam	1st Pregnancy Half (Any Use)	241 / 4890	456 / 8887	15.14 (12.93 - 17.69)	16.10 (14.34 - 18.05)	0.95 (0.80 - 1.13)
Speech/Language Disorder	levetiracetam	Any Time in Pregnancy (Any Use)	296 / 6162	456 / 8887	14.56 (12.60 - 16.80)	15.84 (14.15 - 17.71)	0.94 (0.80 - 1.10)
Speech/Language Disorder	levetiracetam	2nd Pregnancy Half (Strict Monotherapy)	151 / 3360	456 / 8887	14.13 (11.56 - 17.22)	15.65 (13.82 - 17.70)	0.96 (0.78 - 1.17)
Speech/Language Disorder	levetiracetam	1st Pregnancy Half (Strict Monotherapy)	134 / 3045	456 / 8887	14.92 (12.08 - 18.35)	16.04 (14.16 - 18.15)	0.92 (0.74 - 1.13)
Speech/Language Disorder	levetiracetam	Any Time in Pregnancy (Strict Monotherap	174 / 3929	456 / 8887	14.50 (12.06 - 17.39)	15.74 (13.96 - 17.73)	0.96 (0.79 - 1.16)
ADHD	levetiracetam	2nd Pregnancy Half (Any Use)	106 / 5261	366 / 8887	14.48 (11.71 - 17.83)	17.94 (15.58 - 20.61)	0.81 (0.64 - 1.02)
ADHD	levetiracetam	1st Pregnancy Half (Any Use)	102 / 4890	366 / 8887	15.39 (12.39 - 19.02)	18.09 (15.71 - 20.79)	0.80 (0.63 - 1.01)
ADHD	levetiracetam	Any Time in Pregnancy (Any Use)	129 / 6162	366 / 8887	15.41 (12.73 - 18.60)	18.29 (15.97 - 20.89)	0.82 (0.66 - 1.02)
ADHD	levetiracetam	2nd Pregnancy Half (Strict Monotherapy)	57 / 3360	366 / 8887	14.15 (10.55 - 18.85)	16.97 (14.45 - 19.86)	0.87 (0.64 - 1.17)
ADHD	levetiracetam	1st Pregnancy Half (Strict Monotherapy)	54 / 3045	366 / 8887	15.08 (11.14 - 20.23)	16.87 (14.34 - 19.79)	0.90 (0.66 - 1.22)
ADHD	levetiracetam	Any Time in Pregnancy (Strict Monotherap	69 / 3929	366 / 8887	15.16 (11.63 - 19.65)	17.29 (14.84 - 20.09)	0.89 (0.68 - 1.17)
Behavioral Disorder	levetiracetam	2nd Pregnancy Half (Any Use)	89 / 5261	239 / 8887	9.49 (7.39 - 12.14)	9.66 (8.06 - 11.55)	1.04 (0.80 - 1.36)
Behavioral Disorder	levetiracetam	1st Pregnancy Half (Any Use)	91 / 4890	239 / 8887	9.80 (7.64 - 12.52)	9.89 (8.25 - 11.84)	1.10 (0.85 - 1.44)
Behavioral Disorder	levetiracetam	Any Time in Pregnancy (Any Use)	112 / 6162	239 / 8887	10.09 (8.05 - 12.61)	9.92 (8.34 - 11.78)	1.09 (0.85 - 1.38)
Behavioral Disorder	levetiracetam	2nd Pregnancy Half (Strict Monotherapy)	53 / 3360	239 / 8887	8.88 (6.23 - 12.58)	8.93 (7.28 - 10.92)	1.17 (0.85 - 1.61)
Behavioral Disorder	levetiracetam	1st Pregnancy Half (Strict Monotherapy)	54 / 3045	239 / 8887	9.26 (6.69 - 12.73)	9.02 (7.33 - 11.07)	1.31 (0.96 - 1.80)
Behavioral Disorder	levetiracetam	Any Time in Pregnancy (Strict Monotherap	66 / 3929	239 / 8887	9.38 (6.86 - 12.76)	9.17 (7.55 - 11.11)	1.22 (0.91 - 1.63)
Coordination Disorder	levetiracetam	2nd Pregnancy Half (Any Use)	59 / 5261	91 / 8887	2.81 (1.85 - 4.26)	2.29 (1.72 - 3.06)	0.96 (0.67 - 1.38)
Coordination Disorder	levetiracetam	1st Pregnancy Half (Any Use)	54 / 4890	91 / 8887	2.27 (1.53 - 3.38)	2.26 (1.70 - 3.00)	0.87 (0.60 - 1.26)
Coordination Disorder	levetiracetam	Any Time in Pregnancy (Any Use)	66 / 6162	91 / 8887	2.59 (1.74 - 3.84)	2.24 (1.69 - 2.97)	0.91 (0.64 - 1.28)
Coordination Disorder	levetiracetam	2nd Pregnancy Half (Strict Monotherapy)	32 / 3360	91 / 8887	3.22 (1.78 - 5.79)	2.36 (1.74 - 3.20)	0.90 (0.58 - 1.41)
Coordination Disorder	levetiracetam	1st Pregnancy Half (Strict Monotherapy)	31 / 3045	91 / 8887	2.75 (1.62 - 4.67)	2.34 (1.73 - 3.17)	0.91 (0.58 - 1.42)
Coordination Disorder	levetiracetam	Any Time in Pregnancy (Strict Monotherap	36 / 3929	91 / 8887	2.98 (1.71 - 5.16)	2.31 (1.72 - 3.11)	0.86 (0.57 - 1.31)
Autism Spectrum Disorder	levetiracetam	2nd Pregnancy Half (Any Use)	40 / 5261	106 / 8887	3.10 (2.02 - 4.74)	4.22 (3.29 - 5.40)	0.67 (0.45 - 1.00)
Autism Spectrum Disorder	levetiracetam	1st Pregnancy Half (Any Use)	40 / 4890	106 / 8887	3.31 (2.19 - 4.99)	4.29 (3.35 - 5.49)	0.72 (0.49 - 1.08)
Autism Spectrum Disorder	levetiracetam	Any Time in Pregnancy (Any Use)	48 / 6162	106 / 8887	3.18 (2.18 - 4.63)	4.27 (3.35 - 5.44)	0.70 (0.48 - 1.02)
Autism Spectrum Disorder	levetiracetam	2nd Pregnancy Half (Strict Monotherapy)	21 / 3360	106 / 8887	3.33 (1.86 - 5.92)	4.22 (3.23 - 5.50)	0.58 (0.35 - 0.97)
Autism Spectrum Disorder	levetiracetam	1st Pregnancy Half (Strict Monotherapy)	19 / 3045	106 / 8887	2.79 (1.50 - 5.19)	4.22 (3.22 - 5.52)	0.57 (0.34 - 0.97)
Autism Spectrum Disorder	levetiracetam	Any Time in Pregnancy (Strict Monotherap	23 / 3929	106 / 8887	3.01 (1.73 - 5.18)	4.26 (3.28 - 5.52)	0.56 (0.35 - 0.92)
Learning Difficulty	levetiracetam	2nd Pregnancy Half (Any Use)	15 / 5261	43 / 8887	2.34 (1.21 - 4.49)	1.32 (0.76 - 2.28)	1.01 (0.52 - 1.94)
Learning Difficulty	levetiracetam	1st Pregnancy Half (Any Use)	14 / 4890	43 / 8887	2.22 (1.11 - 4.40)	1.36 (0.78 - 2.37)	1.00 (0.51 - 1.94)
Learning Difficulty	levetiracetam	Any Time in Pregnancy (Any Use)	16 / 6162	43 / 8887	2.14 (1.14 - 3.99)	1.32 (0.77 - 2.26)	0.92 (0.49 - 1.73)
Learning Difficulty	levetiracetam	2nd Pregnancy Half (Strict Monotherapy)	*** / 3360	43 / 8887	3.18 (1.46 - 6.87)	1.30 (0.71 - 2.36)	1.03 (0.44 - 2.39)
Learning Difficulty	levetiracetam	1st Pregnancy Half (Strict Monotherapy)	*** / 3045	43 / 8887	2.88 (1.26 - 6.50)	1.29 (0.70 - 2.39)	1.00 (0.42 - 2.43)
Learning Difficulty	levetiracetam	Any Time in Pregnancy (Strict Monotherap	*** / 3929	43 / 8887	2.92 (1.40 - 6.04)	1.29 (0.72 - 2.31)	1.01 (0.45 - 2.24)
Intellectual Disability	levetiracetam	2nd Pregnancy Half (Any Use)	*** / 5261	26 / 8887	0.73 (0.32 - 1.69)	0.81 (0.44 - 1.48)	1.14 (0.49 - 2.64)
Intellectual Disability	levetiracetam	1st Pregnancy Half (Any Use)	*** / 4890	26 / 8887	0.86 (0.37 - 1.99)	0.82 (0.44 - 1.52)	1.31 (0.58 - 2.98)
Intellectual Disability	levetiracetam	Any Time in Pregnancy (Any Use)	12 / 6162	26 / 8887	0.83 (0.40 - 1.73)	0.83 (0.46 - 1.50)	1.26 (0.59 - 2.69)
Intellectual Disability	levetiracetam	2nd Pregnancy Half (Strict Monotherapy)	*** / 3360	26 / 8887	0.46 (0.11 - 1.85)	0.82 (0.44 - 1.55)	0.60 (0.16 - 2.25)
Intellectual Disability	levetiracetam	1st Pregnancy Half (Strict Monotherapy)	*** / 3045	26 / 8887	0.31 (0.05 - 2.09)	0.78 (0.40 - 1.51)	0.71 (0.19 - 2.64)
Intellectual Disability	levetiracetam	Any Time in Pregnancy (Strict Monotherap	*** / 3929	26 / 8887	0.39 (0.10 - 1.57)	0.83 (0.45 - 1.54)	0.73 (0.23 - 2.28)

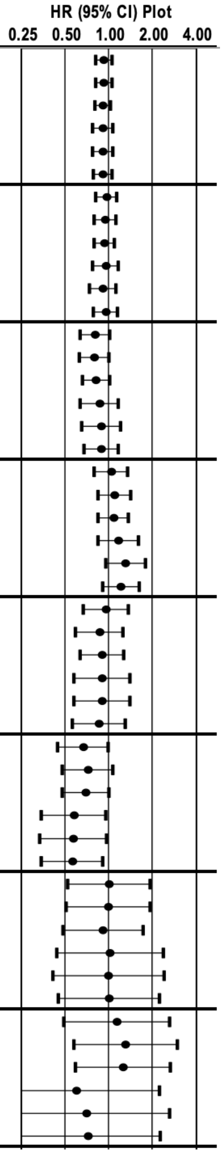

B) Lamotrigine

Outcome	Exposure	Analysis	N Cases / Total Among Exposed	N Cases / Total Among Unexposed	Cumulative Incidence in % (95% CI) at Age 8 Years Among Exposed	Cumulative Incidence in % (95% CI) at Age 8 Years Among Unexposed	HR (95% CI)	HR (95% CI) Plot 0.25 0.50 1.00 2.00 4.00
Any NDD	lamotrigine	2nd Pregnancy Half (Any Use)	404 / 4299	872 / 8887	34.49 (30.98 - 38.27)	32.67 (29.98 - 35.54)	1.05 (0.92 - 1.19)	
Any NDD	lamotrigine	1st Pregnancy Half (Any Use)	408 / 4440	872 / 8887	32.04 (28.73 - 35.62)	33.51 (30.79 - 36.40)	0.98 (0.86 - 1.11)	
Any NDD	lamotrigine	Any Time in Pregnancy (Any Use)	479 / 5040	872 / 8887	34.96 (31.70 - 38.46)	33.55 (30.94 - 36.33)	1.04 (0.92 - 1.17)	
Any NDD	lamotrigine	2nd Pregnancy Half (Strict Monotherapy)	248 / 2864	872 / 8887	32.58 (28.38 - 37.22)	31.02 (28.12 - 34.14)	1.01 (0.86 - 1.18)	
Any NDD	lamotrigine	1st Pregnancy Half (Strict Monotherapy)	251 / 2895	872 / 8887	30.53 (26.50 - 35.02)	32.17 (29.22 - 35.35)	0.94 (0.81 - 1.11)	
Any NDD	lamotrigine	Any Time in Pregnancy (Strict Monotherap	290 / 3273	872 / 8887	33.07 (29.12 - 37.39)	32.25 (29.45 - 35.23)	1.00 (0.87 - 1.16)	
Speech/Language Disorder	lamotrigine	2nd Pregnancy Half (Any Use)	246 / 4299	456 / 8887	16.20 (13.97 - 18.74)	14.78 (13.08 - 16.67)	1.11 (0.93 - 1.32)	
Speech/Language Disorder	lamotrigine	1st Pregnancy Half (Any Use)	252 / 4440	456 / 8887	16.06 (13.84 - 18.59)	14.99 (13.29 - 16.90)	1.09 (0.91 - 1.29)	
Speech/Language Disorder	lamotrigine	Any Time in Pregnancy (Any Use)	282 / 5040	456 / 8887	15.84 (13.78 - 18.17)	14.99 (13.34 - 16.82)	1.08 (0.92 - 1.27)	
Speech/Language Disorder	lamotrigine	2nd Pregnancy Half (Strict Monotherapy)	152 / 2864	456 / 8887	14.41 (11.86 - 17.47)	13.98 (12.20 - 15.99)	1.04 (0.84 - 1.28)	
Speech/Language Disorder	lamotrigine	1st Pregnancy Half (Strict Monotherapy)	157 / 2895	456 / 8887	14.29 (11.81 - 17.24)	14.31 (12.51 - 16.34)	1.04 (0.84 - 1.28)	
Speech/Language Disorder	lamotrigine	Any Time in Pregnancy (Strict Monotherap	175 / 3273	456 / 8887	14.26 (11.93 - 17.00)	14.36 (12.64 - 16.30)	1.04 (0.86 - 1.26)	
ADHD	lamotrigine	2nd Pregnancy Half (Any Use)	136 / 4299	366 / 8887	20.33 (17.16 - 24.01)	17.52 (15.19 - 20.17)	1.04 (0.84 - 1.29)	
ADHD	lamotrigine	1st Pregnancy Half (Any Use)	139 / 4440	366 / 8887	18.83 (15.84 - 22.30)	18.28 (15.89 - 20.97)	0.95 (0.76 - 1.17)	
ADHD	lamotrigine	Any Time in Pregnancy (Any Use)	171 / 5040	366 / 8887	21.23 (18.25 - 24.62)	18.28 (15.99 - 20.86)	1.05 (0.87 - 1.29)	
ADHD	lamotrigine	2nd Pregnancy Half (Strict Monotherapy)	84 / 2864	366 / 8887	19.82 (16.03 - 24.37)	16.58 (14.08 - 19.46)	1.09 (0.84 - 1.42)	
ADHD	lamotrigine	1st Pregnancy Half (Strict Monotherapy)	84 / 2895	366 / 8887	18.37 (14.74 - 22.78)	17.44 (14.87 - 20.40)	0.99 (0.76 - 1.28)	
ADHD	lamotrigine	Any Time in Pregnancy (Strict Monotherap	102 / 3273	366 / 8887	20.54 (16.94 - 24.79)	17.49 (15.06 - 20.26)	1.09 (0.86 - 1.38)	
Behavioral Disorder	lamotrigine	2nd Pregnancy Half (Any Use)	84 / 4299	239 / 8887	10.12 (7.97 - 12.81)	10.06 (8.38 - 12.04)	1.03 (0.79 - 1.35)	
Behavioral Disorder	lamotrigine	1st Pregnancy Half (Any Use)	85 / 4440	239 / 8887	9.21 (7.23 - 11.69)	10.50 (8.78 - 12.53)	0.93 (0.71 - 1.21)	
Behavioral Disorder	lamotrigine	Any Time in Pregnancy (Any Use)	111 / 5040	239 / 8887	10.93 (8.86 - 13.46)	10.53 (8.88 - 12.47)	1.08 (0.85 - 1.38)	
Behavioral Disorder	lamotrigine	2nd Pregnancy Half (Strict Monotherapy)	56 / 2864	239 / 8887	10.30 (7.76 - 13.61)	9.30 (7.54 - 11.45)	1.19 (0.87 - 1.62)	
Behavioral Disorder	lamotrigine	1st Pregnancy Half (Strict Monotherapy)	54 / 2895	239 / 8887	10.19 (7.62 - 13.58)	9.77 (7.95 - 11.98)	1.05 (0.76 - 1.45)	
Behavioral Disorder	lamotrigine	Any Time in Pregnancy (Strict Monotherap	70 / 3273	239 / 8887	11.51 (8.93 - 14.77)	9.97 (8.23 - 12.04)	1.19 (0.90 - 1.59)	
Coordination Disorder	lamotrigine	2nd Pregnancy Half (Any Use)	56 / 4299	91 / 8887	2.62 (1.71 - 4.01)	2.01 (1.48 - 2.72)	1.06 (0.72 - 1.55)	
Coordination Disorder	lamotrigine	1st Pregnancy Half (Any Use)	51 / 4440	91 / 8887	2.28 (1.45 - 3.59)	2.02 (1.50 - 2.72)	0.89 (0.60 - 1.32)	
Coordination Disorder	lamotrigine	Any Time in Pregnancy (Any Use)	60 / 5040	91 / 8887	2.45 (1.63 - 3.68)	2.00 (1.49 - 2.68)	0.95 (0.66 - 1.37)	
Coordination Disorder	lamotrigine	2nd Pregnancy Half (Strict Monotherapy)	27 / 2864	91 / 8887	1.33 (0.75 - 2.35)	2.00 (1.44 - 2.79)	0.74 (0.45 - 1.22)	
Coordination Disorder	lamotrigine	1st Pregnancy Half (Strict Monotherapy)	29 / 2895	91 / 8887	1.67 (0.96 - 2.89)	2.04 (1.48 - 2.83)	0.78 (0.48 - 1.26)	
Coordination Disorder	lamotrigine	Any Time in Pregnancy (Strict Monotherap	30 / 3273	91 / 8887	1.50 (0.87 - 2.57)	1.99 (1.45 - 2.73)	0.72 (0.45 - 1.16)	
Autism Spectrum Disorder	lamotrigine	2nd Pregnancy Half (Any Use)	55 / 4299	106 / 8887	4.84 (3.50 - 6.66)	4.29 (3.33 - 5.51)	1.10 (0.77 - 1.58)	
Autism Spectrum Disorder	lamotrigine	1st Pregnancy Half (Any Use)	57 / 4440	106 / 8887	4.66 (3.38 - 6.40)	4.34 (3.38 - 5.56)	1.06 (0.74 - 1.51)	
Autism Spectrum Disorder	lamotrigine	Any Time in Pregnancy (Any Use)	66 / 5040	106 / 8887	4.74 (3.52 - 6.36)	4.28 (3.35 - 5.46)	1.12 (0.79 - 1.57)	
Autism Spectrum Disorder	lamotrigine	2nd Pregnancy Half (Strict Monotherapy)	28 / 2864	106 / 8887	4.98 (3.29 - 7.49)	4.34 (3.30 - 5.70)	0.93 (0.59 - 1.48)	
Autism Spectrum Disorder	lamotrigine	1st Pregnancy Half (Strict Monotherapy)	29 / 2895	106 / 8887	4.35 (2.82 - 6.68)	4.40 (3.35 - 5.77)	0.89 (0.56 - 1.39)	
Autism Spectrum Disorder	lamotrigine	Any Time in Pregnancy (Strict Monotherap	34 / 3273	106 / 8887	4.67 (3.15 - 6.88)	4.33 (3.33 - 5.63)	0.96 (0.63 - 1.47)	
Learning Difficulty	lamotrigine	2nd Pregnancy Half (Any Use)	18 / 4299	43 / 8887	2.27 (1.20 - 4.29)	1.31 (0.75 - 2.29)	1.10 (0.59 - 2.05)	
Learning Difficulty	lamotrigine	1st Pregnancy Half (Any Use)	21 / 4440	43 / 8887	2.55 (1.42 - 4.56)	1.40 (0.81 - 2.41)	1.22 (0.68 - 2.21)	
Learning Difficulty	lamotrigine	Any Time in Pregnancy (Any Use)	22 / 5040	43 / 8887	2.31 (1.30 - 4.08)	1.32 (0.77 - 2.26)	1.15 (0.65 - 2.05)	
Learning Difficulty	lamotrigine	2nd Pregnancy Half (Strict Monotherapy)	*** / 2864	43 / 8887	2.39 (1.12 - 5.03)	1.22 (0.65 - 2.30)	0.91 (0.42 - 1.97)	
Learning Difficulty	lamotrigine	1st Pregnancy Half (Strict Monotherapy)	12 / 2895	43 / 8887	2.71 (1.35 - 5.40)	1.34 (0.72 - 2.48)	1.10 (0.53 - 2.28)	
Learning Difficulty	lamotrigine	Any Time in Pregnancy (Strict Monotherap	12 / 3273	43 / 8887	2.37 (1.18 - 4.72)	1.26 (0.70 - 2.29)	0.96 (0.47 - 1.96)	
Intellectual Disability	lamotrigine	2nd Pregnancy Half (Any Use)	16 / 4299	26 / 8887	2.20 (1.24 - 3.88)	0.62 (0.31 - 1.22)	2.38 (1.20 - 4.73)	
Intellectual Disability	lamotrigine	1st Pregnancy Half (Any Use)	12 / 4440	26 / 8887	1.43 (0.71 - 2.88)	0.69 (0.36 - 1.32)	1.39 (0.66 - 2.95)	
Intellectual Disability	lamotrigine	Any Time in Pregnancy (Any Use)	17 / 5040	26 / 8887	1.89 (1.08 - 3.32)	0.64 (0.33 - 1.23)	1.98 (1.02 - 3.86)	
Intellectual Disability	lamotrigine	2nd Pregnancy Half (Strict Monotherapy)	12 / 2864	26 / 8887	2.40 (1.23 - 4.67)	0.54 (0.25 - 1.17)	2.96 (1.37 - 6.38)	
Intellectual Disability	lamotrigine	1st Pregnancy Half (Strict Monotherapy)	*** / 2895	26 / 8887	1.22 (0.47 - 3.14)	0.61 (0.29 - 1.29)	1.32 (0.53 - 3.30)	
Intellectual Disability	lamotrigine	Any Time in Pregnancy (Strict Monotherap	12 / 3273	26 / 8887	2.06 (1.05 - 4.05)	0.56 (0.27 - 1.16)	2.46 (1.16 - 5.24)	

C) Carbamazepine

Outcome	Exposure	Analysis	N Cases / Total Among Exposed	N Cases / Total Among Unexposed	Cumulative Incidence in % (95% CI) at Age 8 Years Among Exposed	Cumulative Incidence in % (95% CI) at Age 8 Years Among Unexposed	HR (95% CI)
Any NDD	carbamazepine	2nd Pregnancy Half (Any Use)	275 / 1924	872 / 8887	37.00 (32.88 - 41.45)	31.61 (28.81 - 34.61)	1.16 (1.00 - 1.35)
Any NDD	carbamazepine	1st Pregnancy Half (Any Use)	284 / 2074	872 / 8887	34.71 (30.85 - 38.89)	32.90 (30.12 - 35.86)	1.03 (0.89 - 1.20)
Any NDD	carbamazepine	Any Time in Pregnancy (Any Use)	326 / 2374	872 / 8887	35.29 (31.64 - 39.22)	32.71 (30.00 - 35.60)	1.06 (0.92 - 1.22)
Any NDD	carbamazepine	2nd Pregnancy Half (Strict Monotherapy)	184 / 1333	872 / 8887	37.48 (32.58 - 42.86)	30.69 (27.78 - 33.84)	1.21 (1.02 - 1.45)
Any NDD	carbamazepine	1st Pregnancy Half (Strict Monotherapy)	179 / 1354	872 / 8887	34.25 (29.64 - 39.35)	32.11 (29.22 - 35.22)	1.04 (0.87 - 1.24)
Any NDD	carbamazepine	Any Time in Pregnancy (Strict Monotherap	205 / 1548	872 / 8887	35.15 (30.76 - 39.97)	31.61 (28.80 - 34.62)	1.08 (0.92 - 1.28)
Speech/Language Disorder	carbamazepine	2nd Pregnancy Half (Any Use)	124 / 1924	456 / 8887	13.22 (10.94 - 15.94)	13.24 (11.50 - 15.22)	1.07 (0.86 - 1.34)
Speech/Language Disorder	carbamazepine	1st Pregnancy Half (Any Use)	128 / 2074	456 / 8887	12.85 (10.65 - 15.46)	13.64 (11.92 - 15.59)	0.98 (0.79 - 1.22)
Speech/Language Disorder	carbamazepine	Any Time in Pregnancy (Any Use)	147 / 2374	456 / 8887	13.16 (11.05 - 15.63)	13.68 (11.99 - 15.58)	1.00 (0.81 - 1.22)
Speech/Language Disorder	carbamazepine	2nd Pregnancy Half (Strict Monotherapy)	83 / 1333	456 / 8887	13.39 (10.67 - 16.74)	12.75 (10.95 - 14.81)	1.11 (0.86 - 1.44)
Speech/Language Disorder	carbamazepine	1st Pregnancy Half (Strict Monotherapy)	82 / 1354	456 / 8887	12.58 (10.00 - 15.76)	13.07 (11.30 - 15.09)	1.04 (0.80 - 1.34)
Speech/Language Disorder	carbamazepine	Any Time in Pregnancy (Strict Monotherap	94 / 1548	456 / 8887	13.14 (10.62 - 16.21)	13.10 (11.36 - 15.08)	1.04 (0.82 - 1.33)
ADHD	carbamazepine	2nd Pregnancy Half (Any Use)	144 / 1924	366 / 8887	23.06 (19.35 - 27.36)	18.56 (16.12 - 21.33)	1.23 (0.99 - 1.52)
ADHD	carbamazepine	1st Pregnancy Half (Any Use)	147 / 2074	366 / 8887	21.73 (18.25 - 25.76)	19.29 (16.84 - 22.05)	1.08 (0.87 - 1.33)
ADHD	carbamazepine	Any Time in Pregnancy (Any Use)	172 / 2374	366 / 8887	22.22 (18.92 - 26.01)	19.22 (16.83 - 21.90)	1.13 (0.93 - 1.38)
ADHD	carbamazepine	2nd Pregnancy Half (Strict Monotherapy)	95 / 1333	366 / 8887	23.70 (19.29 - 28.91)	18.13 (15.60 - 21.02)	1.26 (0.98 - 1.62)
ADHD	carbamazepine	1st Pregnancy Half (Strict Monotherapy)	93 / 1354	366 / 8887	21.50 (17.41 - 26.40)	19.06 (16.53 - 21.93)	1.08 (0.84 - 1.39)
ADHD	carbamazepine	Any Time in Pregnancy (Strict Monotherap	107 / 1548	366 / 8887	21.93 (18.03 - 26.53)	18.70 (16.25 - 21.48)	1.13 (0.89 - 1.43)
Behavioral Disorder	carbamazepine	2nd Pregnancy Half (Any Use)	93 / 1924	239 / 8887	13.05 (10.40 - 16.33)	10.39 (8.63 - 12.48)	1.35 (1.04 - 1.77)
Behavioral Disorder	carbamazepine	1st Pregnancy Half (Any Use)	98 / 2074	239 / 8887	12.25 (9.79 - 15.26)	10.86 (9.09 - 12.94)	1.22 (0.94 - 1.58)
Behavioral Disorder	carbamazepine	Any Time in Pregnancy (Any Use)	114 / 2374	239 / 8887	12.95 (10.55 - 15.86)	10.80 (9.07 - 12.82)	1.27 (0.99 - 1.62)
Behavioral Disorder	carbamazepine	2nd Pregnancy Half (Strict Monotherapy)	66 / 1333	239 / 8887	13.65 (10.46 - 17.72)	10.27 (8.44 - 12.48)	1.46 (1.08 - 1.98)
Behavioral Disorder	carbamazepine	1st Pregnancy Half (Strict Monotherapy)	68 / 1354	239 / 8887	13.32 (10.24 - 17.23)	10.87 (9.02 - 13.07)	1.32 (0.99 - 1.78)
Behavioral Disorder	carbamazepine	Any Time in Pregnancy (Strict Monotherap	78 / 1548	239 / 8887	13.72 (10.75 - 17.44)	10.73 (8.93 - 12.86)	1.38 (1.04 - 1.82)
Coordination Disorder	carbamazepine	2nd Pregnancy Half (Any Use)	22 / 1924	91 / 8887	2.80 (1.71 - 4.56)	1.50 (1.01 - 2.22)	1.20 (0.72 - 2.00)
Coordination Disorder	carbamazepine	1st Pregnancy Half (Any Use)	24 / 2074	91 / 8887	2.52 (1.56 - 4.08)	1.63 (1.13 - 2.34)	1.06 (0.65 - 1.75)
Coordination Disorder	carbamazepine	Any Time in Pregnancy (Any Use)	27 / 2374	91 / 8887	2.74 (1.74 - 4.30)	1.56 (1.08 - 2.26)	1.14 (0.70 - 1.83)
Coordination Disorder	carbamazepine	2nd Pregnancy Half (Strict Monotherapy)	15 / 1333	91 / 8887	3.11 (1.73 - 5.56)	1.38 (0.90 - 2.12)	1.30 (0.72 - 2.37)
Coordination Disorder	carbamazepine	1st Pregnancy Half (Strict Monotherapy)	13 / 1354	91 / 8887	2.34 (1.24 - 4.37)	1.50 (1.01 - 2.23)	0.98 (0.52 - 1.83)
Coordination Disorder	carbamazepine	Any Time in Pregnancy (Strict Monotherap	16 / 1548	91 / 8887	2.78 (1.59 - 4.86)	1.41 (0.94 - 2.12)	1.17 (0.66 - 2.09)
Autism Spectrum Disorder	carbamazepine	2nd Pregnancy Half (Any Use)	30 / 1924	106 / 8887	3.80 (2.48 - 5.79)	3.79 (2.87 - 5.01)	0.94 (0.60 - 1.48)
Autism Spectrum Disorder	carbamazepine	1st Pregnancy Half (Any Use)	33 / 2074	106 / 8887	3.90 (2.61 - 5.81)	3.76 (2.86 - 4.93)	0.93 (0.61 - 1.43)
Autism Spectrum Disorder	carbamazepine	Any Time in Pregnancy (Any Use)	35 / 2374	106 / 8887	3.61 (2.42 - 5.34)	3.78 (2.89 - 4.94)	0.86 (0.57 - 1.32)
Autism Spectrum Disorder	carbamazepine	2nd Pregnancy Half (Strict Monotherapy)	21 / 1333	106 / 8887	3.40 (2.08 - 5.53)	3.87 (2.88 - 5.18)	1.00 (0.60 - 1.67)
Autism Spectrum Disorder	carbamazepine	1st Pregnancy Half (Strict Monotherapy)	21 / 1354	106 / 8887	3.33 (2.05 - 5.38)	3.87 (2.91 - 5.14)	0.94 (0.57 - 1.56)
Autism Spectrum Disorder	carbamazepine	Any Time in Pregnancy (Strict Monotherap	22 / 1548	106 / 8887	2.98 (1.85 - 4.79)	3.86 (2.91 - 5.10)	0.88 (0.54 - 1.45)
Learning Difficulty	carbamazepine	2nd Pregnancy Half (Any Use)	19 / 1924	43 / 8887	1.78 (0.89 - 3.55)	1.32 (0.76 - 2.28)	1.16 (0.64 - 2.10)
Learning Difficulty	carbamazepine	1st Pregnancy Half (Any Use)	20 / 2074	43 / 8887	1.59 (0.75 - 3.36)	1.37 (0.81 - 2.33)	1.02 (0.58 - 1.82)
Learning Difficulty	carbamazepine	Any Time in Pregnancy (Any Use)	24 / 2374	43 / 8887	1.71 (0.89 - 3.28)	1.35 (0.80 - 2.27)	1.16 (0.67 - 2.00)
Learning Difficulty	carbamazepine	2nd Pregnancy Half (Strict Monotherapy)	13 / 1333	43 / 8887	2.45 (1.19 - 5.01)	1.31 (0.74 - 2.33)	1.15 (0.58 - 2.29)
Learning Difficulty	carbamazepine	1st Pregnancy Half (Strict Monotherapy)	11 / 1354	43 / 8887	1.90 (0.82 - 4.40)	1.38 (0.79 - 2.41)	0.87 (0.43 - 1.78)
Learning Difficulty	carbamazepine	Any Time in Pregnancy (Strict Monotherap	13 / 1548	43 / 8887	2.06 (1.00 - 4.21)	1.35 (0.78 - 2.33)	0.96 (0.49 - 1.89)
Intellectual Disability	carbamazepine	2nd Pregnancy Half (Any Use)	*** / 1924	26 / 8887	0.98 (0.39 - 2.46)	0.79 (0.41 - 1.52)	0.84 (0.36 - 1.95)
Intellectual Disability	carbamazepine	1st Pregnancy Half (Any Use)	*** / 2074	26 / 8887	0.93 (0.37 - 2.30)	0.89 (0.48 - 1.64)	0.75 (0.33 - 1.72)
Intellectual Disability	carbamazepine	Any Time in Pregnancy (Any Use)	*** / 2374	26 / 8887	0.76 (0.29 - 1.94)	0.82 (0.44 - 1.53)	0.65 (0.28 - 1.50)
Intellectual Disability	carbamazepine	2nd Pregnancy Half (Strict Monotherapy)	*** / 1333	26 / 8887	0.86 (0.29 - 2.51)	0.79 (0.40 - 1.54)	0.95 (0.36 - 2.53)
Intellectual Disability	carbamazepine	1st Pregnancy Half (Strict Monotherapy)	*** / 1354	26 / 8887	0.84 (0.29 - 2.41)	0.88 (0.46 - 1.68)	0.88 (0.34 - 2.29)
Intellectual Disability	carbamazepine	Any Time in Pregnancy (Strict Monotherap	*** / 1548	26 / 8887	0.70 (0.24 - 2.08)	0.80 (0.42 - 1.53)	0.78 (0.30 - 2.04)

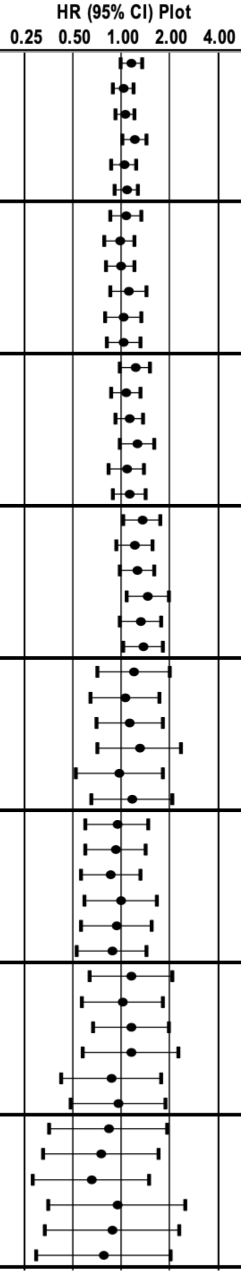

D) Phenytoin

Outcome	Exposure	Analysis	N Cases / Total		Cumulative Incidence in % (95% CI) at Age 8 Years		HR (95% CI)
			Among Exposed	Among Unexposed	Among Exposed	Among Unexposed	
Any NDD	phenytoin	2nd Pregnancy Half (Any Use)	237 / 1595	872 / 8887	31.33 (27.49 - 35.56)	34.35 (31.50 - 37.38)	0.91 (0.78 - 1.07)
Any NDD	phenytoin	1st Pregnancy Half (Any Use)	253 / 1867	872 / 8887	30.79 (27.11 - 34.85)	35.19 (32.40 - 38.15)	0.85 (0.73 - 0.98)
Any NDD	phenytoin	Any Time in Pregnancy (Any Use)	325 / 2295	872 / 8887	31.96 (28.57 - 35.64)	35.13 (32.39 - 38.03)	0.88 (0.77 - 1.02)
Any NDD	phenytoin	2nd Pregnancy Half (Strict Monotherapy)	160 / 1060	872 / 8887	31.21 (26.73 - 36.23)	33.58 (30.67 - 36.68)	0.91 (0.76 - 1.10)
Any NDD	phenytoin	1st Pregnancy Half (Strict Monotherapy)	150 / 1102	872 / 8887	29.73 (25.28 - 34.76)	34.58 (31.72 - 37.62)	0.81 (0.68 - 0.98)
Any NDD	phenytoin	Any Time in Pregnancy (Strict Monotherap	198 / 1390	872 / 8887	31.29 (27.21 - 35.80)	34.34 (31.54 - 37.32)	0.86 (0.72 - 1.01)
Speech/Language Disorder	phenytoin	2nd Pregnancy Half (Any Use)	98 / 1595	456 / 8887	11.93 (9.68 - 14.67)	13.68 (11.92 - 15.69)	0.85 (0.66 - 1.08)
Speech/Language Disorder	phenytoin	1st Pregnancy Half (Any Use)	100 / 1867	456 / 8887	11.18 (9.07 - 13.74)	14.52 (12.75 - 16.52)	0.74 (0.58 - 0.93)
Speech/Language Disorder	phenytoin	Any Time in Pregnancy (Any Use)	130 / 2295	456 / 8887	11.98 (9.99 - 14.33)	14.25 (12.53 - 16.19)	0.80 (0.64 - 0.99)
Speech/Language Disorder	phenytoin	2nd Pregnancy Half (Strict Monotherapy)	62 / 1060	456 / 8887	11.82 (9.16 - 15.20)	13.19 (11.40 - 15.23)	0.83 (0.62 - 1.11)
Speech/Language Disorder	phenytoin	1st Pregnancy Half (Strict Monotherapy)	54 / 1102	456 / 8887	10.44 (7.91 - 13.73)	13.83 (12.04 - 15.86)	0.68 (0.50 - 0.91)
Speech/Language Disorder	phenytoin	Any Time in Pregnancy (Strict Monotherap	75 / 1390	456 / 8887	11.55 (9.14 - 14.54)	13.54 (11.80 - 15.51)	0.76 (0.59 - 0.99)
ADHD	phenytoin	2nd Pregnancy Half (Any Use)	144 / 1595	366 / 8887	20.90 (17.41 - 24.98)	21.14 (18.58 - 23.99)	1.01 (0.82 - 1.24)
ADHD	phenytoin	1st Pregnancy Half (Any Use)	148 / 1867	366 / 8887	20.46 (17.11 - 24.37)	21.20 (18.69 - 24.01)	0.95 (0.77 - 1.17)
ADHD	phenytoin	Any Time in Pregnancy (Any Use)	193 / 2295	366 / 8887	21.01 (17.94 - 24.52)	21.31 (18.85 - 24.05)	0.99 (0.82 - 1.20)
ADHD	phenytoin	2nd Pregnancy Half (Strict Monotherapy)	98 / 1060	366 / 8887	19.99 (16.04 - 24.76)	20.84 (18.25 - 23.74)	1.00 (0.79 - 1.28)
ADHD	phenytoin	1st Pregnancy Half (Strict Monotherapy)	88 / 1102	366 / 8887	18.69 (14.85 - 23.37)	21.24 (18.67 - 24.11)	0.91 (0.71 - 1.17)
ADHD	phenytoin	Any Time in Pregnancy (Strict Monotherap	121 / 1390	366 / 8887	19.99 (16.42 - 24.22)	21.24 (18.73 - 24.04)	0.97 (0.78 - 1.21)
Behavioral Disorder	phenytoin	2nd Pregnancy Half (Any Use)	99 / 1595	239 / 8887	12.43 (9.83 - 15.67)	11.40 (9.58 - 13.54)	1.19 (0.92 - 1.54)
Behavioral Disorder	phenytoin	1st Pregnancy Half (Any Use)	98 / 1867	239 / 8887	12.24 (9.68 - 15.42)	11.50 (9.71 - 13.59)	1.04 (0.81 - 1.35)
Behavioral Disorder	phenytoin	Any Time in Pregnancy (Any Use)	134 / 2295	239 / 8887	13.07 (10.69 - 15.94)	11.66 (9.89 - 13.71)	1.14 (0.90 - 1.44)
Behavioral Disorder	phenytoin	2nd Pregnancy Half (Strict Monotherapy)	68 / 1060	239 / 8887	12.13 (9.18 - 15.94)	10.96 (9.14 - 13.12)	1.24 (0.92 - 1.66)
Behavioral Disorder	phenytoin	1st Pregnancy Half (Strict Monotherapy)	64 / 1102	239 / 8887	13.49 (10.24 - 17.66)	11.14 (9.33 - 13.26)	1.15 (0.85 - 1.54)
Behavioral Disorder	phenytoin	Any Time in Pregnancy (Strict Monotherap	88 / 1390	239 / 8887	13.85 (10.92 - 17.48)	11.30 (9.52 - 13.39)	1.23 (0.94 - 1.60)
Coordination Disorder	phenytoin	2nd Pregnancy Half (Any Use)	20 / 1595	91 / 8887	2.79 (1.67 - 4.64)	1.64 (1.09 - 2.44)	1.08 (0.63 - 1.86)
Coordination Disorder	phenytoin	1st Pregnancy Half (Any Use)	21 / 1867	91 / 8887	2.49 (1.52 - 4.08)	1.70 (1.18 - 2.46)	1.00 (0.59 - 1.69)
Coordination Disorder	phenytoin	Any Time in Pregnancy (Any Use)	27 / 2295	91 / 8887	2.54 (1.62 - 3.96)	1.69 (1.16 - 2.45)	1.07 (0.66 - 1.73)
Coordination Disorder	phenytoin	2nd Pregnancy Half (Strict Monotherapy)	12 / 1060	91 / 8887	2.43 (1.25 - 4.69)	1.58 (1.03 - 2.42)	0.97 (0.50 - 1.88)
Coordination Disorder	phenytoin	1st Pregnancy Half (Strict Monotherapy)	12 / 1102	91 / 8887	2.26 (1.20 - 4.25)	1.61 (1.08 - 2.41)	0.96 (0.50 - 1.85)
Coordination Disorder	phenytoin	Any Time in Pregnancy (Strict Monotherap	16 / 1390	91 / 8887	2.37 (1.34 - 4.18)	1.61 (1.07 - 2.41)	1.02 (0.57 - 1.84)
Autism Spectrum Disorder	phenytoin	2nd Pregnancy Half (Any Use)	21 / 1595	106 / 8887	2.79 (1.75 - 4.44)	3.54 (2.64 - 4.74)	0.78 (0.47 - 1.31)
Autism Spectrum Disorder	phenytoin	1st Pregnancy Half (Any Use)	26 / 1867	106 / 8887	2.93 (1.91 - 4.47)	3.79 (2.87 - 4.99)	0.84 (0.53 - 1.35)
Autism Spectrum Disorder	phenytoin	Any Time in Pregnancy (Any Use)	30 / 2295	106 / 8887	2.81 (1.89 - 4.18)	3.71 (2.81 - 4.88)	0.80 (0.51 - 1.24)
Autism Spectrum Disorder	phenytoin	2nd Pregnancy Half (Strict Monotherapy)	11 / 1060	106 / 8887	2.29 (1.20 - 4.34)	3.56 (2.64 - 4.79)	0.59 (0.31 - 1.13)
Autism Spectrum Disorder	phenytoin	1st Pregnancy Half (Strict Monotherapy)	*** / 1102	106 / 8887	1.95 (0.99 - 3.82)	3.73 (2.80 - 4.96)	0.51 (0.26 - 1.00)
Autism Spectrum Disorder	phenytoin	Any Time in Pregnancy (Strict Monotherap	13 / 1390	106 / 8887	2.14 (1.18 - 3.87)	3.60 (2.70 - 4.78)	0.54 (0.29 - 0.98)
Learning Difficulty	phenytoin	2nd Pregnancy Half (Any Use)	15 / 1595	43 / 8887	1.84 (0.93 - 3.64)	1.58 (0.97 - 2.58)	0.73 (0.39 - 1.37)
Learning Difficulty	phenytoin	1st Pregnancy Half (Any Use)	15 / 1867	43 / 8887	1.48 (0.72 - 3.00)	1.65 (1.02 - 2.66)	0.76 (0.41 - 1.43)
Learning Difficulty	phenytoin	Any Time in Pregnancy (Any Use)	18 / 2295	43 / 8887	1.58 (0.83 - 3.00)	1.64 (1.03 - 2.62)	0.69 (0.38 - 1.23)
Learning Difficulty	phenytoin	2nd Pregnancy Half (Strict Monotherapy)	*** / 1060	43 / 8887	2.00 (0.91 - 4.37)	1.66 (1.01 - 2.71)	0.66 (0.31 - 1.41)
Learning Difficulty	phenytoin	1st Pregnancy Half (Strict Monotherapy)	*** / 1102	43 / 8887	1.66 (0.72 - 3.80)	1.65 (1.01 - 2.70)	0.83 (0.40 - 1.71)
Learning Difficulty	phenytoin	Any Time in Pregnancy (Strict Monotherap	12 / 1390	43 / 8887	1.90 (0.93 - 3.89)	1.78 (1.12 - 2.82)	0.72 (0.37 - 1.42)
Intellectual Disability	phenytoin	2nd Pregnancy Half (Any Use)	11 / 1595	26 / 8887	1.57 (0.77 - 3.16)	1.06 (0.60 - 1.90)	1.05 (0.49 - 2.24)
Intellectual Disability	phenytoin	1st Pregnancy Half (Any Use)	*** / 1867	26 / 8887	1.09 (0.46 - 2.57)	1.11 (0.64 - 1.94)	0.62 (0.25 - 1.50)
Intellectual Disability	phenytoin	Any Time in Pregnancy (Any Use)	11 / 2295	26 / 8887	1.16 (0.57 - 2.37)	1.08 (0.62 - 1.89)	0.77 (0.36 - 1.63)
Intellectual Disability	phenytoin	2nd Pregnancy Half (Strict Monotherapy)	*** / 1060	26 / 8887	1.71 (0.76 - 3.84)	1.01 (0.55 - 1.85)	1.26 (0.54 - 2.90)
Intellectual Disability	phenytoin	1st Pregnancy Half (Strict Monotherapy)	*** / 1102	26 / 8887	1.31 (0.49 - 3.47)	1.10 (0.62 - 1.96)	0.78 (0.29 - 2.07)
Intellectual Disability	phenytoin	Any Time in Pregnancy (Strict Monotherap	*** / 1390	26 / 8887	1.32 (0.58 - 3.02)	1.04 (0.58 - 1.86)	0.95 (0.42 - 2.17)

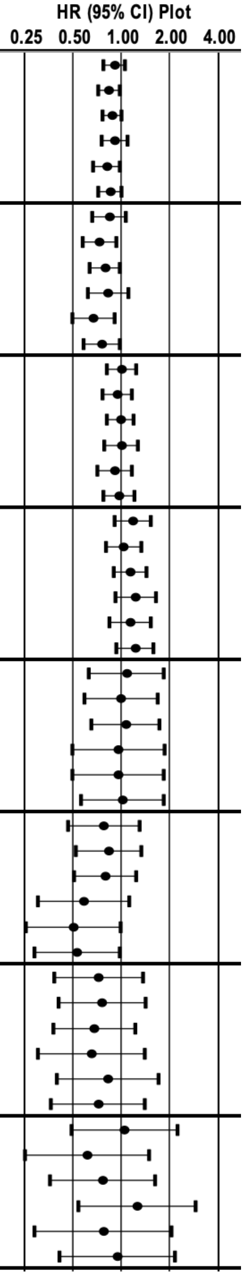

E) Topiramate

Outcome	Exposure	Analysis	N Cases / Total		Cumulative Incidence in % (95% CI) at Age 8 Years		HR (95% CI)
			Among Exposed	Among Unexposed	Among Exposed	Among Unexposed	
Any NDD	topiramate	2nd Pregnancy Half (Any Use)	145 / 1036	872 / 8887	37.97 (32.21 - 44.39)	36.29 (33.30 - 39.45)	1.13 (0.93 - 1.36)
Any NDD	topiramate	1st Pregnancy Half (Any Use)	211 / 1689	872 / 8887	38.10 (33.08 - 43.59)	37.51 (34.55 - 40.64)	1.02 (0.86 - 1.20)
Any NDD	topiramate	Any Time in Pregnancy (Any Use)	239 / 1857	872 / 8887	39.12 (34.30 - 44.36)	37.54 (34.65 - 40.60)	1.05 (0.90 - 1.23)
Any NDD	topiramate	2nd Pregnancy Half (Strict Monotherapy)	73 / 565	872 / 8887	32.73 (25.88 - 40.84)	36.11 (32.97 - 39.45)	1.05 (0.82 - 1.34)
Any NDD	topiramate	1st Pregnancy Half (Strict Monotherapy)	104 / 865	872 / 8887	34.73 (28.51 - 41.86)	37.74 (34.54 - 41.12)	0.94 (0.76 - 1.16)
Any NDD	topiramate	Any Time in Pregnancy (Strict Monotherap	117 / 961	872 / 8887	35.27 (29.32 - 42.01)	37.93 (34.81 - 41.23)	0.98 (0.80 - 1.20)
Speech/Language Disorder	topiramate	2nd Pregnancy Half (Any Use)	75 / 1036	456 / 8887	17.13 (13.43 - 21.71)	15.88 (13.99 - 18.00)	1.10 (0.85 - 1.43)
Speech/Language Disorder	topiramate	1st Pregnancy Half (Any Use)	98 / 1689	456 / 8887	16.14 (12.99 - 19.96)	16.56 (14.66 - 18.68)	0.91 (0.72 - 1.15)
Speech/Language Disorder	topiramate	Any Time in Pregnancy (Any Use)	114 / 1857	456 / 8887	16.69 (13.59 - 20.41)	16.62 (14.74 - 18.70)	0.95 (0.76 - 1.19)
Speech/Language Disorder	topiramate	2nd Pregnancy Half (Strict Monotherapy)	38 / 565	456 / 8887	15.63 (11.28 - 21.44)	15.93 (13.95 - 18.17)	1.04 (0.73 - 1.47)
Speech/Language Disorder	topiramate	1st Pregnancy Half (Strict Monotherapy)	46 / 865	456 / 8887	14.19 (10.42 - 19.16)	16.68 (14.63 - 18.98)	0.81 (0.58 - 1.11)
Speech/Language Disorder	topiramate	Any Time in Pregnancy (Strict Monotherap	56 / 961	456 / 8887	15.27 (11.55 - 20.05)	16.84 (14.83 - 19.10)	0.89 (0.66 - 1.19)
ADHD	topiramate	2nd Pregnancy Half (Any Use)	63 / 1036	366 / 8887	21.90 (16.82 - 28.24)	20.36 (17.69 - 23.36)	1.13 (0.85 - 1.50)
ADHD	topiramate	1st Pregnancy Half (Any Use)	102 / 1689	366 / 8887	24.38 (19.72 - 29.92)	20.68 (18.01 - 23.68)	1.17 (0.92 - 1.50)
ADHD	topiramate	Any Time in Pregnancy (Any Use)	117 / 1857	366 / 8887	25.66 (21.12 - 30.97)	20.95 (18.32 - 23.88)	1.23 (0.98 - 1.55)
ADHD	topiramate	2nd Pregnancy Half (Strict Monotherapy)	31 / 565	366 / 8887	18.03 (12.26 - 26.08)	19.89 (17.14 - 23.02)	0.98 (0.67 - 1.44)
ADHD	topiramate	1st Pregnancy Half (Strict Monotherapy)	51 / 865	366 / 8887	22.32 (16.67 - 29.52)	20.46 (17.62 - 23.69)	1.09 (0.79 - 1.50)
ADHD	topiramate	Any Time in Pregnancy (Strict Monotherap	56 / 961	366 / 8887	22.12 (16.73 - 28.92)	21.07 (18.27 - 24.24)	1.09 (0.81 - 1.48)
Behavioral Disorder	topiramate	2nd Pregnancy Half (Any Use)	35 / 1036	239 / 8887	9.54 (6.54 - 13.82)	11.40 (9.48 - 13.68)	1.03 (0.70 - 1.49)
Behavioral Disorder	topiramate	1st Pregnancy Half (Any Use)	62 / 1689	239 / 8887	12.86 (9.58 - 17.16)	12.06 (10.10 - 14.38)	1.15 (0.85 - 1.57)
Behavioral Disorder	topiramate	Any Time in Pregnancy (Any Use)	71 / 1857	239 / 8887	13.35 (10.16 - 17.44)	12.02 (10.11 - 14.26)	1.20 (0.89 - 1.60)
Behavioral Disorder	topiramate	2nd Pregnancy Half (Strict Monotherapy)	19 / 565	239 / 8887	8.96 (5.38 - 14.73)	10.87 (8.89 - 13.26)	1.08 (0.66 - 1.76)
Behavioral Disorder	topiramate	1st Pregnancy Half (Strict Monotherapy)	31 / 865	239 / 8887	12.27 (8.18 - 18.19)	12.29 (10.14 - 14.86)	1.08 (0.72 - 1.61)
Behavioral Disorder	topiramate	Any Time in Pregnancy (Strict Monotherap	35 / 961	239 / 8887	12.82 (8.79 - 18.49)	12.12 (10.05 - 14.58)	1.14 (0.78 - 1.67)
Coordination Disorder	topiramate	2nd Pregnancy Half (Any Use)	*** / 1036	91 / 8887	2.26 (0.94 - 5.40)	1.90 (1.36 - 2.65)	0.63 (0.30 - 1.34)
Coordination Disorder	topiramate	1st Pregnancy Half (Any Use)	13 / 1689	91 / 8887	2.15 (1.08 - 4.27)	2.20 (1.63 - 2.97)	0.60 (0.32 - 1.13)
Coordination Disorder	topiramate	Any Time in Pregnancy (Any Use)	14 / 1857	91 / 8887	2.40 (1.20 - 4.77)	2.20 (1.62 - 2.98)	0.59 (0.32 - 1.07)
Coordination Disorder	topiramate	2nd Pregnancy Half (Strict Monotherapy)	*** / 565	91 / 8887	1.45 (0.51 - 4.12)	1.90 (1.34 - 2.70)	0.65 (0.23 - 1.81)
Coordination Disorder	topiramate	1st Pregnancy Half (Strict Monotherapy)	*** / 865	91 / 8887	2.20 (0.89 - 5.34)	2.27 (1.65 - 3.13)	0.66 (0.29 - 1.49)
Coordination Disorder	topiramate	Any Time in Pregnancy (Strict Monotherap	*** / 961	91 / 8887	1.96 (0.78 - 4.88)	2.26 (1.64 - 3.10)	0.59 (0.26 - 1.33)
Autism Spectrum Disorder	topiramate	2nd Pregnancy Half (Any Use)	18 / 1036	106 / 8887	5.65 (3.32 - 9.52)	4.57 (3.51 - 5.94)	1.02 (0.60 - 1.74)
Autism Spectrum Disorder	topiramate	1st Pregnancy Half (Any Use)	26 / 1689	106 / 8887	4.84 (2.96 - 7.87)	4.70 (3.62 - 6.08)	0.89 (0.56 - 1.43)
Autism Spectrum Disorder	topiramate	Any Time in Pregnancy (Any Use)	30 / 1857	106 / 8887	4.97 (3.16 - 7.79)	4.77 (3.69 - 6.14)	0.95 (0.61 - 1.49)
Autism Spectrum Disorder	topiramate	2nd Pregnancy Half (Strict Monotherapy)	*** / 565	106 / 8887	5.72 (2.81 - 11.47)	4.67 (3.53 - 6.16)	0.99 (0.50 - 1.94)
Autism Spectrum Disorder	topiramate	1st Pregnancy Half (Strict Monotherapy)	13 / 865	106 / 8887	4.39 (2.19 - 8.68)	4.90 (3.72 - 6.44)	0.75 (0.40 - 1.41)
Autism Spectrum Disorder	topiramate	Any Time in Pregnancy (Strict Monotherap	15 / 961	106 / 8887	4.77 (2.54 - 8.87)	4.99 (3.81 - 6.52)	0.83 (0.46 - 1.48)
Learning Difficulty	topiramate	2nd Pregnancy Half (Any Use)	*** / 1036	43 / 8887	2.60 (1.05 - 6.34)	1.29 (0.71 - 2.33)	1.23 (0.54 - 2.79)
Learning Difficulty	topiramate	1st Pregnancy Half (Any Use)	*** / 1689	43 / 8887	1.02 (0.36 - 2.88)	1.16 (0.62 - 2.18)	1.01 (0.46 - 2.22)
Learning Difficulty	topiramate	Any Time in Pregnancy (Any Use)	11 / 1857	43 / 8887	1.82 (0.76 - 4.37)	1.12 (0.60 - 2.10)	1.17 (0.56 - 2.43)
Learning Difficulty	topiramate	2nd Pregnancy Half (Strict Monotherapy)	*** / 565	43 / 8887	1.09 (0.15 - 7.45)	1.49 (0.82 - 2.71)	0.58 (0.14 - 2.39)
Learning Difficulty	topiramate	1st Pregnancy Half (Strict Monotherapy)	*** / 865	43 / 8887	0.44 (0.04 - 4.41)	1.11 (0.54 - 2.26)	0.41 (0.09 - 1.76)
Learning Difficulty	topiramate	Any Time in Pregnancy (Strict Monotherap	*** / 961	43 / 8887	1.14 (0.25 - 5.17)	1.17 (0.59 - 2.29)	0.59 (0.17 - 1.98)
Intellectual Disability	topiramate	2nd Pregnancy Half (Any Use)	*** / 1036	26 / 8887	2.12 (0.93 - 4.79)	0.86 (0.46 - 1.63)	1.84 (0.75 - 4.51)
Intellectual Disability	topiramate	1st Pregnancy Half (Any Use)	*** / 1689	26 / 8887	2.61 (1.27 - 5.34)	1.02 (0.56 - 1.86)	1.70 (0.76 - 3.83)
Intellectual Disability	topiramate	Any Time in Pregnancy (Any Use)	11 / 1857	26 / 8887	2.61 (1.31 - 5.18)	0.92 (0.50 - 1.70)	1.71 (0.78 - 3.75)
Intellectual Disability	topiramate	2nd Pregnancy Half (Strict Monotherapy)	*** / 565	26 / 8887	2.52 (0.90 - 6.96)	0.92 (0.48 - 1.77)	2.02 (0.67 - 6.13)
Intellectual Disability	topiramate	1st Pregnancy Half (Strict Monotherapy)	*** / 865	26 / 8887	1.86 (0.66 - 5.23)	1.00 (0.52 - 1.92)	1.26 (0.41 - 3.82)
Intellectual Disability	topiramate	Any Time in Pregnancy (Strict Monotherap	*** / 961	26 / 8887	2.26 (0.88 - 5.74)	0.87 (0.44 - 1.68)	1.51 (0.55 - 4.16)

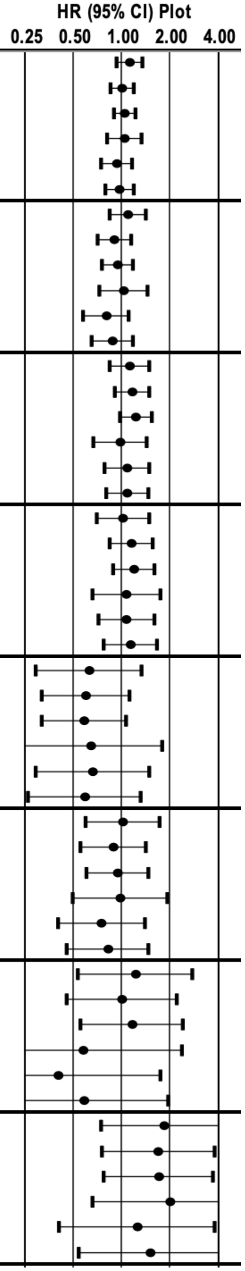

F) Oxcarbazepine

Outcome	Exposure	Analysis	N Cases / Total		Cumulative Incidence in % (95% CI) at Age 8 Years		HR (95% CI)
			Among Exposed	Among Unexposed	Among Exposed	Among Unexposed	
Any NDD	oxcarbazepine	2nd Pregnancy Half (Any Use)	94 / 845	872 / 8887	36.57 (29.82 - 44.31)	34.42 (31.28 - 37.78)	1.09 (0.87 - 1.36)
Any NDD	oxcarbazepine	1st Pregnancy Half (Any Use)	107 / 955	872 / 8887	33.81 (27.46 - 41.15)	35.56 (32.43 - 38.88)	1.03 (0.83 - 1.27)
Any NDD	oxcarbazepine	Any Time in Pregnancy (Any Use)	125 / 1098	872 / 8887	35.36 (29.33 - 42.22)	35.54 (32.57 - 38.69)	1.07 (0.87 - 1.30)
Any NDD	oxcarbazepine	2nd Pregnancy Half (Strict Monotherapy)	51 / 522	872 / 8887	31.32 (23.57 - 40.86)	33.14 (29.77 - 36.78)	1.04 (0.78 - 1.39)
Any NDD	oxcarbazepine	1st Pregnancy Half (Strict Monotherapy)	57 / 572	872 / 8887	31.48 (23.67 - 41.10)	35.36 (32.02 - 38.94)	0.95 (0.72 - 1.25)
Any NDD	oxcarbazepine	Any Time in Pregnancy (Strict Monotherap	68 / 660	872 / 8887	32.04 (24.76 - 40.79)	35.12 (31.99 - 38.45)	1.00 (0.77 - 1.29)
Speech/Language Disorder	oxcarbazepine	2nd Pregnancy Half (Any Use)	44 / 845	456 / 8887	14.42 (10.36 - 19.88)	15.24 (13.24 - 17.51)	0.91 (0.66 - 1.26)
Speech/Language Disorder	oxcarbazepine	1st Pregnancy Half (Any Use)	51 / 955	456 / 8887	14.25 (10.38 - 19.41)	15.67 (13.66 - 17.95)	0.92 (0.68 - 1.24)
Speech/Language Disorder	oxcarbazepine	Any Time in Pregnancy (Any Use)	59 / 1098	456 / 8887	14.99 (11.16 - 19.99)	15.46 (13.55 - 17.62)	0.94 (0.71 - 1.25)
Speech/Language Disorder	oxcarbazepine	2nd Pregnancy Half (Strict Monotherapy)	25 / 522	456 / 8887	13.19 (8.25 - 20.72)	14.69 (12.56 - 17.13)	0.92 (0.61 - 1.39)
Speech/Language Disorder	oxcarbazepine	1st Pregnancy Half (Strict Monotherapy)	30 / 572	456 / 8887	13.84 (8.93 - 21.11)	15.19 (13.05 - 17.65)	0.97 (0.66 - 1.42)
Speech/Language Disorder	oxcarbazepine	Any Time in Pregnancy (Strict Monotherap	35 / 660	456 / 8887	15.36 (10.24 - 22.69)	15.04 (13.03 - 17.33)	0.99 (0.70 - 1.41)
ADHD	oxcarbazepine	2nd Pregnancy Half (Any Use)	48 / 845	366 / 8887	24.99 (18.66 - 32.99)	19.60 (16.87 - 22.70)	1.28 (0.93 - 1.77)
ADHD	oxcarbazepine	1st Pregnancy Half (Any Use)	52 / 955	366 / 8887	23.10 (17.31 - 30.45)	20.16 (17.43 - 23.25)	1.17 (0.85 - 1.60)
ADHD	oxcarbazepine	Any Time in Pregnancy (Any Use)	59 / 1098	366 / 8887	23.04 (17.55 - 29.90)	20.22 (17.61 - 23.16)	1.14 (0.85 - 1.53)
ADHD	oxcarbazepine	2nd Pregnancy Half (Strict Monotherapy)	31 / 522	366 / 8887	28.27 (19.76 - 39.44)	19.15 (16.27 - 22.46)	1.53 (1.04 - 2.26)
ADHD	oxcarbazepine	1st Pregnancy Half (Strict Monotherapy)	33 / 572	366 / 8887	27.23 (19.23 - 37.69)	20.43 (17.54 - 23.73)	1.33 (0.91 - 1.95)
ADHD	oxcarbazepine	Any Time in Pregnancy (Strict Monotherap	38 / 660	366 / 8887	26.54 (19.06 - 36.23)	20.32 (17.61 - 23.39)	1.30 (0.91 - 1.86)
Behavioral Disorder	oxcarbazepine	2nd Pregnancy Half (Any Use)	32 / 845	239 / 8887	13.94 (9.39 - 20.44)	10.96 (9.01 - 13.31)	1.40 (0.94 - 2.07)
Behavioral Disorder	oxcarbazepine	1st Pregnancy Half (Any Use)	39 / 955	239 / 8887	12.46 (8.35 - 18.39)	11.61 (9.61 - 13.99)	1.33 (0.91 - 1.93)
Behavioral Disorder	oxcarbazepine	Any Time in Pregnancy (Any Use)	45 / 1098	239 / 8887	13.49 (9.43 - 19.10)	11.49 (9.60 - 13.73)	1.37 (0.96 - 1.94)
Behavioral Disorder	oxcarbazepine	2nd Pregnancy Half (Strict Monotherapy)	14 / 522	239 / 8887	8.80 (4.93 - 15.44)	10.65 (8.56 - 13.20)	1.07 (0.61 - 1.88)
Behavioral Disorder	oxcarbazepine	1st Pregnancy Half (Strict Monotherapy)	19 / 572	239 / 8887	10.12 (5.71 - 17.59)	11.82 (9.66 - 14.41)	1.12 (0.68 - 1.85)
Behavioral Disorder	oxcarbazepine	Any Time in Pregnancy (Strict Monotherap	23 / 660	239 / 8887	10.94 (6.68 - 17.66)	11.73 (9.71 - 14.13)	1.20 (0.76 - 1.90)
Coordination Disorder	oxcarbazepine	2nd Pregnancy Half (Any Use)	*** / 845	91 / 8887	2.31 (0.92 - 5.73)	1.55 (1.06 - 2.27)	0.82 (0.36 - 1.88)
Coordination Disorder	oxcarbazepine	1st Pregnancy Half (Any Use)	*** / 955	91 / 8887	1.64 (0.63 - 4.24)	1.74 (1.21 - 2.48)	0.76 (0.35 - 1.65)
Coordination Disorder	oxcarbazepine	Any Time in Pregnancy (Any Use)	*** / 1098	91 / 8887	2.17 (0.96 - 4.83)	1.62 (1.13 - 2.30)	0.91 (0.45 - 1.82)
Coordination Disorder	oxcarbazepine	2nd Pregnancy Half (Strict Monotherapy)	*** / 522	91 / 8887	3.47 (1.28 - 9.27)	1.44 (0.93 - 2.23)	1.14 (0.45 - 2.91)
Coordination Disorder	oxcarbazepine	1st Pregnancy Half (Strict Monotherapy)	*** / 572	91 / 8887	2.05 (0.59 - 6.98)	1.63 (1.09 - 2.43)	0.71 (0.25 - 2.01)
Coordination Disorder	oxcarbazepine	Any Time in Pregnancy (Strict Monotherap	*** / 660	91 / 8887	3.01 (1.16 - 7.65)	1.51 (1.01 - 2.24)	1.06 (0.45 - 2.50)
Autism Spectrum Disorder	oxcarbazepine	2nd Pregnancy Half (Any Use)	*** / 845	106 / 8887	3.35 (1.55 - 7.16)	4.12 (3.08 - 5.50)	0.82 (0.41 - 1.64)
Autism Spectrum Disorder	oxcarbazepine	1st Pregnancy Half (Any Use)	*** / 955	106 / 8887	2.00 (0.78 - 5.07)	4.20 (3.16 - 5.57)	0.60 (0.29 - 1.25)
Autism Spectrum Disorder	oxcarbazepine	Any Time in Pregnancy (Any Use)	12 / 1098	106 / 8887	2.84 (1.36 - 5.91)	4.03 (3.06 - 5.31)	0.77 (0.40 - 1.46)
Autism Spectrum Disorder	oxcarbazepine	2nd Pregnancy Half (Strict Monotherapy)	*** / 522	106 / 8887	3.48 (1.19 - 9.98)	3.84 (2.78 - 5.30)	0.64 (0.23 - 1.77)
Autism Spectrum Disorder	oxcarbazepine	1st Pregnancy Half (Strict Monotherapy)	*** / 572	106 / 8887	1.90 (0.41 - 8.60)	4.07 (2.98 - 5.57)	0.38 (0.12 - 1.23)
Autism Spectrum Disorder	oxcarbazepine	Any Time in Pregnancy (Strict Monotherap	*** / 660	106 / 8887	2.59 (0.86 - 7.68)	3.87 (2.86 - 5.23)	0.61 (0.24 - 1.54)
Learning Difficulty	oxcarbazepine	2nd Pregnancy Half (Any Use)	*** / 845	43 / 8887	1.91 (0.60 - 5.99)	1.07 (0.53 - 2.14)	1.09 (0.39 - 3.05)
Learning Difficulty	oxcarbazepine	1st Pregnancy Half (Any Use)	*** / 955	43 / 8887	1.85 (0.59 - 5.74)	1.21 (0.63 - 2.34)	1.25 (0.48 - 3.25)
Learning Difficulty	oxcarbazepine	Any Time in Pregnancy (Any Use)	*** / 1098	43 / 8887	1.59 (0.51 - 4.94)	1.16 (0.62 - 2.19)	1.02 (0.39 - 2.66)
Learning Difficulty	oxcarbazepine	2nd Pregnancy Half (Strict Monotherapy)	*** / 522	43 / 8887	1.68 (0.42 - 6.60)	1.25 (0.61 - 2.56)	1.18 (0.35 - 4.01)
Learning Difficulty	oxcarbazepine	1st Pregnancy Half (Strict Monotherapy)	*** / 572	43 / 8887	1.58 (0.39 - 6.30)	1.27 (0.63 - 2.57)	1.15 (0.33 - 4.02)
Learning Difficulty	oxcarbazepine	Any Time in Pregnancy (Strict Monotherap	*** / 660	43 / 8887	1.36 (0.34 - 5.42)	1.21 (0.62 - 2.36)	0.98 (0.29 - 3.38)
Intellectual Disability	oxcarbazepine	2nd Pregnancy Half (Any Use)	*** / 845	26 / 8887	2.62 (0.99 - 6.82)	0.57 (0.24 - 1.36)	3.30 (1.29 - 8.41)
Intellectual Disability	oxcarbazepine	1st Pregnancy Half (Any Use)	*** / 955	26 / 8887	2.58 (1.00 - 6.60)	0.72 (0.33 - 1.55)	2.72 (1.08 - 6.83)
Intellectual Disability	oxcarbazepine	Any Time in Pregnancy (Any Use)	*** / 1098	26 / 8887	2.06 (0.77 - 5.46)	0.67 (0.32 - 1.42)	2.30 (0.91 - 5.83)
Intellectual Disability	oxcarbazepine	2nd Pregnancy Half (Strict Monotherapy)	*** / 522	26 / 8887	3.01 (0.87 - 10.19)	0.57 (0.23 - 1.40)	2.93 (0.87 - 9.93)
Intellectual Disability	oxcarbazepine	1st Pregnancy Half (Strict Monotherapy)	*** / 572	26 / 8887	2.93 (0.86 - 9.72)	0.65 (0.28 - 1.53)	2.51 (0.75 - 8.44)
Intellectual Disability	oxcarbazepine	Any Time in Pregnancy (Strict Monotherap	*** / 660	26 / 8887	2.32 (0.65 - 8.12)	0.63 (0.28 - 1.41)	2.02 (0.60 - 6.83)

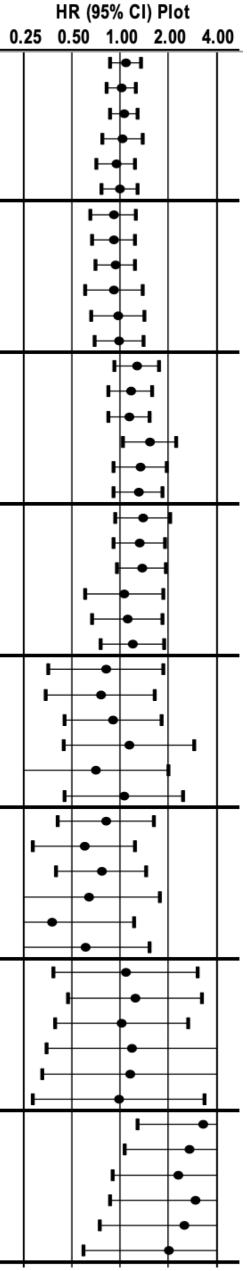

G) Valproate

Outcome	Exposure	Analysis	N Cases / Total Among Exposed	N Cases / Total Among Unexposed	Cumulative Incidence in % (95% CI) at Age 8 Years Among Exposed	Cumulative Incidence in % (95% CI) at Age 8 Years Among Unexposed	HR (95% CI)
Any NDD	valproate	2nd Pregnancy Half (Any Use)	144 / 800	872 / 8887	41.19 (35.28 - 47.68)	35.08 (32.00 - 38.36)	1.29 (1.06 - 1.56)
Any NDD	valproate	1st Pregnancy Half (Any Use)	241 / 1326	872 / 8887	43.97 (38.97 - 49.32)	37.13 (34.19 - 40.24)	1.37 (1.18 - 1.61)
Any NDD	valproate	Any Time in Pregnancy (Any Use)	270 / 1491	872 / 8887	43.53 (38.86 - 48.51)	37.18 (34.30 - 40.22)	1.33 (1.15 - 1.54)
Any NDD	valproate	2nd Pregnancy Half (Strict Monotherapy)	90 / 515	872 / 8887	39.55 (32.57 - 47.41)	34.36 (31.10 - 37.85)	1.25 (0.99 - 1.58)
Any NDD	valproate	1st Pregnancy Half (Strict Monotherapy)	123 / 699	872 / 8887	39.95 (33.65 - 46.95)	36.72 (33.64 - 39.99)	1.29 (1.05 - 1.58)
Any NDD	valproate	Any Time in Pregnancy (Strict Monotherap	141 / 796	872 / 8887	41.10 (35.10 - 47.70)	36.80 (33.79 - 39.99)	1.27 (1.04 - 1.54)
Speech/Language Disorder	valproate	2nd Pregnancy Half (Any Use)	79 / 800	456 / 8887	21.48 (17.15 - 26.71)	14.07 (12.15 - 16.27)	1.52 (1.17 - 1.98)
Speech/Language Disorder	valproate	1st Pregnancy Half (Any Use)	137 / 1326	456 / 8887	23.79 (19.97 - 28.19)	14.90 (13.04 - 17.00)	1.63 (1.32 - 2.02)
Speech/Language Disorder	valproate	Any Time in Pregnancy (Any Use)	147 / 1491	456 / 8887	22.47 (19.01 - 26.45)	14.82 (13.00 - 16.88)	1.54 (1.26 - 1.89)
Speech/Language Disorder	valproate	2nd Pregnancy Half (Strict Monotherapy)	52 / 515	456 / 8887	20.70 (15.66 - 27.09)	13.37 (11.36 - 15.70)	1.60 (1.17 - 2.19)
Speech/Language Disorder	valproate	1st Pregnancy Half (Strict Monotherapy)	73 / 699	456 / 8887	22.80 (18.05 - 28.56)	14.48 (12.52 - 16.71)	1.67 (1.28 - 2.19)
Speech/Language Disorder	valproate	Any Time in Pregnancy (Strict Monotherap	79 / 796	456 / 8887	21.76 (17.39 - 27.02)	14.23 (12.33 - 16.39)	1.58 (1.22 - 2.05)
ADHD	valproate	2nd Pregnancy Half (Any Use)	78 / 800	366 / 8887	26.93 (21.53 - 33.38)	21.76 (19.00 - 24.86)	1.26 (0.97 - 1.64)
ADHD	valproate	1st Pregnancy Half (Any Use)	115 / 1326	366 / 8887	26.43 (21.88 - 31.73)	22.53 (19.85 - 25.50)	1.21 (0.97 - 1.52)
ADHD	valproate	Any Time in Pregnancy (Any Use)	134 / 1491	366 / 8887	26.71 (22.45 - 31.60)	22.62 (20.00 - 25.52)	1.22 (0.99 - 1.52)
ADHD	valproate	2nd Pregnancy Half (Strict Monotherapy)	48 / 515	366 / 8887	25.80 (19.44 - 33.75)	21.44 (18.54 - 24.71)	1.22 (0.88 - 1.68)
ADHD	valproate	1st Pregnancy Half (Strict Monotherapy)	58 / 699	366 / 8887	22.83 (17.30 - 29.78)	22.26 (19.49 - 25.36)	1.10 (0.82 - 1.49)
ADHD	valproate	Any Time in Pregnancy (Strict Monotherap	70 / 796	366 / 8887	24.44 (19.10 - 30.96)	22.51 (19.80 - 25.53)	1.14 (0.86 - 1.49)
Behavioral Disorder	valproate	2nd Pregnancy Half (Any Use)	49 / 800	239 / 8887	16.01 (11.91 - 21.33)	12.02 (10.04 - 14.37)	1.31 (0.94 - 1.82)
Behavioral Disorder	valproate	1st Pregnancy Half (Any Use)	74 / 1326	239 / 8887	14.54 (11.21 - 18.74)	12.72 (10.77 - 14.99)	1.22 (0.92 - 1.62)
Behavioral Disorder	valproate	Any Time in Pregnancy (Any Use)	85 / 1491	239 / 8887	14.78 (11.63 - 18.68)	13.09 (11.16 - 15.33)	1.18 (0.90 - 1.55)
Behavioral Disorder	valproate	2nd Pregnancy Half (Strict Monotherapy)	36 / 515	239 / 8887	18.12 (12.91 - 25.10)	12.29 (10.16 - 14.83)	1.44 (0.99 - 2.10)
Behavioral Disorder	valproate	1st Pregnancy Half (Strict Monotherapy)	44 / 699	239 / 8887	15.18 (10.89 - 20.95)	13.20 (11.12 - 15.64)	1.31 (0.92 - 1.85)
Behavioral Disorder	valproate	Any Time in Pregnancy (Strict Monotherap	53 / 796	239 / 8887	16.37 (12.15 - 21.86)	13.69 (11.62 - 16.09)	1.30 (0.94 - 1.80)
Coordination Disorder	valproate	2nd Pregnancy Half (Any Use)	14 / 800	91 / 8887	2.68 (1.47 - 4.86)	1.51 (0.99 - 2.31)	1.67 (0.89 - 3.13)
Coordination Disorder	valproate	1st Pregnancy Half (Any Use)	30 / 1326	91 / 8887	4.88 (3.20 - 7.40)	1.58 (1.09 - 2.28)	2.34 (1.48 - 3.69)
Coordination Disorder	valproate	Any Time in Pregnancy (Any Use)	33 / 1491	91 / 8887	4.55 (3.05 - 6.78)	1.59 (1.10 - 2.31)	2.23 (1.43 - 3.48)
Coordination Disorder	valproate	2nd Pregnancy Half (Strict Monotherapy)	*** / 515	91 / 8887	2.36 (1.00 - 5.53)	1.49 (0.94 - 2.35)	1.19 (0.52 - 2.75)
Coordination Disorder	valproate	1st Pregnancy Half (Strict Monotherapy)	12 / 699	91 / 8887	3.70 (1.91 - 7.10)	1.42 (0.93 - 2.17)	1.83 (0.96 - 3.50)
Coordination Disorder	valproate	Any Time in Pregnancy (Strict Monotherap	13 / 796	91 / 8887	3.37 (1.78 - 6.35)	1.46 (0.96 - 2.21)	1.68 (0.89 - 3.16)
Autism Spectrum Disorder	valproate	2nd Pregnancy Half (Any Use)	34 / 800	106 / 8887	9.95 (6.96 - 14.12)	3.75 (2.76 - 5.09)	2.60 (1.67 - 4.04)
Autism Spectrum Disorder	valproate	1st Pregnancy Half (Any Use)	49 / 1326	106 / 8887	9.17 (6.78 - 12.34)	3.87 (2.92 - 5.13)	2.30 (1.56 - 3.38)
Autism Spectrum Disorder	valproate	Any Time in Pregnancy (Any Use)	51 / 1491	106 / 8887	8.33 (6.20 - 11.16)	3.86 (2.91 - 5.10)	2.12 (1.45 - 3.09)
Autism Spectrum Disorder	valproate	2nd Pregnancy Half (Strict Monotherapy)	24 / 515	106 / 8887	11.53 (7.61 - 17.26)	3.76 (2.71 - 5.22)	2.89 (1.75 - 4.75)
Autism Spectrum Disorder	valproate	1st Pregnancy Half (Strict Monotherapy)	32 / 699	106 / 8887	11.67 (8.12 - 16.62)	3.85 (2.84 - 5.21)	2.82 (1.81 - 4.38)
Autism Spectrum Disorder	valproate	Any Time in Pregnancy (Strict Monotherap	33 / 796	106 / 8887	10.59 (7.42 - 14.99)	3.92 (2.91 - 5.27)	2.52 (1.63 - 3.90)
Learning Difficulty	valproate	2nd Pregnancy Half (Any Use)	15 / 800	43 / 8887	3.17 (1.42 - 7.03)	1.44 (0.83 - 2.51)	1.49 (0.77 - 2.88)
Learning Difficulty	valproate	1st Pregnancy Half (Any Use)	18 / 1326	43 / 8887	1.64 (0.67 - 3.99)	1.53 (0.91 - 2.58)	1.43 (0.78 - 2.61)
Learning Difficulty	valproate	Any Time in Pregnancy (Any Use)	22 / 1491	43 / 8887	2.30 (1.12 - 4.70)	1.48 (0.88 - 2.48)	1.54 (0.87 - 2.73)
Learning Difficulty	valproate	2nd Pregnancy Half (Strict Monotherapy)	*** / 515	43 / 8887	0.98 (0.17 - 5.42)	1.47 (0.82 - 2.65)	1.11 (0.49 - 2.54)
Learning Difficulty	valproate	1st Pregnancy Half (Strict Monotherapy)	*** / 699	43 / 8887	1.28 (0.36 - 4.48)	1.73 (1.02 - 2.91)	1.20 (0.55 - 2.59)
Learning Difficulty	valproate	Any Time in Pregnancy (Strict Monotherap	11 / 796	43 / 8887	1.02 (0.28 - 3.74)	1.64 (0.97 - 2.76)	1.13 (0.54 - 2.38)
Intellectual Disability	valproate	2nd Pregnancy Half (Any Use)	11 / 800	26 / 8887	1.86 (0.81 - 4.23)	1.05 (0.55 - 1.97)	1.72 (0.77 - 3.83)
Intellectual Disability	valproate	1st Pregnancy Half (Any Use)	18 / 1326	26 / 8887	2.42 (1.35 - 4.32)	0.98 (0.54 - 1.77)	2.16 (1.08 - 4.32)
Intellectual Disability	valproate	Any Time in Pregnancy (Any Use)	18 / 1491	26 / 8887	2.04 (1.12 - 3.70)	0.96 (0.53 - 1.75)	1.80 (0.90 - 3.60)
Intellectual Disability	valproate	2nd Pregnancy Half (Strict Monotherapy)	*** / 515	26 / 8887	1.23 (0.34 - 4.31)	1.19 (0.63 - 2.25)	1.19 (0.44 - 3.25)
Intellectual Disability	valproate	1st Pregnancy Half (Strict Monotherapy)	*** / 699	26 / 8887	1.72 (0.65 - 4.50)	0.99 (0.53 - 1.84)	1.67 (0.72 - 3.92)
Intellectual Disability	valproate	Any Time in Pregnancy (Strict Monotherap	*** / 796	26 / 8887	1.37 (0.50 - 3.71)	0.99 (0.53 - 1.84)	1.32 (0.57 - 3.09)

HR (95% CI) Plot

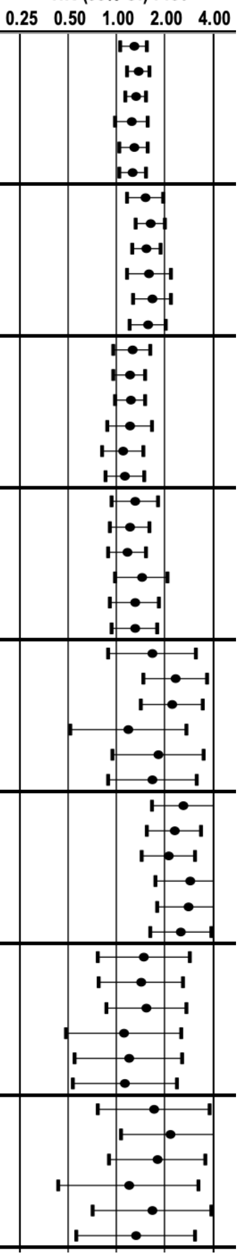

H) Zonisamide

Outcome	Exposure	Analysis	N Cases / Total Among Exposed	N Cases / Total Among Unexposed	Cumulative Incidence in % (95% CI) at Age 8 Years Among Exposed	Cumulative Incidence in % (95% CI) at Age 8 Years Among Unexposed	HR (95% CI)
Any NDD	zonisamide	2nd Pregnancy Half (Any Use)	55 / 446	872 / 8887	43.03 (32.18 - 55.74)	33.02 (29.11 - 37.29)	1.41 (1.06 - 1.88)
Any NDD	zonisamide	1st Pregnancy Half (Any Use)	66 / 522	872 / 8887	41.37 (32.29 - 51.86)	34.79 (31.14 - 38.74)	1.21 (0.93 - 1.58)
Any NDD	zonisamide	Any Time in Pregnancy (Any Use)	73 / 576	872 / 8887	42.39 (33.54 - 52.50)	34.31 (30.83 - 38.07)	1.30 (1.01 - 1.67)
Any NDD	zonisamide	2nd Pregnancy Half (Strict Monotherapy)	20 / 207	840 / 8593	27.95 (15.95 - 46.14)	30.99 (26.66 - 35.84)	1.15 (0.73 - 1.81)
Any NDD	zonisamide	1st Pregnancy Half (Strict Monotherapy)	21 / 219	872 / 8887	27.84 (16.76 - 44.05)	34.31 (30.20 - 38.82)	0.97 (0.62 - 1.50)
Any NDD	zonisamide	Any Time in Pregnancy (Strict Monotherap	24 / 246	872 / 8887	29.42 (18.31 - 45.13)	33.41 (29.57 - 37.60)	1.05 (0.69 - 1.60)
Speech/Language Disorder	zonisamide	2nd Pregnancy Half (Any Use)	30 / 446	456 / 8887	15.72 (10.66 - 22.84)	14.52 (12.24 - 17.18)	1.33 (0.90 - 1.96)
Speech/Language Disorder	zonisamide	1st Pregnancy Half (Any Use)	34 / 522	456 / 8887	14.35 (10.07 - 20.25)	15.28 (13.06 - 17.83)	1.15 (0.80 - 1.66)
Speech/Language Disorder	zonisamide	Any Time in Pregnancy (Any Use)	37 / 576	456 / 8887	14.56 (10.36 - 20.25)	15.17 (13.04 - 17.60)	1.18 (0.83 - 1.68)
Speech/Language Disorder	zonisamide	2nd Pregnancy Half (Strict Monotherapy)	12 / 207	443 / 8593	12.43 (7.02 - 21.49)	12.66 (10.35 - 15.44)	1.21 (0.67 - 2.19)
Speech/Language Disorder	zonisamide	1st Pregnancy Half (Strict Monotherapy)	12 / 219	456 / 8887	11.40 (6.44 - 19.74)	13.46 (11.21 - 16.12)	1.04 (0.58 - 1.87)
Speech/Language Disorder	zonisamide	Any Time in Pregnancy (Strict Monotherap	13 / 246	456 / 8887	11.23 (6.49 - 19.07)	13.44 (11.30 - 15.94)	1.05 (0.59 - 1.84)
ADHD	zonisamide	2nd Pregnancy Half (Any Use)	23 / 446	366 / 8887	23.05 (14.57 - 35.35)	18.99 (15.61 - 23.00)	1.33 (0.85 - 2.06)
ADHD	zonisamide	1st Pregnancy Half (Any Use)	30 / 522	366 / 8887	23.53 (16.20 - 33.44)	19.50 (16.33 - 23.19)	1.23 (0.83 - 1.83)
ADHD	zonisamide	Any Time in Pregnancy (Any Use)	34 / 576	366 / 8887	25.50 (18.04 - 35.31)	19.29 (16.24 - 22.82)	1.34 (0.93 - 1.95)
ADHD	zonisamide	2nd Pregnancy Half (Strict Monotherapy)	*** / 207	346 / 8593	9.81 (3.24 - 27.65)	19.07 (15.26 - 23.69)	0.84 (0.38 - 1.86)
ADHD	zonisamide	1st Pregnancy Half (Strict Monotherapy)	*** / 219	366 / 8887	14.10 (6.16 - 30.45)	20.34 (16.76 - 24.56)	0.88 (0.44 - 1.75)
ADHD	zonisamide	Any Time in Pregnancy (Strict Monotherap	*** / 246	366 / 8887	14.73 (6.83 - 30.16)	20.30 (16.88 - 24.30)	0.93 (0.48 - 1.80)
Behavioral Disorder	zonisamide	2nd Pregnancy Half (Any Use)	16 / 446	239 / 8887	16.73 (9.60 - 28.26)	9.03 (6.84 - 11.88)	1.79 (1.04 - 3.06)
Behavioral Disorder	zonisamide	1st Pregnancy Half (Any Use)	18 / 522	239 / 8887	14.02 (8.44 - 22.80)	9.79 (7.66 - 12.47)	1.29 (0.78 - 2.13)
Behavioral Disorder	zonisamide	Any Time in Pregnancy (Any Use)	22 / 576	239 / 8887	15.18 (9.55 - 23.66)	9.72 (7.66 - 12.29)	1.55 (0.98 - 2.47)
Behavioral Disorder	zonisamide	2nd Pregnancy Half (Strict Monotherapy)	*** / 207	228 / 8593	15.55 (5.83 - 37.82)	8.13 (5.82 - 11.30)	1.42 (0.58 - 3.48)
Behavioral Disorder	zonisamide	1st Pregnancy Half (Strict Monotherapy)	*** / 219	239 / 8887	12.48 (4.57 - 31.59)	9.43 (7.13 - 12.42)	0.80 (0.30 - 2.13)
Behavioral Disorder	zonisamide	Any Time in Pregnancy (Strict Monotherap	*** / 246	239 / 8887	14.60 (6.16 - 32.40)	8.92 (6.75 - 11.75)	1.23 (0.54 - 2.77)
Coordination Disorder	zonisamide	2nd Pregnancy Half (Any Use)	*** / 446	91 / 8887	5.27 (1.90 - 14.15)	1.86 (1.21 - 2.85)	1.43 (0.65 - 3.16)
Coordination Disorder	zonisamide	1st Pregnancy Half (Any Use)	*** / 522	91 / 8887	4.24 (1.62 - 10.82)	2.08 (1.44 - 3.01)	1.17 (0.54 - 2.52)
Coordination Disorder	zonisamide	Any Time in Pregnancy (Any Use)	*** / 576	91 / 8887	4.40 (1.81 - 10.50)	1.96 (1.33 - 2.87)	1.38 (0.67 - 2.81)
Coordination Disorder	zonisamide	2nd Pregnancy Half (Strict Monotherapy)	*** / 207	89 / 8593	0.43 (0.04 - 4.16)	1.63 (0.98 - 2.69)	0.37 (0.05 - 2.74)
Coordination Disorder	zonisamide	1st Pregnancy Half (Strict Monotherapy)	*** / 219	91 / 8887	1.01 (0.24 - 4.21)	1.85 (1.23 - 2.80)	0.72 (0.17 - 3.10)
Coordination Disorder	zonisamide	Any Time in Pregnancy (Strict Monotherap	*** / 246	91 / 8887	0.89 (0.21 - 3.74)	1.66 (1.08 - 2.54)	0.74 (0.18 - 3.15)
Autism Spectrum Disorder	zonisamide	2nd Pregnancy Half (Any Use)	*** / 446	106 / 8887	4.95 (2.02 - 11.88)	4.04 (2.84 - 5.73)	1.08 (0.46 - 2.53)
Autism Spectrum Disorder	zonisamide	1st Pregnancy Half (Any Use)	*** / 522	106 / 8887	4.79 (2.29 - 9.90)	4.36 (3.18 - 5.97)	1.12 (0.52 - 2.38)
Autism Spectrum Disorder	zonisamide	Any Time in Pregnancy (Any Use)	*** / 576	106 / 8887	5.01 (2.49 - 9.96)	4.28 (3.15 - 5.79)	1.15 (0.56 - 2.36)
Autism Spectrum Disorder	zonisamide	2nd Pregnancy Half (Strict Monotherapy)	*** / 207	102 / 8593	2.58 (0.66 - 9.78)	3.80 (2.50 - 5.76)	0.83 (0.20 - 3.44)
Autism Spectrum Disorder	zonisamide	1st Pregnancy Half (Strict Monotherapy)	*** / 219	106 / 8887	3.59 (1.20 - 10.50)	4.02 (2.77 - 5.82)	1.11 (0.34 - 3.57)
Autism Spectrum Disorder	zonisamide	Any Time in Pregnancy (Strict Monotherap	*** / 246	106 / 8887	3.25 (1.08 - 9.61)	4.28 (3.04 - 6.01)	0.92 (0.28 - 2.97)
Learning Difficulty	zonisamide	2nd Pregnancy Half (Any Use)	*	*	*	*	*
Learning Difficulty	zonisamide	1st Pregnancy Half (Any Use)	*** / 522	43 / 8887	0.00 (0.00 - 0.00)	1.66 (0.84 - 3.28)	0.37 (0.05 - 2.80)
Learning Difficulty	zonisamide	Any Time in Pregnancy (Any Use)	*** / 576	43 / 8887	0.00 (0.00 - 0.00)	1.66 (0.87 - 3.16)	0.33 (0.04 - 2.46)
Learning Difficulty	zonisamide	2nd Pregnancy Half (Strict Monotherapy)	*	*	*	*	*
Learning Difficulty	zonisamide	1st Pregnancy Half (Strict Monotherapy)	*	*	*	*	*
Learning Difficulty	zonisamide	Any Time in Pregnancy (Strict Monotherap	*	*	*	*	*
Intellectual Disability	zonisamide	2nd Pregnancy Half (Any Use)	*** / 446	26 / 8887	5.17 (1.56 - 16.38)	0.56 (0.21 - 1.50)	4.50 (1.43 - 14.18)
Intellectual Disability	zonisamide	1st Pregnancy Half (Any Use)	*** / 522	26 / 8887	4.97 (1.95 - 12.36)	0.66 (0.28 - 1.53)	4.77 (1.73 - 13.14)
Intellectual Disability	zonisamide	Any Time in Pregnancy (Any Use)	*** / 576	26 / 8887	4.70 (1.83 - 11.74)	0.64 (0.27 - 1.50)	4.62 (1.71 - 12.49)
Intellectual Disability	zonisamide	2nd Pregnancy Half (Strict Monotherapy)	*** / 207	23 / 8593	6.89 (1.47 - 29.09)	0.55 (0.17 - 1.83)	6.02 (1.34 - 27.09)
Intellectual Disability	zonisamide	1st Pregnancy Half (Strict Monotherapy)	*** / 219	26 / 8887	5.70 (1.22 - 24.39)	0.73 (0.30 - 1.76)	3.87 (0.86 - 17.38)
Intellectual Disability	zonisamide	Any Time in Pregnancy (Strict Monotherap	*** / 246	26 / 8887	5.42 (1.15 - 23.53)	0.73 (0.30 - 1.78)	3.62 (0.81 - 16.24)

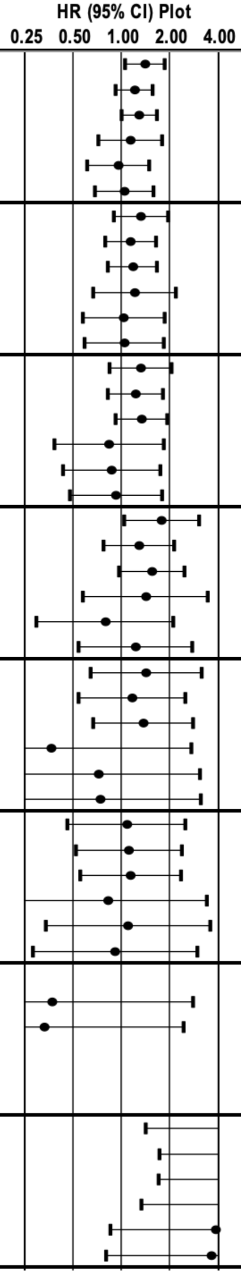

I) Phenobarbital

Outcome	Exposure	Analysis	N Cases / Total Among Exposed	N Cases / Total Among Unexposed	Cumulative Incidence in % (95% CI) at Age 8 Years Among Exposed	Cumulative Incidence in % (95% CI) at Age 8 Years Among Unexposed	HR (95% CI)
Any NDD	phenobarbital	2nd Pregnancy Half (Any Use)	60 / 416	872 / 8887	27.56 (18.98 - 38.99)	29.10 (22.30 - 37.41)	0.92 (0.61 - 1.39)
Any NDD	phenobarbital	1st Pregnancy Half (Any Use)	66 / 448	872 / 8887	32.20 (25.24 - 40.49)	33.41 (29.95 - 37.17)	0.98 (0.75 - 1.28)
Any NDD	phenobarbital	Any Time in Pregnancy (Any Use)	81 / 545	872 / 8887	27.85 (19.50 - 38.81)	29.74 (22.84 - 38.16)	0.91 (0.61 - 1.36)
Any NDD	phenobarbital	2nd Pregnancy Half (Strict Monotherapy)	****	****	****	****	****
Any NDD	phenobarbital	1st Pregnancy Half (Strict Monotherapy)	36 / 211	872 / 8887	32.31 (23.19 - 43.85)	33.63 (29.62 - 38.03)	1.04 (0.73 - 1.47)
Any NDD	phenobarbital	Any Time in Pregnancy (Strict Monotherap	41 / 249	872 / 8887	27.96 (17.51 - 42.79)	25.57 (17.11 - 37.16)	1.00 (0.59 - 1.70)
Speech/Language Disorder	phenobarbital	2nd Pregnancy Half (Any Use)	25 / 416	456 / 8887	11.23 (6.54 - 18.93)	12.51 (8.59 - 18.04)	0.89 (0.48 - 1.66)
Speech/Language Disorder	phenobarbital	1st Pregnancy Half (Any Use)	26 / 448	456 / 8887	11.33 (7.51 - 16.92)	12.98 (10.91 - 15.41)	0.81 (0.53 - 1.24)
Speech/Language Disorder	phenobarbital	Any Time in Pregnancy (Any Use)	32 / 545	456 / 8887	10.77 (6.32 - 18.04)	12.70 (8.72 - 18.31)	0.83 (0.45 - 1.52)
Speech/Language Disorder	phenobarbital	2nd Pregnancy Half (Strict Monotherapy)	****	****	****	****	****
Speech/Language Disorder	phenobarbital	1st Pregnancy Half (Strict Monotherapy)	12 / 211	456 / 8887	9.75 (5.38 - 17.35)	13.63 (11.13 - 16.62)	0.74 (0.41 - 1.36)
Speech/Language Disorder	phenobarbital	Any Time in Pregnancy (Strict Monotherap	12 / 249	456 / 8887	7.99 (3.42 - 18.07)	10.35 (5.84 - 17.99)	0.75 (0.34 - 1.66)
ADHD	phenobarbital	2nd Pregnancy Half (Any Use)	39 / 416	366 / 8887	22.38 (14.00 - 34.65)	15.30 (9.85 - 23.35)	1.11 (0.66 - 1.88)
ADHD	phenobarbital	1st Pregnancy Half (Any Use)	39 / 448	366 / 8887	20.39 (14.35 - 28.50)	21.64 (18.49 - 25.25)	1.03 (0.72 - 1.45)
ADHD	phenobarbital	Any Time in Pregnancy (Any Use)	49 / 545	366 / 8887	22.12 (14.05 - 33.83)	15.98 (10.33 - 24.25)	1.07 (0.64 - 1.79)
ADHD	phenobarbital	2nd Pregnancy Half (Strict Monotherapy)	****	****	****	****	****
ADHD	phenobarbital	1st Pregnancy Half (Strict Monotherapy)	23 / 211	366 / 8887	20.96 (13.13 - 32.51)	20.25 (16.76 - 24.35)	1.22 (0.78 - 1.89)
ADHD	phenobarbital	Any Time in Pregnancy (Strict Monotherap	27 / 249	366 / 8887	20.03 (10.92 - 35.08)	11.63 (5.58 - 23.37)	1.26 (0.58 - 2.77)
Behavioral Disorder	phenobarbital	2nd Pregnancy Half (Any Use)	24 / 416	239 / 8887	11.98 (6.42 - 21.77)	9.66 (5.70 - 16.12)	1.56 (0.83 - 2.92)
Behavioral Disorder	phenobarbital	1st Pregnancy Half (Any Use)	25 / 448	239 / 8887	13.68 (8.97 - 20.58)	10.59 (8.46 - 13.22)	1.26 (0.81 - 1.96)
Behavioral Disorder	phenobarbital	Any Time in Pregnancy (Any Use)	31 / 545	239 / 8887	12.01 (6.65 - 21.17)	10.36 (6.21 - 17.02)	1.44 (0.77 - 2.68)
Behavioral Disorder	phenobarbital	2nd Pregnancy Half (Strict Monotherapy)	****	****	****	****	****
Behavioral Disorder	phenobarbital	1st Pregnancy Half (Strict Monotherapy)	18 / 211	239 / 8887	18.33 (11.30 - 28.95)	10.24 (7.86 - 13.28)	1.83 (1.10 - 3.05)
Behavioral Disorder	phenobarbital	Any Time in Pregnancy (Strict Monotherap	21 / 249	239 / 8887	16.03 (8.40 - 29.37)	9.60 (4.53 - 19.76)	2.32 (1.02 - 5.28)
Coordination Disorder	phenobarbital	2nd Pregnancy Half (Any Use)	*** / 416	91 / 8887	2.50 (0.80 - 7.69)	0.98 (0.23 - 4.15)	2.30 (0.54 - 9.84)
Coordination Disorder	phenobarbital	1st Pregnancy Half (Any Use)	*** / 448	91 / 8887	0.70 (0.16 - 2.95)	1.30 (0.78 - 2.16)	0.44 (0.11 - 1.87)
Coordination Disorder	phenobarbital	Any Time in Pregnancy (Any Use)	*** / 545	91 / 8887	2.36 (0.79 - 6.97)	0.84 (0.18 - 3.94)	2.54 (0.63 - 10.31)
Coordination Disorder	phenobarbital	2nd Pregnancy Half (Strict Monotherapy)	****	****	****	****	****
Coordination Disorder	phenobarbital	1st Pregnancy Half (Strict Monotherapy)	*	*	*	*	*
Coordination Disorder	phenobarbital	Any Time in Pregnancy (Strict Monotherap	*	*	*	*	*
Autism Spectrum Disorder	phenobarbital	2nd Pregnancy Half (Any Use)	*** / 416	106 / 8887	3.93 (1.38 - 10.94)	2.18 (0.84 - 5.58)	1.60 (0.49 - 5.24)
Autism Spectrum Disorder	phenobarbital	1st Pregnancy Half (Any Use)	*** / 448	106 / 8887	2.21 (0.86 - 5.62)	3.14 (2.14 - 4.61)	0.88 (0.39 - 1.98)
Autism Spectrum Disorder	phenobarbital	Any Time in Pregnancy (Any Use)	*** / 545	106 / 8887	3.37 (1.11 - 9.95)	2.07 (0.75 - 5.58)	1.44 (0.41 - 5.03)
Autism Spectrum Disorder	phenobarbital	2nd Pregnancy Half (Strict Monotherapy)	****	****	****	****	****
Autism Spectrum Disorder	phenobarbital	1st Pregnancy Half (Strict Monotherapy)	*** / 211	106 / 8887	1.40 (0.32 - 6.01)	3.00 (1.90 - 4.71)	0.82 (0.25 - 2.63)
Autism Spectrum Disorder	phenobarbital	Any Time in Pregnancy (Strict Monotherap	*** / 249	106 / 8887	0.43 (0.02 - 11.22)	1.98 (0.51 - 7.49)	0.27 (0.03 - 2.36)
Learning Difficulty	phenobarbital	2nd Pregnancy Half (Any Use)	*** / 416	43 / 8887	0.76 (0.05 - 11.50)	1.22 (0.35 - 4.22)	0.27 (0.07 - 0.98)
Learning Difficulty	phenobarbital	1st Pregnancy Half (Any Use)	*** / 448	43 / 8887	3.07 (1.16 - 7.98)	1.64 (0.90 - 2.95)	1.37 (0.55 - 3.41)
Learning Difficulty	phenobarbital	Any Time in Pregnancy (Any Use)	*** / 545	43 / 8887	0.84 (0.08 - 8.59)	1.20 (0.34 - 4.19)	0.41 (0.11 - 1.58)
Learning Difficulty	phenobarbital	2nd Pregnancy Half (Strict Monotherapy)	****	****	****	****	****
Learning Difficulty	phenobarbital	1st Pregnancy Half (Strict Monotherapy)	*** / 211	43 / 8887	4.89 (1.64 - 14.06)	1.75 (0.93 - 3.25)	1.69 (0.54 - 5.25)
Learning Difficulty	phenobarbital	Any Time in Pregnancy (Strict Monotherap	*** / 249	43 / 8887	1.35 (0.10 - 16.25)	1.41 (0.31 - 6.20)	0.47 (0.09 - 2.32)
Intellectual Disability	phenobarbital	2nd Pregnancy Half (Any Use)	*** / 416	26 / 8887	2.20 (0.57 - 8.32)	0.43 (0.03 - 6.42)	3.70 (0.85 - 16.11)
Intellectual Disability	phenobarbital	1st Pregnancy Half (Any Use)	*** / 448	26 / 8887	1.82 (0.71 - 4.58)	1.19 (0.61 - 2.30)	1.92 (0.71 - 5.19)
Intellectual Disability	phenobarbital	Any Time in Pregnancy (Any Use)	*** / 545	26 / 8887	2.16 (0.59 - 7.77)	0.40 (0.02 - 6.84)	4.28 (1.18 - 15.55)
Intellectual Disability	phenobarbital	2nd Pregnancy Half (Strict Monotherapy)	****	****	****	****	****
Intellectual Disability	phenobarbital	1st Pregnancy Half (Strict Monotherapy)	*** / 211	26 / 8887	1.49 (0.38 - 5.81)	1.46 (0.74 - 2.87)	1.71 (0.46 - 6.34)
Intellectual Disability	phenobarbital	Any Time in Pregnancy (Strict Monotherap	*** / 249	26 / 8887	1.38 (0.23 - 7.98)	0.29 (0.00 - 29.22)	6.00 (1.22 - 29.42)

HR (95% CI) Plot

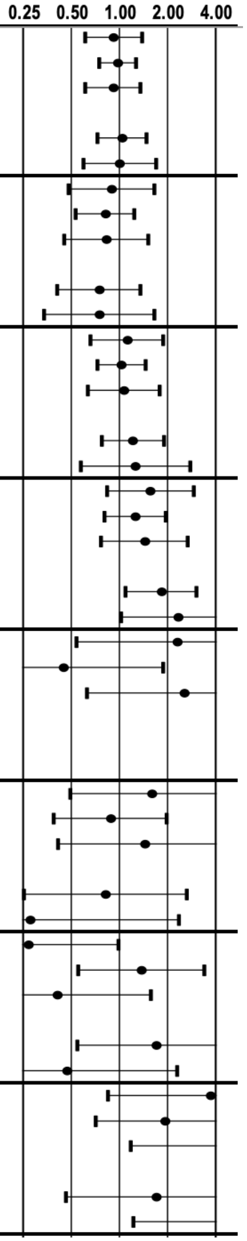

J) Lacosamide

Outcome	Exposure	Analysis	N Cases / Total: Among Exposed	N Cases / Total: Among Unexposed	Cumulative Incidence in % (95% CI) at Age 8 Years: Among Exposed	Cumulative Incidence in % (95% CI) at Age 8 Years: Among Unexposed	HR (95% CI)
Any NDD	lacosamide	2nd Pregnancy Half (Any Use)	13 / 219	462 / 6175	24.28 (13.26 - 41.94)	25.22 (18.19 - 34.34)	1.03 (0.57 - 1.84)
Any NDD	lacosamide	1st Pregnancy Half (Any Use)	13 / 222	872 / 8887	43.83 (17.77 - 81.75)	35.10 (27.35 - 44.29)	0.91 (0.51 - 1.63)
Any NDD	lacosamide	Any Time in Pregnancy (Any Use)	16 / 270	872 / 8887	38.94 (16.62 - 73.78)	35.04 (28.41 - 42.69)	0.95 (0.57 - 1.60)
Any NDD	lacosamide	2nd Pregnancy Half (Strict Monotherapy)	****	****	****	****	****
Any NDD	lacosamide	1st Pregnancy Half (Strict Monotherapy)	****	****	****	****	****
Any NDD	lacosamide	Any Time in Pregnancy (Strict Monotherap	****	****	****	****	****
Speech/Language Disorder	lacosamide	2nd Pregnancy Half (Any Use)	*** / 219	283 / 6175	11.06 (5.20 - 22.70)	14.86 (11.11 - 19.73)	0.77 (0.37 - 1.63)
Speech/Language Disorder	lacosamide	1st Pregnancy Half (Any Use)	*** / 222	456 / 8887	7.19 (3.18 - 15.79)	15.83 (12.35 - 20.17)	0.53 (0.23 - 1.23)
Speech/Language Disorder	lacosamide	Any Time in Pregnancy (Any Use)	*** / 270	456 / 8887	9.84 (4.89 - 19.29)	15.64 (12.45 - 19.55)	0.70 (0.35 - 1.39)
Speech/Language Disorder	lacosamide	2nd Pregnancy Half (Strict Monotherapy)	****	****	****	****	****
Speech/Language Disorder	lacosamide	1st Pregnancy Half (Strict Monotherapy)	****	****	****	****	****
Speech/Language Disorder	lacosamide	Any Time in Pregnancy (Strict Monotherap	****	****	****	****	****
ADHD	lacosamide	2nd Pregnancy Half (Any Use)	*** / 219	116 / 6175	38.27 (8.71 - 92.23)	10.30 (4.80 - 21.35)	2.66 (0.77 - 9.21)
ADHD	lacosamide	1st Pregnancy Half (Any Use)	*** / 222	366 / 8887	30.62 (7.42 - 82.35)	19.86 (13.14 - 29.39)	1.71 (0.54 - 5.37)
ADHD	lacosamide	Any Time in Pregnancy (Any Use)	*** / 270	366 / 8887	25.11 (5.82 - 75.18)	19.71 (13.89 - 27.57)	1.30 (0.41 - 4.11)
ADHD	lacosamide	2nd Pregnancy Half (Strict Monotherapy)	****	****	****	****	****
ADHD	lacosamide	1st Pregnancy Half (Strict Monotherapy)	****	****	****	****	****
ADHD	lacosamide	Any Time in Pregnancy (Strict Monotherap	****	****	****	****	****
Behavioral Disorder	lacosamide	2nd Pregnancy Half (Any Use)	*** / 219	95 / 6175	3.99 (0.60 - 24.11)	5.31 (2.51 - 11.07)	0.65 (0.09 - 4.72)
Behavioral Disorder	lacosamide	1st Pregnancy Half (Any Use)	*** / 222	239 / 8887	31.78 (7.58 - 84.35)	8.11 (4.70 - 13.81)	2.35 (0.69 - 8.05)
Behavioral Disorder	lacosamide	Any Time in Pregnancy (Any Use)	*** / 270	239 / 8887	25.87 (5.94 - 76.86)	8.42 (5.34 - 13.15)	1.65 (0.47 - 5.81)
Behavioral Disorder	lacosamide	2nd Pregnancy Half (Strict Monotherapy)	****	****	****	****	****
Behavioral Disorder	lacosamide	1st Pregnancy Half (Strict Monotherapy)	****	****	****	****	****
Behavioral Disorder	lacosamide	Any Time in Pregnancy (Strict Monotherap	****	****	****	****	****
Coordination Disorder	lacosamide	2nd Pregnancy Half (Any Use)	*** / 219	68 / 6175	0.60 (0.09 - 3.80)	2.38 (1.17 - 4.82)	0.48 (0.06 - 3.60)
Coordination Disorder	lacosamide	1st Pregnancy Half (Any Use)	*** / 222	91 / 8887	0.58 (0.09 - 3.70)	2.80 (1.72 - 4.54)	0.37 (0.05 - 2.78)
Coordination Disorder	lacosamide	Any Time in Pregnancy (Any Use)	*** / 270	91 / 8887	0.48 (0.07 - 3.06)	2.47 (1.51 - 4.05)	0.36 (0.05 - 2.64)
Coordination Disorder	lacosamide	2nd Pregnancy Half (Strict Monotherapy)	****	****	****	****	****
Coordination Disorder	lacosamide	1st Pregnancy Half (Strict Monotherapy)	****	****	****	****	****
Coordination Disorder	lacosamide	Any Time in Pregnancy (Strict Monotherap	****	****	****	****	****
Autism Spectrum Disorder	lacosamide	2nd Pregnancy Half (Any Use)	*** / 219	65 / 6175	0.77 (0.12 - 4.96)	3.50 (1.52 - 7.97)	0.69 (0.09 - 5.23)
Autism Spectrum Disorder	lacosamide	1st Pregnancy Half (Any Use)	*** / 222	106 / 8887	0.76 (0.12 - 4.83)	3.81 (2.09 - 6.91)	0.64 (0.09 - 4.64)
Autism Spectrum Disorder	lacosamide	Any Time in Pregnancy (Any Use)	*** / 270	106 / 8887	0.61 (0.09 - 3.94)	4.21 (2.54 - 6.95)	0.45 (0.06 - 3.29)
Autism Spectrum Disorder	lacosamide	2nd Pregnancy Half (Strict Monotherapy)	****	****	****	****	****
Autism Spectrum Disorder	lacosamide	1st Pregnancy Half (Strict Monotherapy)	****	****	****	****	****
Autism Spectrum Disorder	lacosamide	Any Time in Pregnancy (Strict Monotherap	****	****	****	****	****
Learning Difficulty	lacosamide	2nd Pregnancy Half (Any Use)	*	*	*	*	*
Learning Difficulty	lacosamide	1st Pregnancy Half (Any Use)	*	*	*	*	*
Learning Difficulty	lacosamide	Any Time in Pregnancy (Any Use)	*	*	*	*	*
Learning Difficulty	lacosamide	2nd Pregnancy Half (Strict Monotherapy)	****	****	****	****	****
Learning Difficulty	lacosamide	1st Pregnancy Half (Strict Monotherapy)	****	****	****	****	****
Learning Difficulty	lacosamide	Any Time in Pregnancy (Strict Monotherap	****	****	****	****	****
Intellectual Disability	lacosamide	2nd Pregnancy Half (Any Use)	*	*	*	*	*
Intellectual Disability	lacosamide	1st Pregnancy Half (Any Use)	*	*	*	*	*
Intellectual Disability	lacosamide	Any Time in Pregnancy (Any Use)	*	*	*	*	*
Intellectual Disability	lacosamide	2nd Pregnancy Half (Strict Monotherapy)	****	****	****	****	****
Intellectual Disability	lacosamide	1st Pregnancy Half (Strict Monotherapy)	****	****	****	****	****
Intellectual Disability	lacosamide	Any Time in Pregnancy (Strict Monotherap	****	****	****	****	****

HR (95% CI) Plot

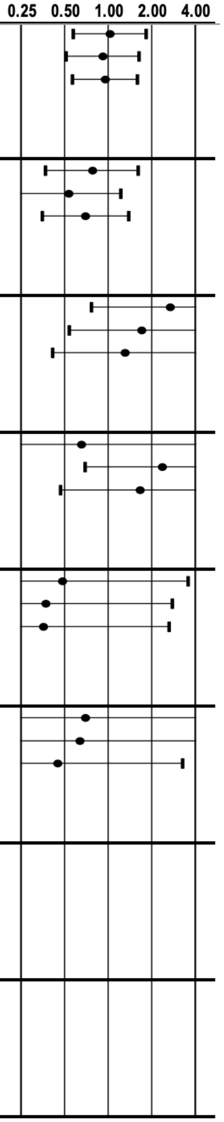

Abbreviation: ADHD, attention deficit hyperactivity disorder; CI, confidence interval; HR, hazard ratio; N, number; NDD, neurodevelopmental disorder.
Individual medications are sorted by the number of exposed pregnancies in both cohorts combined; individual neurodevelopmental disorders are sorted based on the absolute risk observed in the overall population at 8 years of age.
*No results presented due to 0 exposed cases.
***Cell size <11 suppressed in accordance with CMS' cell suppression policy.
****Analyses not conducted as exposure count was <200 for respective comparison.

eTable 1. Counts of Exposure and Reference Groups for Main and Sensitivity Analyses.

	MAIN ANALYSIS	SENSITIVITY ANALYSES						
**Exposure/Reference**	**>=1 Dispensing in Second Pregnancy Half**	**Monotherapy: >=1 Dispensing to Only One ASM in Second Pregnancy Half**	**Polytherapy: >=1 Dispensing to Two ASMs in Second Pregnancy Half, No Valproate**	**Strict Monotherapy: >=1 Dispensing to One ASM in Second Pregnancy Half, No Other ASM Anytime in Pregnancy**	**>=2 Dispensings in Second Pregnancy Half**	**>=1 Dispensing in Second Pregnancy Half, Full Cohort (No Epilepsy Restriction)**	**>=1 Dispensing in Second Pregnancy Half, High Dose Users**	**>=1 Dispensing in Second Pregnancy Half, Low Dose Users**
Unexposed	8,887	8,887	8,887	8,887	8,887	-	8,887	8,887
Levetiracetam	5,261	3,852	1,343	3,360	4,067	5,487	3,036	1,879
Lamotrigine	4,299	3,200	1,043	2,864	3,606	7,381 (ref)[1]	2,542	1,489
Carbamazepine	1,924	1,456	435	1,333	1,545	2,211	1,200	508
Phenytoin	1,595	1,177	382	1,060	1,172	1,702	789	642
Topiramate	1,036	626	383	565	781	2,494	617	363
Oxcarbazepine	845	579	254	522	685	1,355	454	332
Valproate	800	561	-	515	568	1,392	509	209
Zonisamide	446	225	211	207	355	508	276	*
Phenobarbital	416	255	*	196	319	934	221	*
Lacosamide	219	*	*	*	*	226	*	*

	MAIN ANALYSIS	EXPLORATORY ANALYSES			
**Exposure/Reference**	**>=1 Dispensing in Second Pregnancy Half**	**>=1 Dispensing Any Time in Pregnancy**	**Strict Monotherapy: >=1 Dispensing to One ASM Any Time in Pregnancy, No Other ASM Anytime in Pregnancy**	**>=1 Dispensing in First Pregnancy Half**	**Strict Monotherapy: >=1 Dispensing to One ASM in First Pregnancy Half, No Other ASM Anytime in Pregnancy**
Unexposed	8,887	8,887	8,887	8,887	8,887
Levetiracetam	5,261	6,162	3,929	4,890	3,045
Lamotrigine	4,299	5,040	3,273	4,440	2,895
Carbamazepine	1,924	2,374	1,548	2,074	1,354
Phenytoin	1,595	2,295	1,390	1,867	1,102
Topiramate	1,036	1,857	961	1,689	865
Oxcarbazepine	845	1,098	660	955	572
Valproate	800	1,491	796	1,326	699
Zonisamide	446	576	246	522	219
Phenobarbital	416	545	249	448	211
Lacosamide	219	270	*	222	*

Note: Numbers represent pregnancy counts from the publicly and the commercially insured cohort combined.
Abbreviations: ASM, antiseizure medication; MarketScan, Merative MarketScan Commercial Claims and Encounters Database; MAX/TAF, Medicaid Analytic eXtract/Transformed Medicaid Statistical Information System Analytic Files.
[1] The lamotrigine reference group was additionally restricted to those only exposed to lamotrigine but no other antiseizure medication during the second half of pregnancy.
*Analyses not conducted due to exposed counts <200.

eTable 2. Pregnancy Counts with Antiseizure Medication Exposure of Interest by Timing of Exposure During Pregnancy.

	Pregnancy Counts With >=1 Medication Dispensing During Time Period:					
**Exposure**	**Any Time in Pregnancy**	**First Pregnancy Half (Irrespective of Later Exposure)**	**Second Pregnancy Half (Irrespective of Earlier Exposure)**	**First Pregnancy Half Only**	**Second Pregnancy Half Only**	**First And Second Pregnancy Half**
Levetiracetam	6,162	4,890	5,261	901	1,272	3,989
Lamotrigine	5,040	4,440	4,299	741	600	3,699
Carbamazepine	2,374	2,074	1,924	450	300	1,624
Phenytoin	2,295	1,867	1,595	700	428	1,167
Topiramate	1,857	1,689	1,036	821	168	868
Oxcarbazepine	1,098	955	845	253	143	702
Valproate	1,491	1,326	800	691	165	635
Zonisamide	576	522	446	130	54	392
Phenobarbital	545	448	416	129	97	319
Lacosamide	270	222	219	51	48	171

Note: Numbers represent pregnancy counts from the publicly and the commercially insured cohort combined.

eTable 3. Definition of Neurodevelopmental Disorders.[1]

Outcome	Algorithm	ICD-9 Dx	ICD-10 Dx	PPV (95% CI)
Autism Spectrum Disorder	At ≥ 1 year of age: ≥ 2 dates with Dx	299.xx except 299.1x	F84.x (except F84.2, F84.3)	0.94 (0.83 - 0.99)
Attention Deficit Hyperactivity Disorder	At ≥ 2 years of age, any of the following:<br>≥ 2 dates with Dx<br>≥ 2 dispensings of atomoxetine, clonidine, guanfacine, (dextro/lisdex)amphetamine, (dex)methylphenidate<br>≥ 1 Dx & ≥ 1 dispensing	314.xx	F90.x	0.88 (0.76 - 0.95)
Learning Difficulty	At ≥ 2 years of age: ≥ 1 Dx	315.0x-315.2x	F81.0, F81.2, F81.8x, R48.0	0.98 (0.89 - 1.00)
Speech or Language Disorder	At ≥ 1.5 years of age: ≥ 2 dates with Dx	315.3x except 315.34	F80.xx (except F80.4), H93.25	0.98 (0.89 - 1.00)
Intellectual disability	At ≥ 2 years of age: ≥ 2 dates with Dx	317, 318.x, 319	F70-F79	0.82 (0.69 - 0.91)
Developmental Coordination Disorder	≥ 2 dates with Dx (any age)	315.4x	F82	0.38 (0.25 - 0.53) (0.90 (0.82 - 0.98) if also counting coordination issues)
Behavioral Disorder	At ≥ 2 years of age: ≥ 2 dates with Dx	312.xx, 313.xx	F63.xx, F91.x, F93.8, F93.9, F94.x, F98.8, F98.9	0.92 (0.81 - 0.98)
Any Neurodevelopmental Disorder	Presence of any of the outcomes above			

Abbreviations: CI, confidence interval; Dx, diagnosis; ICD, International Classification of Disease; PPV, positive predictive value.

[1] Reference: Straub L, Bateman BT, Hernandez-Diaz S et al. Validity of claims-based algorithms to identify neurodevelopmental disorders in children. Pharmacoepidemiol Drug Saf. 2021 Dec;30(12):1635-1642. doi: 10.1002/pds.5369. Epub 2021 Oct 15. PMID: 34623720; PMCID: PMC8578450.

eTable 4. Full List of Covariates and Corresponding Assessment Periods Included in Propensity Score Models.

Category	Variable	Type	Assessment Period
Cohort Source	MAX/TAF vs. MarketScan indicator	Binary	N/A
Demographics	Maternal age	Categorical (<=19, 20-24, 25-29, 30-34, 35-39, >=40)	at delivery
	US region	Categorical (Midwest, Northeast, South, West, Unknown)	at delivery
	Year of delivery	Categorical (each year as separate category)	at delivery
Mental health/developmental conditions	Adjustment disorder	Binary	LMP-90 days to delivery-1 day
	Anxiety	Binary	LMP-90 days to delivery-1 day
	Bipolar disorder	Binary	LMP-90 days to delivery-1 day
	Depression	Binary	LMP-90 days to delivery-1 day
	Epilepsy	Binary	see algorithm; not included in model restricted to epilepsy-cohort
	Personality disorder	Binary	LMP-90 days to delivery-1 day
	Psychosis	Binary	LMP-90 days to delivery-1 day
	Schizophrenia	Binary	LMP-90 days to delivery-1 day
	Other mental health condition	Binary	LMP-90 days to delivery-1 day
	ADHD	Binary	LMP-90 days to delivery-1 day
	Behavioral disorder	Binary	LMP-90 days to delivery-1 day
	Other developmental disorder*	Binary	LMP-90 days to delivery-1 day
	Chronic fatigue	Binary	LMP-90 days to delivery-1 day
	Chronic pain	Binary	LMP-90 days to delivery-1 day
	Migraine/headache	Binary	LMP-90 days to delivery-1 day
	Neuromuscular disorder	Binary	LMP-90 days to delivery-1 day
	Neuropathic pain	Binary	LMP-90 days to delivery-1 day
	Sleep disorder	Binary	LMP-90 days to delivery-1 day
Healthcare Utilization/Mental Health Severity	N mental health diagnoses	Categorical (0, 1, 2-3, >=4)	LMP-90 days to end of first half of pregnancy
	N mental health-related ED visits	Categorical (0, 1, 2-3, >=4)	LMP-90 days to end of first half of pregnancy
	N mental health-related hospitalizations	Categorical (0, 1, 2-3, >=4)	LMP-90 days to end of first half of pregnancy
	N outpatient visits	Categorical (<=3, 4-6, 7-10, >=11)	LMP-90 days to end of first half of pregnancy
	N other psychiatric medications	Continuous	LMP-90 days to end of first half of pregnancy
	Obstetric comorbidity score[1]	Categorical (0, 1, 2, >=3)	LMP-90 days to delivery
Substance Use	Alcohol use disorder	Binary	LMP-90 days to delivery-1 day
	Tobacco use	Binary	LMP-90 days to delivery-1 day
	Substance use disorder	Binary	LMP-90 days to delivery-1 day
Other prescription medications	Antidepressants	Binary	LMP-90 days to end of first half of pregnancy
	Antipsychotics	Binary	LMP-90 days to end of first half of pregnancy
	Anxiolytics/hypnotics/sedatives	Binary	LMP-90 days to end of first half of pregnancy
	Barbiturates	Binary	LMP-90 days to end of first half of pregnancy
	Benzodiazepines	Binary	LMP-90 days to end of first half of pregnancy
	Opioids	Binary	LMP-90 days to end of first half of pregnancy

Category	Variable	Type	Assessment Period
	Psychostimulants	Binary	LMP-90 days to end of first half of pregnancy
	Antidiabetics	Binary	LMP-90 days to end of first half of pregnancy
	Antihypertensives	Binary	LMP-90 days to end of first half of pregnancy
	Corticosteroids	Binary	LMP-90 days to end of first half of pregnancy
	Medication assisted treatment (buprenorphine, methadone)	Binary	LMP-90 days to end of first half of pregnancy
	Lithium	Binary	LMP-90 days to end of first half of pregnancy
	NSAIDs	Binary	LMP-90 days to end of first half of pregnancy
	Prenatal folic acid supplementation	Binary	LMP-90 days to end of first half of pregnancy
	Progestins	Binary	LMP-90 days to end of first half of pregnancy
	Known teratogens	Binary	LMP-90 days to end of first half of pregnancy
	Suspected teratogens	Binary	LMP-90 days to end of first half of pregnancy
	Triptans	Binary	LMP-90 days to end of first half of pregnancy
Comorbidities	Asthma	Binary	LMP-90 days to delivery-1 day
	Autoimmune disorder	Binary	LMP-90 days to delivery-1 day
	Pregestational diabetes	Binary	LMP-90 days to LMP+90 days
	Hyperemesis gravidarum/nausea and vomiting in pregnancy	Binary	LMP-90 days to delivery-1 day
	Pregestational hypertension	Binary	LMP-90 days to delivery-1 day
	Influenza infection	Binary	LMP-90 days to delivery-1 day
	Other sexually transmitted disease	Binary	LMP-90 days to delivery-1 day
	Overweight/obesity	Binary	LMP-90 days to delivery-1 day
	Poor nutrition	Binary	LMP-90 days to delivery-1 day
	Renal disease	Binary	LMP-90 days to delivery-1 day
	TORCH infection	Binary	LMP-90 days to delivery-1 day
Other obstetric conditions	Adequacy of Prenatal Care Utilization[2]	Categorical (Inadequate, Intermediate, Adequate, Adequate Plus)	LMP to delivery-1
	Multiple gestation	Binary	LMP to delivery+60 days
	Time of prenatal care initiation	Categorical (separate categories for individual gestational months, no initiation)	LMP to delivery-1 day
	Time of first prenatal vitamin dispensing	Categorical (separate categories for individual months starting at 3 months before pregnancy, no dispensing)	LMP-3 months to delivery-1 day

Note: Further information on the statistical analysis plan, details on variable definitions, and analytic coding will be made available upon request.
Abbreviations: ADHD, attention deficit hyperactivity disorder; ED, emergency department; LMP, last menstrual period; MarketScan, MarketScan Research Database; MAX/TAF, Medicaid Analytic eXtract/Transformed Medicaid Statistical Information System Analytic Files; N, number; N/A, not applicable; NSAIDs, non-steroidal anti-inflammatory drug; TORCH, toxoplasmosis, others, rubella, cytomegalovirus, herpes simplex.
*Other developmental disorders include pervasive developmental disorders, speech or language disorders, reading disorders, mathematics disorders, learning difficulties, coordination disorders, intellectual disabilities.
[1] from Bateman BT, Mhyre JM, Hernandez-Diaz S, et al. Development of a comorbidity index for use in obstetric patients. Obstet Gynecol. 2013;122(5):957-965. doi:10.1097/AOG.0b013e3182a603bb

[2] adapted from Kotelchuck M. The Adequacy of Prenatal Care Utilization Index: its US distribution and association with low birthweight. Am J Public Health. 1994;84(9):1486-1489. doi:10.2105/ajph.84.9.1486

eTable 5. Patient Characteristics Among Publicly (MAX/TAF) and Commercially (MarketScan) Insured Women Exposed to the Specific Antiseizure Medication of Interest (Based on >=1 Dispensation in the Second Half of Pregnancy) Compared with Unexposed Pregnancies within the Epilepsy-Restricted Cohort.

	Unadjusted, N (column %)						PS Overlap-Weighted, N (column %)		
	MAX/TAF			MarketScan			Both Cohorts Combined		
**Variable**	**Exposed**	**Unexposed**	**SMD**	**Exposed**	**Unexposed**	**SMD**	**Exposed**	**Unexposed**	**SMD**
**Levetiracetam**									
N Total	3,728	7,245		1,533	1,642		2,770.5	2,770.5	
Age in Years: <=19	471 (12.6)	1,399 (19.3)	-0.18	3 (0.2)	18 (1.1)	-0.11	311.9 (11.3)	311.9 (11.3)	0.00
Age in Years: 20-24	1,145 (30.7)	2,612 (36.1)	-0.11	78 (5.1)	149 (9.1)	-0.16	742.8 (26.8)	742.8 (26.8)	0.00
Age in Years: 25-29	1,123 (30.1)	1,941 (26.8)	0.07	430 (28.1)	558 (34.0)	-0.13	823.1 (29.7)	823.1 (29.7)	0.00
Age in Years: 30-34	686 (18.4)	904 (12.5)	0.16	594 (38.8)	606 (36.9)	0.04	588.5 (21.2)	588.5 (21.2)	0.00
Age in Years: 35-39	251 (6.7)	318 (4.4)	0.10	342 (22.3)	250 (15.2)	0.18	246.2 (8.9)	246.2 (8.9)	0.00
Age in Years: >=40	52 (1.4)	71 (1.0)	0.04	86 (5.6)	61 (3.7)	0.09	58.0 (2.1)	58.0 (2.1)	0.00
Race/Ethnicity: Asian or other Pacific Islander	63 (1.7)	74 (1.0)	0.06	N/A	N/A	N/A	N/A	N/A	N/A
Race/Ethnicity: Black or African American	940 (25.2)	2,139 (29.5)	-0.10	N/A	N/A	N/A	N/A	N/A	N/A
Race/Ethnicity: Hispanic or Latino	603 (16.2)	963 (13.3)	0.08	N/A	N/A	N/A	N/A	N/A	N/A
Race/Ethnicity: Unknown or Other	230 (6.2)	340 (4.7)	0.07	N/A	N/A	N/A	N/A	N/A	N/A
Race/Ethnicity: White	1,892 (50.8)	3,729 (51.5)	-0.01	N/A	N/A	N/A	N/A	N/A	N/A
US Region: Midwest	1,361 (36.5)	2,598 (35.9)	0.01	374 (24.4)	402 (24.5)	0.00	931.5 (33.6)	931.5 (33.6)	0.00
US Region: Northeast	594 (15.9)	1,194 (16.5)	-0.01	249 (16.2)	270 (16.4)	-0.01	459.0 (16.6)	459.0 (16.6)	0.00
US Region: South	981 (26.3)	2,202 (30.4)	-0.09	653 (42.6)	692 (42.1)	0.01	872.8 (31.5)	872.8 (31.5)	0.00
US Region: Unknown	0	0	0.00	61 (4.0)	43 (2.6)	0.08	20.2 (0.7)	20.2 (0.7)	0.00
US Region: West	792 (21.2)	1,251 (17.3)	0.10	196 (12.8)	235 (14.3)	-0.04	487.1 (17.6)	487.1 (17.6)	0.00
Year of Delivery: 2000	*	<11	-0.02	0 (0.0)	0 (0.0)	0.00	0.0 (0.0)	0.0 (0.0)	0.00
Year of Delivery: 2001	<11	*	-0.19	0 (0.0)	0 (0.0)	0.00	3.9 (0.1)	3.9 (0.1)	0.00
Year of Delivery: 2002	14 (0.4)	227 (3.1)	-0.21	0 (0.0)	0 (0.0)	0.00	12.4 (0.5)	12.4 (0.5)	0.00
Year of Delivery: 2003	33 (0.9)	262 (3.6)	-0.18	0 (0.0)	0 (0.0)	0.00	27.9 (1.0)	27.9 (1.0)	0.00
Year of Delivery: 2004	48 (1.3)	368 (5.1)	-0.22	7 (0.5)	27 (1.6)	-0.12	46.4 (1.7)	46.4 (1.7)	0.00
Year of Delivery: 2005	51 (1.4)	329 (4.5)	-0.19	16 (1.0)	43 (2.6)	-0.12	51.8 (1.9)	51.8 (1.9)	0.00
Year of Delivery: 2006	90 (2.4)	347 (4.8)	-0.13	15 (1.0)	55 (3.4)	-0.16	78.3 (2.8)	78.3 (2.8)	0.00
Year of Delivery: 2007	99 (2.7)	306 (4.2)	-0.09	37 (2.4)	61 (3.7)	-0.08	89.3 (3.2)	89.3 (3.2)	0.00
Year of Delivery: 2008	126 (3.4)	330 (4.6)	-0.06	56 (3.7)	84 (5.1)	-0.07	117.3 (4.2)	117.3 (4.2)	0.00
Year of Delivery: 2009	169 (4.5)	370 (5.1)	-0.03	92 (6.0)	132 (8.0)	-0.08	157.1 (5.7)	157.1 (5.7)	0.00
Year of Delivery: 2010	225 (6.0)	484 (6.7)	-0.03	78 (5.1)	147 (9.0)	-0.15	185.1 (6.7)	185.1 (6.7)	0.00
Year of Delivery: 2011	286 (7.7)	520 (7.2)	0.02	112 (7.3)	147 (9.0)	-0.06	225.2 (8.1)	225.2 (8.1)	0.00
Year of Delivery: 2012	290 (7.8)	541 (7.5)	0.01	139 (9.1)	156 (9.5)	-0.01	246.1 (8.9)	229.4 (8.3)	-0.02
Year of Delivery: 2013	332 (8.9)	625 (8.6)	0.01	112 (7.3)	107 (6.5)	0.03	247.2 (8.9)	247.2 (8.9)	0.00
Year of Delivery: 2014	321 (8.6)	480 (6.6)	0.07	126 (8.2)	102 (6.2)	0.08	228.6 (8.3)	228.6 (8.3)	0.00
Year of Delivery: 2015	254 (6.8)	383 (5.3)	0.06	109 (7.1)	103 (6.3)	0.03	181.4 (6.6)	181.4 (6.6)	0.00
Year of Delivery: 2016	441 (11.8)	507 (7.0)	0.17	104 (6.8)	105 (6.4)	0.02	254.5 (9.2)	254.5 (9.2)	0.00
Year of Delivery: 2017	527 (14.1)	594 (8.2)	0.19	104 (6.8)	84 (5.1)	0.07	289.5 (10.5)	289.5 (10.5)	0.00
Year of Delivery: 2018	418 (11.2)	425 (5.9)	0.19	124 (8.1)	71 (4.3)	0.16	229.1 (8.3)	229.1 (8.3)	0.00
Year of Delivery: 2019	0	0	0.00	112 (7.3)	85 (5.2)	0.09	39.8 (1.4)	39.8 (1.4)	0.00
Year of Delivery: 2020	0	0	0.00	111 (7.2)	74 (4.5)	0.12	38.3 (1.4)	38.3 (1.4)	0.00
N MH Diagnoses: 0	2,252 (60.4)	4,225 (58.3)	0.04	1,250 (81.5)	1,224 (74.5)	0.17	1,776.4 (64.1)	1,776.4 (64.1)	0.00
N MH Diagnoses: 1	539 (14.5)	1,137 (15.7)	-0.03	132 (8.6)	173 (10.5)	-0.07	377.1 (13.6)	377.1 (13.6)	0.00

	Unadjusted, N (column %)						PS Overlap-Weighted, N (column %)		
	MAX/TAF			MarketScan			Both Cohorts Combined		
Variable	Exposed	Unexposed	SMD	Exposed	Unexposed	SMD	Exposed	Unexposed	SMD
N MH Diagnoses: 2-3	495 (13.3)	1,047 (14.5)	-0.03	88 (5.7)	144 (8.8)	-0.12	332.0 (12.0)	332.0 (12.0)	0.00
N MH Diagnoses: >=4	442 (11.9)	836 (11.5)	0.01	63 (4.1)	101 (6.2)	-0.09	285.1 (10.3)	285.1 (10.3)	0.00
N ED Visits: 0	3,244 (87.0)	6,204 (85.6)	0.04	1,485 (96.9)	1,552 (94.5)	0.12	2,449.7 (88.4)	2,449.7 (88.4)	0.00
N ED Visits: 1	332 (8.9)	704 (9.7)	-0.03	34 (2.2)	58 (3.5)	-0.08	220.5 (8.0)	220.5 (8.0)	0.00
N ED Visits: 2-3	118 (3.2)	268 (3.7)	-0.03	9 (0.6)	28 (1.7)	-0.11	79.3 (2.9)	79.3 (2.9)	0.00
N ED Visits: >=4	34 (0.9)	69 (1.0)	0.00	5 (0.3)	4 (0.2)	0.02	21.0 (0.8)	21.0 (0.8)	0.00
N MH Hospitalizations: 0	3,499 (93.9)	6,749 (93.2)	0.03	1,509 (98.4)	1,609 (98.0)	0.03	2,624.5 (94.7)	2,624.5 (94.7)	0.00
N MH Hospitalizations: 1	172 (4.6)	384 (5.3)	-0.03	19 (1.2)	26 (1.6)	-0.03	111.2 (4.0)	111.2 (4.0)	0.00
N MH Hospitalizations: 2-3	*	*	0.00	2 (0.1)	7 (0.4)	-0.06	28.3 (1.0)	28.3 (1.0)	0.00
N MH Hospitalizations: >=4	<11	*	-0.01	3 (0.2)	0 (0.0)	0.06	6.5 (0.2)	6.5 (0.2)	0.00
N Outpatient Visits: <=3	361 (9.7)	1,012 (14.0)	-0.13	64 (4.2)	99 (6.0)	-0.08	266.3 (9.6)	266.3 (9.6)	0.00
N Outpatient Visits: 4-6	611 (16.4)	1,299 (17.9)	-0.04	283 (18.5)	336 (20.5)	-0.05	492.1 (17.8)	492.1 (17.8)	0.00
N Outpatient Visits: 7-10	924 (24.8)	1,788 (24.7)	0.00	528 (34.4)	482 (29.4)	0.11	742.4 (26.8)	742.4 (26.8)	0.00
N Outpatient Visits: >=11	1,832 (49.1)	3,146 (43.4)	0.11	658 (42.9)	725 (44.2)	-0.02	1,269.8 (45.8)	1,269.8 (45.8)	0.00
N Psychotropic Medications (Except ASMs), Mean (SD)	0.0 (0.2)	0.0 (0.2)	0.02	0.0 (0.2)	0.0 (0.2)	-0.06	0.0 (0.2)	0.0 (0.2)	0.00
Alcohol Use Disorder	112 (3.0)	277 (3.8)	-0.05	9 (0.6)	21 (1.3)	-0.07	74.7 (2.7)	74.7 (2.7)	0.00
Smoking	759 (20.4)	1,475 (20.4)	0.00	61 (4.0)	96 (5.9)	-0.09	467.2 (16.9)	467.2 (16.9)	0.00
Substance Use Disorder	516 (13.8)	1,007 (13.9)	0.00	38 (2.5)	43 (2.6)	-0.01	311.2 (11.2)	311.2 (11.2)	0.00
ADHD	92 (2.5)	191 (2.6)	-0.01	20 (1.3)	40 (2.4)	-0.08	66.1 (2.4)	66.1 (2.4)	0.00
Adjustment Disorder	66 (1.8)	125 (1.7)	0.00	12 (0.8)	8 (0.5)	0.04	40.8 (1.5)	40.8 (1.5)	0.00
Anxiety	767 (20.6)	1,489 (20.6)	0.00	157 (10.2)	251 (15.3)	-0.15	521.5 (18.8)	521.5 (18.8)	0.00
Behavioral Disorder	57 (1.5)	131 (1.8)	-0.02	2 (0.1)	5 (0.3)	-0.04	35.3 (1.3)	35.3 (1.3)	0.00
Bipolar Disorder	333 (8.9)	610 (8.4)	0.02	23 (1.5)	43 (2.6)	-0.08	206.0 (7.4)	206.0 (7.4)	0.00
Chronic Pain	104 (2.8)	173 (2.4)	0.03	20 (1.3)	29 (1.8)	-0.04	66.7 (2.4)	66.7 (2.4)	0.00
Chronic Fatigue	366 (9.8)	695 (9.6)	0.01	142 (9.3)	210 (12.8)	-0.11	279.4 (10.1)	279.4 (10.1)	0.00
Depression	794 (21.3)	1,660 (22.9)	-0.04	170 (11.1)	234 (14.3)	-0.10	546.3 (19.7)	546.3 (19.7)	0.00
Migraine/Headache	577 (15.5)	991 (13.7)	0.05	202 (13.2)	316 (19.2)	-0.17	419.9 (15.2)	419.9 (15.2)	0.00
Neuromuscular Disorder	142 (3.8)	312 (4.3)	-0.03	31 (2.0)	73 (4.5)	-0.14	102.0 (3.7)	102.0 (3.7)	0.00
Neuropathic Pain	239 (6.4)	463 (6.4)	0.00	99 (6.5)	169 (10.3)	-0.14	188.9 (6.8)	188.9 (6.8)	0.00
Other MH Disorders	174 (4.7)	358 (4.9)	-0.01	30 (2.0)	50 (3.1)	-0.07	121.4 (4.4)	121.4 (4.4)	0.00
Other Developmental Disorders	49 (1.3)	82 (1.1)	0.02	2 (0.1)	4 (0.2)	-0.03	26.2 (1.0)	26.2 (1.0)	0.00
Personality Disorder	52 (1.4)	126 (1.7)	-0.03	5 (0.3)	7 (0.4)	-0.02	35.5 (1.3)	35.5 (1.3)	0.00
Psychosis	112 (3.0)	186 (2.6)	0.03	13 (0.9)	21 (1.3)	-0.04	69.5 (2.5)	69.5 (2.5)	0.00
Schizophrenia/Schizoaffective Disorder	57 (1.5)	90 (1.2)	0.02	1 (0.1)	6 (0.4)	-0.06	32.2 (1.2)	32.2 (1.2)	0.00
Sleep Disorder	174 (4.7)	282 (3.9)	0.04	84 (5.5)	98 (6.0)	-0.02	133.2 (4.8)	133.2 (4.8)	0.00
Asthma	522 (14.0)	1,144 (15.8)	-0.05	86 (5.6)	140 (8.5)	-0.11	366.1 (13.2)	366.1 (13.2)	0.00
Autoimmune Disorder	113 (3.0)	206 (2.8)	0.01	73 (4.8)	96 (5.9)	-0.05	101.1 (3.7)	101.1 (3.7)	0.00
Hyperemesis/NVP	1,180 (31.7)	2,529 (34.9)	-0.07	221 (14.4)	351 (21.4)	-0.18	826.8 (29.8)	826.8 (29.8)	0.00
Influenca Infection	82 (2.2)	180 (2.5)	-0.02	38 (2.5)	35 (2.1)	0.02	63.4 (2.3)	63.4 (2.3)	0.00
Obstetric Comorbidity Score: 0	1,330 (35.7)	2,642 (36.5)	-0.02	554 (36.1)	634 (38.6)	-0.05	992.2 (35.8)	992.2 (35.8)	0.00
Obstetric Comorbidity Score: 1	808 (21.7)	1,590 (22.0)	-0.01	404 (26.4)	369 (22.5)	0.09	628.3 (22.7)	628.3 (22.7)	0.00
Obstetric Comorbidity Score: 2	574 (15.4)	1,133 (15.6)	-0.01	251 (16.4)	265 (16.1)	0.01	439.6 (15.9)	439.6 (15.9)	0.00
Obstetric Comorbidity Score: >=3	1,016 (27.3)	1,880 (26.0)	0.03	324 (21.1)	374 (22.8)	-0.04	710.5 (25.7)	710.5 (25.7)	0.00
Overweight/Obesity	572 (15.3)	811 (11.2)	0.12	190 (12.4)	185 (11.3)	0.03	378.2 (13.7)	378.2 (13.7)	0.00
Pre-gestational Diabetes	114 (3.1)	241 (3.3)	-0.02	33 (2.2)	43 (2.6)	-0.03	84.6 (3.1)	84.6 (3.1)	0.00

	Unadjusted, N (column %)						PS Overlap-Weighted, N (column %)		
	MAX/TAF			MarketScan			Both Cohorts Combined		
Variable	Exposed	Unexposed	SMD	Exposed	Unexposed	SMD	Exposed	Unexposed	SMD
Pre-gestational Hypertension	409 (11.0)	835 (11.5)	-0.02	143 (9.3)	170 (10.4)	-0.03	306.9 (11.1)	306.9 (11.1)	0.00
Poor Nutrition	172 (4.6)	247 (3.4)	0.06	98 (6.4)	123 (7.5)	-0.04	132.7 (4.8)	132.7 (4.8)	0.00
Renal Disease	37 (1.0)	85 (1.2)	-0.02	9 (0.6)	20 (1.2)	-0.07	26.7 (1.0)	26.7 (1.0)	0.00
TORCH Infection	140 (3.8)	277 (3.8)	0.00	33 (2.2)	36 (2.2)	0.00	98.5 (3.6)	98.5 (3.6)	0.00
Other Sexually Transmitted Diseases	430 (11.5)	812 (11.2)	0.01	50 (3.3)	56 (3.4)	-0.01	267.8 (9.7)	267.8 (9.7)	0.00
Prenatal Folic Acid Supplementation	2,674 (71.7)	4,000 (55.2)	0.35	882 (57.5)	527 (32.1)	0.53	1,669.0 (60.2)	1,669.0 (60.2)	0.00
Antidepressants	877 (23.5)	1,452 (20.0)	0.08	219 (14.3)	277 (16.9)	-0.07	569.0 (20.5)	569.0 (20.5)	0.00
Antidiabetics	69 (1.9)	86 (1.2)	0.05	56 (3.7)	52 (3.2)	0.03	55.3 (2.0)	55.3 (2.0)	0.00
Antihypertensives	260 (7.0)	450 (6.2)	0.03	71 (4.6)	104 (6.3)	-0.07	175.5 (6.3)	175.5 (6.3)	0.00
Antipsychotics	269 (7.2)	430 (5.9)	0.05	26 (1.7)	43 (2.6)	-0.06	159.9 (5.8)	159.9 (5.8)	0.00
Anxiolytics/Hypnotics/Other Sedatives	401 (10.8)	742 (10.2)	0.02	81 (5.3)	87 (5.3)	0.00	261.8 (9.5)	261.8 (9.5)	0.00
Barbiturates	180 (4.8)	299 (4.1)	0.03	65 (4.2)	56 (3.4)	0.04	125.3 (4.5)	125.3 (4.5)	0.00
Benzodiazepines	537 (14.4)	760 (10.5)	0.12	196 (12.8)	160 (9.7)	0.10	339.3 (12.3)	339.3 (12.3)	0.00
Corticosteroids	682 (18.3)	1,221 (16.9)	0.04	259 (16.9)	273 (16.6)	0.01	490.2 (17.7)	490.2 (17.7)	0.00
Insulin	65 (1.7)	131 (1.8)	0.00	24 (1.6)	26 (1.6)	0.00	47.3 (1.7)	47.3 (1.7)	0.00
Lithium	16 (0.4)	29 (0.4)	0.00	2 (0.1)	2 (0.1)	0.00	10.5 (0.4)	10.5 (0.4)	0.00
NSAIDs	954 (25.6)	1,747 (24.1)	0.03	129 (8.4)	148 (9.0)	-0.02	592.9 (21.4)	592.9 (21.4)	0.00
Opioid Agonist Therapy	57 (1.5)	76 (1.1)	0.04	5 (0.3)	8 (0.5)	-0.03	31.9 (1.2)	31.9 (1.2)	0.00
Prescription Opioids	1,223 (32.8)	2,514 (34.7)	-0.04	223 (14.6)	296 (18.0)	-0.09	826.6 (29.8)	826.6 (29.8)	0.00
Progestins	237 (6.4)	459 (6.3)	0.00	250 (16.3)	231 (14.1)	0.06	239.7 (8.7)	239.7 (8.7)	0.00
Psychostimulants	68 (1.8)	131 (1.8)	0.00	28 (1.8)	44 (2.7)	-0.06	53.5 (1.9)	53.5 (1.9)	0.00
Teratogens, Known	26 (0.7)	34 (0.5)	0.03	12 (0.8)	8 (0.5)	0.04	16.8 (0.6)	16.8 (0.6)	0.00
Teratogens, Suspected	593 (15.9)	1,123 (15.5)	0.01	148 (9.7)	181 (11.0)	-0.04	404.2 (14.6)	404.2 (14.6)	0.00
Triptans	152 (4.1)	183 (2.5)	0.09	52 (3.4)	60 (3.7)	-0.01	95.5 (3.5)	95.5 (3.5)	0.00
Adequacy of Prenatal Care Utilization: Inadequate	1,323 (35.5)	2,867 (39.6)	-0.08	705 (46.0)	860 (52.4)	-0.13	1,095.6 (39.5)	1,095.6 (39.5)	0.00
Adequacy of Prenatal Care Utilization: Intermediate	592 (15.9)	1,100 (15.2)	0.02	348 (22.7)	358 (21.8)	0.02	478.1 (17.3)	478.1 (17.3)	0.00
Adequacy of Prenatal Care Utilization: Adequate	613 (16.4)	1,121 (15.5)	0.03	241 (15.7)	208 (12.7)	0.09	435.4 (15.7)	435.4 (15.7)	0.00
Adequacy of Prenatal Care Utilization: Adequate Plus	1,200 (32.2)	2,157 (29.8)	0.05	239 (15.6)	216 (13.2)	0.07	761.4 (27.5)	761.4 (27.5)	0.00
Multiple Gestation	78 (2.1)	165 (2.3)	-0.01	53 (3.5)	43 (2.6)	0.05	67.1 (2.4)	67.1 (2.4)	0.00
Gestational Month of Prenatal Care Initiation: 1	231 (6.2)	390 (5.4)	0.03	102 (6.7)	110 (6.7)	0.00	161.4 (5.8)	161.4 (5.8)	0.00
Gestational Month of Prenatal Care Initiation: 2	1,380 (37.0)	2,637 (36.4)	0.01	738 (48.1)	714 (43.5)	0.09	1,081.1 (39.0)	1,081.1 (39.0)	0.00
Gestational Month of Prenatal Care Initiation: 3	982 (26.3)	1,788 (24.7)	0.04	417 (27.2)	429 (26.1)	0.02	729.2 (26.3)	729.2 (26.3)	0.00
Gestational Month of Prenatal Care Initiation: 4	381 (10.2)	781 (10.8)	-0.02	87 (5.7)	100 (6.1)	-0.02	261.3 (9.4)	261.3 (9.4)	0.00
Gestational Month of Prenatal Care Initiation: 5	230 (6.2)	486 (6.7)	-0.02	33 (2.2)	63 (3.8)	-0.10	151.4 (5.5)	151.4 (5.5)	0.00
Gestational Month of Prenatal Care Initiation: 6	148 (4.0)	277 (3.8)	0.01	15 (1.0)	30 (1.8)	-0.07	91.3 (3.3)	91.3 (3.3)	0.00
Gestational Month of Prenatal Care Initiation: 7	107 (2.9)	197 (2.7)	0.01	33 (2.2)	30 (1.8)	0.02	73.4 (2.7)	73.4 (2.7)	0.00
Gestational Month of Prenatal Care Initiation: 8	59 (1.6)	142 (2.0)	-0.03	15 (1.0)	19 (1.2)	-0.02	43.7 (1.6)	43.7 (1.6)	0.00
Gestational Month of Prenatal Care Initiation: 9	64 (1.7)	134 (1.9)	-0.01	16 (1.0)	17 (1.0)	0.00	42.2 (1.5)	42.2 (1.5)	0.00
Gestational Month of Prenatal Care Initiation: None	146 (3.9)	413 (5.7)	-0.08	77 (5.0)	130 (7.9)	-0.12	135.6 (4.9)	135.6 (4.9)	0.00
Gestational Month of Prenatal Vitamin Initiation: 1	311 (8.3)	468 (6.5)	0.07	98 (6.4)	64 (3.9)	0.11	202.9 (7.3)	202.9 (7.3)	0.00
Gestational Month of Prenatal Vitamin Initiation: 2	1,000 (26.8)	1,711 (23.6)	0.07	211 (13.8)	188 (11.5)	0.07	657.1 (23.7)	657.1 (23.7)	0.00
Gestational Month of Prenatal Vitamin Initiation: 3	523 (14.0)	946 (13.1)	0.03	82 (5.4)	104 (6.3)	-0.04	340.6 (12.3)	340.6 (12.3)	0.00
Gestational Month of Prenatal Vitamin Initiation: 4	263 (7.1)	479 (6.6)	0.02	41 (2.7)	39 (2.4)	0.02	172.2 (6.2)	172.2 (6.2)	0.00
Gestational Month of Prenatal Vitamin Initiation: 5	167 (4.5)	286 (4.0)	0.03	19 (1.2)	25 (1.5)	-0.02	100.8 (3.6)	100.8 (3.6)	0.00
Gestational Month of Prenatal Vitamin Initiation: 6	108 (2.9)	197 (2.7)	0.01	19 (1.2)	14 (0.9)	0.04	70.1 (2.5)	70.1 (2.5)	0.00

	Unadjusted, N (column %)						PS Overlap-Weighted, N (column %)		
	MAX/TAF			MarketScan			Both Cohorts Combined		
**Variable**	**Exposed**	**Unexposed**	**SMD**	**Exposed**	**Unexposed**	**SMD**	**Exposed**	**Unexposed**	**SMD**
Gestational Month of Prenatal Vitamin Initiation: 7	69 (1.9)	166 (2.3)	-0.03	16 (1.0)	25 (1.5)	-0.04	54.3 (2.0)	54.3 (2.0)	0.00
Gestational Month of Prenatal Vitamin Initiation: 8	50 (1.3)	98 (1.4)	0.00	13 (0.9)	14 (0.9)	0.00	35.4 (1.3)	35.4 (1.3)	0.00
Gestational Month of Prenatal Vitamin Initiation: 9	58 (1.6)	224 (3.1)	-0.10	20 (1.3)	31 (1.9)	-0.05	55.0 (2.0)	55.0 (2.0)	0.00
Gestational Month of Prenatal Vitamin Initiation: -1	85 (2.3)	60 (0.8)	0.12	79 (5.2)	16 (1.0)	0.24	42.7 (1.5)	42.7 (1.5)	0.00
Gestational Month of Prenatal Vitamin Initiation: -2	143 (3.8)	113 (1.6)	0.14	107 (7.0)	34 (2.1)	0.24	80.0 (2.9)	80.0 (2.9)	0.00
Gestational Month of Prenatal Vitamin Initiation: -3	301 (8.1)	163 (2.3)	0.27	257 (16.8)	74 (4.5)	0.41	147.1 (5.3)	147.1 (5.3)	0.00
Gestational Month of Prenatal Vitamin Initiation: None	650 (17.4)	2,334 (32.2)	-0.35	571 (37.3)	1,014 (61.8)	-0.51	812.3 (29.3)	812.3 (29.3)	0.00
**Lamotrigine**									
N Total	2,323	7,245		1,976	1,642		2,275.3	2,275.3	
Age in Years: <=19	302 (13.0)	1,399 (19.3)	-0.17	2 (0.1)	18 (1.1)	-0.13	228.9 (10.1)	228.9 (10.1)	0.00
Age in Years: 20-24	741 (31.9)	2,612 (36.1)	-0.09	59 (3.0)	149 (9.1)	-0.26	550.2 (24.2)	550.2 (24.2)	0.00
Age in Years: 25-29	696 (30.0)	1,941 (26.8)	0.07	523 (26.5)	558 (34.0)	-0.16	678.5 (29.8)	678.5 (29.8)	0.00
Age in Years: 30-34	395 (17.0)	904 (12.5)	0.13	851 (43.1)	606 (36.9)	0.13	529.4 (23.3)	529.4 (23.3)	0.00
Age in Years: 35-39	158 (6.8)	318 (4.4)	0.11	456 (23.1)	250 (15.2)	0.20	236.1 (10.4)	236.1 (10.4)	0.00
Age in Years: >=40	31 (1.3)	71 (1.0)	0.03	85 (4.3)	61 (3.7)	0.03	52.2 (2.3)	52.2 (2.3)	0.00
Race/Ethnicity: Asian or other Pacific Islander	43 (1.9)	74 (1.0)	0.07	N/A	N/A	N/A	N/A	N/A	N/A
Race/Ethnicity: Black or African American	407 (17.5)	2,139 (29.5)	-0.29	N/A	N/A	N/A	N/A	N/A	N/A
Race/Ethnicity: Hispanic or Latino	302 (13.0)	963 (13.3)	-0.01	N/A	N/A	N/A	N/A	N/A	N/A
Race/Ethnicity: Unknown or Other	123 (5.3)	340 (4.7)	0.03	N/A	N/A	N/A	N/A	N/A	N/A
Race/Ethnicity: White	1,448 (62.3)	3,729 (51.5)	0.22	N/A	N/A	N/A	N/A	N/A	N/A
US Region: Midwest	924 (39.8)	2,598 (35.9)	0.08	510 (25.8)	402 (24.5)	0.03	780.6 (34.3)	780.6 (34.3)	0.00
US Region: Northeast	448 (19.3)	1,194 (16.5)	0.07	344 (17.4)	270 (16.4)	0.03	401.7 (17.7)	401.7 (17.7)	0.00
US Region: South	556 (23.9)	2,202 (30.4)	-0.15	728 (36.8)	692 (42.1)	-0.11	687.5 (30.2)	687.5 (30.2)	0.00
US Region: Unknown	0	0	0.00	67 (3.4)	43 (2.6)	0.05	20.1 (0.9)	20.1 (0.9)	0.00
US Region: West	395 (17.0)	1,251 (17.3)	-0.01	327 (16.6)	235 (14.3)	0.06	385.4 (16.9)	385.4 (16.9)	0.00
Year of Delivery: 2000	*	<11	-0.02	0 (0.0)	0 (0.0)	0.00	0.0 (0.0)	0.0 (0.0)	0.00
Year of Delivery: 2001	*	*	-0.09	0 (0.0)	0 (0.0)	0.00	17.1 (0.8)	17.1 (0.8)	0.00
Year of Delivery: 2002	27 (1.2)	227 (3.1)	-0.14	0 (0.0)	0 (0.0)	0.00	22.8 (1.0)	22.8 (1.0)	0.00
Year of Delivery: 2003	34 (1.5)	262 (3.6)	-0.14	0 (0.0)	0 (0.0)	0.00	28.2 (1.2)	28.2 (1.2)	0.00
Year of Delivery: 2004	91 (3.9)	368 (5.1)	-0.06	20 (1.0)	27 (1.6)	-0.06	76.2 (3.4)	76.2 (3.4)	0.00
Year of Delivery: 2005	104 (4.5)	329 (4.5)	0.00	45 (2.3)	43 (2.6)	-0.02	87.9 (3.9)	87.9 (3.9)	0.00
Year of Delivery: 2006	117 (5.0)	347 (4.8)	0.01	57 (2.9)	55 (3.4)	-0.03	102.4 (4.5)	102.4 (4.5)	0.00
Year of Delivery: 2007	111 (4.8)	306 (4.2)	0.03	68 (3.4)	61 (3.7)	-0.01	96.8 (4.3)	96.8 (4.3)	0.00
Year of Delivery: 2008	116 (5.0)	330 (4.6)	0.02	114 (5.8)	84 (5.1)	0.03	120.8 (5.3)	120.8 (5.3)	0.00
Year of Delivery: 2009	129 (5.6)	370 (5.1)	0.02	156 (7.9)	132 (8.0)	-0.01	145.6 (6.4)	145.6 (6.4)	0.00
Year of Delivery: 2010	144 (6.2)	484 (6.7)	-0.02	143 (7.2)	147 (9.0)	-0.06	157.7 (6.9)	157.7 (6.9)	0.00
Year of Delivery: 2011	175 (7.5)	520 (7.2)	0.01	167 (8.5)	147 (9.0)	-0.02	179.9 (7.9)	179.9 (7.9)	0.00
Year of Delivery: 2012	192 (8.3)	541 (7.5)	0.03	185 (9.4)	156 (9.5)	0.00	198.7 (8.7)	190.0 (8.4)	-0.01
Year of Delivery: 2013	167 (7.2)	625 (8.6)	-0.05	149 (7.5)	107 (6.5)	0.04	177.9 (7.8)	177.9 (7.8)	0.00
Year of Delivery: 2014	164 (7.1)	480 (6.6)	0.02	144 (7.3)	102 (6.2)	0.04	160.9 (7.1)	160.9 (7.1)	0.00
Year of Delivery: 2015	119 (5.1)	383 (5.3)	-0.01	123 (6.2)	103 (6.3)	0.00	124.1 (5.5)	124.1 (5.5)	0.00
Year of Delivery: 2016	212 (9.1)	507 (7.0)	0.08	115 (5.8)	105 (6.4)	-0.02	169.4 (7.4)	169.4 (7.4)	0.00
Year of Delivery: 2017	226 (9.7)	594 (8.2)	0.05	97 (4.9)	84 (5.1)	-0.01	174.6 (7.7)	174.6 (7.7)	0.00
Year of Delivery: 2018	173 (7.5)	425 (5.9)	0.06	122 (6.2)	71 (4.3)	0.08	141.8 (6.2)	141.8 (6.2)	0.00
Year of Delivery: 2019	0	0	0.00	106 (5.4)	85 (5.2)	0.01	35.7 (1.6)	35.7 (1.6)	0.00

	Unadjusted, N (column %)						PS Overlap-Weighted, N (column %)		
	MAX/TAF			MarketScan			Both Cohorts Combined		
**Variable**	**Exposed**	**Unexposed**	**SMD**	**Exposed**	**Unexposed**	**SMD**	**Exposed**	**Unexposed**	**SMD**
Year of Delivery: 2020	0	0	0.00	90 (4.6)	74 (4.5)	0.00	33.2 (1.5)	33.2 (1.5)	0.00
N MH Diagnoses: 0	1,372 (59.1)	4,225 (58.3)	0.02	1,649 (83.5)	1,224 (74.5)	0.22	1,502.8 (66.1)	1,502.8 (66.1)	0.00
N MH Diagnoses: 1	303 (13.0)	1,137 (15.7)	-0.08	145 (7.3)	173 (10.5)	-0.11	274.1 (12.1)	274.1 (12.1)	0.00
N MH Diagnoses: 2-3	316 (13.6)	1,047 (14.5)	-0.02	106 (5.4)	144 (8.8)	-0.13	259.1 (11.4)	259.1 (11.4)	0.00
N MH Diagnoses: >=4	332 (14.3)	836 (11.5)	0.08	76 (3.9)	101 (6.2)	-0.11	239.3 (10.5)	239.3 (10.5)	0.00
N ED Visits: 0	2,053 (88.4)	6,204 (85.6)	0.08	1,948 (98.6)	1,552 (94.5)	0.22	2,067.7 (90.9)	2,067.7 (90.9)	0.00
N ED Visits: 1	181 (7.8)	704 (9.7)	-0.07	17 (0.9)	58 (3.5)	-0.18	139.4 (6.1)	139.4 (6.1)	0.00
N ED Visits: 2-3	*	*	-0.01	9 (0.5)	28 (1.7)	-0.12	60.3 (2.7)	60.3 (2.7)	0.00
N ED Visits: >=4	<11	*	-0.08	2 (0.1)	4 (0.2)	-0.03	7.9 (0.4)	7.9 (0.4)	0.00
N MH Hospitalizations: 0	2,202 (94.8)	6,749 (93.2)	0.07	1,958 (99.1)	1,609 (98.0)	0.09	2,179.9 (95.8)	2,179.9 (95.8)	0.00
N MH Hospitalizations: 1	95 (4.1)	384 (5.3)	-0.06	11 (0.6)	26 (1.6)	-0.10	73.4 (3.2)	73.4 (3.2)	0.00
N MH Hospitalizations: 2-3	*	*	-0.02	6 (0.3)	7 (0.4)	-0.02	19.3 (0.9)	19.3 (0.9)	0.00
N MH Hospitalizations: >=4	<11	*	-0.05	1 (0.1)	0 (0.0)	0.03	2.7 (0.1)	2.7 (0.1)	0.00
N Outpatient Visits: <=3	171 (7.4)	1,012 (14.0)	-0.22	71 (3.6)	99 (6.0)	-0.11	171.4 (7.5)	171.4 (7.5)	0.00
N Outpatient Visits: 4-6	369 (15.9)	1,299 (17.9)	-0.05	370 (18.7)	336 (20.5)	-0.04	409.3 (18.0)	409.3 (18.0)	0.00
N Outpatient Visits: 7-10	588 (25.3)	1,788 (24.7)	0.01	650 (32.9)	482 (29.4)	0.08	630.5 (27.7)	630.5 (27.7)	0.00
N Outpatient Visits: >=11	1,195 (51.4)	3,146 (43.4)	0.16	885 (44.8)	725 (44.2)	0.01	1,064.1 (46.8)	1,064.1 (46.8)	0.00
N Psychotropic Medications (Except ASMs), Mean (SD)	0.0 (0.2)	0.0 (0.2)	0.05	0.0 (0.2)	0.0 (0.2)	-0.07	0.0 (0.2)	0.0 (0.2)	0.00
Alcohol Use Disorder	55 (2.4)	277 (3.8)	-0.08	6 (0.3)	21 (1.3)	-0.11	45.4 (2.0)	45.4 (2.0)	0.00
Smoking	433 (18.6)	1,475 (20.4)	-0.04	40 (2.0)	96 (5.9)	-0.20	319.2 (14.0)	319.2 (14.0)	0.00
Substance Use Disorder	259 (11.2)	1,007 (13.9)	-0.08	46 (2.3)	43 (2.6)	-0.02	202.8 (8.9)	202.8 (8.9)	0.00
ADHD	77 (3.3)	191 (2.6)	0.04	27 (1.4)	40 (2.4)	-0.08	59.6 (2.6)	59.6 (2.6)	0.00
Adjustment Disorder	48 (2.1)	125 (1.7)	0.02	12 (0.6)	8 (0.5)	0.02	31.9 (1.4)	31.9 (1.4)	0.00
Anxiety	470 (20.2)	1,489 (20.6)	-0.01	196 (9.9)	251 (15.3)	-0.16	394.7 (17.4)	394.7 (17.4)	0.00
Behavioral Disorder	41 (1.8)	131 (1.8)	0.00	4 (0.2)	5 (0.3)	-0.02	28.6 (1.3)	28.6 (1.3)	0.00
Bipolar Disorder	322 (13.9)	610 (8.4)	0.17	44 (2.2)	43 (2.6)	-0.03	203.7 (9.0)	203.7 (9.0)	0.00
Chronic Pain	65 (2.8)	173 (2.4)	0.03	21 (1.1)	29 (1.8)	-0.06	52.1 (2.3)	52.1 (2.3)	0.00
Chronic Fatigue	213 (9.2)	695 (9.6)	-0.01	156 (7.9)	210 (12.8)	-0.16	218.1 (9.6)	218.1 (9.6)	0.00
Depression	551 (23.7)	1,660 (22.9)	0.02	175 (8.9)	234 (14.3)	-0.17	444.4 (19.5)	444.4 (19.5)	0.00
Migraine/Headache	349 (15.0)	991 (13.7)	0.04	212 (10.7)	316 (19.2)	-0.24	327.7 (14.4)	327.7 (14.4)	0.00
Neuromuscular Disorder	71 (3.1)	312 (4.3)	-0.07	27 (1.4)	73 (4.5)	-0.18	67.8 (3.0)	67.8 (3.0)	0.00
Neuropathic Pain	145 (6.2)	463 (6.4)	-0.01	102 (5.2)	169 (10.3)	-0.19	148.3 (6.5)	148.3 (6.5)	0.00
Other MH Disorders	90 (3.9)	358 (4.9)	-0.05	40 (2.0)	50 (3.1)	-0.06	82.7 (3.6)	82.7 (3.6)	0.00
Other Developmental Disorders	31 (1.3)	82 (1.1)	0.02	4 (0.2)	4 (0.2)	-0.01	19.6 (0.9)	19.6 (0.9)	0.00
Personality Disorder	51 (2.2)	126 (1.7)	0.03	5 (0.3)	7 (0.4)	-0.03	33.6 (1.5)	33.6 (1.5)	0.00
Psychosis	63 (2.7)	186 (2.6)	0.01	9 (0.5)	21 (1.3)	-0.09	47.0 (2.1)	47.0 (2.1)	0.00
Schizophrenia/Schizoaffective Disorder	24 (1.0)	90 (1.2)	-0.02	4 (0.2)	6 (0.4)	-0.03	17.9 (0.8)	17.9 (0.8)	0.00
Sleep Disorder	115 (5.0)	282 (3.9)	0.05	91 (4.6)	98 (6.0)	-0.06	113.8 (5.0)	113.8 (5.0)	0.00
Asthma	259 (11.2)	1,144 (15.8)	-0.14	108 (5.5)	140 (8.5)	-0.12	241.3 (10.6)	241.3 (10.6)	0.00
Autoimmune Disorder	66 (2.8)	206 (2.8)	0.00	72 (3.6)	96 (5.9)	-0.10	77.4 (3.4)	77.4 (3.4)	0.00
Hyperemesis/NVP	695 (29.9)	2,529 (34.9)	-0.11	226 (11.4)	351 (21.4)	-0.27	597.3 (26.3)	597.3 (26.3)	0.00
Influenca Infection	40 (1.7)	180 (2.5)	-0.05	39 (2.0)	35 (2.1)	-0.01	44.3 (1.9)	44.3 (1.9)	0.00
Obstetric Comorbidity Score: 0	961 (41.4)	2,642 (36.5)	0.10	734 (37.2)	634 (38.6)	-0.03	886.8 (39.0)	886.8 (39.0)	0.00
Obstetric Comorbidity Score: 1	505 (21.7)	1,590 (22.0)	-0.01	512 (25.9)	369 (22.5)	0.08	513.9 (22.6)	513.9 (22.6)	0.00
Obstetric Comorbidity Score: 2	315 (13.6)	1,133 (15.6)	-0.06	328 (16.6)	265 (16.1)	0.01	340.2 (15.0)	340.2 (15.0)	0.00

	Unadjusted, N (column %)						PS Overlap-Weighted, N (column %)		
	MAX/TAF			MarketScan			Both Cohorts Combined		
Variable	Exposed	Unexposed	SMD	Exposed	Unexposed	SMD	Exposed	Unexposed	SMD
Obstetric Comorbidity Score: >=3	542 (23.3)	1,880 (26.0)	-0.06	402 (20.3)	374 (22.8)	-0.06	534.5 (23.5)	534.5 (23.5)	0.00
Overweight/Obesity	294 (12.7)	811 (11.2)	0.05	195 (9.9)	185 (11.3)	-0.05	267.6 (11.8)	267.6 (11.8)	0.00
Pre-gestational Diabetes	52 (2.2)	241 (3.3)	-0.07	32 (1.6)	43 (2.6)	-0.07	57.7 (2.5)	57.7 (2.5)	0.00
Pre-gestational Hypertension	214 (9.2)	835 (11.5)	-0.08	180 (9.1)	170 (10.4)	-0.04	226.4 (10.0)	226.4 (10.0)	0.00
Poor Nutrition	84 (3.6)	247 (3.4)	0.01	105 (5.3)	123 (7.5)	-0.09	95.6 (4.2)	95.6 (4.2)	0.00
Renal Disease	26 (1.1)	85 (1.2)	-0.01	12 (0.6)	20 (1.2)	-0.06	22.7 (1.0)	22.7 (1.0)	0.00
TORCH Infection	72 (3.1)	277 (3.8)	-0.04	46 (2.3)	36 (2.2)	0.01	65.3 (2.9)	65.3 (2.9)	0.00
Other Sexually Transmitted Diseases	195 (8.4)	812 (11.2)	-0.09	43 (2.2)	56 (3.4)	-0.07	161.0 (7.1)	161.0 (7.1)	0.00
Prenatal Folic Acid Supplementation	1,692 (72.8)	4,000 (55.2)	0.37	1,224 (61.9)	527 (32.1)	0.63	1,362.9 (59.9)	1,362.9 (59.9)	0.00
Antidepressants	615 (26.5)	1,452 (20.0)	0.15	250 (12.7)	277 (16.9)	-0.12	483.2 (21.2)	483.2 (21.2)	0.00
Antidiabetics	29 (1.3)	86 (1.2)	0.01	47 (2.4)	52 (3.2)	-0.05	39.1 (1.7)	39.1 (1.7)	0.00
Antihypertensives	148 (6.4)	450 (6.2)	0.01	87 (4.4)	104 (6.3)	-0.09	137.9 (6.1)	137.9 (6.1)	0.00
Antipsychotics	200 (8.6)	430 (5.9)	0.10	35 (1.8)	43 (2.6)	-0.06	137.7 (6.1)	137.7 (6.1)	0.00
Anxiolytics/Hypnotics/Other Sedatives	249 (10.7)	742 (10.2)	0.02	103 (5.2)	87 (5.3)	0.00	197.7 (8.7)	197.7 (8.7)	0.00
Barbiturates	121 (5.2)	299 (4.1)	0.05	84 (4.3)	56 (3.4)	0.04	99.2 (4.4)	99.2 (4.4)	0.00
Benzodiazepines	363 (15.6)	760 (10.5)	0.15	236 (11.9)	160 (9.7)	0.07	286.4 (12.6)	286.4 (12.6)	0.00
Corticosteroids	459 (19.8)	1,221 (16.9)	0.08	332 (16.8)	273 (16.6)	0.00	405.4 (17.8)	405.4 (17.8)	0.00
Insulin	23 (1.0)	131 (1.8)	-0.07	21 (1.1)	26 (1.6)	-0.05	29.0 (1.3)	29.0 (1.3)	0.00
Lithium	15 (0.7)	29 (0.4)	0.03	6 (0.3)	2 (0.1)	0.04	11.0 (0.5)	11.0 (0.5)	0.00
NSAIDs	505 (21.7)	1,747 (24.1)	-0.06	155 (7.8)	148 (9.0)	-0.04	414.3 (18.2)	414.3 (18.2)	0.00
Opioid Agonist Therapy	29 (1.3)	76 (1.1)	0.02	4 (0.2)	8 (0.5)	-0.05	20.6 (0.9)	20.6 (0.9)	0.00
Prescription Opioids	764 (32.9)	2,514 (34.7)	-0.04	282 (14.3)	296 (18.0)	-0.10	638.5 (28.1)	638.5 (28.1)	0.00
Progestins	181 (7.8)	459 (6.3)	0.06	304 (15.4)	231 (14.1)	0.04	221.1 (9.7)	221.1 (9.7)	0.00
Psychostimulants	54 (2.3)	131 (1.8)	0.04	34 (1.7)	44 (2.7)	-0.07	47.4 (2.1)	47.4 (2.1)	0.00
Teratogens, Known	<11	*	-0.04	11 (0.6)	8 (0.5)	0.01	8.3 (0.4)	8.3 (0.4)	0.00
Teratogens, Suspected	336 (14.5)	1,123 (15.5)	-0.03	170 (8.6)	181 (11.0)	-0.08	298.0 (13.1)	298.0 (13.1)	0.00
Triptans	99 (4.3)	183 (2.5)	0.10	78 (4.0)	60 (3.7)	0.02	83.4 (3.7)	83.4 (3.7)	0.00
Adequacy of Prenatal Care Utilization: Inadequate	872 (37.5)	2,867 (39.6)	-0.04	1,023 (51.8)	860 (52.4)	-0.01	967.1 (42.5)	967.1 (42.5)	0.00
Adequacy of Prenatal Care Utilization: Intermediate	360 (15.5)	1,100 (15.2)	0.01	422 (21.4)	358 (21.8)	-0.01	395.7 (17.4)	395.7 (17.4)	0.00
Adequacy of Prenatal Care Utilization: Adequate	363 (15.6)	1,121 (15.5)	0.00	251 (12.7)	208 (12.7)	0.00	332.8 (14.6)	332.8 (14.6)	0.00
Adequacy of Prenatal Care Utilization: Adequate Plus	728 (31.3)	2,157 (29.8)	0.03	280 (14.2)	216 (13.2)	0.03	579.6 (25.5)	579.6 (25.5)	0.00
Multiple Gestation	55 (2.4)	165 (2.3)	0.01	78 (4.0)	43 (2.6)	0.07	60.5 (2.7)	60.5 (2.7)	0.00
Gestational Month of Prenatal Care Initiation: 1	134 (5.8)	390 (5.4)	0.02	149 (7.5)	110 (6.7)	0.03	137.3 (6.0)	137.3 (6.0)	0.00
Gestational Month of Prenatal Care Initiation: 2	885 (38.1)	2,637 (36.4)	0.04	905 (45.8)	714 (43.5)	0.05	904.6 (39.8)	904.6 (39.8)	0.00
Gestational Month of Prenatal Care Initiation: 3	627 (27.0)	1,788 (24.7)	0.05	530 (26.8)	429 (26.1)	0.02	603.1 (26.5)	603.1 (26.5)	0.00
Gestational Month of Prenatal Care Initiation: 4	236 (10.2)	781 (10.8)	-0.02	106 (5.4)	100 (6.1)	-0.03	204.5 (9.0)	204.5 (9.0)	0.00
Gestational Month of Prenatal Care Initiation: 5	121 (5.2)	486 (6.7)	-0.06	49 (2.5)	63 (3.8)	-0.08	108.8 (4.8)	108.8 (4.8)	0.00
Gestational Month of Prenatal Care Initiation: 6	70 (3.0)	277 (3.8)	-0.04	21 (1.1)	30 (1.8)	-0.06	60.6 (2.7)	60.6 (2.7)	0.00
Gestational Month of Prenatal Care Initiation: 7	61 (2.6)	197 (2.7)	-0.01	39 (2.0)	30 (1.8)	0.01	55.0 (2.4)	55.0 (2.4)	0.00
Gestational Month of Prenatal Care Initiation: 8	38 (1.6)	142 (2.0)	-0.02	17 (0.9)	19 (1.2)	-0.03	34.4 (1.5)	34.4 (1.5)	0.00
Gestational Month of Prenatal Care Initiation: 9	43 (1.9)	134 (1.9)	0.00	17 (0.9)	17 (1.0)	-0.02	34.4 (1.5)	34.4 (1.5)	0.00
Gestational Month of Prenatal Care Initiation: None	108 (4.7)	413 (5.7)	-0.05	143 (7.2)	130 (7.9)	-0.03	132.7 (5.8)	132.7 (5.8)	0.00
Gestational Month of Prenatal Vitamin Initiation: 1	179 (7.7)	468 (6.5)	0.05	126 (6.4)	64 (3.9)	0.11	162.8 (7.2)	162.8 (7.2)	0.00
Gestational Month of Prenatal Vitamin Initiation: 2	613 (26.4)	1,711 (23.6)	0.06	239 (12.1)	188 (11.5)	0.02	519.3 (22.8)	519.3 (22.8)	0.00
Gestational Month of Prenatal Vitamin Initiation: 3	313 (13.5)	946 (13.1)	0.01	119 (6.0)	104 (6.3)	-0.01	272.0 (12.0)	272.0 (12.0)	0.00

	Unadjusted, N (column %)						PS Overlap-Weighted, N (column %)		
	MAX/TAF			MarketScan			Both Cohorts Combined		
**Variable**	**Exposed**	**Unexposed**	**SMD**	**Exposed**	**Unexposed**	**SMD**	**Exposed**	**Unexposed**	**SMD**
Gestational Month of Prenatal Vitamin Initiation: 4	144 (6.2)	479 (6.6)	-0.02	44 (2.2)	39 (2.4)	-0.01	123.0 (5.4)	123.0 (5.4)	0.00
Gestational Month of Prenatal Vitamin Initiation: 5	84 (3.6)	286 (4.0)	-0.02	22 (1.1)	25 (1.5)	-0.04	70.9 (3.1)	70.9 (3.1)	0.00
Gestational Month of Prenatal Vitamin Initiation: 6	57 (2.5)	197 (2.7)	-0.02	13 (0.7)	14 (0.9)	-0.02	47.1 (2.1)	47.1 (2.1)	0.00
Gestational Month of Prenatal Vitamin Initiation: 7	43 (1.9)	166 (2.3)	-0.03	13 (0.7)	25 (1.5)	-0.08	40.1 (1.8)	40.1 (1.8)	0.00
Gestational Month of Prenatal Vitamin Initiation: 8	29 (1.3)	98 (1.4)	-0.01	7 (0.4)	14 (0.9)	-0.06	25.2 (1.1)	25.2 (1.1)	0.00
Gestational Month of Prenatal Vitamin Initiation: 9	34 (1.5)	224 (3.1)	-0.11	20 (1.0)	31 (1.9)	-0.07	40.5 (1.8)	40.5 (1.8)	0.00
Gestational Month of Prenatal Vitamin Initiation: -1	72 (3.1)	60 (0.8)	0.16	126 (6.4)	16 (1.0)	0.29	43.5 (1.9)	43.5 (1.9)	0.00
Gestational Month of Prenatal Vitamin Initiation: -2	97 (4.2)	113 (1.6)	0.16	174 (8.8)	34 (2.1)	0.30	75.9 (3.3)	75.9 (3.3)	0.00
Gestational Month of Prenatal Vitamin Initiation: -3	251 (10.8)	163 (2.3)	0.35	391 (19.8)	74 (4.5)	0.48	148.1 (6.5)	148.1 (6.5)	0.00
Gestational Month of Prenatal Vitamin Initiation: None	407 (17.5)	2,334 (32.2)	-0.34	682 (34.5)	1,014 (61.8)	-0.57	707.0 (31.1)	707.0 (31.1)	0.00
**Carbamazepine**									
N Total	1,406	7,245		518	1,642		1,273.8	1,273.8	
Age in Years: <=19	172 (12.2)	1,399 (19.3)	-0.20	0 (0.0)	18 (1.1)	-0.15	132.9 (10.4)	132.9 (10.4)	0.00
Age in Years: 20-24	461 (32.8)	2,612 (36.1)	-0.07	23 (4.4)	149 (9.1)	-0.19	344.8 (27.1)	344.8 (27.1)	0.00
Age in Years: 25-29	409 (29.1)	1,941 (26.8)	0.05	119 (23.0)	558 (34.0)	-0.25	350.7 (27.5)	350.7 (27.5)	0.00
Age in Years: 30-34	222 (15.8)	904 (12.5)	0.10	210 (40.5)	606 (36.9)	0.07	272.1 (21.4)	272.1 (21.4)	0.00
Age in Years: 35-39	120 (8.5)	318 (4.4)	0.17	140 (27.0)	250 (15.2)	0.29	143.6 (11.3)	143.6 (11.3)	0.00
Age in Years: >=40	22 (1.6)	71 (1.0)	0.05	26 (5.0)	61 (3.7)	0.06	29.7 (2.3)	29.7 (2.3)	0.00
Race/Ethnicity: Asian or other Pacific Islander	29 (2.1)	74 (1.0)	0.08	N/A	N/A	N/A	N/A	N/A	N/A
Race/Ethnicity: Black or African American	333 (23.7)	2,139 (29.5)	-0.13	N/A	N/A	N/A	N/A	N/A	N/A
Race/Ethnicity: Hispanic or Latino	242 (17.2)	963 (13.3)	0.11	N/A	N/A	N/A	N/A	N/A	N/A
Race/Ethnicity: Unknown or Other	59 (4.2)	340 (4.7)	-0.02	N/A	N/A	N/A	N/A	N/A	N/A
Race/Ethnicity: White	743 (52.8)	3,729 (51.5)	0.03	N/A	N/A	N/A	N/A	N/A	N/A
US Region: Midwest	515 (36.6)	2,598 (35.9)	0.02	168 (32.4)	402 (24.5)	0.18	437.5 (34.4)	437.5 (34.4)	0.00
US Region: Northeast	231 (16.4)	1,194 (16.5)	0.00	72 (13.9)	270 (16.4)	-0.07	205.3 (16.1)	205.3 (16.1)	0.00
US Region: South	369 (26.2)	2,202 (30.4)	-0.09	184 (35.5)	692 (42.1)	-0.14	378.8 (29.7)	378.8 (29.7)	0.00
US Region: Unknown	0	0	0.00	7 (1.4)	43 (2.6)	-0.09	5.0 (0.4)	5.0 (0.4)	0.00
US Region: West	291 (20.7)	1,251 (17.3)	0.09	87 (16.8)	235 (14.3)	0.07	247.3 (19.4)	247.3 (19.4)	0.00
Year of Delivery: 2000	<11	<11	0.06	0 (0.0)	0 (0.0)	0.00	0.6 (0.0)	0.6 (0.0)	0.00
Year of Delivery: 2001	*	*	0.27	0 (0.0)	0 (0.0)	0.00	56.1 (4.4)	56.1 (4.4)	0.00
Year of Delivery: 2002	110 (7.8)	227 (3.1)	0.21	0 (0.0)	0 (0.0)	0.00	63.7 (5.0)	63.7 (5.0)	0.00
Year of Delivery: 2003	144 (10.2)	262 (3.6)	0.26	1 (0.2)	0 (0.0)	0.06	78.4 (6.2)	78.4 (6.2)	0.00
Year of Delivery: 2004	156 (11.1)	368 (5.1)	0.22	40 (7.7)	27 (1.6)	0.29	111.7 (8.8)	111.7 (8.8)	0.00
Year of Delivery: 2005	124 (8.8)	329 (4.5)	0.17	43 (8.3)	43 (2.6)	0.25	97.0 (7.6)	97.0 (7.6)	0.00
Year of Delivery: 2006	109 (7.8)	347 (4.8)	0.12	21 (4.1)	55 (3.4)	0.04	85.1 (6.7)	85.1 (6.7)	0.00
Year of Delivery: 2007	80 (5.7)	306 (4.2)	0.07	49 (9.5)	61 (3.7)	0.23	81.2 (6.4)	81.2 (6.4)	0.00
Year of Delivery: 2008	66 (4.7)	330 (4.6)	0.01	43 (8.3)	84 (5.1)	0.13	74.4 (5.8)	74.4 (5.8)	0.00
Year of Delivery: 2009	71 (5.1)	370 (5.1)	0.00	49 (9.5)	132 (8.0)	0.05	81.1 (6.4)	81.1 (6.4)	0.00
Year of Delivery: 2010	78 (5.6)	484 (6.7)	-0.05	41 (7.9)	147 (9.0)	-0.04	88.7 (7.0)	88.7 (7.0)	0.00
Year of Delivery: 2011	74 (5.3)	520 (7.2)	-0.08	50 (9.7)	147 (9.0)	0.02	88.9 (7.0)	88.9 (7.0)	0.00
Year of Delivery: 2012	45 (3.2)	541 (7.5)	-0.19	41 (7.9)	156 (9.5)	-0.06	65.7 (5.2)	67.2 (5.3)	0.01
Year of Delivery: 2013	51 (3.6)	625 (8.6)	-0.21	34 (6.6)	107 (6.5)	0.00	66.4 (5.2)	66.4 (5.2)	0.00
Year of Delivery: 2014	46 (3.3)	480 (6.6)	-0.16	27 (5.2)	102 (6.2)	-0.04	56.6 (4.4)	56.6 (4.4)	0.00
Year of Delivery: 2015	37 (2.6)	383 (5.3)	-0.14	19 (3.7)	103 (6.3)	-0.12	43.0 (3.4)	43.0 (3.4)	0.00
Year of Delivery: 2016	43 (3.1)	507 (7.0)	-0.18	14 (2.7)	105 (6.4)	-0.18	46.1 (3.6)	46.1 (3.6)	0.00

	Unadjusted, N (column %)						PS Overlap-Weighted, N (column %)		
	MAX/TAF			MarketScan			Both Cohorts Combined		
**Variable**	**Exposed**	**Unexposed**	**SMD**	**Exposed**	**Unexposed**	**SMD**	**Exposed**	**Unexposed**	**SMD**
Year of Delivery: 2017	35 (2.5)	594 (8.2)	-0.26	14 (2.7)	84 (5.1)	-0.12	41.3 (3.2)	41.3 (3.2)	0.00
Year of Delivery: 2018	24 (1.7)	425 (5.9)	-0.22	7 (1.4)	71 (4.3)	-0.18	26.4 (2.1)	26.4 (2.1)	0.00
Year of Delivery: 2019	0	0	0.00	7 (1.4)	85 (5.2)	-0.22	6.1 (0.5)	6.1 (0.5)	0.00
Year of Delivery: 2020	0	0	0.00	11 (2.1)	74 (4.5)	-0.13	8.7 (0.7)	8.7 (0.7)	0.00
N MH Diagnoses: 0	1,039 (73.9)	4,225 (58.3)	0.33	464 (89.6)	1,224 (74.5)	0.40	956.1 (75.1)	956.1 (75.1)	0.00
N MH Diagnoses: 1	148 (10.5)	1,137 (15.7)	-0.15	32 (6.2)	173 (10.5)	-0.16	135.4 (10.6)	135.4 (10.6)	0.00
N MH Diagnoses: 2-3	127 (9.0)	1,047 (14.5)	-0.17	19 (3.7)	144 (8.8)	-0.21	106.4 (8.4)	106.4 (8.4)	0.00
N MH Diagnoses: >=4	92 (6.5)	836 (11.5)	-0.17	3 (0.6)	101 (6.2)	-0.31	75.9 (6.0)	75.9 (6.0)	0.00
N ED Visits: 0	1,312 (93.3)	6,204 (85.6)	0.25	511 (98.7)	1,552 (94.5)	0.23	1,194.2 (93.8)	1,194.2 (93.8)	0.00
N ED Visits: 1	70 (5.0)	704 (9.7)	-0.18	6 (1.2)	58 (3.5)	-0.16	58.9 (4.6)	58.9 (4.6)	0.00
N ED Visits: 2-3	*	*	-0.13	1 (0.2)	28 (1.7)	-0.16	18.9 (1.5)	18.9 (1.5)	0.00
N ED Visits: >=4	<11	*	-0.11	0 (0.0)	4 (0.2)	-0.07	1.9 (0.2)	1.9 (0.2)	0.00
N MH Hospitalizations: 0	1,359 (96.7)	6,749 (93.2)	0.16	513 (99.0)	1,609 (98.0)	0.09	1,232.9 (96.8)	1,232.9 (96.8)	0.00
N MH Hospitalizations: 1	35 (2.5)	384 (5.3)	-0.15	4 (0.8)	26 (1.6)	-0.08	30.6 (2.4)	30.6 (2.4)	0.00
N MH Hospitalizations: 2-3	12 (0.9)	90 (1.2)	-0.04	1 (0.2)	7 (0.4)	-0.04	10.3 (0.8)	10.3 (0.8)	0.00
N MH Hospitalizations: >=4	0	22 (0.3)	-0.08	0 (0.0)	0 (0.0)	0.00	0.0 (0.0)	0.0 (0.0)	0.00
N Outpatient Visits: <=3	147 (10.5)	1,012 (14.0)	-0.11	29 (5.6)	99 (6.0)	-0.02	129.9 (10.2)	129.9 (10.2)	0.00
N Outpatient Visits: 4-6	252 (17.9)	1,299 (17.9)	0.00	123 (23.8)	336 (20.5)	0.08	248.1 (19.5)	248.1 (19.5)	0.00
N Outpatient Visits: 7-10	355 (25.3)	1,788 (24.7)	0.01	173 (33.4)	482 (29.4)	0.09	344.4 (27.0)	344.4 (27.0)	0.00
N Outpatient Visits: >=11	652 (46.4)	3,146 (43.4)	0.06	193 (37.3)	725 (44.2)	-0.14	551.5 (43.3)	551.5 (43.3)	0.00
N Psychotropic Medications (Except ASMs), Mean (SD)	0.0 (0.1)	0.0 (0.2)	-0.07	0.0 (0.1)	0.0 (0.2)	-0.17	0.0 (0.1)	0.0 (0.1)	0.00
Alcohol Use Disorder	17 (1.2)	277 (3.8)	-0.17	2 (0.4)	21 (1.3)	-0.10	16.6 (1.3)	16.6 (1.3)	0.00
Smoking	117 (8.3)	1,475 (20.4)	-0.35	10 (1.9)	96 (5.9)	-0.20	106.4 (8.4)	106.4 (8.4)	0.00
Substance Use Disorder	82 (5.8)	1,007 (13.9)	-0.27	6 (1.2)	43 (2.6)	-0.11	71.1 (5.6)	71.1 (5.6)	0.00
ADHD	15 (1.1)	191 (2.6)	-0.12	3 (0.6)	40 (2.4)	-0.15	15.6 (1.2)	15.6 (1.2)	0.00
Adjustment Disorder	20 (1.4)	125 (1.7)	-0.02	2 (0.4)	8 (0.5)	-0.02	15.6 (1.2)	15.6 (1.2)	0.00
Anxiety	156 (11.1)	1,489 (20.6)	-0.26	35 (6.8)	251 (15.3)	-0.27	147.7 (11.6)	147.7 (11.6)	0.00
Behavioral Disorder	20 (1.4)	131 (1.8)	-0.03	0 (0.0)	5 (0.3)	-0.08	15.0 (1.2)	15.0 (1.2)	0.00
Bipolar Disorder	81 (5.8)	610 (8.4)	-0.10	5 (1.0)	43 (2.6)	-0.12	66.9 (5.3)	66.9 (5.3)	0.00
Chronic Pain	16 (1.1)	173 (2.4)	-0.10	2 (0.4)	29 (1.8)	-0.13	15.7 (1.2)	15.7 (1.2)	0.00
Chronic Fatigue	111 (7.9)	695 (9.6)	-0.06	38 (7.3)	210 (12.8)	-0.18	107.5 (8.4)	107.5 (8.4)	0.00
Depression	196 (13.9)	1,660 (22.9)	-0.23	28 (5.4)	234 (14.3)	-0.30	170.9 (13.4)	170.9 (13.4)	0.00
Migraine/Headache	133 (9.5)	991 (13.7)	-0.13	32 (6.2)	316 (19.2)	-0.40	128.1 (10.1)	128.1 (10.1)	0.00
Neuromuscular Disorder	23 (1.6)	312 (4.3)	-0.16	9 (1.7)	73 (4.5)	-0.16	27.4 (2.2)	27.4 (2.2)	0.00
Neuropathic Pain	53 (3.8)	463 (6.4)	-0.12	28 (5.4)	169 (10.3)	-0.18	61.2 (4.8)	61.2 (4.8)	0.00
Other MH Disorders	36 (2.6)	358 (4.9)	-0.13	1 (0.2)	50 (3.1)	-0.23	30.3 (2.4)	30.3 (2.4)	0.00
Other Developmental Disorders	23 (1.6)	82 (1.1)	0.04	0 (0.0)	4 (0.2)	-0.07	14.0 (1.1)	14.0 (1.1)	0.00
Personality Disorder	12 (0.9)	126 (1.7)	-0.08	0 (0.0)	7 (0.4)	-0.09	9.9 (0.8)	9.9 (0.8)	0.00
Psychosis	25 (1.8)	186 (2.6)	-0.05	0 (0.0)	21 (1.3)	-0.16	18.4 (1.4)	18.4 (1.4)	0.00
Schizophrenia/Schizoaffective Disorder	<11	*	-0.06	1 (0.2)	6 (0.4)	-0.03	6.9 (0.5)	6.9 (0.5)	0.00
Sleep Disorder	32 (2.3)	282 (3.9)	-0.09	11 (2.1)	98 (6.0)	-0.20	33.4 (2.6)	33.4 (2.6)	0.00
Asthma	156 (11.1)	1,144 (15.8)	-0.14	17 (3.3)	140 (8.5)	-0.22	129.0 (10.1)	129.0 (10.1)	0.00
Autoimmune Disorder	17 (1.2)	206 (2.8)	-0.12	17 (3.3)	96 (5.9)	-0.12	27.2 (2.1)	27.2 (2.1)	0.00
Hyperemesis/NVP	304 (21.6)	2,529 (34.9)	-0.30	33 (6.4)	351 (21.4)	-0.44	262.6 (20.6)	262.6 (20.6)	0.00
Influenca Infection	24 (1.7)	180 (2.5)	-0.05	3 (0.6)	35 (2.1)	-0.13	21.6 (1.7)	21.6 (1.7)	0.00

	Unadjusted, N (column %)						PS Overlap-Weighted, N (column %)		
	MAX/TAF			MarketScan			Both Cohorts Combined		
Variable	Exposed	Unexposed	SMD	Exposed	Unexposed	SMD	Exposed	Unexposed	SMD
Obstetric Comorbidity Score: 0	604 (43.0)	2,642 (36.5)	0.13	181 (34.9)	634 (38.6)	-0.08	521.6 (41.0)	521.6 (41.0)	0.00
Obstetric Comorbidity Score: 1	353 (25.1)	1,590 (22.0)	0.07	149 (28.8)	369 (22.5)	0.14	317.0 (24.9)	317.0 (24.9)	0.00
Obstetric Comorbidity Score: 2	169 (12.0)	1,133 (15.6)	-0.10	85 (16.4)	265 (16.1)	0.01	173.1 (13.6)	173.1 (13.6)	0.00
Obstetric Comorbidity Score: >=3	280 (19.9)	1,880 (26.0)	-0.14	103 (19.9)	374 (22.8)	-0.07	262.1 (20.6)	262.1 (20.6)	0.00
Overweight/Obesity	97 (6.9)	811 (11.2)	-0.15	40 (7.7)	185 (11.3)	-0.12	101.2 (7.9)	101.2 (7.9)	0.00
Pre-gestational Diabetes	35 (2.5)	241 (3.3)	-0.05	6 (1.2)	43 (2.6)	-0.11	31.4 (2.5)	31.4 (2.5)	0.00
Pre-gestational Hypertension	112 (8.0)	835 (11.5)	-0.12	45 (8.7)	170 (10.4)	-0.06	113.1 (8.9)	113.1 (8.9)	0.00
Poor Nutrition	30 (2.1)	247 (3.4)	-0.08	16 (3.1)	123 (7.5)	-0.20	35.5 (2.8)	35.5 (2.8)	0.00
Renal Disease	<11	*	-0.05	3 (0.6)	20 (1.2)	-0.07	9.8 (0.8)	9.8 (0.8)	0.00
TORCH Infection	32 (2.3)	277 (3.8)	-0.09	8 (1.5)	36 (2.2)	-0.05	30.2 (2.4)	30.2 (2.4)	0.00
Other Sexually Transmitted Diseases	85 (6.1)	812 (11.2)	-0.18	3 (0.6)	56 (3.4)	-0.20	69.9 (5.5)	69.9 (5.5)	0.00
Prenatal Folic Acid Supplementation	1,038 (73.8)	4,000 (55.2)	0.40	314 (60.6)	527 (32.1)	0.60	832.6 (65.4)	832.6 (65.4)	0.00
Antidepressants	249 (17.7)	1,452 (20.0)	-0.06	57 (11.0)	277 (16.9)	-0.17	211.3 (16.6)	211.3 (16.6)	0.00
Antidiabetics	16 (1.1)	86 (1.2)	0.00	13 (2.5)	52 (3.2)	-0.04	20.5 (1.6)	20.5 (1.6)	0.00
Antihypertensives	70 (5.0)	450 (6.2)	-0.05	19 (3.7)	104 (6.3)	-0.12	66.5 (5.2)	66.5 (5.2)	0.00
Antipsychotics	69 (4.9)	430 (5.9)	-0.05	5 (1.0)	43 (2.6)	-0.12	54.4 (4.3)	54.4 (4.3)	0.00
Anxiolytics/Hypnotics/Other Sedatives	103 (7.3)	742 (10.2)	-0.10	12 (2.3)	87 (5.3)	-0.16	87.1 (6.8)	87.1 (6.8)	0.00
Barbiturates	83 (5.9)	299 (4.1)	0.08	17 (3.3)	56 (3.4)	-0.01	55.7 (4.4)	55.7 (4.4)	0.00
Benzodiazepines	159 (11.3)	760 (10.5)	0.03	37 (7.1)	160 (9.7)	-0.09	131.1 (10.3)	131.1 (10.3)	0.00
Corticosteroids	253 (18.0)	1,221 (16.9)	0.03	73 (14.1)	273 (16.6)	-0.07	209.6 (16.5)	209.6 (16.5)	0.00
Insulin	17 (1.2)	131 (1.8)	-0.05	4 (0.8)	26 (1.6)	-0.08	16.5 (1.3)	16.5 (1.3)	0.00
Lithium	<11	*	-0.05	0 (0.0)	2 (0.1)	-0.05	1.7 (0.1)	1.7 (0.1)	0.00
NSAIDs	283 (20.1)	1,747 (24.1)	-0.10	32 (6.2)	148 (9.0)	-0.11	227.7 (17.9)	227.7 (17.9)	0.00
Opioid Agonist Therapy	<11	*	-0.09	0 (0.0)	8 (0.5)	-0.10	3.8 (0.3)	3.8 (0.3)	0.00
Prescription Opioids	392 (27.9)	2,514 (34.7)	-0.15	63 (12.2)	296 (18.0)	-0.16	330.5 (25.9)	330.5 (25.9)	0.00
Progestins	87 (6.2)	459 (6.3)	-0.01	80 (15.4)	231 (14.1)	0.04	104.5 (8.2)	104.5 (8.2)	0.00
Psychostimulants	13 (0.9)	131 (1.8)	-0.08	3 (0.6)	44 (2.7)	-0.17	12.5 (1.0)	12.5 (1.0)	0.00
Teratogens, Known	<11	*	-0.04	2 (0.4)	8 (0.5)	-0.02	3.6 (0.3)	3.6 (0.3)	0.00
Teratogens, Suspected	199 (14.2)	1,123 (15.5)	-0.04	39 (7.5)	181 (11.0)	-0.12	163.6 (12.8)	163.6 (12.8)	0.00
Triptans	29 (2.1)	183 (2.5)	-0.03	10 (1.9)	60 (3.7)	-0.10	29.3 (2.3)	29.3 (2.3)	0.00
Adequacy of Prenatal Care Utilization: Inadequate	606 (43.1)	2,867 (39.6)	0.07	314 (60.6)	860 (52.4)	0.17	591.7 (46.5)	591.7 (46.5)	0.00
Adequacy of Prenatal Care Utilization: Intermediate	203 (14.4)	1,100 (15.2)	-0.02	102 (19.7)	358 (21.8)	-0.05	204.9 (16.1)	204.9 (16.1)	0.00
Adequacy of Prenatal Care Utilization: Adequate	168 (12.0)	1,121 (15.5)	-0.10	62 (12.0)	208 (12.7)	-0.02	154.2 (12.1)	154.2 (12.1)	0.00
Adequacy of Prenatal Care Utilization: Adequate Plus	429 (30.5)	2,157 (29.8)	0.02	40 (7.7)	216 (13.2)	-0.18	323.1 (25.4)	323.1 (25.4)	0.00
Multiple Gestation	16 (1.1)	165 (2.3)	-0.09	14 (2.7)	43 (2.6)	0.01	21.6 (1.7)	21.6 (1.7)	0.00
Gestational Month of Prenatal Care Initiation: 1	69 (4.9)	390 (5.4)	-0.02	35 (6.8)	110 (6.7)	0.00	68.4 (5.4)	68.4 (5.4)	0.00
Gestational Month of Prenatal Care Initiation: 2	501 (35.6)	2,637 (36.4)	-0.02	213 (41.1)	714 (43.5)	-0.05	474.9 (37.3)	474.9 (37.3)	0.00
Gestational Month of Prenatal Care Initiation: 3	331 (23.5)	1,788 (24.7)	-0.03	127 (24.5)	429 (26.1)	-0.04	302.5 (23.8)	302.5 (23.8)	0.00
Gestational Month of Prenatal Care Initiation: 4	147 (10.5)	781 (10.8)	-0.01	35 (6.8)	100 (6.1)	0.03	123.1 (9.7)	123.1 (9.7)	0.00
Gestational Month of Prenatal Care Initiation: 5	113 (8.0)	486 (6.7)	0.05	16 (3.1)	63 (3.8)	-0.04	86.3 (6.8)	86.3 (6.8)	0.00
Gestational Month of Prenatal Care Initiation: 6	42 (3.0)	277 (3.8)	-0.05	8 (1.5)	30 (1.8)	-0.02	37.1 (2.9)	37.1 (2.9)	0.00
Gestational Month of Prenatal Care Initiation: 7	45 (3.2)	197 (2.7)	0.03	13 (2.5)	30 (1.8)	0.05	35.1 (2.8)	35.1 (2.8)	0.00
Gestational Month of Prenatal Care Initiation: 8	30 (2.1)	142 (2.0)	0.01	7 (1.4)	19 (1.2)	0.02	24.9 (2.0)	24.9 (2.0)	0.00
Gestational Month of Prenatal Care Initiation: 9	26 (1.9)	134 (1.9)	0.00	8 (1.5)	17 (1.0)	0.05	21.6 (1.7)	21.6 (1.7)	0.00
Gestational Month of Prenatal Care Initiation: None	102 (7.3)	413 (5.7)	0.06	56 (10.8)	130 (7.9)	0.10	99.9 (7.9)	99.9 (7.9)	0.00

	Unadjusted, N (column %)						PS Overlap-Weighted, N (column %)		
	MAX/TAF			MarketScan			Both Cohorts Combined		
**Variable**	**Exposed**	**Unexposed**	**SMD**	**Exposed**	**Unexposed**	**SMD**	**Exposed**	**Unexposed**	**SMD**
Gestational Month of Prenatal Vitamin Initiation: 1	115 (8.2)	468 (6.5)	0.07	38 (7.3)	64 (3.9)	0.15	93.4 (7.3)	93.4 (7.3)	0.00
Gestational Month of Prenatal Vitamin Initiation: 2	418 (29.7)	1,711 (23.6)	0.14	90 (17.4)	188 (11.5)	0.17	335.7 (26.4)	335.7 (26.4)	0.00
Gestational Month of Prenatal Vitamin Initiation: 3	203 (14.4)	946 (13.1)	0.04	39 (7.5)	104 (6.3)	0.05	163.7 (12.9)	163.7 (12.9)	0.00
Gestational Month of Prenatal Vitamin Initiation: 4	94 (6.7)	479 (6.6)	0.00	11 (2.1)	39 (2.4)	-0.02	74.1 (5.8)	74.1 (5.8)	0.00
Gestational Month of Prenatal Vitamin Initiation: 5	75 (5.3)	286 (4.0)	0.07	7 (1.4)	25 (1.5)	-0.01	56.1 (4.4)	56.1 (4.4)	0.00
Gestational Month of Prenatal Vitamin Initiation: 6	43 (3.1)	197 (2.7)	0.02	3 (0.6)	14 (0.9)	-0.03	31.5 (2.5)	31.5 (2.5)	0.00
Gestational Month of Prenatal Vitamin Initiation: 7	30 (2.1)	166 (2.3)	-0.01	9 (1.7)	25 (1.5)	0.02	27.1 (2.1)	27.1 (2.1)	0.00
Gestational Month of Prenatal Vitamin Initiation: 8	22 (1.6)	98 (1.4)	0.02	5 (1.0)	14 (0.9)	0.01	19.2 (1.5)	19.2 (1.5)	0.00
Gestational Month of Prenatal Vitamin Initiation: 9	25 (1.8)	224 (3.1)	-0.09	1 (0.2)	31 (1.9)	-0.17	20.7 (1.6)	20.7 (1.6)	0.00
Gestational Month of Prenatal Vitamin Initiation: -1	28 (2.0)	60 (0.8)	0.10	29 (5.6)	16 (1.0)	0.26	24.6 (1.9)	24.6 (1.9)	0.00
Gestational Month of Prenatal Vitamin Initiation: -2	52 (3.7)	113 (1.6)	0.13	35 (6.8)	34 (2.1)	0.23	42.6 (3.4)	42.6 (3.4)	0.00
Gestational Month of Prenatal Vitamin Initiation: -3	110 (7.8)	163 (2.3)	0.26	68 (13.1)	74 (4.5)	0.31	84.7 (6.7)	84.7 (6.7)	0.00
Gestational Month of Prenatal Vitamin Initiation: None	191 (13.6)	2,334 (32.2)	-0.45	183 (35.3)	1,014 (61.8)	-0.55	300.6 (23.6)	300.6 (23.6)	0.00
**Phenytoin**									
N Total	1,431	7,245		164	1,642		873.5	873.5	
Age in Years: <=19	145 (10.1)	1,399 (19.3)	-0.26	0 (0.0)	18 (1.1)	-0.15	88.5 (10.1)	88.5 (10.1)	0.00
Age in Years: 20-24	511 (35.7)	2,612 (36.1)	-0.01	11 (6.7)	149 (9.1)	-0.09	295.7 (33.9)	295.7 (33.9)	0.00
Age in Years: 25-29	400 (28.0)	1,941 (26.8)	0.03	37 (22.6)	558 (34.0)	-0.26	238.2 (27.3)	238.2 (27.3)	0.00
Age in Years: 30-34	217 (15.2)	904 (12.5)	0.08	57 (34.8)	606 (36.9)	-0.04	144.0 (16.5)	144.0 (16.5)	0.00
Age in Years: 35-39	132 (9.2)	318 (4.4)	0.19	39 (23.8)	250 (15.2)	0.22	86.6 (9.9)	86.6 (9.9)	0.00
Age in Years: >=40	26 (1.8)	71 (1.0)	0.07	20 (12.2)	61 (3.7)	0.32	20.4 (2.3)	20.4 (2.3)	0.00
Race/Ethnicity: Asian or other Pacific Islander	<11	*	-0.03	N/A	N/A	N/A	N/A	N/A	N/A
Race/Ethnicity: Black or African American	*	*	0.08	N/A	N/A	N/A	N/A	N/A	N/A
Race/Ethnicity: Hispanic or Latino	257 (18.0)	963 (13.3)	0.13	N/A	N/A	N/A	N/A	N/A	N/A
Race/Ethnicity: Unknown or Other	44 (3.1)	340 (4.7)	-0.08	N/A	N/A	N/A	N/A	N/A	N/A
Race/Ethnicity: White	643 (44.9)	3,729 (51.5)	-0.13	N/A	N/A	N/A	N/A	N/A	N/A
US Region: Midwest	440 (30.8)	2,598 (35.9)	-0.11	40 (24.4)	402 (24.5)	0.00	269.6 (30.9)	269.6 (30.9)	0.00
US Region: Northeast	189 (13.2)	1,194 (16.5)	-0.09	20 (12.2)	270 (16.4)	-0.12	117.6 (13.5)	117.6 (13.5)	0.00
US Region: South	467 (32.6)	2,202 (30.4)	0.05	70 (42.7)	692 (42.1)	0.01	296.2 (33.9)	296.2 (33.9)	0.00
US Region: Unknown	0	0	0.00	3 (1.8)	43 (2.6)	-0.05	1.0 (0.1)	1.0 (0.1)	0.00
US Region: West	335 (23.4)	1,251 (17.3)	0.15	31 (18.9)	235 (14.3)	0.12	189.1 (21.7)	189.1 (21.7)	0.00
Year of Delivery: 2000	<11	<11	0.07	0 (0.0)	0 (0.0)	0.00	0.7 (0.1)	0.7 (0.1)	0.00
Year of Delivery: 2001	*	*	0.26	0 (0.0)	0 (0.0)	0.00	50.9 (5.8)	50.9 (5.8)	0.00
Year of Delivery: 2002	131 (9.2)	227 (3.1)	0.25	0 (0.0)	0 (0.0)	0.00	65.6 (7.5)	65.6 (7.5)	0.00
Year of Delivery: 2003	146 (10.2)	262 (3.6)	0.26	0 (0.0)	0 (0.0)	0.00	65.0 (7.4)	65.0 (7.4)	0.00
Year of Delivery: 2004	157 (11.0)	368 (5.1)	0.22	15 (9.2)	27 (1.6)	0.34	88.3 (10.1)	88.3 (10.1)	0.00
Year of Delivery: 2005	146 (10.2)	329 (4.5)	0.22	15 (9.2)	43 (2.6)	0.28	86.8 (9.9)	86.8 (9.9)	0.00
Year of Delivery: 2006	120 (8.4)	347 (4.8)	0.15	13 (7.9)	55 (3.4)	0.20	73.8 (8.5)	73.8 (8.5)	0.00
Year of Delivery: 2007	104 (7.3)	306 (4.2)	0.13	16 (9.8)	61 (3.7)	0.24	67.2 (7.7)	67.2 (7.7)	0.00
Year of Delivery: 2008	102 (7.1)	330 (4.6)	0.11	17 (10.4)	84 (5.1)	0.20	71.9 (8.2)	71.9 (8.2)	0.00
Year of Delivery: 2009	77 (5.4)	370 (5.1)	0.01	16 (9.8)	132 (8.0)	0.06	55.5 (6.4)	55.5 (6.4)	0.00
Year of Delivery: 2010	75 (5.2)	484 (6.7)	-0.06	15 (9.2)	147 (9.0)	0.01	53.3 (6.1)	53.3 (6.1)	0.00
Year of Delivery: 2011	71 (5.0)	520 (7.2)	-0.09	13 (7.9)	147 (9.0)	-0.04	53.4 (6.1)	53.4 (6.1)	0.00
Year of Delivery: 2012	44 (3.1)	541 (7.5)	-0.20	7 (4.3)	156 (9.5)	-0.21	29.5 (3.4)	31.1 (3.6)	0.01
Year of Delivery: 2013	41 (2.9)	625 (8.6)	-0.25	14 (8.5)	107 (6.5)	0.08	36.6 (4.2)	36.6 (4.2)	0.00

	Unadjusted, N (column %)						PS Overlap-Weighted, N (column %)		
	MAX/TAF			MarketScan			Both Cohorts Combined		
Variable	Exposed	Unexposed	SMD	Exposed	Unexposed	SMD	Exposed	Unexposed	SMD
Year of Delivery: 2014	32 (2.2)	480 (6.6)	-0.21	10 (6.1)	102 (6.2)	0.00	27.6 (3.2)	27.6 (3.2)	0.00
Year of Delivery: 2015	13 (0.9)	383 (5.3)	-0.25	5 (3.1)	103 (6.3)	-0.15	9.7 (1.1)	9.7 (1.1)	0.00
Year of Delivery: 2016	14 (1.0)	507 (7.0)	-0.31	0 (0.0)	105 (6.4)	-0.37	6.8 (0.8)	6.8 (0.8)	0.00
Year of Delivery: 2017	26 (1.8)	594 (8.2)	-0.30	3 (1.8)	84 (5.1)	-0.18	16.3 (1.9)	16.3 (1.9)	0.00
Year of Delivery: 2018	19 (1.3)	425 (5.9)	-0.25	3 (1.8)	71 (4.3)	-0.14	13.1 (1.5)	13.1 (1.5)	0.00
Year of Delivery: 2019	0	0	0.00	1 (0.6)	85 (5.2)	-0.27	0.0 (0.0)	0.0 (0.0)	0.00
Year of Delivery: 2020	0	0	0.00	1 (0.6)	74 (4.5)	-0.25	0.0 (0.0)	0.0 (0.0)	0.00
N MH Diagnoses: 0	995 (69.5)	4,225 (58.3)	0.24	142 (86.6)	1,224 (74.5)	0.31	611.6 (70.0)	611.6 (70.0)	0.00
N MH Diagnoses: 1	172 (12.0)	1,137 (15.7)	-0.11	10 (6.1)	173 (10.5)	-0.16	112.5 (12.9)	112.5 (12.9)	0.00
N MH Diagnoses: 2-3	171 (12.0)	1,047 (14.5)	-0.07	8 (4.9)	144 (8.8)	-0.15	98.3 (11.3)	98.3 (11.3)	0.00
N MH Diagnoses: >=4	93 (6.5)	836 (11.5)	-0.18	4 (2.4)	101 (6.2)	-0.18	51.1 (5.9)	51.1 (5.9)	0.00
N ED Visits: 0	1,312 (91.7)	6,204 (85.6)	0.19	161 (98.2)	1,552 (94.5)	0.20	803.8 (92.0)	803.8 (92.0)	0.00
N ED Visits: 1	78 (5.5)	704 (9.7)	-0.16	3 (1.8)	58 (3.5)	-0.11	51.1 (5.9)	51.1 (5.9)	0.00
N ED Visits: 2-3	*	*	-0.07	0 (0.0)	28 (1.7)	-0.19	14.5 (1.7)	14.5 (1.7)	0.00
N ED Visits: >=4	<11	*	-0.06	0 (0.0)	4 (0.2)	-0.07	4.1 (0.5)	4.1 (0.5)	0.00
N MH Hospitalizations: 0	1,362 (95.2)	6,749 (93.2)	0.09	162 (98.8)	1,609 (98.0)	0.06	834.8 (95.6)	834.8 (95.6)	0.00
N MH Hospitalizations: 1	54 (3.8)	384 (5.3)	-0.07	2 (1.2)	26 (1.6)	-0.03	30.6 (3.5)	30.6 (3.5)	0.00
N MH Hospitalizations: 2-3	<11	*	-0.06	0 (0.0)	7 (0.4)	-0.09	5.6 (0.6)	5.6 (0.6)	0.00
N MH Hospitalizations: >=4	<11	*	0.01	0 (0.0)	0 (0.0)	0.00	2.5 (0.3)	2.5 (0.3)	0.00
N Outpatient Visits: <=3	202 (14.1)	1,012 (14.0)	0.00	9 (5.5)	99 (6.0)	-0.02	120.9 (13.8)	120.9 (13.8)	0.00
N Outpatient Visits: 4-6	239 (16.7)	1,299 (17.9)	-0.03	42 (25.6)	336 (20.5)	0.12	161.3 (18.5)	161.3 (18.5)	0.00
N Outpatient Visits: 7-10	351 (24.5)	1,788 (24.7)	0.00	54 (32.9)	482 (29.4)	0.08	227.5 (26.1)	227.5 (26.1)	0.00
N Outpatient Visits: >=11	639 (44.7)	3,146 (43.4)	0.02	59 (36.0)	725 (44.2)	-0.17	363.8 (41.7)	363.8 (41.7)	0.00
N Psychotropic Medications (Except ASMs), Mean (SD)	0.0 (0.1)	0.0 (0.2)	-0.12	0.0 (0.1)	0.0 (0.2)	-0.13	0.0 (0.1)	0.0 (0.1)	0.00
Alcohol Use Disorder	36 (2.5)	277 (3.8)	-0.07	0 (0.0)	21 (1.3)	-0.16	21.1 (2.4)	21.1 (2.4)	0.00
Smoking	200 (14.0)	1,475 (20.4)	-0.17	5 (3.1)	96 (5.9)	-0.14	115.2 (13.2)	115.2 (13.2)	0.00
Substance Use Disorder	144 (10.1)	1,007 (13.9)	-0.12	4 (2.4)	43 (2.6)	-0.01	78.2 (9.0)	78.2 (9.0)	0.00
ADHD	11 (0.8)	191 (2.6)	-0.14	2 (1.2)	40 (2.4)	-0.09	8.8 (1.0)	8.8 (1.0)	0.00
Adjustment Disorder	28 (2.0)	125 (1.7)	0.02	0 (0.0)	8 (0.5)	-0.10	13.0 (1.5)	13.0 (1.5)	0.00
Anxiety	190 (13.3)	1,489 (20.6)	-0.19	10 (6.1)	251 (15.3)	-0.30	109.3 (12.5)	109.3 (12.5)	0.00
Behavioral Disorder	16 (1.1)	131 (1.8)	-0.06	1 (0.6)	5 (0.3)	0.05	6.6 (0.8)	6.6 (0.8)	0.00
Bipolar Disorder	75 (5.2)	610 (8.4)	-0.13	3 (1.8)	43 (2.6)	-0.05	41.9 (4.8)	41.9 (4.8)	0.00
Chronic Pain	13 (0.9)	173 (2.4)	-0.12	2 (1.2)	29 (1.8)	-0.05	7.4 (0.9)	7.4 (0.9)	0.00
Chronic Fatigue	125 (8.7)	695 (9.6)	-0.03	10 (6.1)	210 (12.8)	-0.23	70.9 (8.1)	70.9 (8.1)	0.00
Depression	262 (18.3)	1,660 (22.9)	-0.11	13 (7.9)	234 (14.3)	-0.20	148.5 (17.0)	148.5 (17.0)	0.00
Migraine/Headache	153 (10.7)	991 (13.7)	-0.09	13 (7.9)	316 (19.2)	-0.33	87.2 (10.0)	87.2 (10.0)	0.00
Neuromuscular Disorder	38 (2.7)	312 (4.3)	-0.09	2 (1.2)	73 (4.5)	-0.20	18.3 (2.1)	18.3 (2.1)	0.00
Neuropathic Pain	69 (4.8)	463 (6.4)	-0.07	10 (6.1)	169 (10.3)	-0.15	41.3 (4.7)	41.3 (4.7)	0.00
Other MH Disorders	48 (3.4)	358 (4.9)	-0.08	5 (3.1)	50 (3.1)	0.00	24.0 (2.8)	24.0 (2.8)	0.00
Other Developmental Disorders	11 (0.8)	82 (1.1)	-0.04	1 (0.6)	4 (0.2)	0.06	7.5 (0.9)	7.5 (0.9)	0.00
Personality Disorder	16 (1.1)	126 (1.7)	-0.05	1 (0.6)	7 (0.4)	0.03	8.6 (1.0)	8.6 (1.0)	0.00
Psychosis	30 (2.1)	186 (2.6)	-0.03	2 (1.2)	21 (1.3)	-0.01	14.1 (1.6)	14.1 (1.6)	0.00
Schizophrenia/Schizoaffective Disorder	24 (1.7)	90 (1.2)	0.04	0 (0.0)	6 (0.4)	-0.09	11.4 (1.3)	11.4 (1.3)	0.00
Sleep Disorder	50 (3.5)	282 (3.9)	-0.02	5 (3.1)	98 (6.0)	-0.14	27.3 (3.1)	27.3 (3.1)	0.00
Asthma	198 (13.8)	1,144 (15.8)	-0.06	8 (4.9)	140 (8.5)	-0.15	112.2 (12.9)	112.2 (12.9)	0.00

	Unadjusted, N (column %)						PS Overlap-Weighted, N (column %)		
	MAX/TAF			MarketScan			Both Cohorts Combined		
**Variable**	**Exposed**	**Unexposed**	**SMD**	**Exposed**	**Unexposed**	**SMD**	**Exposed**	**Unexposed**	**SMD**
Autoimmune Disorder	34 (2.4)	206 (2.8)	-0.03	7 (4.3)	96 (5.9)	-0.07	21.1 (2.4)	21.1 (2.4)	0.00
Hyperemesis/NVP	361 (25.2)	2,529 (34.9)	-0.21	30 (18.3)	351 (21.4)	-0.08	216.7 (24.8)	216.7 (24.8)	0.00
Influenca Infection	26 (1.8)	180 (2.5)	-0.05	7 (4.3)	35 (2.1)	0.12	19.6 (2.2)	19.6 (2.2)	0.00
Obstetric Comorbidity Score: 0	532 (37.2)	2,642 (36.5)	0.01	52 (31.7)	634 (38.6)	-0.14	326.1 (37.3)	326.1 (37.3)	0.00
Obstetric Comorbidity Score: 1	323 (22.6)	1,590 (22.0)	0.02	42 (25.6)	369 (22.5)	0.07	207.0 (23.7)	207.0 (23.7)	0.00
Obstetric Comorbidity Score: 2	192 (13.4)	1,133 (15.6)	-0.06	24 (14.6)	265 (16.1)	-0.04	118.4 (13.6)	118.4 (13.6)	0.00
Obstetric Comorbidity Score: >=3	384 (26.8)	1,880 (26.0)	0.02	46 (28.1)	374 (22.8)	0.12	221.9 (25.4)	221.9 (25.4)	0.00
Overweight/Obesity	84 (5.9)	811 (11.2)	-0.19	11 (6.7)	185 (11.3)	-0.16	54.5 (6.2)	54.5 (6.2)	0.00
Pre-gestational Diabetes	38 (2.7)	241 (3.3)	-0.04	3 (1.8)	43 (2.6)	-0.05	25.9 (3.0)	25.9 (3.0)	0.00
Pre-gestational Hypertension	145 (10.1)	835 (11.5)	-0.04	14 (8.5)	170 (10.4)	-0.06	88.4 (10.1)	88.4 (10.1)	0.00
Poor Nutrition	33 (2.3)	247 (3.4)	-0.07	3 (1.8)	123 (7.5)	-0.27	20.6 (2.4)	20.6 (2.4)	0.00
Renal Disease	<11	*	-0.06	2 (1.2)	20 (1.2)	0.00	6.3 (0.7)	6.3 (0.7)	0.00
TORCH Infection	42 (2.9)	277 (3.8)	-0.05	3 (1.8)	36 (2.2)	-0.03	21.7 (2.5)	21.7 (2.5)	0.00
Other Sexually Transmitted Diseases	143 (10.0)	812 (11.2)	-0.04	4 (2.4)	56 (3.4)	-0.06	79.9 (9.2)	79.9 (9.2)	0.00
Prenatal Folic Acid Supplementation	975 (68.1)	4,000 (55.2)	0.27	97 (59.2)	527 (32.1)	0.56	557.0 (63.8)	557.0 (63.8)	0.00
Antidepressants	280 (19.6)	1,452 (20.0)	-0.01	23 (14.0)	277 (16.9)	-0.08	144.6 (16.6)	144.6 (16.6)	0.00
Antidiabetics	16 (1.1)	86 (1.2)	-0.01	4 (2.4)	52 (3.2)	-0.04	12.4 (1.4)	12.4 (1.4)	0.00
Antihypertensives	83 (5.8)	450 (6.2)	-0.02	8 (4.9)	104 (6.3)	-0.06	45.3 (5.2)	45.3 (5.2)	0.00
Antipsychotics	100 (7.0)	430 (5.9)	0.04	5 (3.1)	43 (2.6)	0.03	50.5 (5.8)	50.5 (5.8)	0.00
Anxiolytics/Hypnotics/Other Sedatives	157 (11.0)	742 (10.2)	0.02	12 (7.3)	87 (5.3)	0.08	87.4 (10.0)	87.4 (10.0)	0.00
Barbiturates	113 (7.9)	299 (4.1)	0.16	12 (7.3)	56 (3.4)	0.17	40.6 (4.7)	40.6 (4.7)	0.00
Benzodiazepines	172 (12.0)	760 (10.5)	0.05	15 (9.2)	160 (9.7)	-0.02	94.3 (10.8)	94.3 (10.8)	0.00
Corticosteroids	274 (19.2)	1,221 (16.9)	0.06	30 (18.3)	273 (16.6)	0.04	162.8 (18.6)	162.8 (18.6)	0.00
Insulin	18 (1.3)	131 (1.8)	-0.04	1 (0.6)	26 (1.6)	-0.09	12.3 (1.4)	12.3 (1.4)	0.00
Lithium	<11	*	-0.02	2 (1.2)	2 (0.1)	0.13	4.1 (0.5)	4.1 (0.5)	0.00
NSAIDs	351 (24.5)	1,747 (24.1)	0.01	16 (9.8)	148 (9.0)	0.03	188.2 (21.6)	188.2 (21.6)	0.00
Opioid Agonist Therapy	<11	*	-0.07	0 (0.0)	8 (0.5)	-0.10	3.7 (0.4)	3.7 (0.4)	0.00
Prescription Opioids	569 (39.8)	2,514 (34.7)	0.10	33 (20.1)	296 (18.0)	0.05	312.0 (35.7)	312.0 (35.7)	0.00
Progestins	85 (5.9)	459 (6.3)	-0.02	21 (12.8)	231 (14.1)	-0.04	60.8 (7.0)	60.8 (7.0)	0.00
Psychostimulants	<11	*	-0.16	1 (0.6)	44 (2.7)	-0.16	2.9 (0.3)	2.9 (0.3)	0.00
Teratogens, Known	11 (0.8)	34 (0.5)	0.04	0 (0.0)	8 (0.5)	-0.10	6.1 (0.7)	6.1 (0.7)	0.00
Teratogens, Suspected	224 (15.7)	1,123 (15.5)	0.00	27 (16.5)	181 (11.0)	0.16	139.5 (16.0)	139.5 (16.0)	0.00
Triptans	46 (3.2)	183 (2.5)	0.04	4 (2.4)	60 (3.7)	-0.07	21.4 (2.5)	21.4 (2.5)	0.00
Adequacy of Prenatal Care Utilization: Inadequate	673 (47.0)	2,867 (39.6)	0.15	99 (60.4)	860 (52.4)	0.16	429.4 (49.2)	429.4 (49.2)	0.00
Adequacy of Prenatal Care Utilization: Intermediate	196 (13.7)	1,100 (15.2)	-0.04	31 (18.9)	358 (21.8)	-0.07	129.0 (14.8)	129.0 (14.8)	0.00
Adequacy of Prenatal Care Utilization: Adequate	187 (13.1)	1,121 (15.5)	-0.07	16 (9.8)	208 (12.7)	-0.09	104.8 (12.0)	104.8 (12.0)	0.00
Adequacy of Prenatal Care Utilization: Adequate Plus	375 (26.2)	2,157 (29.8)	-0.08	18 (11.0)	216 (13.2)	-0.07	210.3 (24.1)	210.3 (24.1)	0.00
Multiple Gestation	40 (2.8)	165 (2.3)	0.03	5 (3.1)	43 (2.6)	0.03	22.9 (2.6)	22.9 (2.6)	0.00
Gestational Month of Prenatal Care Initiation: 1	81 (5.7)	390 (5.4)	0.01	7 (4.3)	110 (6.7)	-0.11	49.8 (5.7)	49.8 (5.7)	0.00
Gestational Month of Prenatal Care Initiation: 2	436 (30.5)	2,637 (36.4)	-0.13	69 (42.1)	714 (43.5)	-0.03	287.0 (32.9)	287.0 (32.9)	0.00
Gestational Month of Prenatal Care Initiation: 3	353 (24.7)	1,788 (24.7)	0.00	36 (22.0)	429 (26.1)	-0.10	207.1 (23.7)	207.1 (23.7)	0.00
Gestational Month of Prenatal Care Initiation: 4	150 (10.5)	781 (10.8)	-0.01	13 (7.9)	100 (6.1)	0.07	85.5 (9.8)	85.5 (9.8)	0.00
Gestational Month of Prenatal Care Initiation: 5	112 (7.8)	486 (6.7)	0.04	8 (4.9)	63 (3.8)	0.05	68.5 (7.9)	68.5 (7.9)	0.00
Gestational Month of Prenatal Care Initiation: 6	78 (5.5)	277 (3.8)	0.08	4 (2.4)	30 (1.8)	0.04	39.8 (4.6)	39.8 (4.6)	0.00
Gestational Month of Prenatal Care Initiation: 7	45 (3.1)	197 (2.7)	0.03	3 (1.8)	30 (1.8)	0.00	26.9 (3.1)	26.9 (3.1)	0.00

	Unadjusted, N (column %)						PS Overlap-Weighted, N (column %)		
	MAX/TAF			MarketScan			Both Cohorts Combined		
Variable	Exposed	Unexposed	SMD	Exposed	Unexposed	SMD	Exposed	Unexposed	SMD
Gestational Month of Prenatal Care Initiation: 8	41 (2.9)	142 (2.0)	0.06	2 (1.2)	19 (1.2)	0.01	18.7 (2.1)	18.7 (2.1)	0.00
Gestational Month of Prenatal Care Initiation: 9	19 (1.3)	134 (1.9)	-0.04	2 (1.2)	17 (1.0)	0.02	14.9 (1.7)	14.9 (1.7)	0.00
Gestational Month of Prenatal Care Initiation: None	116 (8.1)	413 (5.7)	0.09	20 (12.2)	130 (7.9)	0.14	75.2 (8.6)	75.2 (8.6)	0.00
Gestational Month of Prenatal Vitamin Initiation: 1	107 (7.5)	468 (6.5)	0.04	13 (7.9)	64 (3.9)	0.17	59.3 (6.8)	59.3 (6.8)	0.00
Gestational Month of Prenatal Vitamin Initiation: 2	366 (25.6)	1,711 (23.6)	0.05	31 (18.9)	188 (11.5)	0.21	216.8 (24.8)	216.8 (24.8)	0.00
Gestational Month of Prenatal Vitamin Initiation: 3	225 (15.7)	946 (13.1)	0.08	12 (7.3)	104 (6.3)	0.04	124.2 (14.2)	124.2 (14.2)	0.00
Gestational Month of Prenatal Vitamin Initiation: 4	130 (9.1)	479 (6.6)	0.09	8 (4.9)	39 (2.4)	0.13	71.3 (8.2)	71.3 (8.2)	0.00
Gestational Month of Prenatal Vitamin Initiation: 5	91 (6.4)	286 (4.0)	0.11	6 (3.7)	25 (1.5)	0.13	52.0 (6.0)	52.0 (6.0)	0.00
Gestational Month of Prenatal Vitamin Initiation: 6	69 (4.8)	197 (2.7)	0.11	0 (0.0)	14 (0.9)	-0.13	30.9 (3.5)	30.9 (3.5)	0.00
Gestational Month of Prenatal Vitamin Initiation: 7	47 (3.3)	166 (2.3)	0.06	0 (0.0)	25 (1.5)	-0.18	25.5 (2.9)	25.5 (2.9)	0.00
Gestational Month of Prenatal Vitamin Initiation: 8	27 (1.9)	98 (1.4)	0.04	1 (0.6)	14 (0.9)	-0.03	14.7 (1.7)	14.7 (1.7)	0.00
Gestational Month of Prenatal Vitamin Initiation: 9	30 (2.1)	224 (3.1)	-0.06	2 (1.2)	31 (1.9)	-0.05	21.6 (2.5)	21.6 (2.5)	0.00
Gestational Month of Prenatal Vitamin Initiation: -1	25 (1.8)	60 (0.8)	0.08	7 (4.3)	16 (1.0)	0.21	10.4 (1.2)	10.4 (1.2)	0.00
Gestational Month of Prenatal Vitamin Initiation: -2	33 (2.3)	113 (1.6)	0.05	8 (4.9)	34 (2.1)	0.15	21.1 (2.4)	21.1 (2.4)	0.00
Gestational Month of Prenatal Vitamin Initiation: -3	69 (4.8)	163 (2.3)	0.14	17 (10.4)	74 (4.5)	0.22	41.1 (4.7)	41.1 (4.7)	0.00
Gestational Month of Prenatal Vitamin Initiation: None	212 (14.8)	2,334 (32.2)	-0.42	59 (36.0)	1,014 (61.8)	-0.53	184.7 (21.1)	184.7 (21.1)	0.00
**Topiramate**									
N Total	730	7,245		306	1,642		831.8	831.8	
Age in Years: <=19	90 (12.3)	1,399 (19.3)	-0.19	3 (1.0)	18 (1.1)	-0.01	80.9 (9.7)	80.9 (9.7)	0.00
Age in Years: 20-24	219 (30.0)	2,612 (36.1)	-0.13	28 (9.2)	149 (9.1)	0.00	209.5 (25.2)	209.5 (25.2)	0.00
Age in Years: 25-29	225 (30.8)	1,941 (26.8)	0.09	87 (28.4)	558 (34.0)	-0.12	253.3 (30.5)	253.3 (30.5)	0.00
Age in Years: 30-34	124 (17.0)	904 (12.5)	0.13	122 (39.9)	606 (36.9)	0.06	185.3 (22.3)	185.3 (22.3)	0.00
Age in Years: 35-39	*	*	0.19	59 (19.3)	250 (15.2)	0.11	93.4 (11.2)	93.4 (11.2)	0.00
Age in Years: >=40	<11	*	-0.03	7 (2.3)	61 (3.7)	-0.08	9.4 (1.1)	9.4 (1.1)	0.00
Race/Ethnicity: Asian or other Pacific Islander	<11	*	-0.01	N/A	N/A	N/A	N/A	N/A	N/A
Race/Ethnicity: Black or African American	*	*	-0.23	N/A	N/A	N/A	N/A	N/A	N/A
Race/Ethnicity: Hispanic or Latino	84 (11.5)	963 (13.3)	-0.05	N/A	N/A	N/A	N/A	N/A	N/A
Race/Ethnicity: Unknown or Other	33 (4.5)	340 (4.7)	-0.01	N/A	N/A	N/A	N/A	N/A	N/A
Race/Ethnicity: White	461 (63.2)	3,729 (51.5)	0.24	N/A	N/A	N/A	N/A	N/A	N/A
US Region: Midwest	261 (35.8)	2,598 (35.9)	0.00	72 (23.5)	402 (24.5)	-0.02	270.5 (32.5)	270.5 (32.5)	0.00
US Region: Northeast	123 (16.9)	1,194 (16.5)	0.01	40 (13.1)	270 (16.4)	-0.10	132.5 (15.9)	132.5 (15.9)	0.00
US Region: South	236 (32.3)	2,202 (30.4)	0.04	141 (46.1)	692 (42.1)	0.08	295.9 (35.6)	295.9 (35.6)	0.00
US Region: Unknown	0	0	0.00	5 (1.6)	43 (2.6)	-0.07	4.2 (0.5)	4.2 (0.5)	0.00
US Region: West	110 (15.1)	1,251 (17.3)	-0.06	48 (15.7)	235 (14.3)	0.04	128.6 (15.5)	128.6 (15.5)	0.00
Year of Delivery: 2000	*	<11	-0.02	0 (0.0)	0 (0.0)	0.00	0.0 (0.0)	0.0 (0.0)	0.00
Year of Delivery: 2001	*	*	-0.04	0 (0.0)	0 (0.0)	0.00	9.9 (1.2)	9.9 (1.2)	0.00
Year of Delivery: 2002	12 (1.6)	227 (3.1)	-0.10	0 (0.0)	0 (0.0)	0.00	10.5 (1.3)	10.5 (1.3)	0.00
Year of Delivery: 2003	25 (3.4)	262 (3.6)	-0.01	0 (0.0)	0 (0.0)	0.00	21.0 (2.5)	21.0 (2.5)	0.00
Year of Delivery: 2004	38 (5.2)	368 (5.1)	0.01	9 (2.9)	27 (1.6)	0.09	39.0 (4.7)	39.0 (4.7)	0.00
Year of Delivery: 2005	36 (4.9)	329 (4.5)	0.02	11 (3.6)	43 (2.6)	0.06	38.0 (4.6)	38.0 (4.6)	0.00
Year of Delivery: 2006	56 (7.7)	347 (4.8)	0.12	9 (2.9)	55 (3.4)	-0.02	50.3 (6.0)	50.3 (6.0)	0.00
Year of Delivery: 2007	44 (6.0)	306 (4.2)	0.08	19 (6.2)	61 (3.7)	0.11	47.9 (5.8)	47.9 (5.8)	0.00
Year of Delivery: 2008	39 (5.3)	330 (4.6)	0.04	19 (6.2)	84 (5.1)	0.05	47.2 (5.7)	47.2 (5.7)	0.00
Year of Delivery: 2009	52 (7.1)	370 (5.1)	0.08	36 (11.8)	132 (8.0)	0.12	69.8 (8.4)	69.8 (8.4)	0.00
Year of Delivery: 2010	73 (10.0)	484 (6.7)	0.12	41 (13.4)	147 (9.0)	0.14	86.1 (10.4)	86.1 (10.4)	0.00

	Unadjusted, N (column %)						PS Overlap-Weighted, N (column %)		
	MAX/TAF			MarketScan			Both Cohorts Combined		
**Variable**	**Exposed**	**Unexposed**	**SMD**	**Exposed**	**Unexposed**	**SMD**	**Exposed**	**Unexposed**	**SMD**
Year of Delivery: 2011	63 (8.6)	520 (7.2)	0.05	38 (12.4)	147 (9.0)	0.11	78.0 (9.4)	78.0 (9.4)	0.00
Year of Delivery: 2012	51 (7.0)	541 (7.5)	-0.02	28 (9.2)	156 (9.5)	-0.01	60.4 (7.3)	63.3 (7.6)	0.01
Year of Delivery: 2013	53 (7.3)	625 (8.6)	-0.05	17 (5.6)	107 (6.5)	-0.04	58.8 (7.1)	58.8 (7.1)	0.00
Year of Delivery: 2014	32 (4.4)	480 (6.6)	-0.10	23 (7.5)	102 (6.2)	0.05	44.7 (5.4)	44.7 (5.4)	0.00
Year of Delivery: 2015	32 (4.4)	383 (5.3)	-0.04	11 (3.6)	103 (6.3)	-0.12	36.4 (4.4)	36.4 (4.4)	0.00
Year of Delivery: 2016	35 (4.8)	507 (7.0)	-0.09	11 (3.6)	105 (6.4)	-0.13	37.6 (4.5)	37.6 (4.5)	0.00
Year of Delivery: 2017	49 (6.7)	594 (8.2)	-0.06	9 (2.9)	84 (5.1)	-0.11	48.3 (5.8)	48.3 (5.8)	0.00
Year of Delivery: 2018	29 (4.0)	425 (5.9)	-0.09	8 (2.6)	71 (4.3)	-0.09	31.9 (3.8)	31.9 (3.8)	0.00
Year of Delivery: 2019	0	0	0.00	4 (1.3)	85 (5.2)	-0.22	3.6 (0.4)	3.6 (0.4)	0.00
Year of Delivery: 2020	0	0	0.00	8 (2.6)	74 (4.5)	-0.10	6.1 (0.7)	6.1 (0.7)	0.00
N MH Diagnoses: 0	422 (57.8)	4,225 (58.3)	-0.01	250 (81.7)	1,224 (74.5)	0.17	539.7 (64.9)	539.7 (64.9)	0.00
N MH Diagnoses: 1	107 (14.7)	1,137 (15.7)	-0.03	27 (8.8)	173 (10.5)	-0.06	110.2 (13.3)	110.2 (13.3)	0.00
N MH Diagnoses: 2-3	112 (15.3)	1,047 (14.5)	0.03	23 (7.5)	144 (8.8)	-0.05	106.4 (12.8)	106.4 (12.8)	0.00
N MH Diagnoses: >=4	89 (12.2)	836 (11.5)	0.02	6 (2.0)	101 (6.2)	-0.21	75.5 (9.1)	75.5 (9.1)	0.00
N ED Visits: 0	647 (88.6)	6,204 (85.6)	0.09	303 (99.0)	1,552 (94.5)	0.26	759.7 (91.3)	759.7 (91.3)	0.00
N ED Visits: 1	65 (8.9)	704 (9.7)	-0.03	2 (0.7)	58 (3.5)	-0.20	55.3 (6.7)	55.3 (6.7)	0.00
N ED Visits: 2-3	*	*	-0.11	0 (0.0)	28 (1.7)	-0.19	12.7 (1.5)	12.7 (1.5)	0.00
N ED Visits: >=4	<11	*	-0.05	1 (0.3)	4 (0.2)	0.02	4.0 (0.5)	4.0 (0.5)	0.00
N MH Hospitalizations: 0	689 (94.4)	6,749 (93.2)	0.05	301 (98.4)	1,609 (98.0)	0.03	795.4 (95.6)	795.4 (95.6)	0.00
N MH Hospitalizations: 1	29 (4.0)	384 (5.3)	-0.06	3 (1.0)	26 (1.6)	-0.05	25.7 (3.1)	25.7 (3.1)	0.00
N MH Hospitalizations: 2-3	*	*	0.02	2 (0.7)	7 (0.4)	0.03	10.0 (1.2)	10.0 (1.2)	0.00
N MH Hospitalizations: >=4	<11	*	-0.04	0 (0.0)	0 (0.0)	0.00	0.8 (0.1)	0.8 (0.1)	0.00
N Outpatient Visits: <=3	50 (6.9)	1,012 (14.0)	-0.23	9 (2.9)	99 (6.0)	-0.15	53.7 (6.5)	53.7 (6.5)	0.00
N Outpatient Visits: 4-6	87 (11.9)	1,299 (17.9)	-0.17	62 (20.3)	336 (20.5)	0.00	124.8 (15.0)	124.8 (15.0)	0.00
N Outpatient Visits: 7-10	172 (23.6)	1,788 (24.7)	-0.03	99 (32.4)	482 (29.4)	0.06	219.9 (26.4)	219.9 (26.4)	0.00
N Outpatient Visits: >=11	421 (57.7)	3,146 (43.4)	0.29	136 (44.4)	725 (44.2)	0.01	433.3 (52.1)	433.3 (52.1)	0.00
N Psychotropic Medications (Except ASMs), Mean (SD)	0.0 (0.2)	0.0 (0.2)	0.02	0.0 (0.1)	0.0 (0.2)	-0.08	0.0 (0.2)	0.0 (0.2)	0.00
Alcohol Use Disorder	18 (2.5)	277 (3.8)	-0.08	1 (0.3)	21 (1.3)	-0.11	16.4 (2.0)	16.4 (2.0)	0.00
Smoking	105 (14.4)	1,475 (20.4)	-0.16	7 (2.3)	96 (5.9)	-0.18	96.2 (11.6)	96.2 (11.6)	0.00
Substance Use Disorder	70 (9.6)	1,007 (13.9)	-0.13	9 (2.9)	43 (2.6)	0.02	66.4 (8.0)	66.4 (8.0)	0.00
ADHD	18 (2.5)	191 (2.6)	-0.01	3 (1.0)	40 (2.4)	-0.11	17.8 (2.1)	17.8 (2.1)	0.00
Adjustment Disorder	12 (1.6)	125 (1.7)	-0.01	3 (1.0)	8 (0.5)	0.06	12.5 (1.5)	12.5 (1.5)	0.00
Anxiety	131 (18.0)	1,489 (20.6)	-0.07	25 (8.2)	251 (15.3)	-0.22	129.5 (15.6)	129.5 (15.6)	0.00
Behavioral Disorder	16 (2.2)	131 (1.8)	0.03	1 (0.3)	5 (0.3)	0.00	14.2 (1.7)	14.2 (1.7)	0.00
Bipolar Disorder	81 (11.1)	610 (8.4)	0.09	6 (2.0)	43 (2.6)	-0.04	68.2 (8.2)	68.2 (8.2)	0.00
Chronic Pain	26 (3.6)	173 (2.4)	0.07	1 (0.3)	29 (1.8)	-0.14	19.6 (2.4)	19.6 (2.4)	0.00
Chronic Fatigue	87 (11.9)	695 (9.6)	0.08	26 (8.5)	210 (12.8)	-0.14	88.8 (10.7)	88.8 (10.7)	0.00
Depression	174 (23.8)	1,660 (22.9)	0.02	45 (14.7)	234 (14.3)	0.01	173.0 (20.8)	173.0 (20.8)	0.00
Migraine/Headache	205 (28.1)	991 (13.7)	0.36	76 (24.8)	316 (19.2)	0.14	206.7 (24.9)	206.7 (24.9)	0.00
Neuromuscular Disorder	21 (2.9)	312 (4.3)	-0.08	3 (1.0)	73 (4.5)	-0.21	21.4 (2.6)	21.4 (2.6)	0.00
Neuropathic Pain	56 (7.7)	463 (6.4)	0.05	15 (4.9)	169 (10.3)	-0.20	58.5 (7.0)	58.5 (7.0)	0.00
Other MH Disorders	24 (3.3)	358 (4.9)	-0.08	7 (2.3)	50 (3.1)	-0.05	25.5 (3.1)	25.5 (3.1)	0.00
Other Developmental Disorders	15 (2.1)	82 (1.1)	0.07	0 (0.0)	4 (0.2)	-0.07	11.3 (1.4)	11.3 (1.4)	0.00
Personality Disorder	11 (1.5)	126 (1.7)	-0.02	0 (0.0)	7 (0.4)	-0.09	9.3 (1.1)	9.3 (1.1)	0.00
Psychosis	20 (2.7)	186 (2.6)	0.01	2 (0.7)	21 (1.3)	-0.06	18.6 (2.2)	18.6 (2.2)	0.00

	Unadjusted, N (column %)						PS Overlap-Weighted, N (column %)		
	MAX/TAF			MarketScan			Both Cohorts Combined		
**Variable**	**Exposed**	**Unexposed**	**SMD**	**Exposed**	**Unexposed**	**SMD**	**Exposed**	**Unexposed**	**SMD**
Schizophrenia/Schizoaffective Disorder	<11	*	0.01	0 (0.0)	6 (0.4)	-0.09	7.9 (1.0)	7.9 (1.0)	0.00
Sleep Disorder	50 (6.9)	282 (3.9)	0.13	10 (3.3)	98 (6.0)	-0.13	47.4 (5.7)	47.4 (5.7)	0.00
Asthma	121 (16.6)	1,144 (15.8)	0.02	15 (4.9)	140 (8.5)	-0.15	111.4 (13.4)	111.4 (13.4)	0.00
Autoimmune Disorder	22 (3.0)	206 (2.8)	0.01	11 (3.6)	96 (5.9)	-0.11	26.8 (3.2)	26.8 (3.2)	0.00
Hyperemesis/NVP	243 (33.3)	2,529 (34.9)	-0.03	56 (18.3)	351 (21.4)	-0.08	242.2 (29.1)	242.2 (29.1)	0.00
Influenca Infection	13 (1.8)	180 (2.5)	-0.05	8 (2.6)	35 (2.1)	0.03	16.3 (2.0)	16.3 (2.0)	0.00
Obstetric Comorbidity Score: 0	268 (36.7)	2,642 (36.5)	0.01	121 (39.5)	634 (38.6)	0.02	313.9 (37.7)	313.9 (37.7)	0.00
Obstetric Comorbidity Score: 1	177 (24.3)	1,590 (22.0)	0.05	87 (28.4)	369 (22.5)	0.14	207.2 (24.9)	207.2 (24.9)	0.00
Obstetric Comorbidity Score: 2	86 (11.8)	1,133 (15.6)	-0.11	46 (15.0)	265 (16.1)	-0.03	107.4 (12.9)	107.4 (12.9)	0.00
Obstetric Comorbidity Score: >=3	199 (27.3)	1,880 (26.0)	0.03	52 (17.0)	374 (22.8)	-0.15	203.3 (24.4)	203.3 (24.4)	0.00
Overweight/Obesity	94 (12.9)	811 (11.2)	0.05	32 (10.5)	185 (11.3)	-0.03	99.4 (12.0)	99.4 (12.0)	0.00
Pre-gestational Diabetes	20 (2.7)	241 (3.3)	-0.03	2 (0.7)	43 (2.6)	-0.16	18.4 (2.2)	18.4 (2.2)	0.00
Pre-gestational Hypertension	75 (10.3)	835 (11.5)	-0.04	27 (8.8)	170 (10.4)	-0.05	83.6 (10.1)	83.6 (10.1)	0.00
Poor Nutrition	31 (4.3)	247 (3.4)	0.04	16 (5.2)	123 (7.5)	-0.09	37.3 (4.5)	37.3 (4.5)	0.00
Renal Disease	<11	*	-0.05	3 (1.0)	20 (1.2)	-0.02	6.7 (0.8)	6.7 (0.8)	0.00
TORCH Infection	26 (3.6)	277 (3.8)	-0.01	5 (1.6)	36 (2.2)	-0.04	25.8 (3.1)	25.8 (3.1)	0.00
Other Sexually Transmitted Diseases	69 (9.5)	812 (11.2)	-0.06	10 (3.3)	56 (3.4)	-0.01	65.5 (7.9)	65.5 (7.9)	0.00
Prenatal Folic Acid Supplementation	523 (71.6)	4,000 (55.2)	0.35	189 (61.8)	527 (32.1)	0.62	544.0 (65.4)	544.0 (65.4)	0.00
Antidepressants	223 (30.6)	1,452 (20.0)	0.24	63 (20.6)	277 (16.9)	0.10	218.2 (26.2)	218.2 (26.2)	0.00
Antidiabetics	20 (2.7)	86 (1.2)	0.11	11 (3.6)	52 (3.2)	0.02	21.5 (2.6)	21.5 (2.6)	0.00
Antihypertensives	79 (10.8)	450 (6.2)	0.17	13 (4.3)	104 (6.3)	-0.09	70.5 (8.5)	70.5 (8.5)	0.00
Antipsychotics	74 (10.1)	430 (5.9)	0.15	6 (2.0)	43 (2.6)	-0.04	60.3 (7.3)	60.3 (7.3)	0.00
Anxiolytics/Hypnotics/Other Sedatives	86 (11.8)	742 (10.2)	0.05	19 (6.2)	87 (5.3)	0.04	83.1 (10.0)	83.1 (10.0)	0.00
Barbiturates	54 (7.4)	299 (4.1)	0.14	23 (7.5)	56 (3.4)	0.18	51.6 (6.2)	51.6 (6.2)	0.00
Benzodiazepines	129 (17.7)	760 (10.5)	0.21	38 (12.4)	160 (9.7)	0.09	124.0 (14.9)	124.0 (14.9)	0.00
Corticosteroids	179 (24.5)	1,221 (16.9)	0.19	52 (17.0)	273 (16.6)	0.01	177.0 (21.3)	177.0 (21.3)	0.00
Insulin	13 (1.8)	131 (1.8)	0.00	3 (1.0)	26 (1.6)	-0.05	12.2 (1.5)	12.2 (1.5)	0.00
Lithium	<11	*	0.08	0 (0.0)	2 (0.1)	-0.05	5.7 (0.7)	5.7 (0.7)	0.00
NSAIDs	190 (26.0)	1,747 (24.1)	0.04	23 (7.5)	148 (9.0)	-0.05	171.8 (20.7)	171.8 (20.7)	0.00
Opioid Agonist Therapy	<11	*	-0.04	0 (0.0)	8 (0.5)	-0.10	4.6 (0.6)	4.6 (0.6)	0.00
Prescription Opioids	303 (41.5)	2,514 (34.7)	0.14	78 (25.5)	296 (18.0)	0.18	296.1 (35.6)	296.1 (35.6)	0.00
Progestins	46 (6.3)	459 (6.3)	0.00	36 (11.8)	231 (14.1)	-0.07	65.0 (7.8)	65.0 (7.8)	0.00
Psychostimulants	13 (1.8)	131 (1.8)	0.00	6 (2.0)	44 (2.7)	-0.05	16.3 (2.0)	16.3 (2.0)	0.00
Teratogens, Known	<11	*	0.09	4 (1.3)	8 (0.5)	0.09	9.3 (1.1)	9.3 (1.1)	0.00
Teratogens, Suspected	129 (17.7)	1,123 (15.5)	0.06	39 (12.8)	181 (11.0)	0.05	132.4 (15.9)	132.4 (15.9)	0.00
Triptans	70 (9.6)	183 (2.5)	0.30	21 (6.9)	60 (3.7)	0.14	59.0 (7.1)	59.0 (7.1)	0.00
Adequacy of Prenatal Care Utilization: Inadequate	287 (39.3)	2,867 (39.6)	-0.01	168 (54.9)	860 (52.4)	0.05	362.1 (43.5)	362.1 (43.5)	0.00
Adequacy of Prenatal Care Utilization: Intermediate	128 (17.5)	1,100 (15.2)	0.06	78 (25.5)	358 (21.8)	0.09	159.4 (19.2)	159.4 (19.2)	0.00
Adequacy of Prenatal Care Utilization: Adequate	110 (15.1)	1,121 (15.5)	-0.01	28 (9.2)	208 (12.7)	-0.11	112.6 (13.5)	112.6 (13.5)	0.00
Adequacy of Prenatal Care Utilization: Adequate Plus	205 (28.1)	2,157 (29.8)	-0.04	32 (10.5)	216 (13.2)	-0.08	197.7 (23.8)	197.7 (23.8)	0.00
Multiple Gestation	18 (2.5)	165 (2.3)	0.01	10 (3.3)	43 (2.6)	0.04	21.5 (2.6)	21.5 (2.6)	0.00
Gestational Month of Prenatal Care Initiation: 1	46 (6.3)	390 (5.4)	0.04	21 (6.9)	110 (6.7)	0.01	53.0 (6.4)	53.0 (6.4)	0.00
Gestational Month of Prenatal Care Initiation: 2	286 (39.2)	2,637 (36.4)	0.06	153 (50.0)	714 (43.5)	0.13	345.8 (41.6)	345.8 (41.6)	0.00
Gestational Month of Prenatal Care Initiation: 3	173 (23.7)	1,788 (24.7)	-0.02	72 (23.5)	429 (26.1)	-0.06	199.3 (24.0)	199.3 (24.0)	0.00
Gestational Month of Prenatal Care Initiation: 4	73 (10.0)	781 (10.8)	-0.03	16 (5.2)	100 (6.1)	-0.04	73.5 (8.8)	73.5 (8.8)	0.00

	Unadjusted, N (column %)						PS Overlap-Weighted, N (column %)		
	MAX/TAF			MarketScan			Both Cohorts Combined		
**Variable**	**Exposed**	**Unexposed**	**SMD**	**Exposed**	**Unexposed**	**SMD**	**Exposed**	**Unexposed**	**SMD**
Gestational Month of Prenatal Care Initiation: 5	45 (6.2)	486 (6.7)	-0.02	11 (3.6)	63 (3.8)	-0.01	46.1 (5.5)	46.1 (5.5)	0.00
Gestational Month of Prenatal Care Initiation: 6	23 (3.2)	277 (3.8)	-0.04	1 (0.3)	30 (1.8)	-0.15	20.3 (2.4)	20.3 (2.4)	0.00
Gestational Month of Prenatal Care Initiation: 7	21 (2.9)	197 (2.7)	0.01	6 (2.0)	30 (1.8)	0.01	23.1 (2.8)	23.1 (2.8)	0.00
Gestational Month of Prenatal Care Initiation: 8	13 (1.8)	142 (2.0)	-0.01	3 (1.0)	19 (1.2)	-0.02	12.6 (1.5)	12.6 (1.5)	0.00
Gestational Month of Prenatal Care Initiation: 9	11 (1.5)	134 (1.9)	-0.03	4 (1.3)	17 (1.0)	0.03	11.8 (1.4)	11.8 (1.4)	0.00
Gestational Month of Prenatal Care Initiation: None	39 (5.3)	413 (5.7)	-0.02	19 (6.2)	130 (7.9)	-0.07	46.3 (5.6)	46.3 (5.6)	0.00
Gestational Month of Prenatal Vitamin Initiation: 1	74 (10.1)	468 (6.5)	0.13	27 (8.8)	64 (3.9)	0.20	77.0 (9.3)	77.0 (9.3)	0.00
Gestational Month of Prenatal Vitamin Initiation: 2	192 (26.3)	1,711 (23.6)	0.06	51 (16.7)	188 (11.5)	0.15	197.8 (23.8)	197.8 (23.8)	0.00
Gestational Month of Prenatal Vitamin Initiation: 3	79 (10.8)	946 (13.1)	-0.07	20 (6.5)	104 (6.3)	0.01	84.6 (10.2)	84.6 (10.2)	0.00
Gestational Month of Prenatal Vitamin Initiation: 4	50 (6.9)	479 (6.6)	0.01	13 (4.3)	39 (2.4)	0.10	50.3 (6.1)	50.3 (6.1)	0.00
Gestational Month of Prenatal Vitamin Initiation: 5	23 (3.2)	286 (4.0)	-0.04	1 (0.3)	25 (1.5)	-0.13	21.3 (2.6)	21.3 (2.6)	0.00
Gestational Month of Prenatal Vitamin Initiation: 6	17 (2.3)	197 (2.7)	-0.02	3 (1.0)	14 (0.9)	0.01	17.4 (2.1)	17.4 (2.1)	0.00
Gestational Month of Prenatal Vitamin Initiation: 7	<11	*	-0.07	1 (0.3)	25 (1.5)	-0.13	10.2 (1.2)	10.2 (1.2)	0.00
Gestational Month of Prenatal Vitamin Initiation: 8	<11	*	-0.02	0 (0.0)	14 (0.9)	-0.13	7.5 (0.9)	7.5 (0.9)	0.00
Gestational Month of Prenatal Vitamin Initiation: 9	21 (2.9)	224 (3.1)	-0.01	1 (0.3)	31 (1.9)	-0.15	19.3 (2.3)	19.3 (2.3)	0.00
Gestational Month of Prenatal Vitamin Initiation: -1	20 (2.7)	60 (0.8)	0.14	18 (5.9)	16 (1.0)	0.27	22.6 (2.7)	22.6 (2.7)	0.00
Gestational Month of Prenatal Vitamin Initiation: -2	36 (4.9)	113 (1.6)	0.19	27 (8.8)	34 (2.1)	0.30	40.5 (4.9)	40.5 (4.9)	0.00
Gestational Month of Prenatal Vitamin Initiation: -3	66 (9.0)	163 (2.3)	0.30	33 (10.8)	74 (4.5)	0.24	65.7 (7.9)	65.7 (7.9)	0.00
Gestational Month of Prenatal Vitamin Initiation: None	134 (18.4)	2,334 (32.2)	-0.32	111 (36.3)	1,014 (61.8)	-0.53	217.5 (26.2)	217.5 (26.2)	0.00
**Oxcarbazepine**									
N Total	572	7,245		273	1,642		686.7	686.7	
Age in Years: <=19	92 (16.1)	1,399 (19.3)	-0.08	0 (0.0)	18 (1.1)	-0.15	82.3 (12.0)	82.3 (12.0)	0.00
Age in Years: 20-24	179 (31.3)	2,612 (36.1)	-0.10	13 (4.8)	149 (9.1)	-0.17	168.5 (24.5)	168.5 (24.5)	0.00
Age in Years: 25-29	171 (29.9)	1,941 (26.8)	0.07	82 (30.0)	558 (34.0)	-0.08	201.8 (29.4)	201.8 (29.4)	0.00
Age in Years: 30-34	97 (17.0)	904 (12.5)	0.13	104 (38.1)	606 (36.9)	0.02	153.7 (22.4)	153.7 (22.4)	0.00
Age in Years: 35-39	*	*	0.01	61 (22.3)	250 (15.2)	0.18	65.0 (9.5)	65.0 (9.5)	0.00
Age in Years: >=40	<11	*	0.02	13 (4.8)	61 (3.7)	0.05	15.5 (2.3)	15.5 (2.3)	0.00
Race/Ethnicity: Asian or other Pacific Islander	<11	*	0.05	N/A	N/A	N/A	N/A	N/A	N/A
Race/Ethnicity: Black or African American	*	*	-0.21	N/A	N/A	N/A	N/A	N/A	N/A
Race/Ethnicity: Hispanic or Latino	106 (18.5)	963 (13.3)	0.14	N/A	N/A	N/A	N/A	N/A	N/A
Race/Ethnicity: Unknown or Other	18 (3.2)	340 (4.7)	-0.08	N/A	N/A	N/A	N/A	N/A	N/A
Race/Ethnicity: White	323 (56.5)	3,729 (51.5)	0.10	N/A	N/A	N/A	N/A	N/A	N/A
US Region: Midwest	227 (39.7)	2,598 (35.9)	0.08	72 (26.4)	402 (24.5)	0.04	242.2 (35.3)	242.2 (35.3)	0.00
US Region: Northeast	94 (16.4)	1,194 (16.5)	0.00	49 (18.0)	270 (16.4)	0.04	113.8 (16.6)	113.8 (16.6)	0.00
US Region: South	161 (28.2)	2,202 (30.4)	-0.05	107 (39.2)	692 (42.1)	-0.06	219.7 (32.0)	219.7 (32.0)	0.00
US Region: Unknown	0	0	0.00	10 (3.7)	43 (2.6)	0.06	6.3 (0.9)	6.3 (0.9)	0.00
US Region: West	90 (15.7)	1,251 (17.3)	-0.04	35 (12.8)	235 (14.3)	-0.04	104.7 (15.3)	104.7 (15.3)	0.00
Year of Delivery: 2000	*	<11	-0.02	0 (0.0)	0 (0.0)	0.00	0.0 (0.0)	0.0 (0.0)	0.00
Year of Delivery: 2001	<11	*	-0.13	0 (0.0)	0 (0.0)	0.00	2.8 (0.4)	2.8 (0.4)	0.00
Year of Delivery: 2002	12 (2.1)	227 (3.1)	-0.06	0 (0.0)	0 (0.0)	0.00	11.1 (1.6)	11.1 (1.6)	0.00
Year of Delivery: 2003	28 (4.9)	262 (3.6)	0.06	0 (0.0)	0 (0.0)	0.00	22.7 (3.3)	22.7 (3.3)	0.00
Year of Delivery: 2004	34 (5.9)	368 (5.1)	0.04	6 (2.2)	27 (1.6)	0.04	34.7 (5.1)	34.7 (5.1)	0.00
Year of Delivery: 2005	36 (6.3)	329 (4.5)	0.08	10 (3.7)	43 (2.6)	0.06	35.3 (5.1)	35.3 (5.1)	0.00
Year of Delivery: 2006	36 (6.3)	347 (4.8)	0.07	8 (2.9)	55 (3.4)	-0.02	34.8 (5.1)	34.8 (5.1)	0.00
Year of Delivery: 2007	38 (6.6)	306 (4.2)	0.11	14 (5.1)	61 (3.7)	0.07	40.9 (6.0)	40.9 (6.0)	0.00

	Unadjusted, N (column %)						PS Overlap-Weighted, N (column %)		
	MAX/TAF			MarketScan			Both Cohorts Combined		
**Variable**	**Exposed**	**Unexposed**	**SMD**	**Exposed**	**Unexposed**	**SMD**	**Exposed**	**Unexposed**	**SMD**
Year of Delivery: 2008	25 (4.4)	330 (4.6)	-0.01	23 (8.4)	84 (5.1)	0.13	37.5 (5.5)	37.5 (5.5)	0.00
Year of Delivery: 2009	30 (5.2)	370 (5.1)	0.01	24 (8.8)	132 (8.0)	0.03	43.0 (6.3)	43.0 (6.3)	0.00
Year of Delivery: 2010	36 (6.3)	484 (6.7)	-0.02	32 (11.7)	147 (9.0)	0.09	56.6 (8.2)	56.6 (8.2)	0.00
Year of Delivery: 2011	41 (7.2)	520 (7.2)	0.00	21 (7.7)	147 (9.0)	-0.05	50.6 (7.4)	50.6 (7.4)	0.00
Year of Delivery: 2012	45 (7.9)	541 (7.5)	0.02	27 (9.9)	156 (9.5)	0.01	56.7 (8.3)	58.8 (8.6)	0.01
Year of Delivery: 2013	38 (6.6)	625 (8.6)	-0.07	18 (6.6)	107 (6.5)	0.00	46.8 (6.8)	46.8 (6.8)	0.00
Year of Delivery: 2014	33 (5.8)	480 (6.6)	-0.04	17 (6.2)	102 (6.2)	0.00	40.5 (5.9)	40.5 (5.9)	0.00
Year of Delivery: 2015	22 (3.9)	383 (5.3)	-0.07	13 (4.8)	103 (6.3)	-0.07	28.6 (4.2)	28.6 (4.2)	0.00
Year of Delivery: 2016	36 (6.3)	507 (7.0)	-0.03	16 (5.9)	105 (6.4)	-0.02	43.1 (6.3)	43.1 (6.3)	0.00
Year of Delivery: 2017	39 (6.8)	594 (8.2)	-0.05	16 (5.9)	84 (5.1)	0.03	46.2 (6.7)	46.2 (6.7)	0.00
Year of Delivery: 2018	40 (7.0)	425 (5.9)	0.05	7 (2.6)	71 (4.3)	-0.10	37.7 (5.5)	37.7 (5.5)	0.00
Year of Delivery: 2019	0	0	0.00	6 (2.2)	85 (5.2)	-0.16	4.6 (0.7)	4.6 (0.7)	0.00
Year of Delivery: 2020	0	0	0.00	10 (3.7)	74 (4.5)	-0.04	6.9 (1.0)	6.9 (1.0)	0.00
N MH Diagnoses: 0	332 (58.0)	4,225 (58.3)	-0.01	227 (83.2)	1,224 (74.5)	0.21	449.3 (65.4)	449.3 (65.4)	0.00
N MH Diagnoses: 1	85 (14.9)	1,137 (15.7)	-0.02	25 (9.2)	173 (10.5)	-0.05	92.8 (13.5)	92.8 (13.5)	0.00
N MH Diagnoses: 2-3	84 (14.7)	1,047 (14.5)	0.01	16 (5.9)	144 (8.8)	-0.11	82.0 (11.9)	82.0 (11.9)	0.00
N MH Diagnoses: >=4	71 (12.4)	836 (11.5)	0.03	5 (1.8)	101 (6.2)	-0.22	62.6 (9.1)	62.6 (9.1)	0.00
N ED Visits: 0	510 (89.2)	6,204 (85.6)	0.11	268 (98.2)	1,552 (94.5)	0.20	630.0 (91.7)	630.0 (91.7)	0.00
N ED Visits: 1	44 (7.7)	704 (9.7)	-0.07	5 (1.8)	58 (3.5)	-0.11	41.2 (6.0)	41.2 (6.0)	0.00
N ED Visits: 2-3	*	*	-0.10	0 (0.0)	28 (1.7)	-0.19	10.8 (1.6)	10.8 (1.6)	0.00
N ED Visits: >=4	<11	*	0.01	0 (0.0)	4 (0.2)	-0.07	4.7 (0.7)	4.7 (0.7)	0.00
N MH Hospitalizations: 0	538 (94.1)	6,749 (93.2)	0.04	270 (98.9)	1,609 (98.0)	0.07	656.4 (95.6)	656.4 (95.6)	0.00
N MH Hospitalizations: 1	27 (4.7)	384 (5.3)	-0.03	3 (1.1)	26 (1.6)	-0.04	24.9 (3.6)	24.9 (3.6)	0.00
N MH Hospitalizations: 2-3	<11	*	0.00	0 (0.0)	7 (0.4)	-0.09	5.4 (0.8)	5.4 (0.8)	0.00
N MH Hospitalizations: >=4	*	*	-0.08	0 (0.0)	0 (0.0)	0.00	0.0 (0.0)	0.0 (0.0)	0.00
N Outpatient Visits: <=3	54 (9.4)	1,012 (14.0)	-0.14	15 (5.5)	99 (6.0)	-0.02	61.0 (8.9)	61.0 (8.9)	0.00
N Outpatient Visits: 4-6	79 (13.8)	1,299 (17.9)	-0.11	49 (18.0)	336 (20.5)	-0.06	107.8 (15.7)	107.8 (15.7)	0.00
N Outpatient Visits: 7-10	143 (25.0)	1,788 (24.7)	0.01	85 (31.1)	482 (29.4)	0.04	184.1 (26.8)	184.1 (26.8)	0.00
N Outpatient Visits: >=11	296 (51.8)	3,146 (43.4)	0.17	124 (45.4)	725 (44.2)	0.03	333.9 (48.6)	333.9 (48.6)	0.00
N Psychotropic Medications (Except ASMs), Mean (SD)	0.0 (0.2)	0.0 (0.2)	0.08	0.0 (0.2)	0.0 (0.2)	-0.11	0.0 (0.2)	0.0 (0.2)	0.00
Alcohol Use Disorder	14 (2.5)	277 (3.8)	-0.08	0 (0.0)	21 (1.3)	-0.16	12.9 (1.9)	12.9 (1.9)	0.00
Smoking	98 (17.1)	1,475 (20.4)	-0.08	6 (2.2)	96 (5.9)	-0.19	91.8 (13.4)	91.8 (13.4)	0.00
Substance Use Disorder	54 (9.4)	1,007 (13.9)	-0.14	8 (2.9)	43 (2.6)	0.02	52.8 (7.7)	52.8 (7.7)	0.00
ADHD	16 (2.8)	191 (2.6)	0.01	2 (0.7)	40 (2.4)	-0.14	14.7 (2.1)	14.7 (2.1)	0.00
Adjustment Disorder	11 (1.9)	125 (1.7)	0.01	1 (0.4)	8 (0.5)	-0.02	9.9 (1.4)	9.9 (1.4)	0.00
Anxiety	111 (19.4)	1,489 (20.6)	-0.03	19 (7.0)	251 (15.3)	-0.27	108.5 (15.8)	108.5 (15.8)	0.00
Behavioral Disorder	<11	*	0.00	0 (0.0)	5 (0.3)	-0.08	8.5 (1.2)	8.5 (1.2)	0.00
Bipolar Disorder	84 (14.7)	610 (8.4)	0.20	6 (2.2)	43 (2.6)	-0.03	71.1 (10.4)	71.1 (10.4)	0.00
Chronic Pain	<11	*	-0.10	2 (0.7)	29 (1.8)	-0.09	7.3 (1.1)	7.3 (1.1)	0.00
Chronic Fatigue	44 (7.7)	695 (9.6)	-0.07	28 (10.3)	210 (12.8)	-0.08	60.0 (8.7)	60.0 (8.7)	0.00
Depression	119 (20.8)	1,660 (22.9)	-0.05	26 (9.5)	234 (14.3)	-0.15	122.7 (17.9)	122.7 (17.9)	0.00
Migraine/Headache	88 (15.4)	991 (13.7)	0.05	26 (9.5)	316 (19.2)	-0.28	96.3 (14.0)	96.3 (14.0)	0.00
Neuromuscular Disorder	27 (4.7)	312 (4.3)	0.02	14 (5.1)	73 (4.5)	0.03	32.1 (4.7)	32.1 (4.7)	0.00
Neuropathic Pain	20 (3.5)	463 (6.4)	-0.13	21 (7.7)	169 (10.3)	-0.09	35.2 (5.1)	35.2 (5.1)	0.00
Other MH Disorders	25 (4.4)	358 (4.9)	-0.03	4 (1.5)	50 (3.1)	-0.11	24.1 (3.5)	24.1 (3.5)	0.00

	Unadjusted, N (column %)						PS Overlap-Weighted, N (column %)		
	MAX/TAF			MarketScan			Both Cohorts Combined		
Variable	Exposed	Unexposed	SMD	Exposed	Unexposed	SMD	Exposed	Unexposed	SMD
Other Developmental Disorders	<11	*	0.01	1 (0.4)	4 (0.2)	0.02	6.7 (1.0)	6.7 (1.0)	0.00
Personality Disorder	13 (2.3)	126 (1.7)	0.04	0 (0.0)	7 (0.4)	-0.09	11.0 (1.6)	11.0 (1.6)	0.00
Psychosis	21 (3.7)	186 (2.6)	0.06	2 (0.7)	21 (1.3)	-0.05	18.4 (2.7)	18.4 (2.7)	0.00
Schizophrenia/Schizoaffective Disorder	<11	*	0.00	0 (0.0)	6 (0.4)	-0.09	6.6 (1.0)	6.6 (1.0)	0.00
Sleep Disorder	22 (3.9)	282 (3.9)	0.00	14 (5.1)	98 (6.0)	-0.04	29.9 (4.4)	29.9 (4.4)	0.00
Asthma	67 (11.7)	1,144 (15.8)	-0.12	11 (4.0)	140 (8.5)	-0.19	67.7 (9.9)	67.7 (9.9)	0.00
Autoimmune Disorder	<11	*	-0.11	15 (5.5)	96 (5.9)	-0.02	18.1 (2.6)	18.1 (2.6)	0.00
Hyperemesis/NVP	152 (26.6)	2,529 (34.9)	-0.18	40 (14.7)	351 (21.4)	-0.18	164.3 (23.9)	164.3 (23.9)	0.00
Influenca Infection	11 (1.9)	180 (2.5)	-0.04	6 (2.2)	35 (2.1)	0.00	13.2 (1.9)	13.2 (1.9)	0.00
Obstetric Comorbidity Score: 0	243 (42.5)	2,642 (36.5)	0.12	90 (33.0)	634 (38.6)	-0.12	272.0 (39.6)	272.0 (39.6)	0.00
Obstetric Comorbidity Score: 1	128 (22.4)	1,590 (22.0)	0.01	83 (30.4)	369 (22.5)	0.18	167.3 (24.4)	167.3 (24.4)	0.00
Obstetric Comorbidity Score: 2	71 (12.4)	1,133 (15.6)	-0.09	38 (13.9)	265 (16.1)	-0.06	90.3 (13.2)	90.3 (13.2)	0.00
Obstetric Comorbidity Score: >=3	130 (22.7)	1,880 (26.0)	-0.08	62 (22.7)	374 (22.8)	0.00	157.1 (22.9)	157.1 (22.9)	0.00
Overweight/Obesity	66 (11.5)	811 (11.2)	0.01	30 (11.0)	185 (11.3)	-0.01	80.7 (11.8)	80.7 (11.8)	0.00
Pre-gestational Diabetes	12 (2.1)	241 (3.3)	-0.08	6 (2.2)	43 (2.6)	-0.03	15.6 (2.3)	15.6 (2.3)	0.00
Pre-gestational Hypertension	51 (8.9)	835 (11.5)	-0.09	37 (13.6)	170 (10.4)	0.10	73.1 (10.7)	73.1 (10.7)	0.00
Poor Nutrition	22 (3.9)	247 (3.4)	0.02	18 (6.6)	123 (7.5)	-0.04	31.7 (4.6)	31.7 (4.6)	0.00
Renal Disease	<11	*	-0.03	1 (0.4)	20 (1.2)	-0.10	5.3 (0.8)	5.3 (0.8)	0.00
TORCH Infection	24 (4.2)	277 (3.8)	0.02	11 (4.0)	36 (2.2)	0.11	29.0 (4.2)	29.0 (4.2)	0.00
Other Sexually Transmitted Diseases	48 (8.4)	812 (11.2)	-0.09	6 (2.2)	56 (3.4)	-0.07	47.4 (6.9)	47.4 (6.9)	0.00
Prenatal Folic Acid Supplementation	425 (74.3)	4,000 (55.2)	0.41	174 (63.7)	527 (32.1)	0.67	462.0 (67.3)	462.0 (67.3)	0.00
Antidepressants	154 (26.9)	1,452 (20.0)	0.16	36 (13.2)	277 (16.9)	-0.10	154.4 (22.5)	154.4 (22.5)	0.00
Antidiabetics	<11	*	0.05	10 (3.7)	52 (3.2)	0.03	16.1 (2.3)	16.1 (2.3)	0.00
Antihypertensives	32 (5.6)	450 (6.2)	-0.03	18 (6.6)	104 (6.3)	0.01	40.7 (5.9)	40.7 (5.9)	0.00
Antipsychotics	55 (9.6)	430 (5.9)	0.14	7 (2.6)	43 (2.6)	0.00	48.3 (7.0)	48.3 (7.0)	0.00
Anxiolytics/Hypnotics/Other Sedatives	72 (12.6)	742 (10.2)	0.07	12 (4.4)	87 (5.3)	-0.04	68.2 (9.9)	68.2 (9.9)	0.00
Barbiturates	52 (9.1)	299 (4.1)	0.20	10 (3.7)	56 (3.4)	0.01	46.4 (6.8)	46.4 (6.8)	0.00
Benzodiazepines	91 (15.9)	760 (10.5)	0.16	33 (12.1)	160 (9.7)	0.08	93.3 (13.6)	93.3 (13.6)	0.00
Corticosteroids	121 (21.2)	1,221 (16.9)	0.11	52 (19.1)	273 (16.6)	0.06	135.2 (19.7)	135.2 (19.7)	0.00
Insulin	14 (2.5)	131 (1.8)	0.04	4 (1.5)	26 (1.6)	-0.01	13.4 (2.0)	13.4 (2.0)	0.00
Lithium	<11	*	0.02	1 (0.4)	2 (0.1)	0.05	3.1 (0.5)	3.1 (0.5)	0.00
NSAIDs	139 (24.3)	1,747 (24.1)	0.00	27 (9.9)	148 (9.0)	0.03	138.8 (20.2)	138.8 (20.2)	0.00
Opioid Agonist Therapy	<11	*	-0.02	0 (0.0)	8 (0.5)	-0.10	4.0 (0.6)	4.0 (0.6)	0.00
Prescription Opioids	186 (32.5)	2,514 (34.7)	-0.05	45 (16.5)	296 (18.0)	-0.04	194.4 (28.3)	194.4 (28.3)	0.00
Progestins	42 (7.3)	459 (6.3)	0.04	39 (14.3)	231 (14.1)	0.01	61.8 (9.0)	61.8 (9.0)	0.00
Psychostimulants	19 (3.3)	131 (1.8)	0.10	3 (1.1)	44 (2.7)	-0.12	16.3 (2.4)	16.3 (2.4)	0.00
Teratogens, Known	<11	*	0.05	1 (0.4)	8 (0.5)	-0.02	4.7 (0.7)	4.7 (0.7)	0.00
Teratogens, Suspected	80 (14.0)	1,123 (15.5)	-0.04	30 (11.0)	181 (11.0)	0.00	90.5 (13.2)	90.5 (13.2)	0.00
Triptans	28 (4.9)	183 (2.5)	0.13	10 (3.7)	60 (3.7)	0.00	28.7 (4.2)	28.7 (4.2)	0.00
Adequacy of Prenatal Care Utilization: Inadequate	195 (34.1)	2,867 (39.6)	-0.11	143 (52.4)	860 (52.4)	0.00	277.3 (40.4)	277.3 (40.4)	0.00
Adequacy of Prenatal Care Utilization: Intermediate	84 (14.7)	1,100 (15.2)	-0.01	51 (18.7)	358 (21.8)	-0.08	109.4 (15.9)	109.4 (15.9)	0.00
Adequacy of Prenatal Care Utilization: Adequate	81 (14.2)	1,121 (15.5)	-0.04	34 (12.5)	208 (12.7)	-0.01	95.0 (13.8)	95.0 (13.8)	0.00
Adequacy of Prenatal Care Utilization: Adequate Plus	212 (37.1)	2,157 (29.8)	0.15	45 (16.5)	216 (13.2)	0.09	205.1 (29.9)	205.1 (29.9)	0.00
Multiple Gestation	<11	*	-0.04	8 (2.9)	43 (2.6)	0.02	14.6 (2.1)	14.6 (2.1)	0.00
Gestational Month of Prenatal Care Initiation: 1	31 (5.4)	390 (5.4)	0.00	17 (6.2)	110 (6.7)	-0.02	37.8 (5.5)	37.8 (5.5)	0.00

	Unadjusted, N (column %)						PS Overlap-Weighted, N (column %)		
	MAX/TAF			MarketScan			Both Cohorts Combined		
**Variable**	**Exposed**	**Unexposed**	**SMD**	**Exposed**	**Unexposed**	**SMD**	**Exposed**	**Unexposed**	**SMD**
Gestational Month of Prenatal Care Initiation: 2	212 (37.1)	2,637 (36.4)	0.01	124 (45.4)	714 (43.5)	0.04	272.7 (39.7)	272.7 (39.7)	0.00
Gestational Month of Prenatal Care Initiation: 3	158 (27.6)	1,788 (24.7)	0.07	65 (23.8)	429 (26.1)	-0.05	177.3 (25.8)	177.3 (25.8)	0.00
Gestational Month of Prenatal Care Initiation: 4	72 (12.6)	781 (10.8)	0.06	15 (5.5)	100 (6.1)	-0.03	71.6 (10.4)	71.6 (10.4)	0.00
Gestational Month of Prenatal Care Initiation: 5	35 (6.1)	486 (6.7)	-0.02	10 (3.7)	63 (3.8)	-0.01	38.9 (5.7)	38.9 (5.7)	0.00
Gestational Month of Prenatal Care Initiation: 6	<11	*	-0.13	5 (1.8)	30 (1.8)	0.00	13.8 (2.0)	13.8 (2.0)	0.00
Gestational Month of Prenatal Care Initiation: 7	12 (2.1)	197 (2.7)	-0.04	4 (1.5)	30 (1.8)	-0.03	13.7 (2.0)	13.7 (2.0)	0.00
Gestational Month of Prenatal Care Initiation: 8	<11	*	-0.07	4 (1.5)	19 (1.2)	0.03	8.2 (1.2)	8.2 (1.2)	0.00
Gestational Month of Prenatal Care Initiation: 9	<11	*	-0.07	6 (2.2)	17 (1.0)	0.09	9.7 (1.4)	9.7 (1.4)	0.00
Gestational Month of Prenatal Care Initiation: None	30 (5.2)	413 (5.7)	-0.02	23 (8.4)	130 (7.9)	0.02	43.0 (6.3)	43.0 (6.3)	0.00
Gestational Month of Prenatal Vitamin Initiation: 1	44 (7.7)	468 (6.5)	0.05	21 (7.7)	64 (3.9)	0.16	51.8 (7.5)	51.8 (7.5)	0.00
Gestational Month of Prenatal Vitamin Initiation: 2	147 (25.7)	1,711 (23.6)	0.05	40 (14.7)	188 (11.5)	0.10	162.8 (23.7)	162.8 (23.7)	0.00
Gestational Month of Prenatal Vitamin Initiation: 3	74 (12.9)	946 (13.1)	0.00	13 (4.8)	104 (6.3)	-0.07	77.4 (11.3)	77.4 (11.3)	0.00
Gestational Month of Prenatal Vitamin Initiation: 4	35 (6.1)	479 (6.6)	-0.02	5 (1.8)	39 (2.4)	-0.04	36.4 (5.3)	36.4 (5.3)	0.00
Gestational Month of Prenatal Vitamin Initiation: 5	23 (4.0)	286 (4.0)	0.00	9 (3.3)	25 (1.5)	0.12	27.3 (4.0)	27.3 (4.0)	0.00
Gestational Month of Prenatal Vitamin Initiation: 6	18 (3.2)	197 (2.7)	0.03	2 (0.7)	14 (0.9)	-0.01	17.4 (2.5)	17.4 (2.5)	0.00
Gestational Month of Prenatal Vitamin Initiation: 7	<11	*	-0.08	1 (0.4)	25 (1.5)	-0.12	7.6 (1.1)	7.6 (1.1)	0.00
Gestational Month of Prenatal Vitamin Initiation: 8	<11	*	0.02	1 (0.4)	14 (0.9)	-0.06	8.9 (1.3)	8.9 (1.3)	0.00
Gestational Month of Prenatal Vitamin Initiation: 9	<11	*	-0.11	3 (1.1)	31 (1.9)	-0.07	10.2 (1.5)	10.2 (1.5)	0.00
Gestational Month of Prenatal Vitamin Initiation: -1	22 (3.9)	60 (0.8)	0.20	18 (6.6)	16 (1.0)	0.30	23.0 (3.4)	23.0 (3.4)	0.00
Gestational Month of Prenatal Vitamin Initiation: -2	25 (4.4)	113 (1.6)	0.17	24 (8.8)	34 (2.1)	0.30	33.1 (4.8)	33.1 (4.8)	0.00
Gestational Month of Prenatal Vitamin Initiation: -3	74 (12.9)	163 (2.3)	0.41	52 (19.1)	74 (4.5)	0.46	73.1 (10.6)	73.1 (10.6)	0.00
Gestational Month of Prenatal Vitamin Initiation: None	86 (15.0)	2,334 (32.2)	-0.41	84 (30.8)	1,014 (61.8)	-0.65	157.7 (23.0)	157.7 (23.0)	0.00
**Valproate**									
N Total	687	7,245		113	1,642		636.2	636.2	
Age in Years: <=19	100 (14.6)	1,399 (19.3)	-0.13	2 (1.8)	18 (1.1)	0.06	84.9 (13.3)	84.9 (13.3)	0.00
Age in Years: 20-24	240 (34.9)	2,612 (36.1)	-0.02	8 (7.1)	149 (9.1)	-0.07	197.1 (31.0)	197.1 (31.0)	0.00
Age in Years: 25-29	204 (29.7)	1,941 (26.8)	0.06	28 (24.8)	558 (34.0)	-0.20	183.4 (28.8)	183.4 (28.8)	0.00
Age in Years: 30-34	100 (14.6)	904 (12.5)	0.06	47 (41.6)	606 (36.9)	0.10	114.9 (18.1)	114.9 (18.1)	0.00
Age in Years: 35-39	32 (4.7)	318 (4.4)	0.01	20 (17.7)	250 (15.2)	0.07	41.7 (6.6)	41.7 (6.6)	0.00
Age in Years: >=40	11 (1.6)	71 (1.0)	0.06	8 (7.1)	61 (3.7)	0.15	14.3 (2.2)	14.3 (2.2)	0.00
Race/Ethnicity: Asian or other Pacific Islander	14 (2.0)	74 (1.0)	0.08	N/A	N/A	N/A	N/A	N/A	N/A
Race/Ethnicity: Black or African American	139 (20.2)	2,139 (29.5)	-0.22	N/A	N/A	N/A	N/A	N/A	N/A
Race/Ethnicity: Hispanic or Latino	82 (11.9)	963 (13.3)	-0.04	N/A	N/A	N/A	N/A	N/A	N/A
Race/Ethnicity: Unknown or Other	32 (4.7)	340 (4.7)	0.00	N/A	N/A	N/A	N/A	N/A	N/A
Race/Ethnicity: White	420 (61.1)	3,729 (51.5)	0.20	N/A	N/A	N/A	N/A	N/A	N/A
US Region: Midwest	279 (40.6)	2,598 (35.9)	0.10	39 (34.5)	402 (24.5)	0.22	242.0 (38.0)	242.0 (38.0)	0.00
US Region: Northeast	101 (14.7)	1,194 (16.5)	-0.05	17 (15.0)	270 (16.4)	-0.04	95.3 (15.0)	95.3 (15.0)	0.00
US Region: South	195 (28.4)	2,202 (30.4)	-0.04	44 (38.9)	692 (42.1)	-0.07	193.5 (30.4)	193.5 (30.4)	0.00
US Region: Unknown	0	0	0.00	1 (0.9)	43 (2.6)	-0.13	0.9 (0.1)	0.9 (0.1)	0.00
US Region: West	112 (16.3)	1,251 (17.3)	-0.03	12 (10.6)	235 (14.3)	-0.11	104.6 (16.4)	104.6 (16.4)	0.00
Year of Delivery: 2000	*	<11	-0.02	0 (0.0)	0 (0.0)	0.00	0.0 (0.0)	0.0 (0.0)	0.00
Year of Delivery: 2001	59 (8.6)	146 (2.0)	0.30	0 (0.0)	0 (0.0)	0.00	37.4 (5.9)	37.4 (5.9)	0.00
Year of Delivery: 2002	68 (9.9)	227 (3.1)	0.28	0 (0.0)	0 (0.0)	0.00	48.4 (7.6)	48.4 (7.6)	0.00
Year of Delivery: 2003	76 (11.1)	262 (3.6)	0.29	0 (0.0)	0 (0.0)	0.00	52.2 (8.2)	52.2 (8.2)	0.00
Year of Delivery: 2004	72 (10.5)	368 (5.1)	0.20	11 (9.7)	27 (1.6)	0.35	61.0 (9.6)	61.0 (9.6)	0.00

	Unadjusted, N (column %)						PS Overlap-Weighted, N (column %)		
	MAX/TAF			MarketScan			Both Cohorts Combined		
Variable	Exposed	Unexposed	SMD	Exposed	Unexposed	SMD	Exposed	Unexposed	SMD
Year of Delivery: 2005	65 (9.5)	329 (4.5)	0.19	11 (9.7)	43 (2.6)	0.30	58.6 (9.2)	58.6 (9.2)	0.00
Year of Delivery: 2006	45 (6.6)	347 (4.8)	0.08	8 (7.1)	55 (3.4)	0.17	43.9 (6.9)	43.9 (6.9)	0.00
Year of Delivery: 2007	44 (6.4)	306 (4.2)	0.10	8 (7.1)	61 (3.7)	0.15	41.1 (6.5)	41.1 (6.5)	0.00
Year of Delivery: 2008	42 (6.1)	330 (4.6)	0.07	14 (12.4)	84 (5.1)	0.26	45.5 (7.2)	45.5 (7.2)	0.00
Year of Delivery: 2009	41 (6.0)	370 (5.1)	0.04	13 (11.5)	132 (8.0)	0.12	44.3 (7.0)	44.3 (7.0)	0.00
Year of Delivery: 2010	28 (4.1)	484 (6.7)	-0.12	6 (5.3)	147 (9.0)	-0.14	30.2 (4.8)	30.2 (4.8)	0.00
Year of Delivery: 2011	30 (4.4)	520 (7.2)	-0.12	5 (4.4)	147 (9.0)	-0.18	31.8 (5.0)	31.8 (5.0)	0.00
Year of Delivery: 2012	24 (3.5)	541 (7.5)	-0.18	13 (11.5)	156 (9.5)	0.07	30.6 (4.8)	32.6 (5.1)	0.01
Year of Delivery: 2013	33 (4.8)	625 (8.6)	-0.15	6 (5.3)	107 (6.5)	-0.05	35.8 (5.6)	35.8 (5.6)	0.00
Year of Delivery: 2014	<11	*	-0.26	5 (4.4)	102 (6.2)	-0.08	14.1 (2.2)	14.1 (2.2)	0.00
Year of Delivery: 2015	<11	*	-0.23	4 (3.5)	103 (6.3)	-0.13	11.4 (1.8)	11.4 (1.8)	0.00
Year of Delivery: 2016	15 (2.2)	507 (7.0)	-0.23	4 (3.5)	105 (6.4)	-0.13	17.5 (2.8)	17.5 (2.8)	0.00
Year of Delivery: 2017	16 (2.3)	594 (8.2)	-0.27	1 (0.9)	84 (5.1)	-0.25	16.1 (2.5)	16.1 (2.5)	0.00
Year of Delivery: 2018	11 (1.6)	425 (5.9)	-0.23	2 (1.8)	71 (4.3)	-0.15	12.5 (2.0)	12.5 (2.0)	0.00
Year of Delivery: 2019	0	0	0.00	2 (1.8)	85 (5.2)	-0.19	1.9 (0.3)	1.9 (0.3)	0.00
Year of Delivery: 2020	0	0	0.00	0 (0.0)	74 (4.5)	-0.31	0.0 (0.0)	0.0 (0.0)	0.00
N MH Diagnoses: 0	472 (68.7)	4,225 (58.3)	0.22	98 (86.7)	1,224 (74.5)	0.31	445.9 (70.1)	445.9 (70.1)	0.00
N MH Diagnoses: 1	75 (10.9)	1,137 (15.7)	-0.14	4 (3.5)	173 (10.5)	-0.28	66.9 (10.5)	66.9 (10.5)	0.00
N MH Diagnoses: 2-3	67 (9.8)	1,047 (14.5)	-0.14	8 (7.1)	144 (8.8)	-0.06	62.3 (9.8)	62.3 (9.8)	0.00
N MH Diagnoses: >=4	73 (10.6)	836 (11.5)	-0.03	3 (2.7)	101 (6.2)	-0.17	61.1 (9.6)	61.1 (9.6)	0.00
N ED Visits: 0	627 (91.3)	6,204 (85.6)	0.18	111 (98.2)	1,552 (94.5)	0.20	582.3 (91.5)	582.3 (91.5)	0.00
N ED Visits: 1	40 (5.8)	704 (9.7)	-0.15	1 (0.9)	58 (3.5)	-0.18	36.1 (5.7)	36.1 (5.7)	0.00
N ED Visits: 2-3	*	*	-0.07	0 (0.0)	28 (1.7)	-0.19	14.1 (2.2)	14.1 (2.2)	0.00
N ED Visits: >=4	<11	*	-0.06	1 (0.9)	4 (0.2)	0.09	3.7 (0.6)	3.7 (0.6)	0.00
N MH Hospitalizations: 0	654 (95.2)	6,749 (93.2)	0.09	111 (98.2)	1,609 (98.0)	0.02	607.9 (95.6)	607.9 (95.6)	0.00
N MH Hospitalizations: 1	27 (3.9)	384 (5.3)	-0.07	2 (1.8)	26 (1.6)	0.01	23.3 (3.7)	23.3 (3.7)	0.00
N MH Hospitalizations: 2-3	<11	*	-0.05	0 (0.0)	7 (0.4)	-0.09	4.1 (0.6)	4.1 (0.6)	0.00
N MH Hospitalizations: >=4	<11	*	-0.03	0 (0.0)	0 (0.0)	0.00	0.9 (0.1)	0.9 (0.1)	0.00
N Outpatient Visits: <=3	68 (9.9)	1,012 (14.0)	-0.13	5 (4.4)	99 (6.0)	-0.07	62.7 (9.9)	62.7 (9.9)	0.00
N Outpatient Visits: 4-6	119 (17.3)	1,299 (17.9)	-0.02	21 (18.6)	336 (20.5)	-0.05	115.0 (18.1)	115.0 (18.1)	0.00
N Outpatient Visits: 7-10	169 (24.6)	1,788 (24.7)	0.00	33 (29.2)	482 (29.4)	0.00	163.0 (25.6)	163.0 (25.6)	0.00
N Outpatient Visits: >=11	331 (48.2)	3,146 (43.4)	0.10	54 (47.8)	725 (44.2)	0.07	295.5 (46.4)	295.5 (46.4)	0.00
N Psychotropic Medications (Except ASMs), Mean (SD)	0.0 (0.2)	0.0 (0.2)	0.01	0.0 (0.2)	0.0 (0.2)	-0.03	0.0 (0.2)	0.0 (0.2)	0.00
Alcohol Use Disorder	17 (2.5)	277 (3.8)	-0.08	0 (0.0)	21 (1.3)	-0.16	14.3 (2.2)	14.3 (2.2)	0.00
Smoking	72 (10.5)	1,475 (20.4)	-0.28	2 (1.8)	96 (5.9)	-0.21	65.2 (10.2)	65.2 (10.2)	0.00
Substance Use Disorder	56 (8.2)	1,007 (13.9)	-0.18	5 (4.4)	43 (2.6)	0.10	51.8 (8.1)	51.8 (8.1)	0.00
ADHD	15 (2.2)	191 (2.6)	-0.03	1 (0.9)	40 (2.4)	-0.12	14.3 (2.3)	14.3 (2.3)	0.00
Adjustment Disorder	13 (1.9)	125 (1.7)	0.01	0 (0.0)	8 (0.5)	-0.10	10.2 (1.6)	10.2 (1.6)	0.00
Anxiety	93 (13.5)	1,489 (20.6)	-0.19	10 (8.9)	251 (15.3)	-0.20	88.1 (13.9)	88.1 (13.9)	0.00
Behavioral Disorder	12 (1.8)	131 (1.8)	0.00	1 (0.9)	5 (0.3)	0.08	11.1 (1.8)	11.1 (1.8)	0.00
Bipolar Disorder	63 (9.2)	610 (8.4)	0.03	3 (2.7)	43 (2.6)	0.00	50.7 (8.0)	50.7 (8.0)	0.00
Chronic Pain	<11	*	-0.13	1 (0.9)	29 (1.8)	-0.08	5.9 (0.9)	5.9 (0.9)	0.00
Chronic Fatigue	44 (6.4)	695 (9.6)	-0.12	16 (14.2)	210 (12.8)	0.04	51.1 (8.0)	51.1 (8.0)	0.00
Depression	125 (18.2)	1,660 (22.9)	-0.12	6 (5.3)	234 (14.3)	-0.30	109.1 (17.1)	109.1 (17.1)	0.00
Migraine/Headache	94 (13.7)	991 (13.7)	0.00	15 (13.3)	316 (19.2)	-0.16	88.3 (13.9)	88.3 (13.9)	0.00

	Unadjusted, N (column %)						PS Overlap-Weighted, N (column %)		
	MAX/TAF			MarketScan			Both Cohorts Combined		
Variable	Exposed	Unexposed	SMD	Exposed	Unexposed	SMD	Exposed	Unexposed	SMD
Neuromuscular Disorder	12 (1.8)	312 (4.3)	-0.15	1 (0.9)	73 (4.5)	-0.22	12.0 (1.9)	12.0 (1.9)	0.00
Neuropathic Pain	28 (4.1)	463 (6.4)	-0.10	4 (3.5)	169 (10.3)	-0.27	28.3 (4.5)	28.3 (4.5)	0.00
Other MH Disorders	18 (2.6)	358 (4.9)	-0.12	1 (0.9)	50 (3.1)	-0.16	16.3 (2.6)	16.3 (2.6)	0.00
Other Developmental Disorders	17 (2.5)	82 (1.1)	0.10	0 (0.0)	4 (0.2)	-0.07	10.8 (1.7)	10.8 (1.7)	0.00
Personality Disorder	<11	*	-0.06	0 (0.0)	7 (0.4)	-0.09	6.4 (1.0)	6.4 (1.0)	0.00
Psychosis	13 (1.9)	186 (2.6)	-0.05	0 (0.0)	21 (1.3)	-0.16	10.5 (1.7)	10.5 (1.7)	0.00
Schizophrenia/Schizoaffective Disorder	16 (2.3)	90 (1.2)	0.08	0 (0.0)	6 (0.4)	-0.09	9.9 (1.6)	9.9 (1.6)	0.00
Sleep Disorder	19 (2.8)	282 (3.9)	-0.06	4 (3.5)	98 (6.0)	-0.11	20.1 (3.2)	20.1 (3.2)	0.00
Asthma	88 (12.8)	1,144 (15.8)	-0.09	2 (1.8)	140 (8.5)	-0.31	71.1 (11.2)	71.1 (11.2)	0.00
Autoimmune Disorder	11 (1.6)	206 (2.8)	-0.08	0 (0.0)	96 (5.9)	-0.35	9.6 (1.5)	9.6 (1.5)	0.00
Hyperemesis/NVP	151 (22.0)	2,529 (34.9)	-0.29	14 (12.4)	351 (21.4)	-0.24	142.7 (22.4)	142.7 (22.4)	0.00
Influenca Infection	<11	*	-0.10	6 (5.3)	35 (2.1)	0.17	12.1 (1.9)	12.1 (1.9)	0.00
Obstetric Comorbidity Score: 0	300 (43.7)	2,642 (36.5)	0.15	30 (26.6)	634 (38.6)	-0.26	261.2 (41.1)	261.2 (41.1)	0.00
Obstetric Comorbidity Score: 1	155 (22.6)	1,590 (22.0)	0.01	38 (33.6)	369 (22.5)	0.25	151.1 (23.8)	151.1 (23.8)	0.00
Obstetric Comorbidity Score: 2	80 (11.6)	1,133 (15.6)	-0.12	20 (17.7)	265 (16.1)	0.04	81.4 (12.8)	81.4 (12.8)	0.00
Obstetric Comorbidity Score: >=3	152 (22.1)	1,880 (26.0)	-0.09	25 (22.1)	374 (22.8)	-0.02	142.5 (22.4)	142.5 (22.4)	0.00
Overweight/Obesity	46 (6.7)	811 (11.2)	-0.16	7 (6.2)	185 (11.3)	-0.18	46.0 (7.2)	46.0 (7.2)	0.00
Pre-gestational Diabetes	21 (3.1)	241 (3.3)	-0.02	4 (3.5)	43 (2.6)	0.05	19.3 (3.0)	19.3 (3.0)	0.00
Pre-gestational Hypertension	53 (7.7)	835 (11.5)	-0.13	9 (8.0)	170 (10.4)	-0.08	52.7 (8.3)	52.7 (8.3)	0.00
Poor Nutrition	<11	*	-0.14	5 (4.4)	123 (7.5)	-0.13	13.0 (2.1)	13.0 (2.1)	0.00
Renal Disease	<11	*	-0.10	2 (1.8)	20 (1.2)	0.05	3.0 (0.5)	3.0 (0.5)	0.00
TORCH Infection	22 (3.2)	277 (3.8)	-0.03	2 (1.8)	36 (2.2)	-0.03	20.5 (3.2)	20.5 (3.2)	0.00
Other Sexually Transmitted Diseases	56 (8.2)	812 (11.2)	-0.10	0 (0.0)	56 (3.4)	-0.27	47.2 (7.4)	47.2 (7.4)	0.00
Prenatal Folic Acid Supplementation	502 (73.1)	4,000 (55.2)	0.38	74 (65.5)	527 (32.1)	0.71	440.2 (69.2)	440.2 (69.2)	0.00
Antidepressants	154 (22.4)	1,452 (20.0)	0.06	15 (13.3)	277 (16.9)	-0.10	132.8 (20.9)	132.8 (20.9)	0.00
Antidiabetics	14 (2.0)	86 (1.2)	0.07	5 (4.4)	52 (3.2)	0.07	14.2 (2.2)	14.2 (2.2)	0.00
Antihypertensives	37 (5.4)	450 (6.2)	-0.04	6 (5.3)	104 (6.3)	-0.04	36.1 (5.7)	36.1 (5.7)	0.00
Antipsychotics	40 (5.8)	430 (5.9)	0.00	4 (3.5)	43 (2.6)	0.05	34.3 (5.4)	34.3 (5.4)	0.00
Anxiolytics/Hypnotics/Other Sedatives	57 (8.3)	742 (10.2)	-0.07	7 (6.2)	87 (5.3)	0.04	52.8 (8.3)	52.8 (8.3)	0.00
Barbiturates	43 (6.3)	299 (4.1)	0.10	7 (6.2)	56 (3.4)	0.13	36.0 (5.7)	36.0 (5.7)	0.00
Benzodiazepines	88 (12.8)	760 (10.5)	0.07	19 (16.8)	160 (9.7)	0.21	83.1 (13.1)	83.1 (13.1)	0.00
Corticosteroids	111 (16.2)	1,221 (16.9)	-0.02	19 (16.8)	273 (16.6)	0.01	102.1 (16.1)	102.1 (16.1)	0.00
Insulin	<11	*	-0.03	4 (3.5)	26 (1.6)	0.12	11.0 (1.7)	11.0 (1.7)	0.00
Lithium	<11	*	-0.02	1 (0.9)	2 (0.1)	0.11	2.2 (0.4)	2.2 (0.4)	0.00
NSAIDs	158 (23.0)	1,747 (24.1)	-0.03	13 (11.5)	148 (9.0)	0.08	139.8 (22.0)	139.8 (22.0)	0.00
Opioid Agonist Therapy	<11	*	-0.09	0 (0.0)	8 (0.5)	-0.10	1.9 (0.3)	1.9 (0.3)	0.00
Prescription Opioids	216 (31.4)	2,514 (34.7)	-0.07	27 (23.9)	296 (18.0)	0.14	198.3 (31.2)	198.3 (31.2)	0.00
Progestins	37 (5.4)	459 (6.3)	-0.04	17 (15.0)	231 (14.1)	0.03	43.9 (6.9)	43.9 (6.9)	0.00
Psychostimulants	14 (2.0)	131 (1.8)	0.02	2 (1.8)	44 (2.7)	-0.06	13.0 (2.0)	13.0 (2.0)	0.00
Teratogens, Known	<11	*	-0.03	0 (0.0)	8 (0.5)	-0.10	1.9 (0.3)	1.9 (0.3)	0.00
Teratogens, Suspected	101 (14.7)	1,123 (15.5)	-0.02	12 (10.6)	181 (11.0)	-0.01	92.4 (14.5)	92.4 (14.5)	0.00
Triptans	16 (2.3)	183 (2.5)	-0.01	2 (1.8)	60 (3.7)	-0.12	15.1 (2.4)	15.1 (2.4)	0.00
Adequacy of Prenatal Care Utilization: Inadequate	309 (45.0)	2,867 (39.6)	0.11	69 (61.1)	860 (52.4)	0.18	297.7 (46.8)	297.7 (46.8)	0.00
Adequacy of Prenatal Care Utilization: Intermediate	93 (13.5)	1,100 (15.2)	-0.05	22 (19.5)	358 (21.8)	-0.06	92.2 (14.5)	92.2 (14.5)	0.00
Adequacy of Prenatal Care Utilization: Adequate	101 (14.7)	1,121 (15.5)	-0.02	7 (6.2)	208 (12.7)	-0.22	87.9 (13.8)	87.9 (13.8)	0.00

	Unadjusted, N (column %)						PS Overlap-Weighted, N (column %)		
	MAX/TAF			MarketScan			Both Cohorts Combined		
**Variable**	**Exposed**	**Unexposed**	**SMD**	**Exposed**	**Unexposed**	**SMD**	**Exposed**	**Unexposed**	**SMD**
Adequacy of Prenatal Care Utilization: Adequate Plus	184 (26.8)	2,157 (29.8)	-0.07	15 (13.3)	216 (13.2)	0.00	158.5 (24.9)	158.5 (24.9)	0.00
Multiple Gestation	16 (2.3)	165 (2.3)	0.00	10 (8.9)	43 (2.6)	0.27	18.4 (2.9)	18.4 (2.9)	0.00
Gestational Month of Prenatal Care Initiation: 1	42 (6.1)	390 (5.4)	0.03	11 (9.7)	110 (6.7)	0.11	39.9 (6.3)	39.9 (6.3)	0.00
Gestational Month of Prenatal Care Initiation: 2	249 (36.2)	2,637 (36.4)	0.00	34 (30.1)	714 (43.5)	-0.28	226.2 (35.6)	226.2 (35.6)	0.00
Gestational Month of Prenatal Care Initiation: 3	145 (21.1)	1,788 (24.7)	-0.09	22 (19.5)	429 (26.1)	-0.16	137.3 (21.6)	137.3 (21.6)	0.00
Gestational Month of Prenatal Care Initiation: 4	74 (10.8)	781 (10.8)	0.00	14 (12.4)	100 (6.1)	0.22	67.6 (10.6)	67.6 (10.6)	0.00
Gestational Month of Prenatal Care Initiation: 5	43 (6.3)	486 (6.7)	-0.02	11 (9.7)	63 (3.8)	0.24	44.4 (7.0)	44.4 (7.0)	0.00
Gestational Month of Prenatal Care Initiation: 6	31 (4.5)	277 (3.8)	0.03	4 (3.5)	30 (1.8)	0.11	27.0 (4.2)	27.0 (4.2)	0.00
Gestational Month of Prenatal Care Initiation: 7	36 (5.2)	197 (2.7)	0.13	4 (3.5)	30 (1.8)	0.11	29.2 (4.6)	29.2 (4.6)	0.00
Gestational Month of Prenatal Care Initiation: 8	*	*	-0.03	0 (0.0)	19 (1.2)	-0.15	9.2 (1.4)	9.2 (1.4)	0.00
Gestational Month of Prenatal Care Initiation: 9	<11	*	-0.03	1 (0.9)	17 (1.0)	-0.02	8.9 (1.4)	8.9 (1.4)	0.00
Gestational Month of Prenatal Care Initiation: None	46 (6.7)	413 (5.7)	0.04	12 (10.6)	130 (7.9)	0.09	46.6 (7.3)	46.6 (7.3)	0.00
Gestational Month of Prenatal Vitamin Initiation: 1	54 (7.9)	468 (6.5)	0.05	9 (8.0)	64 (3.9)	0.17	48.1 (7.6)	48.1 (7.6)	0.00
Gestational Month of Prenatal Vitamin Initiation: 2	202 (29.4)	1,711 (23.6)	0.13	22 (19.5)	188 (11.5)	0.22	178.1 (28.0)	178.1 (28.0)	0.00
Gestational Month of Prenatal Vitamin Initiation: 3	88 (12.8)	946 (13.1)	-0.01	6 (5.3)	104 (6.3)	-0.04	78.6 (12.4)	78.6 (12.4)	0.00
Gestational Month of Prenatal Vitamin Initiation: 4	51 (7.4)	479 (6.6)	0.03	2 (1.8)	39 (2.4)	-0.04	41.7 (6.6)	41.7 (6.6)	0.00
Gestational Month of Prenatal Vitamin Initiation: 5	35 (5.1)	286 (4.0)	0.06	2 (1.8)	25 (1.5)	0.02	29.3 (4.6)	29.3 (4.6)	0.00
Gestational Month of Prenatal Vitamin Initiation: 6	23 (3.4)	197 (2.7)	0.04	0 (0.0)	14 (0.9)	-0.13	17.8 (2.8)	17.8 (2.8)	0.00
Gestational Month of Prenatal Vitamin Initiation: 7	*	*	0.00	0 (0.0)	25 (1.5)	-0.18	13.3 (2.1)	13.3 (2.1)	0.00
Gestational Month of Prenatal Vitamin Initiation: 8	<11	*	-0.02	0 (0.0)	14 (0.9)	-0.13	6.8 (1.1)	6.8 (1.1)	0.00
Gestational Month of Prenatal Vitamin Initiation: 9	18 (2.6)	224 (3.1)	-0.03	1 (0.9)	31 (1.9)	-0.09	17.1 (2.7)	17.1 (2.7)	0.00
Gestational Month of Prenatal Vitamin Initiation: -1	19 (2.8)	60 (0.8)	0.15	6 (5.3)	16 (1.0)	0.25	14.3 (2.3)	14.3 (2.3)	0.00
Gestational Month of Prenatal Vitamin Initiation: -2	25 (3.6)	113 (1.6)	0.13	10 (8.9)	34 (2.1)	0.30	23.5 (3.7)	23.5 (3.7)	0.00
Gestational Month of Prenatal Vitamin Initiation: -3	52 (7.6)	163 (2.3)	0.25	19 (16.8)	74 (4.5)	0.41	47.5 (7.5)	47.5 (7.5)	0.00
Gestational Month of Prenatal Vitamin Initiation: None	96 (14.0)	2,334 (32.2)	-0.44	36 (31.9)	1,014 (61.8)	-0.63	120.2 (18.9)	120.2 (18.9)	0.00
**Zonisamide**									
N Total	285	7,245		161	1,642		385.6	385.6	
Age in Years: <=19	45 (15.8)	1,399 (19.3)	-0.09	1 (0.6)	18 (1.1)	-0.05	42.0 (10.9)	42.0 (10.9)	0.00
Age in Years: 20-24	104 (36.5)	2,612 (36.1)	0.01	11 (6.8)	149 (9.1)	-0.08	103.8 (26.9)	103.8 (26.9)	0.00
Age in Years: 25-29	77 (27.0)	1,941 (26.8)	0.01	48 (29.8)	558 (34.0)	-0.09	107.9 (28.0)	107.9 (28.0)	0.00
Age in Years: 30-34	42 (14.7)	904 (12.5)	0.07	62 (38.5)	606 (36.9)	0.03	85.7 (22.2)	85.7 (22.2)	0.00
Age in Years: 35-39	*	*	0.04	31 (19.3)	250 (15.2)	0.11	38.1 (9.9)	38.1 (9.9)	0.00
Age in Years: >=40	<11	*	-0.03	8 (5.0)	61 (3.7)	0.06	8.1 (2.1)	8.1 (2.1)	0.00
Race/Ethnicity: Asian or other Pacific Islander	<11	*	0.00	N/A	N/A	N/A	N/A	N/A	N/A
Race/Ethnicity: Black or African American	*	*	-0.26	N/A	N/A	N/A	N/A	N/A	N/A
Race/Ethnicity: Hispanic or Latino	35 (12.3)	963 (13.3)	-0.03	N/A	N/A	N/A	N/A	N/A	N/A
Race/Ethnicity: Unknown or Other	25 (8.8)	340 (4.7)	0.16	N/A	N/A	N/A	N/A	N/A	N/A
Race/Ethnicity: White	169 (59.3)	3,729 (51.5)	0.16	N/A	N/A	N/A	N/A	N/A	N/A
US Region: Midwest	107 (37.5)	2,598 (35.9)	0.03	42 (26.1)	402 (24.5)	0.04	127.7 (33.1)	127.7 (33.1)	0.00
US Region: Northeast	62 (21.8)	1,194 (16.5)	0.13	11 (6.8)	270 (16.4)	-0.30	64.2 (16.6)	64.2 (16.6)	0.00
US Region: South	66 (23.2)	2,202 (30.4)	-0.16	72 (44.7)	692 (42.1)	0.05	120.8 (31.3)	120.8 (31.3)	0.00
US Region: Unknown	0	0	0.00	7 (4.4)	43 (2.6)	0.09	4.7 (1.2)	4.7 (1.2)	0.00
US Region: West	50 (17.5)	1,251 (17.3)	0.01	29 (18.0)	235 (14.3)	0.10	68.3 (17.7)	68.3 (17.7)	0.00
Year of Delivery: 2000	*	<11	-0.02	0 (0.0)	0 (0.0)	0.00	0.0 (0.0)	0.0 (0.0)	0.00
Year of Delivery: 2001	0	146 (2.0)	-0.20	0 (0.0)	0 (0.0)	0.00	0.0 (0.0)	0.0 (0.0)	0.00

	Unadjusted, N (column %)						PS Overlap-Weighted, N (column %)		
	MAX/TAF			MarketScan			Both Cohorts Combined		
Variable	Exposed	Unexposed	SMD	Exposed	Unexposed	SMD	Exposed	Unexposed	SMD
Year of Delivery: 2002	<11	*	-0.18	0 (0.0)	0 (0.0)	0.00	1.9 (0.5)	1.9 (0.5)	0.00
Year of Delivery: 2003	13 (4.6)	262 (3.6)	0.05	0 (0.0)	0 (0.0)	0.00	12.2 (3.2)	12.2 (3.2)	0.00
Year of Delivery: 2004	14 (4.9)	368 (5.1)	-0.01	0 (0.0)	27 (1.6)	-0.18	12.8 (3.3)	12.8 (3.3)	0.00
Year of Delivery: 2005	<11	*	-0.11	1 (0.6)	43 (2.6)	-0.16	7.2 (1.9)	7.2 (1.9)	0.00
Year of Delivery: 2006	15 (5.3)	347 (4.8)	0.02	7 (4.4)	55 (3.4)	0.05	18.8 (4.9)	18.8 (4.9)	0.00
Year of Delivery: 2007	12 (4.2)	306 (4.2)	0.00	7 (4.4)	61 (3.7)	0.03	16.3 (4.2)	16.3 (4.2)	0.00
Year of Delivery: 2008	16 (5.6)	330 (4.6)	0.05	8 (5.0)	84 (5.1)	-0.01	20.6 (5.3)	20.6 (5.3)	0.00
Year of Delivery: 2009	14 (4.9)	370 (5.1)	-0.01	13 (8.1)	132 (8.0)	0.00	24.1 (6.2)	24.1 (6.2)	0.00
Year of Delivery: 2010	19 (6.7)	484 (6.7)	0.00	8 (5.0)	147 (9.0)	-0.16	24.8 (6.4)	24.8 (6.4)	0.00
Year of Delivery: 2011	15 (5.3)	520 (7.2)	-0.08	18 (11.2)	147 (9.0)	0.07	27.6 (7.2)	27.6 (7.2)	0.00
Year of Delivery: 2012	18 (6.3)	541 (7.5)	-0.05	8 (5.0)	156 (9.5)	-0.18	32.6 (8.5)	23.3 (6.0)	-0.09
Year of Delivery: 2013	22 (7.7)	625 (8.6)	-0.03	9 (5.6)	107 (6.5)	-0.04	27.9 (7.2)	27.9 (7.2)	0.00
Year of Delivery: 2014	21 (7.4)	480 (6.6)	0.03	9 (5.6)	102 (6.2)	-0.03	26.3 (6.8)	26.3 (6.8)	0.00
Year of Delivery: 2015	15 (5.3)	383 (5.3)	0.00	10 (6.2)	103 (6.3)	0.00	21.8 (5.7)	21.8 (5.7)	0.00
Year of Delivery: 2016	19 (6.7)	507 (7.0)	-0.01	11 (6.8)	105 (6.4)	0.02	26.1 (6.8)	26.1 (6.8)	0.00
Year of Delivery: 2017	29 (10.2)	594 (8.2)	0.07	9 (5.6)	84 (5.1)	0.02	31.9 (8.3)	31.9 (8.3)	0.00
Year of Delivery: 2018	34 (11.9)	425 (5.9)	0.21	7 (4.4)	71 (4.3)	0.00	34.5 (9.0)	34.5 (9.0)	0.00
Year of Delivery: 2019	0	0	0.00	8 (5.0)	85 (5.2)	-0.01	5.7 (1.5)	5.7 (1.5)	0.00
Year of Delivery: 2020	0	0	0.00	11 (6.8)	74 (4.5)	0.10	8.5 (2.2)	8.5 (2.2)	0.00
N MH Diagnoses: 0	178 (62.5)	4,225 (58.3)	0.08	136 (84.5)	1,224 (74.5)	0.25	270.3 (70.1)	270.3 (70.1)	0.00
N MH Diagnoses: 1	33 (11.6)	1,137 (15.7)	-0.12	9 (5.6)	173 (10.5)	-0.18	38.0 (9.9)	38.0 (9.9)	0.00
N MH Diagnoses: 2-3	45 (15.8)	1,047 (14.5)	0.04	9 (5.6)	144 (8.8)	-0.12	46.1 (11.9)	46.1 (11.9)	0.00
N MH Diagnoses: >=4	29 (10.2)	836 (11.5)	-0.04	7 (4.4)	101 (6.2)	-0.08	31.3 (8.1)	31.3 (8.1)	0.00
N ED Visits: 0	248 (87.0)	6,204 (85.6)	0.04	155 (96.3)	1,552 (94.5)	0.08	347.7 (90.2)	347.7 (90.2)	0.00
N ED Visits: 1	29 (10.2)	704 (9.7)	0.02	6 (3.7)	58 (3.5)	0.01	30.4 (7.9)	30.4 (7.9)	0.00
N ED Visits: 2-3	<11	*	-0.12	0 (0.0)	28 (1.7)	-0.19	4.8 (1.2)	4.8 (1.2)	0.00
N ED Visits: >=4	<11	*	0.01	0 (0.0)	4 (0.2)	-0.07	2.7 (0.7)	2.7 (0.7)	0.00
N MH Hospitalizations: 0	273 (95.8)	6,749 (93.2)	0.12	160 (99.4)	1,609 (98.0)	0.12	373.7 (96.9)	373.7 (96.9)	0.00
N MH Hospitalizations: 1	<11	*	-0.11	1 (0.6)	26 (1.6)	-0.09	9.1 (2.4)	9.1 (2.4)	0.00
N MH Hospitalizations: 2-3	<11	*	-0.02	0 (0.0)	7 (0.4)	-0.09	2.9 (0.8)	2.9 (0.8)	0.00
N MH Hospitalizations: >=4	0	22 (0.3)	-0.08	0 (0.0)	0 (0.0)	0.00	0.0 (0.0)	0.0 (0.0)	0.00
N Outpatient Visits: <=3	26 (9.1)	1,012 (14.0)	-0.15	4 (2.5)	99 (6.0)	-0.18	28.1 (7.3)	28.1 (7.3)	0.00
N Outpatient Visits: 4-6	47 (16.5)	1,299 (17.9)	-0.04	30 (18.6)	336 (20.5)	-0.05	69.4 (18.0)	69.4 (18.0)	0.00
N Outpatient Visits: 7-10	49 (17.2)	1,788 (24.7)	-0.18	65 (40.4)	482 (29.4)	0.23	97.4 (25.3)	97.4 (25.3)	0.00
N Outpatient Visits: >=11	163 (57.2)	3,146 (43.4)	0.28	62 (38.5)	725 (44.2)	-0.11	190.7 (49.5)	190.7 (49.5)	0.00
N Psychotropic Medications (Except ASMs), Mean (SD)	0.0 (0.2)	0.0 (0.2)	0.08	0.0 (0.1)	0.0 (0.2)	-0.13	0.0 (0.2)	0.0 (0.2)	0.00
Alcohol Use Disorder	<11	*	-0.13	2 (1.2)	21 (1.3)	0.00	6.4 (1.7)	6.4 (1.7)	0.00
Smoking	43 (15.1)	1,475 (20.4)	-0.14	7 (4.4)	96 (5.9)	-0.07	45.2 (11.7)	45.2 (11.7)	0.00
Substance Use Disorder	27 (9.5)	1,007 (13.9)	-0.14	3 (1.9)	43 (2.6)	-0.05	27.5 (7.1)	27.5 (7.1)	0.00
ADHD	<11	*	0.01	2 (1.2)	40 (2.4)	-0.09	8.8 (2.3)	8.8 (2.3)	0.00
Adjustment Disorder	<11	*	0.03	1 (0.6)	8 (0.5)	0.02	5.9 (1.5)	5.9 (1.5)	0.00
Anxiety	45 (15.8)	1,489 (20.6)	-0.12	20 (12.4)	251 (15.3)	-0.08	56.5 (14.7)	56.5 (14.7)	0.00
Behavioral Disorder	<11	*	0.02	0 (0.0)	5 (0.3)	-0.08	5.3 (1.4)	5.3 (1.4)	0.00
Bipolar Disorder	18 (6.3)	610 (8.4)	-0.08	1 (0.6)	43 (2.6)	-0.16	16.9 (4.4)	16.9 (4.4)	0.00
Chronic Pain	<11	*	-0.02	1 (0.6)	29 (1.8)	-0.11	6.6 (1.7)	6.6 (1.7)	0.00

	Unadjusted, N (column %)						PS Overlap-Weighted, N (column %)		
	MAX/TAF			MarketScan			Both Cohorts Combined		
Variable	Exposed	Unexposed	SMD	Exposed	Unexposed	SMD	Exposed	Unexposed	SMD
Chronic Fatigue	20 (7.0)	695 (9.6)	-0.09	15 (9.3)	210 (12.8)	-0.11	31.8 (8.3)	31.8 (8.3)	0.00
Depression	56 (19.7)	1,660 (22.9)	-0.08	17 (10.6)	234 (14.3)	-0.11	64.3 (16.7)	64.3 (16.7)	0.00
Migraine/Headache	45 (15.8)	991 (13.7)	0.06	18 (11.2)	316 (19.2)	-0.23	55.6 (14.4)	55.6 (14.4)	0.00
Neuromuscular Disorder	13 (4.6)	312 (4.3)	0.01	3 (1.9)	73 (4.5)	-0.15	14.2 (3.7)	14.2 (3.7)	0.00
Neuropathic Pain	18 (6.3)	463 (6.4)	0.00	9 (5.6)	169 (10.3)	-0.17	24.1 (6.3)	24.1 (6.3)	0.00
Other MH Disorders	16 (5.6)	358 (4.9)	0.03	2 (1.2)	50 (3.1)	-0.12	16.0 (4.2)	16.0 (4.2)	0.00
Other Developmental Disorders	<11	*	0.16	1 (0.6)	4 (0.2)	0.06	8.3 (2.1)	8.3 (2.1)	0.00
Personality Disorder	<11	*	0.00	1 (0.6)	7 (0.4)	0.03	5.2 (1.4)	5.2 (1.4)	0.00
Psychosis	12 (4.2)	186 (2.6)	0.09	0 (0.0)	21 (1.3)	-0.16	10.0 (2.6)	10.0 (2.6)	0.00
Schizophrenia/Schizoaffective Disorder	<11	*	-0.10	0 (0.0)	6 (0.4)	-0.09	1.0 (0.3)	1.0 (0.3)	0.00
Sleep Disorder	16 (5.6)	282 (3.9)	0.08	9 (5.6)	98 (6.0)	-0.02	21.4 (5.6)	21.4 (5.6)	0.00
Asthma	31 (10.9)	1,144 (15.8)	-0.14	10 (6.2)	140 (8.5)	-0.09	36.4 (9.5)	36.4 (9.5)	0.00
Autoimmune Disorder	<11	*	-0.13	5 (3.1)	96 (5.9)	-0.13	7.5 (1.9)	7.5 (1.9)	0.00
Hyperemesis/NVP	81 (28.4)	2,529 (34.9)	-0.14	24 (14.9)	351 (21.4)	-0.17	93.6 (24.3)	93.6 (24.3)	0.00
Influenca Infection	<11	*	-0.11	2 (1.2)	35 (2.1)	-0.07	4.7 (1.2)	4.7 (1.2)	0.00
Obstetric Comorbidity Score: 0	138 (48.4)	2,642 (36.5)	0.24	64 (39.8)	634 (38.6)	0.02	173.2 (44.9)	173.2 (44.9)	0.00
Obstetric Comorbidity Score: 1	55 (19.3)	1,590 (22.0)	-0.07	37 (23.0)	369 (22.5)	0.01	79.4 (20.6)	79.4 (20.6)	0.00
Obstetric Comorbidity Score: 2	44 (15.4)	1,133 (15.6)	-0.01	32 (19.9)	265 (16.1)	0.10	65.7 (17.1)	65.7 (17.1)	0.00
Obstetric Comorbidity Score: >=3	48 (16.8)	1,880 (26.0)	-0.22	28 (17.4)	374 (22.8)	-0.13	67.3 (17.5)	67.3 (17.5)	0.00
Overweight/Obesity	38 (13.3)	811 (11.2)	0.07	31 (19.3)	185 (11.3)	0.22	58.0 (15.0)	58.0 (15.0)	0.00
Pre-gestational Diabetes	<11	*	-0.01	2 (1.2)	43 (2.6)	-0.10	10.0 (2.6)	10.0 (2.6)	0.00
Pre-gestational Hypertension	19 (6.7)	835 (11.5)	-0.17	13 (8.1)	170 (10.4)	-0.08	28.5 (7.4)	28.5 (7.4)	0.00
Poor Nutrition	<11	*	-0.01	9 (5.6)	123 (7.5)	-0.08	16.1 (4.2)	16.1 (4.2)	0.00
Renal Disease	<11	*	-0.09	2 (1.2)	20 (1.2)	0.00	2.8 (0.7)	2.8 (0.7)	0.00
TORCH Infection	<11	*	-0.08	6 (3.7)	36 (2.2)	0.09	11.4 (3.0)	11.4 (3.0)	0.00
Other Sexually Transmitted Diseases	24 (8.4)	812 (11.2)	-0.09	5 (3.1)	56 (3.4)	-0.02	26.1 (6.8)	26.1 (6.8)	0.00
Prenatal Folic Acid Supplementation	209 (73.3)	4,000 (55.2)	0.38	103 (64.0)	527 (32.1)	0.67	259.8 (67.4)	259.8 (67.4)	0.00
Antidepressants	68 (23.9)	1,452 (20.0)	0.09	25 (15.5)	277 (16.9)	-0.04	78.6 (20.4)	78.6 (20.4)	0.00
Antidiabetics	<11	*	-0.01	6 (3.7)	52 (3.2)	0.03	7.6 (2.0)	7.6 (2.0)	0.00
Antihypertensives	11 (3.9)	450 (6.2)	-0.11	10 (6.2)	104 (6.3)	-0.01	19.4 (5.0)	19.4 (5.0)	0.00
Antipsychotics	14 (4.9)	430 (5.9)	-0.05	2 (1.2)	43 (2.6)	-0.10	14.9 (3.9)	14.9 (3.9)	0.00
Anxiolytics/Hypnotics/Other Sedatives	19 (6.7)	742 (10.2)	-0.13	11 (6.8)	87 (5.3)	0.06	25.8 (6.7)	25.8 (6.7)	0.00
Barbiturates	15 (5.3)	299 (4.1)	0.05	6 (3.7)	56 (3.4)	0.02	16.9 (4.4)	16.9 (4.4)	0.00
Benzodiazepines	45 (15.8)	760 (10.5)	0.16	18 (11.2)	160 (9.7)	0.05	51.6 (13.4)	51.6 (13.4)	0.00
Corticosteroids	49 (17.2)	1,221 (16.9)	0.01	26 (16.2)	273 (16.6)	-0.01	64.0 (16.6)	64.0 (16.6)	0.00
Insulin	<11	*	-0.03	1 (0.6)	26 (1.6)	-0.09	4.5 (1.2)	4.5 (1.2)	0.00
Lithium	<11	*	0.11	0 (0.0)	2 (0.1)	-0.05	3.3 (0.9)	3.3 (0.9)	0.00
NSAIDs	57 (20.0)	1,747 (24.1)	-0.10	14 (8.7)	148 (9.0)	-0.01	64.1 (16.6)	64.1 (16.6)	0.00
Opioid Agonist Therapy	0	76 (1.1)	-0.15	0 (0.0)	8 (0.5)	-0.10	0.0 (0.0)	0.0 (0.0)	0.00
Prescription Opioids	77 (27.0)	2,514 (34.7)	-0.17	22 (13.7)	296 (18.0)	-0.12	87.3 (22.6)	87.3 (22.6)	0.00
Progestins	23 (8.1)	459 (6.3)	0.07	28 (17.4)	231 (14.1)	0.09	40.8 (10.6)	40.8 (10.6)	0.00
Psychostimulants	<11	*	0.04	1 (0.6)	44 (2.7)	-0.16	7.2 (1.9)	7.2 (1.9)	0.00
Teratogens, Known	0	34 (0.5)	-0.10	1 (0.6)	8 (0.5)	0.02	0.8 (0.2)	0.8 (0.2)	0.00
Teratogens, Suspected	40 (14.0)	1,123 (15.5)	-0.04	16 (9.9)	181 (11.0)	-0.04	49.3 (12.8)	49.3 (12.8)	0.00
Triptans	14 (4.9)	183 (2.5)	0.13	10 (6.2)	60 (3.7)	0.12	20.5 (5.3)	20.5 (5.3)	0.00

	Unadjusted, N (column %)						PS Overlap-Weighted, N (column %)		
	MAX/TAF			MarketScan			Both Cohorts Combined		
**Variable**	**Exposed**	**Unexposed**	**SMD**	**Exposed**	**Unexposed**	**SMD**	**Exposed**	**Unexposed**	**SMD**
Adequacy of Prenatal Care Utilization: Inadequate	96 (33.7)	2,867 (39.6)	-0.12	77 (47.8)	860 (52.4)	-0.09	147.8 (38.3)	147.8 (38.3)	0.00
Adequacy of Prenatal Care Utilization: Intermediate	41 (14.4)	1,100 (15.2)	-0.02	42 (26.1)	358 (21.8)	0.10	71.6 (18.6)	71.6 (18.6)	0.00
Adequacy of Prenatal Care Utilization: Adequate	44 (15.4)	1,121 (15.5)	0.00	24 (14.9)	208 (12.7)	0.06	59.3 (15.4)	59.3 (15.4)	0.00
Adequacy of Prenatal Care Utilization: Adequate Plus	104 (36.5)	2,157 (29.8)	0.14	18 (11.2)	216 (13.2)	-0.06	106.9 (27.7)	106.9 (27.7)	0.00
Multiple Gestation	<11	*	0.07	5 (3.1)	43 (2.6)	0.03	12.3 (3.2)	12.3 (3.2)	0.00
Gestational Month of Prenatal Care Initiation: 1	17 (6.0)	390 (5.4)	0.03	12 (7.5)	110 (6.7)	0.03	24.7 (6.4)	24.7 (6.4)	0.00
Gestational Month of Prenatal Care Initiation: 2	130 (45.6)	2,637 (36.4)	0.19	64 (39.8)	714 (43.5)	-0.08	166.0 (43.0)	166.0 (43.0)	0.00
Gestational Month of Prenatal Care Initiation: 3	55 (19.3)	1,788 (24.7)	-0.13	50 (31.1)	429 (26.1)	0.11	92.0 (23.9)	92.0 (23.9)	0.00
Gestational Month of Prenatal Care Initiation: 4	25 (8.8)	781 (10.8)	-0.07	13 (8.1)	100 (6.1)	0.08	32.5 (8.4)	32.5 (8.4)	0.00
Gestational Month of Prenatal Care Initiation: 5	17 (6.0)	486 (6.7)	-0.03	4 (2.5)	63 (3.8)	-0.08	18.5 (4.8)	18.5 (4.8)	0.00
Gestational Month of Prenatal Care Initiation: 6	19 (6.7)	277 (3.8)	0.13	2 (1.2)	30 (1.8)	-0.05	18.5 (4.8)	18.5 (4.8)	0.00
Gestational Month of Prenatal Care Initiation: 7	<11	*	-0.02	2 (1.2)	30 (1.8)	-0.05	7.7 (2.0)	7.7 (2.0)	0.00
Gestational Month of Prenatal Care Initiation: 8	0	142 (2.0)	-0.20	2 (1.2)	19 (1.2)	0.01	1.8 (0.5)	1.8 (0.5)	0.00
Gestational Month of Prenatal Care Initiation: 9	<11	*	-0.01	3 (1.9)	17 (1.0)	0.07	6.6 (1.7)	6.6 (1.7)	0.00
Gestational Month of Prenatal Care Initiation: None	<11	*	-0.10	9 (5.6)	130 (7.9)	-0.09	17.2 (4.5)	17.2 (4.5)	0.00
Gestational Month of Prenatal Vitamin Initiation: 1	22 (7.7)	468 (6.5)	0.05	14 (8.7)	64 (3.9)	0.20	31.2 (8.1)	31.2 (8.1)	0.00
Gestational Month of Prenatal Vitamin Initiation: 2	72 (25.3)	1,711 (23.6)	0.04	19 (11.8)	188 (11.5)	0.01	83.8 (21.7)	83.8 (21.7)	0.00
Gestational Month of Prenatal Vitamin Initiation: 3	37 (13.0)	946 (13.1)	0.00	9 (5.6)	104 (6.3)	-0.03	43.0 (11.2)	43.0 (11.2)	0.00
Gestational Month of Prenatal Vitamin Initiation: 4	11 (3.9)	479 (6.6)	-0.12	7 (4.4)	39 (2.4)	0.11	16.5 (4.3)	16.5 (4.3)	0.00
Gestational Month of Prenatal Vitamin Initiation: 5	12 (4.2)	286 (4.0)	0.01	2 (1.2)	25 (1.5)	-0.02	12.8 (3.3)	12.8 (3.3)	0.00
Gestational Month of Prenatal Vitamin Initiation: 6	13 (4.6)	197 (2.7)	0.10	0 (0.0)	14 (0.9)	-0.13	11.5 (3.0)	11.5 (3.0)	0.00
Gestational Month of Prenatal Vitamin Initiation: 7	<11	*	-0.07	1 (0.6)	25 (1.5)	-0.09	4.7 (1.2)	4.7 (1.2)	0.00
Gestational Month of Prenatal Vitamin Initiation: 8	<11	*	-0.03	1 (0.6)	14 (0.9)	-0.03	3.8 (1.0)	3.8 (1.0)	0.00
Gestational Month of Prenatal Vitamin Initiation: 9	<11	*	-0.14	3 (1.9)	31 (1.9)	0.00	5.7 (1.5)	5.7 (1.5)	0.00
Gestational Month of Prenatal Vitamin Initiation: -1	12 (4.2)	60 (0.8)	0.22	6 (3.7)	16 (1.0)	0.18	12.8 (3.3)	12.8 (3.3)	0.00
Gestational Month of Prenatal Vitamin Initiation: -2	15 (5.3)	113 (1.6)	0.20	16 (9.9)	34 (2.1)	0.33	21.8 (5.7)	21.8 (5.7)	0.00
Gestational Month of Prenatal Vitamin Initiation: -3	38 (13.3)	163 (2.3)	0.42	32 (19.9)	74 (4.5)	0.48	49.0 (12.7)	49.0 (12.7)	0.00
Gestational Month of Prenatal Vitamin Initiation: None	43 (15.1)	2,334 (32.2)	-0.41	51 (31.7)	1,014 (61.8)	-0.63	89.2 (23.1)	89.2 (23.1)	0.00
**Phenobarbital**									
N Total	346	7,245		70	1,642		162.2	162.2	
Age in Years: <=19	42 (12.1)	1,399 (19.3)	-0.20	0 (0.0)	18 (1.1)	-0.15	18.8 (11.6)	18.8 (11.6)	0.00
Age in Years: 20-24	105 (30.4)	2,612 (36.1)	-0.12	6 (8.6)	149 (9.1)	-0.02	52.3 (32.2)	52.3 (32.2)	0.00
Age in Years: 25-29	96 (27.8)	1,941 (26.8)	0.02	16 (22.9)	558 (34.0)	-0.25	45.1 (27.8)	45.1 (27.8)	0.00
Age in Years: 30-34	55 (15.9)	904 (12.5)	0.10	18 (25.7)	606 (36.9)	-0.24	26.3 (16.2)	26.3 (16.2)	0.00
Age in Years: 35-39	37 (10.7)	318 (4.4)	0.24	26 (37.1)	250 (15.2)	0.51	16.2 (10.0)	16.2 (10.0)	0.00
Age in Years: >=40	11 (3.2)	71 (1.0)	0.15	4 (5.7)	61 (3.7)	0.09	3.5 (2.2)	3.5 (2.2)	0.00
Race/Ethnicity: Asian or other Pacific Islander	<11	*	0.04	N/A	N/A	N/A	N/A	N/A	N/A
Race/Ethnicity: Black or African American	*	*	0.04	N/A	N/A	N/A	N/A	N/A	N/A
Race/Ethnicity: Hispanic or Latino	48 (13.9)	963 (13.3)	0.02	N/A	N/A	N/A	N/A	N/A	N/A
Race/Ethnicity: Unknown or Other	17 (4.9)	340 (4.7)	0.01	N/A	N/A	N/A	N/A	N/A	N/A
Race/Ethnicity: White	167 (48.3)	3,729 (51.5)	-0.06	N/A	N/A	N/A	N/A	N/A	N/A
US Region: Midwest	90 (26.0)	2,598 (35.9)	-0.21	13 (18.6)	402 (24.5)	-0.14	43.7 (27.0)	43.7 (27.0)	0.00
US Region: Northeast	47 (13.6)	1,194 (16.5)	-0.08	7 (10.0)	270 (16.4)	-0.19	20.2 (12.5)	20.2 (12.5)	0.00
US Region: South	134 (38.7)	2,202 (30.4)	0.18	35 (50.0)	692 (42.1)	0.16	68.6 (42.3)	68.6 (42.3)	0.00
US Region: Unknown	0	0	0.00	0 (0.0)	43 (2.6)	-0.23	0.0 (0.0)	0.0 (0.0)	0.00

	Unadjusted, N (column %)						PS Overlap-Weighted, N (column %)		
	MAX/TAF			MarketScan			Both Cohorts Combined		
Variable	Exposed	Unexposed	SMD	Exposed	Unexposed	SMD	Exposed	Unexposed	SMD
US Region: West	75 (21.7)	1,251 (17.3)	0.11	15 (21.4)	235 (14.3)	0.19	29.7 (18.3)	29.7 (18.3)	0.00
Year of Delivery: 2000	<11	<11	0.10	0 (0.0)	0 (0.0)	0.00	0.7 (0.4)	0.7 (0.4)	0.00
Year of Delivery: 2001	39 (11.3)	146 (2.0)	0.38	0 (0.0)	0 (0.0)	0.00	10.2 (6.3)	10.2 (6.3)	0.00
Year of Delivery: 2002	36 (10.4)	227 (3.1)	0.29	0 (0.0)	0 (0.0)	0.00	8.7 (5.4)	8.7 (5.4)	0.00
Year of Delivery: 2003	40 (11.6)	262 (3.6)	0.30	0 (0.0)	0 (0.0)	0.00	14.7 (9.1)	14.7 (9.1)	0.00
Year of Delivery: 2004	34 (9.8)	368 (5.1)	0.18	13 (18.6)	27 (1.6)	0.58	15.3 (9.4)	15.3 (9.4)	0.00
Year of Delivery: 2005	38 (11.0)	329 (4.5)	0.24	8 (11.4)	43 (2.6)	0.35	14.8 (9.1)	14.8 (9.1)	0.00
Year of Delivery: 2006	25 (7.2)	347 (4.8)	0.10	7 (10.0)	55 (3.4)	0.27	15.0 (9.2)	15.0 (9.2)	0.00
Year of Delivery: 2007	25 (7.2)	306 (4.2)	0.13	6 (8.6)	61 (3.7)	0.20	10.8 (6.7)	10.8 (6.7)	0.00
Year of Delivery: 2008	14 (4.1)	330 (4.6)	-0.03	10 (14.3)	84 (5.1)	0.31	9.4 (5.8)	9.4 (5.8)	0.00
Year of Delivery: 2009	28 (8.1)	370 (5.1)	0.12	7 (10.0)	132 (8.0)	0.07	12.5 (7.7)	12.5 (7.7)	0.00
Year of Delivery: 2010	13 (3.8)	484 (6.7)	-0.13	3 (4.3)	147 (9.0)	-0.19	8.3 (5.1)	8.3 (5.1)	0.00
Year of Delivery: 2011	14 (4.1)	520 (7.2)	-0.14	3 (4.3)	147 (9.0)	-0.19	8.6 (5.3)	8.6 (5.3)	0.00
Year of Delivery: 2012	<11	*	-0.24	4 (5.7)	156 (9.5)	-0.14	7.8 (4.8)	7.9 (4.9)	0.00
Year of Delivery: 2013	<11	*	-0.26	4 (5.7)	107 (6.5)	-0.03	7.3 (4.5)	7.3 (4.5)	0.00
Year of Delivery: 2014	<11	*	-0.27	1 (1.4)	102 (6.2)	-0.25	5.0 (3.1)	5.0 (3.1)	0.00
Year of Delivery: 2015	<11	*	-0.24	1 (1.4)	103 (6.3)	-0.25	3.6 (2.2)	3.6 (2.2)	0.00
Year of Delivery: 2016	<11	*	-0.30	1 (1.4)	105 (6.4)	-0.26	4.0 (2.5)	4.0 (2.5)	0.00
Year of Delivery: 2017	<11	*	-0.34	1 (1.4)	84 (5.1)	-0.21	2.9 (1.8)	2.9 (1.8)	0.00
Year of Delivery: 2018	<11	*	-0.26	1 (1.4)	71 (4.3)	-0.17	2.6 (1.6)	2.6 (1.6)	0.00
Year of Delivery: 2019	0	0	0.00	0 (0.0)	85 (5.2)	-0.33	0.0 (0.0)	0.0 (0.0)	0.00
Year of Delivery: 2020	0	0	0.00	0 (0.0)	74 (4.5)	-0.31	0.0 (0.0)	0.0 (0.0)	0.00
N MH Diagnoses: 0	244 (70.5)	4,225 (58.3)	0.26	64 (91.4)	1,224 (74.5)	0.46	105.6 (65.1)	105.6 (65.1)	0.00
N MH Diagnoses: 1	32 (9.3)	1,137 (15.7)	-0.20	1 (1.4)	173 (10.5)	-0.39	19.3 (11.9)	19.3 (11.9)	0.00
N MH Diagnoses: 2-3	42 (12.1)	1,047 (14.5)	-0.07	5 (7.1)	144 (8.8)	-0.06	21.4 (13.2)	21.4 (13.2)	0.00
N MH Diagnoses: >=4	28 (8.1)	836 (11.5)	-0.12	0 (0.0)	101 (6.2)	-0.36	15.9 (9.8)	15.9 (9.8)	0.00
N ED Visits: 0	316 (91.3)	6,204 (85.6)	0.18	70 (100.0)	1,552 (94.5)	0.34	144.5 (89.1)	144.5 (89.1)	0.00
N ED Visits: 1	22 (6.4)	704 (9.7)	-0.12	0 (0.0)	58 (3.5)	-0.27	12.9 (8.0)	12.9 (8.0)	0.00
N ED Visits: 2-3	<11	*	-0.12	0 (0.0)	28 (1.7)	-0.19	3.3 (2.1)	3.3 (2.1)	0.00
N ED Visits: >=4	<11	*	-0.04	0 (0.0)	4 (0.2)	-0.07	1.4 (0.9)	1.4 (0.9)	0.00
N MH Hospitalizations: 0	329 (95.1)	6,749 (93.2)	0.08	70 (100.0)	1,609 (98.0)	0.20	152.2 (93.8)	152.2 (93.8)	0.00
N MH Hospitalizations: 1	14 (4.1)	384 (5.3)	-0.06	0 (0.0)	26 (1.6)	-0.18	7.3 (4.5)	7.3 (4.5)	0.00
N MH Hospitalizations: 2-3	<11	*	-0.04	0 (0.0)	7 (0.4)	-0.09	2.7 (1.7)	2.7 (1.7)	0.00
N MH Hospitalizations: >=4	*	*	-0.08	0 (0.0)	0 (0.0)	0.00	0.0 (0.0)	0.0 (0.0)	0.00
N Outpatient Visits: <=3	44 (12.7)	1,012 (14.0)	-0.04	2 (2.9)	99 (6.0)	-0.15	19.4 (12.0)	19.4 (12.0)	0.00
N Outpatient Visits: 4-6	53 (15.3)	1,299 (17.9)	-0.07	16 (22.9)	336 (20.5)	0.06	26.7 (16.5)	26.7 (16.5)	0.00
N Outpatient Visits: 7-10	86 (24.9)	1,788 (24.7)	0.00	21 (30.0)	482 (29.4)	0.01	40.8 (25.2)	40.8 (25.2)	0.00
N Outpatient Visits: >=11	163 (47.1)	3,146 (43.4)	0.07	31 (44.3)	725 (44.2)	0.00	75.3 (46.4)	75.3 (46.4)	0.00
N Psychotropic Medications (Except ASMs), Mean (SD)	0.0 (0.1)	0.0 (0.2)	-0.08	0.0 (0.0)	0.0 (0.2)	-0.24	0.0 (0.1)	0.0 (0.1)	0.00
Alcohol Use Disorder	13 (3.8)	277 (3.8)	0.00	0 (0.0)	21 (1.3)	-0.16	8.8 (5.4)	8.8 (5.4)	0.00
Smoking	39 (11.3)	1,475 (20.4)	-0.25	1 (1.4)	96 (5.9)	-0.24	23.2 (14.3)	23.2 (14.3)	0.00
Substance Use Disorder	34 (9.8)	1,007 (13.9)	-0.13	0 (0.0)	43 (2.6)	-0.23	20.6 (12.7)	20.6 (12.7)	0.00
ADHD	<11	*	-0.16	0 (0.0)	40 (2.4)	-0.22	0.8 (0.5)	0.8 (0.5)	0.00
Adjustment Disorder	<11	*	0.08	0 (0.0)	8 (0.5)	-0.10	4.0 (2.4)	4.0 (2.4)	0.00
Anxiety	49 (14.2)	1,489 (20.6)	-0.17	5 (7.1)	251 (15.3)	-0.26	29.4 (18.1)	29.4 (18.1)	0.00

	Unadjusted, N (column %)						PS Overlap-Weighted, N (column %)		
	MAX/TAF			MarketScan			Both Cohorts Combined		
**Variable**	**Exposed**	**Unexposed**	**SMD**	**Exposed**	**Unexposed**	**SMD**	**Exposed**	**Unexposed**	**SMD**
Behavioral Disorder	<11	*	-0.05	0 (0.0)	5 (0.3)	-0.08	1.6 (1.0)	1.6 (1.0)	0.00
Bipolar Disorder	24 (6.9)	610 (8.4)	-0.06	0 (0.0)	43 (2.6)	-0.23	11.5 (7.1)	11.5 (7.1)	0.00
Chronic Pain	<11	*	-0.12	0 (0.0)	29 (1.8)	-0.19	2.4 (1.5)	2.4 (1.5)	0.00
Chronic Fatigue	33 (9.5)	695 (9.6)	0.00	3 (4.3)	210 (12.8)	-0.31	16.6 (10.2)	16.6 (10.2)	0.00
Depression	58 (16.8)	1,660 (22.9)	-0.15	3 (4.3)	234 (14.3)	-0.35	30.9 (19.0)	30.9 (19.0)	0.00
Migraine/Headache	37 (10.7)	991 (13.7)	-0.09	7 (10.0)	316 (19.2)	-0.26	29.0 (17.9)	29.0 (17.9)	0.00
Neuromuscular Disorder	<11	*	-0.09	1 (1.4)	73 (4.5)	-0.18	5.1 (3.1)	5.1 (3.1)	0.00
Neuropathic Pain	18 (5.2)	463 (6.4)	-0.05	2 (2.9)	169 (10.3)	-0.30	11.1 (6.8)	11.1 (6.8)	0.00
Other MH Disorders	13 (3.8)	358 (4.9)	-0.06	0 (0.0)	50 (3.1)	-0.25	6.0 (3.7)	6.0 (3.7)	0.00
Other Developmental Disorders	<11	*	0.03	0 (0.0)	4 (0.2)	-0.07	2.0 (1.2)	2.0 (1.2)	0.00
Personality Disorder	<11	*	-0.02	1 (1.4)	7 (0.4)	0.10	3.0 (1.8)	3.0 (1.8)	0.00
Psychosis	<11	*	-0.08	0 (0.0)	21 (1.3)	-0.16	1.4 (0.9)	1.4 (0.9)	0.00
Schizophrenia/Schizoaffective Disorder	<11	*	0.02	0 (0.0)	6 (0.4)	-0.09	2.7 (1.7)	2.7 (1.7)	0.00
Sleep Disorder	<11	*	-0.13	2 (2.9)	98 (6.0)	-0.15	6.1 (3.7)	6.1 (3.7)	0.00
Asthma	50 (14.5)	1,144 (15.8)	-0.04	1 (1.4)	140 (8.5)	-0.33	26.3 (16.2)	26.3 (16.2)	0.00
Autoimmune Disorder	<11	*	-0.05	4 (5.7)	96 (5.9)	-0.01	5.0 (3.1)	5.0 (3.1)	0.00
Hyperemesis/NVP	81 (23.4)	2,529 (34.9)	-0.25	4 (5.7)	351 (21.4)	-0.47	40.3 (24.8)	40.3 (24.8)	0.00
Influenca Infection	<11	*	-0.03	0 (0.0)	35 (2.1)	-0.21	2.7 (1.7)	2.7 (1.7)	0.00
Obstetric Comorbidity Score: 0	126 (36.4)	2,642 (36.5)	0.00	19 (27.1)	634 (38.6)	-0.25	52.8 (32.5)	52.8 (32.5)	0.00
Obstetric Comorbidity Score: 1	80 (23.1)	1,590 (22.0)	0.03	23 (32.9)	369 (22.5)	0.23	35.1 (21.7)	35.1 (21.7)	0.00
Obstetric Comorbidity Score: 2	44 (12.7)	1,133 (15.6)	-0.08	16 (22.9)	265 (16.1)	0.17	24.9 (15.3)	24.9 (15.3)	0.00
Obstetric Comorbidity Score: >=3	96 (27.8)	1,880 (26.0)	0.04	12 (17.1)	374 (22.8)	-0.14	49.4 (30.5)	49.4 (30.5)	0.00
Overweight/Obesity	16 (4.6)	811 (11.2)	-0.25	2 (2.9)	185 (11.3)	-0.33	12.7 (7.8)	12.7 (7.8)	0.00
Pre-gestational Diabetes	<11	*	-0.08	0 (0.0)	43 (2.6)	-0.23	3.8 (2.3)	3.8 (2.3)	0.00
Pre-gestational Hypertension	32 (9.3)	835 (11.5)	-0.07	5 (7.1)	170 (10.4)	-0.11	15.7 (9.7)	15.7 (9.7)	0.00
Poor Nutrition	12 (3.5)	247 (3.4)	0.00	0 (0.0)	123 (7.5)	-0.40	7.7 (4.8)	7.7 (4.8)	0.00
Renal Disease	<11	*	-0.10	0 (0.0)	20 (1.2)	-0.16	0.5 (0.3)	0.5 (0.3)	0.00
TORCH Infection	11 (3.2)	277 (3.8)	-0.04	0 (0.0)	36 (2.2)	-0.21	5.1 (3.1)	5.1 (3.1)	0.00
Other Sexually Transmitted Diseases	36 (10.4)	812 (11.2)	-0.03	3 (4.3)	56 (3.4)	0.05	17.1 (10.5)	17.1 (10.5)	0.00
Prenatal Folic Acid Supplementation	246 (71.1)	4,000 (55.2)	0.33	37 (52.9)	527 (32.1)	0.43	102.7 (63.3)	102.7 (63.3)	0.00
Antidepressants	81 (23.4)	1,452 (20.0)	0.08	8 (11.4)	277 (16.9)	-0.16	38.3 (23.6)	38.3 (23.6)	0.00
Antidiabetics	<11	*	-0.07	3 (4.3)	52 (3.2)	0.06	2.0 (1.2)	2.0 (1.2)	0.00
Antihypertensives	20 (5.8)	450 (6.2)	-0.02	5 (7.1)	104 (6.3)	0.03	11.0 (6.8)	11.0 (6.8)	0.00
Antipsychotics	24 (6.9)	430 (5.9)	0.04	0 (0.0)	43 (2.6)	-0.23	11.6 (7.2)	11.6 (7.2)	0.00
Anxiolytics/Hypnotics/Other Sedatives	33 (9.5)	742 (10.2)	-0.02	4 (5.7)	87 (5.3)	0.02	17.2 (10.6)	17.2 (10.6)	0.00
Barbiturates	274 (79.2)	299 (4.1)	2.35	65 (92.9)	56 (3.4)	4.00	88.9 (54.8)	88.9 (54.8)	0.00
Benzodiazepines	48 (13.9)	760 (10.5)	0.10	4 (5.7)	160 (9.7)	-0.15	28.4 (17.5)	28.4 (17.5)	0.00
Corticosteroids	63 (18.2)	1,221 (16.9)	0.04	10 (14.3)	273 (16.6)	-0.06	30.6 (18.9)	30.6 (18.9)	0.00
Insulin	<11	*	-0.05	0 (0.0)	26 (1.6)	-0.18	2.5 (1.6)	2.5 (1.6)	0.00
Lithium	<11	*	0.09	0 (0.0)	2 (0.1)	-0.05	0.5 (0.3)	0.5 (0.3)	0.00
NSAIDs	84 (24.3)	1,747 (24.1)	0.00	6 (8.6)	148 (9.0)	-0.02	44.5 (27.5)	44.5 (27.5)	0.00
Opioid Agonist Therapy	<11	*	-0.02	0 (0.0)	8 (0.5)	-0.10	2.3 (1.4)	2.3 (1.4)	0.00
Prescription Opioids	133 (38.4)	2,514 (34.7)	0.08	15 (21.4)	296 (18.0)	0.09	66.6 (41.1)	66.6 (41.1)	0.00
Progestins	17 (4.9)	459 (6.3)	-0.06	7 (10.0)	231 (14.1)	-0.12	11.5 (7.1)	11.5 (7.1)	0.00
Psychostimulants	<11	*	-0.08	0 (0.0)	44 (2.7)	-0.23	1.0 (0.6)	1.0 (0.6)	0.00

	Unadjusted, N (column %)						PS Overlap-Weighted, N (column %)		
	MAX/TAF			MarketScan			Both Cohorts Combined		
Variable	Exposed	Unexposed	SMD	Exposed	Unexposed	SMD	Exposed	Unexposed	SMD
Teratogens, Known	0	34 (0.5)	-0.10	0 (0.0)	8 (0.5)	-0.10	0.0 (0.0)	0.0 (0.0)	0.00
Teratogens, Suspected	62 (17.9)	1,123 (15.5)	0.06	10 (14.3)	181 (11.0)	0.10	30.4 (18.8)	30.4 (18.8)	0.00
Triptans	<11	*	-0.01	3 (4.3)	60 (3.7)	0.03	8.7 (5.4)	8.7 (5.4)	0.00
Adequacy of Prenatal Care Utilization: Inadequate	172 (49.7)	2,867 (39.6)	0.20	45 (64.3)	860 (52.4)	0.24	81.6 (50.3)	81.6 (50.3)	0.00
Adequacy of Prenatal Care Utilization: Intermediate	44 (12.7)	1,100 (15.2)	-0.07	13 (18.6)	358 (21.8)	-0.08	23.6 (14.6)	23.6 (14.6)	0.00
Adequacy of Prenatal Care Utilization: Adequate	40 (11.6)	1,121 (15.5)	-0.11	6 (8.6)	208 (12.7)	-0.13	17.8 (11.0)	17.8 (11.0)	0.00
Adequacy of Prenatal Care Utilization: Adequate Plus	90 (26.0)	2,157 (29.8)	-0.08	6 (8.6)	216 (13.2)	-0.15	39.2 (24.2)	39.2 (24.2)	0.00
Multiple Gestation	11 (3.2)	165 (2.3)	0.06	1 (1.4)	43 (2.6)	-0.08	4.8 (3.0)	4.8 (3.0)	0.00
Gestational Month of Prenatal Care Initiation: 1	14 (4.1)	390 (5.4)	-0.06	5 (7.1)	110 (6.7)	0.02	7.4 (4.6)	7.4 (4.6)	0.00
Gestational Month of Prenatal Care Initiation: 2	111 (32.1)	2,637 (36.4)	-0.09	31 (44.3)	714 (43.5)	0.02	54.4 (33.6)	54.4 (33.6)	0.00
Gestational Month of Prenatal Care Initiation: 3	74 (21.4)	1,788 (24.7)	-0.08	15 (21.4)	429 (26.1)	-0.11	35.9 (22.1)	35.9 (22.1)	0.00
Gestational Month of Prenatal Care Initiation: 4	42 (12.1)	781 (10.8)	0.04	1 (1.4)	100 (6.1)	-0.25	16.0 (9.9)	16.0 (9.9)	0.00
Gestational Month of Prenatal Care Initiation: 5	31 (9.0)	486 (6.7)	0.08	2 (2.9)	63 (3.8)	-0.05	15.1 (9.3)	15.1 (9.3)	0.00
Gestational Month of Prenatal Care Initiation: 6	15 (4.3)	277 (3.8)	0.03	1 (1.4)	30 (1.8)	-0.03	8.3 (5.1)	8.3 (5.1)	0.00
Gestational Month of Prenatal Care Initiation: 7	<11	*	-0.07	4 (5.7)	30 (1.8)	0.20	3.9 (2.4)	3.9 (2.4)	0.00
Gestational Month of Prenatal Care Initiation: 8	<11	*	0.06	1 (1.4)	19 (1.2)	0.02	5.4 (3.3)	5.4 (3.3)	0.00
Gestational Month of Prenatal Care Initiation: 9	<11	*	-0.03	0 (0.0)	17 (1.0)	-0.14	1.9 (1.2)	1.9 (1.2)	0.00
Gestational Month of Prenatal Care Initiation: None	38 (11.0)	413 (5.7)	0.19	10 (14.3)	130 (7.9)	0.20	13.9 (8.6)	13.9 (8.6)	0.00
Gestational Month of Prenatal Vitamin Initiation: 1	27 (7.8)	468 (6.5)	0.05	6 (8.6)	64 (3.9)	0.19	10.2 (6.3)	10.2 (6.3)	0.00
Gestational Month of Prenatal Vitamin Initiation: 2	95 (27.5)	1,711 (23.6)	0.09	6 (8.6)	188 (11.5)	-0.10	42.2 (26.0)	42.2 (26.0)	0.00
Gestational Month of Prenatal Vitamin Initiation: 3	59 (17.1)	946 (13.1)	0.11	3 (4.3)	104 (6.3)	-0.09	26.4 (16.3)	26.4 (16.3)	0.00
Gestational Month of Prenatal Vitamin Initiation: 4	25 (7.2)	479 (6.6)	0.02	2 (2.9)	39 (2.4)	0.03	10.0 (6.1)	10.0 (6.1)	0.00
Gestational Month of Prenatal Vitamin Initiation: 5	16 (4.6)	286 (4.0)	0.03	1 (1.4)	25 (1.5)	-0.01	7.6 (4.7)	7.6 (4.7)	0.00
Gestational Month of Prenatal Vitamin Initiation: 6	19 (5.5)	197 (2.7)	0.14	1 (1.4)	14 (0.9)	0.05	7.5 (4.6)	7.5 (4.6)	0.00
Gestational Month of Prenatal Vitamin Initiation: 7	<11	*	-0.04	1 (1.4)	25 (1.5)	-0.01	3.1 (1.9)	3.1 (1.9)	0.00
Gestational Month of Prenatal Vitamin Initiation: 8	<11	*	0.05	0 (0.0)	14 (0.9)	-0.13	3.2 (1.9)	3.2 (1.9)	0.00
Gestational Month of Prenatal Vitamin Initiation: 9	<11	*	-0.11	0 (0.0)	31 (1.9)	-0.20	2.2 (1.4)	2.2 (1.4)	0.00
Gestational Month of Prenatal Vitamin Initiation: -1	<11	*	0.06	5 (7.1)	16 (1.0)	0.31	1.8 (1.1)	1.8 (1.1)	0.00
Gestational Month of Prenatal Vitamin Initiation: -2	<11	*	0.09	8 (11.4)	34 (2.1)	0.38	3.2 (2.0)	3.2 (2.0)	0.00
Gestational Month of Prenatal Vitamin Initiation: -3	21 (6.1)	163 (2.3)	0.19	6 (8.6)	74 (4.5)	0.16	7.7 (4.7)	7.7 (4.7)	0.00
Gestational Month of Prenatal Vitamin Initiation: None	51 (14.7)	2,334 (32.2)	-0.42	31 (44.3)	1,014 (61.8)	-0.35	37.3 (23.0)	37.3 (23.0)	0.00
**Lacosamide**									
N Total	150	7,245		69	1,642		185.1	185.1	
Age in Years: <=19	13 (8.7)	1,399 (19.3)	-0.31	0 (0.0)	18 (1.1)	-0.15	11.7 (6.4)	11.7 (6.4)	0.00
Age in Years: 20-24	41 (27.3)	2,612 (36.1)	-0.19	4 (5.8)	149 (9.1)	-0.12	40.3 (21.8)	40.3 (21.8)	0.00
Age in Years: 25-29	49 (32.7)	1,941 (26.8)	0.13	14 (20.3)	558 (34.0)	-0.31	53.2 (28.8)	53.2 (28.8)	0.00
Age in Years: 30-34	30 (20.0)	904 (12.5)	0.20	33 (47.8)	606 (36.9)	0.22	51.1 (27.6)	51.1 (27.6)	0.00
Age in Years: 35-39	*	*	0.17	16 (23.2)	250 (15.2)	0.20	23.9 (12.9)	23.9 (12.9)	0.00
Age in Years: >=40	<11	*	0.13	2 (2.9)	61 (3.7)	-0.05	4.8 (2.6)	4.8 (2.6)	0.00
Race/Ethnicity: Asian or other Pacific Islander	<11	*	0.16	N/A	N/A	N/A	N/A	N/A	N/A
Race/Ethnicity: Black or African American	*	*	-0.22	N/A	N/A	N/A	N/A	N/A	N/A
Race/Ethnicity: Hispanic or Latino	15 (10.0)	963 (13.3)	-0.10	N/A	N/A	N/A	N/A	N/A	N/A
Race/Ethnicity: Unknown or Other	11 (7.3)	340 (4.7)	0.11	N/A	N/A	N/A	N/A	N/A	N/A
Race/Ethnicity: White	89 (59.3)	3,729 (51.5)	0.16	N/A	N/A	N/A	N/A	N/A	N/A
US Region: Midwest	54 (36.0)	2,598 (35.9)	0.00	19 (27.5)	402 (24.5)	0.07	63.0 (34.1)	63.0 (34.1)	0.00

	Unadjusted, N (column %)						PS Overlap-Weighted, N (column %)		
	MAX/TAF			MarketScan			Both Cohorts Combined		
**Variable**	**Exposed**	**Unexposed**	**SMD**	**Exposed**	**Unexposed**	**SMD**	**Exposed**	**Unexposed**	**SMD**
US Region: Northeast	<11	*	-0.31	9 (13.0)	270 (16.4)	-0.10	17.5 (9.5)	17.5 (9.5)	0.00
US Region: South	*	*	0.27	23 (33.3)	692 (42.1)	-0.18	71.7 (38.8)	71.7 (38.8)	0.00
US Region: Unknown	0	0	0.00	6 (8.7)	43 (2.6)	0.26	4.2 (2.3)	4.2 (2.3)	0.00
US Region: West	21 (14.0)	1,251 (17.3)	-0.09	12 (17.4)	235 (14.3)	0.08	28.7 (15.5)	28.7 (15.5)	0.00
Year of Delivery: 2000	*	<11	-0.02	0 (0.0)	0 (0.0)	0.00	0.0 (0.0)	0.0 (0.0)	0.00
Year of Delivery: 2001	0	146 (2.0)	-0.20	0 (0.0)	0 (0.0)	0.00	0.0 (0.0)	0.0 (0.0)	0.00
Year of Delivery: 2002	0	227 (3.1)	-0.25	0 (0.0)	0 (0.0)	0.00	0.0 (0.0)	0.0 (0.0)	0.00
Year of Delivery: 2003	0	262 (3.6)	-0.27	0 (0.0)	0 (0.0)	0.00	0.0 (0.0)	0.0 (0.0)	0.00
Year of Delivery: 2004	0	368 (5.1)	-0.33	0 (0.0)	27 (1.6)	-0.18	0.0 (0.0)	0.0 (0.0)	0.00
Year of Delivery: 2005	0	329 (4.5)	-0.31	0 (0.0)	43 (2.6)	-0.23	0.0 (0.0)	0.0 (0.0)	0.00
Year of Delivery: 2006	0	347 (4.8)	-0.32	0 (0.0)	55 (3.4)	-0.26	0.0 (0.0)	0.0 (0.0)	0.00
Year of Delivery: 2007	0	306 (4.2)	-0.30	0 (0.0)	61 (3.7)	-0.28	0.0 (0.0)	0.0 (0.0)	0.00
Year of Delivery: 2008	0	330 (4.6)	-0.31	0 (0.0)	84 (5.1)	-0.33	0.0 (0.0)	0.0 (0.0)	0.00
Year of Delivery: 2009	<11	*	-0.27	0 (0.0)	132 (8.0)	-0.42	1.0 (0.5)	1.0 (0.5)	0.00
Year of Delivery: 2010	<11	*	0.00	1 (1.5)	147 (9.0)	-0.34	10.6 (5.7)	10.6 (5.7)	0.00
Year of Delivery: 2011	<11	*	-0.05	1 (1.5)	147 (9.0)	-0.34	9.4 (5.1)	9.4 (5.1)	0.00
Year of Delivery: 2012	<11	*	-0.06	4 (5.8)	156 (9.5)	-0.14	16.1 (8.7)	11.7 (6.3)	-0.09
Year of Delivery: 2013	22 (14.7)	625 (8.6)	0.19	7 (10.1)	107 (6.5)	0.13	25.7 (13.9)	25.7 (13.9)	0.00
Year of Delivery: 2014	11 (7.3)	480 (6.6)	0.03	3 (4.4)	102 (6.2)	-0.08	12.2 (6.6)	12.2 (6.6)	0.00
Year of Delivery: 2015	12 (8.0)	383 (5.3)	0.11	9 (13.0)	103 (6.3)	0.23	17.4 (9.4)	17.4 (9.4)	0.00
Year of Delivery: 2016	30 (20.0)	507 (7.0)	0.39	7 (10.1)	105 (6.4)	0.14	29.7 (16.0)	29.7 (16.0)	0.00
Year of Delivery: 2017	24 (16.0)	594 (8.2)	0.24	8 (11.6)	84 (5.1)	0.23	27.2 (14.7)	27.2 (14.7)	0.00
Year of Delivery: 2018	22 (14.7)	425 (5.9)	0.29	8 (11.6)	71 (4.3)	0.27	24.2 (13.1)	24.2 (13.1)	0.00
Year of Delivery: 2019	0	0	0.00	7 (10.1)	85 (5.2)	0.19	4.7 (2.6)	4.7 (2.6)	0.00
Year of Delivery: 2020	0	0	0.00	8 (11.6)	74 (4.5)	0.26	5.7 (3.1)	5.7 (3.1)	0.00
N MH Diagnoses: 0	78 (52.0)	4,225 (58.3)	-0.13	47 (68.1)	1,224 (74.5)	-0.14	107.1 (57.9)	107.1 (57.9)	0.00
N MH Diagnoses: 1	22 (14.7)	1,137 (15.7)	-0.03	5 (7.3)	173 (10.5)	-0.12	23.6 (12.8)	23.6 (12.8)	0.00
N MH Diagnoses: 2-3	24 (16.0)	1,047 (14.5)	0.04	10 (14.5)	144 (8.8)	0.18	28.9 (15.6)	28.9 (15.6)	0.00
N MH Diagnoses: >=4	26 (17.3)	836 (11.5)	0.17	7 (10.1)	101 (6.2)	0.15	25.5 (13.8)	25.5 (13.8)	0.00
N ED Visits: 0	130 (86.7)	6,204 (85.6)	0.03	64 (92.8)	1,552 (94.5)	-0.07	164.7 (89.0)	164.6 (89.0)	0.00
N ED Visits: 1	15 (10.0)	704 (9.7)	0.01	3 (4.4)	58 (3.5)	0.04	15.1 (8.2)	15.1 (8.2)	0.00
N ED Visits: 2-3	<11	*	-0.02	1 (1.5)	28 (1.7)	-0.02	4.7 (2.5)	4.7 (2.5)	0.00
N ED Visits: >=4	*	*	-0.14	1 (1.5)	4 (0.2)	0.13	0.7 (0.4)	0.7 (0.4)	0.00
N MH Hospitalizations: 0	131 (87.3)	6,749 (93.2)	-0.20	66 (95.7)	1,609 (98.0)	-0.13	168.9 (91.3)	168.9 (91.3)	0.00
N MH Hospitalizations: 1	14 (9.3)	384 (5.3)	0.16	2 (2.9)	26 (1.6)	0.09	11.9 (6.4)	11.9 (6.4)	0.00
N MH Hospitalizations: 2-3	<11	*	0.14	0 (0.0)	7 (0.4)	-0.09	3.6 (1.9)	3.6 (1.9)	0.00
N MH Hospitalizations: >=4	*	*	-0.08	1 (1.5)	0 (0.0)	0.17	0.7 (0.4)	0.7 (0.4)	0.00
N Outpatient Visits: <=3	<11	*	-0.45	5 (7.3)	99 (6.0)	0.05	7.5 (4.1)	7.5 (4.1)	0.00
N Outpatient Visits: 4-6	*	*	-0.15	12 (17.4)	336 (20.5)	-0.08	27.4 (14.8)	27.4 (14.8)	0.00
N Outpatient Visits: 7-10	37 (24.7)	1,788 (24.7)	0.00	20 (29.0)	482 (29.4)	-0.01	49.7 (26.9)	49.7 (26.9)	0.00
N Outpatient Visits: >=11	91 (60.7)	3,146 (43.4)	0.35	32 (46.4)	725 (44.2)	0.04	100.5 (54.3)	100.5 (54.3)	0.00
N Psychotropic Medications (Except ASMs), Mean (SD)	0.1 (0.2)	0.0 (0.2)	0.10	0.0 (0.0)	0.0 (0.2)	-0.24	0.0 (0.2)	0.0 (0.2)	0.00
Alcohol Use Disorder	<11	*	-0.16	1 (1.5)	21 (1.3)	0.01	2.6 (1.4)	2.6 (1.4)	0.00
Smoking	35 (23.3)	1,475 (20.4)	0.07	4 (5.8)	96 (5.9)	0.00	33.2 (17.9)	33.2 (17.9)	0.00
Substance Use Disorder	19 (12.7)	1,007 (13.9)	-0.04	4 (5.8)	43 (2.6)	0.16	20.1 (10.8)	20.1 (10.8)	0.00

	Unadjusted, N (column %)						PS Overlap-Weighted, N (column %)		
	MAX/TAF			MarketScan			Both Cohorts Combined		
Variable	Exposed	Unexposed	SMD	Exposed	Unexposed	SMD	Exposed	Unexposed	SMD
ADHD	<11	*	-0.09	1 (1.5)	40 (2.4)	-0.07	2.9 (1.6)	2.9 (1.6)	0.00
Adjustment Disorder	<11	*	-0.03	1 (1.5)	8 (0.5)	0.10	2.3 (1.2)	2.3 (1.2)	0.00
Anxiety	46 (30.7)	1,489 (20.6)	0.23	14 (20.3)	251 (15.3)	0.13	48.6 (26.3)	48.6 (26.3)	0.00
Behavioral Disorder	<11	*	-0.10	0 (0.0)	5 (0.3)	-0.08	1.0 (0.5)	1.0 (0.5)	0.00
Bipolar Disorder	19 (12.7)	610 (8.4)	0.14	1 (1.5)	43 (2.6)	-0.08	16.5 (8.9)	16.5 (8.9)	0.00
Chronic Pain	<11	*	0.15	1 (1.5)	29 (1.8)	-0.03	7.6 (4.1)	7.6 (4.1)	0.00
Chronic Fatigue	23 (15.3)	695 (9.6)	0.17	10 (14.5)	210 (12.8)	0.05	26.7 (14.4)	26.7 (14.4)	0.00
Depression	38 (25.3)	1,660 (22.9)	0.06	14 (20.3)	234 (14.3)	0.16	43.5 (23.5)	43.5 (23.5)	0.00
Migraine/Headache	29 (19.3)	991 (13.7)	0.15	8 (11.6)	316 (19.2)	-0.21	30.7 (16.6)	30.7 (16.6)	0.00
Neuromuscular Disorder	<11	*	-0.02	1 (1.5)	73 (4.5)	-0.18	5.8 (3.1)	5.8 (3.1)	0.00
Neuropathic Pain	13 (8.7)	463 (6.4)	0.09	5 (7.3)	169 (10.3)	-0.11	15.8 (8.5)	15.8 (8.5)	0.00
Other MH Disorders	<11	*	0.05	2 (2.9)	50 (3.1)	-0.01	9.0 (4.9)	9.0 (4.9)	0.00
Other Developmental Disorders	<11	*	0.21	0 (0.0)	4 (0.2)	-0.07	4.9 (2.7)	4.9 (2.7)	0.00
Personality Disorder	<11	*	-0.03	1 (1.5)	7 (0.4)	0.11	2.8 (1.5)	2.8 (1.5)	0.00
Psychosis	<11	*	0.11	2 (2.9)	21 (1.3)	0.11	7.1 (3.8)	7.1 (3.8)	0.00
Schizophrenia/Schizoaffective Disorder	<11	*	-0.06	0 (0.0)	6 (0.4)	-0.09	1.0 (0.5)	1.0 (0.5)	0.00
Sleep Disorder	<11	*	0.07	7 (10.1)	98 (6.0)	0.15	11.4 (6.2)	11.4 (6.2)	0.00
Asthma	19 (12.7)	1,144 (15.8)	-0.09	1 (1.5)	140 (8.5)	-0.33	18.4 (9.9)	18.4 (9.9)	0.00
Autoimmune Disorder	<11	*	0.03	4 (5.8)	96 (5.9)	0.00	7.9 (4.3)	7.9 (4.3)	0.00
Hyperemesis/NVP	47 (31.3)	2,529 (34.9)	-0.08	14 (20.3)	351 (21.4)	-0.03	52.5 (28.4)	52.5 (28.4)	0.00
Influenca Infection	<11	*	0.09	0 (0.0)	35 (2.1)	-0.21	5.6 (3.0)	5.6 (3.0)	0.00
Obstetric Comorbidity Score: 0	49 (32.7)	2,642 (36.5)	-0.08	23 (33.3)	634 (38.6)	-0.11	60.0 (32.4)	60.0 (32.4)	0.00
Obstetric Comorbidity Score: 1	29 (19.3)	1,590 (22.0)	-0.06	13 (18.8)	369 (22.5)	-0.09	36.4 (19.7)	36.4 (19.7)	0.00
Obstetric Comorbidity Score: 2	29 (19.3)	1,133 (15.6)	0.10	18 (26.1)	265 (16.1)	0.24	39.6 (21.4)	39.6 (21.4)	0.00
Obstetric Comorbidity Score: >=3	43 (28.7)	1,880 (26.0)	0.06	15 (21.7)	374 (22.8)	-0.02	49.1 (26.5)	49.1 (26.5)	0.00
Overweight/Obesity	37 (24.7)	811 (11.2)	0.36	16 (23.2)	185 (11.3)	0.32	42.0 (22.7)	42.0 (22.7)	0.00
Pre-gestational Diabetes	<11	*	0.00	2 (2.9)	43 (2.6)	0.02	5.8 (3.1)	5.8 (3.1)	0.00
Pre-gestational Hypertension	14 (9.3)	835 (11.5)	-0.07	6 (8.7)	170 (10.4)	-0.06	16.7 (9.0)	16.7 (9.0)	0.00
Poor Nutrition	17 (11.3)	247 (3.4)	0.31	4 (5.8)	123 (7.5)	-0.07	17.1 (9.2)	17.1 (9.2)	0.00
Renal Disease	<11	*	-0.05	0 (0.0)	20 (1.2)	-0.16	1.0 (0.5)	1.0 (0.5)	0.00
TORCH Infection	<11	*	0.13	3 (4.4)	36 (2.2)	0.12	10.6 (5.7)	10.6 (5.7)	0.00
Other Sexually Transmitted Diseases	23 (15.3)	812 (11.2)	0.12	5 (7.3)	56 (3.4)	0.17	23.9 (12.9)	23.9 (12.9)	0.00
Prenatal Folic Acid Supplementation	120 (80.0)	4,000 (55.2)	0.55	33 (47.8)	527 (32.1)	0.32	124.5 (67.3)	124.5 (67.3)	0.00
Antidepressants	48 (32.0)	1,452 (20.0)	0.27	16 (23.2)	277 (16.9)	0.16	51.1 (27.6)	51.1 (27.6)	0.00
Antidiabetics	<11	*	0.11	3 (4.4)	52 (3.2)	0.06	6.2 (3.4)	6.2 (3.4)	0.00
Antihypertensives	13 (8.7)	450 (6.2)	0.09	1 (1.5)	104 (6.3)	-0.25	11.8 (6.4)	11.8 (6.4)	0.00
Antipsychotics	<11	*	0.03	3 (4.4)	43 (2.6)	0.09	10.6 (5.7)	10.6 (5.7)	0.00
Anxiolytics/Hypnotics/Other Sedatives	19 (12.7)	742 (10.2)	0.08	1 (1.5)	87 (5.3)	-0.21	17.2 (9.3)	17.2 (9.3)	0.00
Barbiturates	12 (8.0)	299 (4.1)	0.16	0 (0.0)	56 (3.4)	-0.27	10.2 (5.5)	10.2 (5.5)	0.00
Benzodiazepines	43 (28.7)	760 (10.5)	0.47	15 (21.7)	160 (9.7)	0.33	43.2 (23.3)	43.2 (23.3)	0.00
Corticosteroids	29 (19.3)	1,221 (16.9)	0.06	14 (20.3)	273 (16.6)	0.09	36.0 (19.4)	36.0 (19.4)	0.00
Insulin	<11	*	-0.10	0 (0.0)	26 (1.6)	-0.18	0.9 (0.5)	0.9 (0.5)	0.00
Lithium	<11	*	0.04	0 (0.0)	2 (0.1)	-0.05	0.8 (0.4)	0.8 (0.4)	0.00
NSAIDs	37 (24.7)	1,747 (24.1)	0.01	7 (10.1)	148 (9.0)	0.04	38.3 (20.7)	38.3 (20.7)	0.00
Opioid Agonist Therapy	<11	*	-0.04	0 (0.0)	8 (0.5)	-0.10	1.0 (0.5)	1.0 (0.5)	0.00

	Unadjusted, N (column %)						PS Overlap-Weighted, N (column %)		
	MAX/TAF			MarketScan			Both Cohorts Combined		
Variable	Exposed	Unexposed	SMD	Exposed	Unexposed	SMD	Exposed	Unexposed	SMD
Prescription Opioids	55 (36.7)	2,514 (34.7)	0.04	13 (18.8)	296 (18.0)	0.02	56.8 (30.7)	56.8 (30.7)	0.00
Progestins	15 (10.0)	459 (6.3)	0.13	12 (17.4)	231 (14.1)	0.09	23.1 (12.5)	23.1 (12.5)	0.00
Psychostimulants	<11	*	0.10	0 (0.0)	44 (2.7)	-0.23	4.1 (2.2)	4.1 (2.2)	0.00
Teratogens, Known	<11	*	0.14	1 (1.5)	8 (0.5)	0.10	2.6 (1.4)	2.6 (1.4)	0.00
Teratogens, Suspected	30 (20.0)	1,123 (15.5)	0.12	5 (7.3)	181 (11.0)	-0.13	29.7 (16.1)	29.7 (16.1)	0.00
Triptans	13 (8.7)	183 (2.5)	0.27	0 (0.0)	60 (3.7)	-0.28	10.2 (5.5)	10.2 (5.5)	0.00
Adequacy of Prenatal Care Utilization: Inadequate	58 (38.7)	2,867 (39.6)	-0.02	31 (44.9)	860 (52.4)	-0.15	76.0 (41.1)	76.0 (41.1)	0.00
Adequacy of Prenatal Care Utilization: Intermediate	18 (12.0)	1,100 (15.2)	-0.09	12 (17.4)	358 (21.8)	-0.11	25.9 (14.0)	25.9 (14.0)	0.00
Adequacy of Prenatal Care Utilization: Adequate	24 (16.0)	1,121 (15.5)	0.01	9 (13.0)	208 (12.7)	0.01	28.6 (15.4)	28.6 (15.4)	0.00
Adequacy of Prenatal Care Utilization: Adequate Plus	50 (33.3)	2,157 (29.8)	0.08	17 (24.6)	216 (13.2)	0.30	54.7 (29.5)	54.7 (29.5)	0.00
Multiple Gestation	<11	*	-0.02	4 (5.8)	43 (2.6)	0.16	6.1 (3.3)	6.1 (3.3)	0.00
Gestational Month of Prenatal Care Initiation: 1	<11	*	0.05	4 (5.8)	110 (6.7)	-0.04	11.6 (6.3)	11.6 (6.3)	0.00
Gestational Month of Prenatal Care Initiation: 2	65 (43.3)	2,637 (36.4)	0.14	40 (58.0)	714 (43.5)	0.29	87.7 (47.4)	87.7 (47.4)	0.00
Gestational Month of Prenatal Care Initiation: 3	36 (24.0)	1,788 (24.7)	-0.02	12 (17.4)	429 (26.1)	-0.21	41.8 (22.6)	41.8 (22.6)	0.00
Gestational Month of Prenatal Care Initiation: 4	<11	*	-0.15	4 (5.8)	100 (6.1)	-0.01	11.5 (6.2)	11.5 (6.2)	0.00
Gestational Month of Prenatal Care Initiation: 5	<11	*	-0.03	5 (7.3)	63 (3.8)	0.15	11.9 (6.4)	11.9 (6.4)	0.00
Gestational Month of Prenatal Care Initiation: 6	<11	*	-0.03	0 (0.0)	30 (1.8)	-0.19	4.0 (2.2)	4.0 (2.2)	0.00
Gestational Month of Prenatal Care Initiation: 7	<11	*	-0.05	0 (0.0)	30 (1.8)	-0.19	2.9 (1.6)	2.9 (1.6)	0.00
Gestational Month of Prenatal Care Initiation: 8	<11	*	-0.11	1 (1.5)	19 (1.2)	0.03	1.7 (0.9)	1.7 (0.9)	0.00
Gestational Month of Prenatal Care Initiation: 9	<11	*	0.01	1 (1.5)	17 (1.0)	0.04	3.2 (1.7)	3.2 (1.7)	0.00
Gestational Month of Prenatal Care Initiation: None	<11	*	-0.02	2 (2.9)	130 (7.9)	-0.22	8.6 (4.6)	8.6 (4.6)	0.00
Gestational Month of Prenatal Vitamin Initiation: 1	22 (14.7)	468 (6.5)	0.27	3 (4.4)	64 (3.9)	0.02	20.2 (10.9)	20.2 (10.9)	0.00
Gestational Month of Prenatal Vitamin Initiation: 2	39 (26.0)	1,711 (23.6)	0.06	7 (10.1)	188 (11.5)	-0.04	42.2 (22.8)	42.2 (22.8)	0.00
Gestational Month of Prenatal Vitamin Initiation: 3	21 (14.0)	946 (13.1)	0.03	5 (7.3)	104 (6.3)	0.04	21.2 (11.4)	21.2 (11.4)	0.00
Gestational Month of Prenatal Vitamin Initiation: 4	<11	*	-0.03	3 (4.4)	39 (2.4)	0.11	9.8 (5.3)	9.8 (5.3)	0.00
Gestational Month of Prenatal Vitamin Initiation: 5	<11	*	0.00	1 (1.5)	25 (1.5)	-0.01	6.2 (3.4)	6.2 (3.4)	0.00
Gestational Month of Prenatal Vitamin Initiation: 6	<11	*	0.04	2 (2.9)	14 (0.9)	0.15	5.8 (3.2)	5.8 (3.2)	0.00
Gestational Month of Prenatal Vitamin Initiation: 7	0	166 (2.3)	-0.22	0 (0.0)	25 (1.5)	-0.18	0.0 (0.0)	0.0 (0.0)	0.00
Gestational Month of Prenatal Vitamin Initiation: 8	<11	*	0.00	0 (0.0)	14 (0.9)	-0.13	1.9 (1.0)	1.9 (1.0)	0.00
Gestational Month of Prenatal Vitamin Initiation: 9	<11	*	-0.07	5 (7.3)	31 (1.9)	0.26	6.2 (3.4)	6.2 (3.4)	0.00
Gestational Month of Prenatal Vitamin Initiation: -1	<11	*	0.10	7 (10.1)	16 (1.0)	0.41	5.8 (3.1)	5.8 (3.1)	0.00
Gestational Month of Prenatal Vitamin Initiation: -2	<11	*	0.15	0 (0.0)	34 (2.1)	-0.21	5.5 (3.0)	5.5 (3.0)	0.00
Gestational Month of Prenatal Vitamin Initiation: -3	17 (11.3)	163 (2.3)	0.37	8 (11.6)	74 (4.5)	0.26	17.1 (9.2)	17.1 (9.2)	0.00
Gestational Month of Prenatal Vitamin Initiation: None	17 (11.3)	2,334 (32.2)	-0.52	28 (40.6)	1,014 (61.8)	-0.43	43.3 (23.4)	43.3 (23.4)	0.00

Abbreviations: ADHD, attention deficit/hyperactivity disorder; ASM, antiseizure medication; ED, emergency department; MarketScan, Merative MarketScan Commercial Claims and Encounters Database; MAX/TAF, Medicaid Analytic eXtract/Transformed Medicaid Statistical Information System Analytic Files; MH, mental health; N, number; N/A, not available; NVP, nausea and vomiting in pregnancy; NSAIDs, non-steroidal anti-inflammatory drugs; PS, propensity score; SD, standard deviation; SMD, standardized mean difference; TORCH, toxoplasmosis, other agents, rubella, cytomegalovirus, herpes.
Individual medications are sorted by the number of exposed pregnancies in both cohorts combined (from top [highest] to bottom [lowest]).
Patient characteristics are presented as column % for binary or categorical variables and as mean (SD) for continuous variables.
Standardized mean difference[1] was used to assess balance of covariates between the respective exposure group and the unexposed reference.

For continuous variables, standardized mean difference is calculated as $\frac{(\overline{x}_{treatment} - \overline{x}_{control})}{\sqrt{\frac{s^2_{treatment} + s^2_{control}}{2}}}$, where $\overline{x}$ denotes the sample mean and s denotes the standard deviation of the covariate. For binary/categorical variables, standardized mean difference is calculated as $\frac{(\hat{p}_{treatment} - \hat{p}_{control})}{\sqrt{\frac{\hat{p}_{treatment}(1-\hat{p}_{treatment}) + \hat{p}_{control}(1-\hat{p}_{control})}{2}}}$, where $\hat{p}$ denotes the prevalence of the variable in treatment and control subjects, respectively.*

Absolute standardized mean differences <0.1 were considered well-balanced.

Race/ethnicity was information only available in MAX/TAF. Race and ethnicity information was not available for the commerically (MarketScan) insured study cohort. Race/ethnicity was therefore not adjusted for in PS-weighted analyses. Information on race/ethnicity is based on data submitted to the Centers for Medicare & Medicaid Services (CMS) by each state from collected and coded Medicaid applications. The category other/unknown included American Indian/Alaska Native, Native Hawaiian/other Pacific Islander, Hispanic/Latino, more than 1 race, and unknown; category Hispanic/Latino included this ethnicity with missing race information, whereas other/unknown included Hispanic/Latino with 1 or more races.

Cell counts <11 in MAX/TAF are suppressed in accordance with CMS' cell suppression policies.

*Cell size was suppressed in order to avoid back-calculation.

Reference:

[1]Austin PC. Balance diagnostics for comparing the distribution of baseline covariates between treatment groups in propensity-score matched samples. Stat Med. Nov 10 2009;28(25):3083-107. doi:10.1002/sim.3697

eTable 6. Length of Follow-Up in Years Since Birth in Children Born to Mothers with Epilepsy

A) Distribution of Length of Follow-up Stratified by Prenatal Exposure to the Specific Antiseizure Medication of Interest & Database.

Cohort	Exposure	N	Mean	SD	Minimum	Maximum	1st Quartile	Median	3rd Quartile
MAX/TAF	Overall	17590	3.5	3.3	0.0	18.0	1.1	2.3	4.9
	Unexposed	7245	3.4	3.1	0.0	18.0	1.1	2.2	4.6
	Levetiracetam	3728	2.7	2.4	0.0	16.8	1.0	1.9	3.7
	Lamotrigine	2323	3.2	2.9	0.0	16.8	1.1	2.2	4.5
	Carbamazepine	1406	4.6	4.0	0.0	18.0	1.4	3.1	6.7
	Phenytoin	1431	5.0	4.3	0.0	18.0	1.4	3.6	7.7
	Topiramate	730	4.0	3.4	0.2	16.0	1.3	2.9	5.7
	Oxcarbazepine	572	3.8	3.5	0.0	16.7	1.3	2.5	5.4
	Valproate	687	4.6	4.2	0.0	17.8	1.2	3.1	7.0
	Zonisamide	285	3.5	3.3	0.3	15.8	1.1	2.3	5.5
	Phenobarbital	346	5.2	4.6	0.3	18.0	1.1	3.2	8.7
	Lacosamide	150	2.0	1.4	0.0	6.1	0.9	1.7	2.6
MarketScan	Overall	6290	3.1	3.0	0.0	18.0	0.9	1.9	4.1
	Unexposed	1642	2.8	2.9	0.0	17.2	0.8	1.8	3.8
	Levetiracetam	1533	2.8	2.8	0.3	17.6	0.9	1.9	3.7
	Lamotrigine	1976	3.2	3.0	0.3	18.0	0.9	2.1	4.3
	Carbamazepine	518	3.6	3.5	0.3	17.9	1.0	2.2	5.4
	Phenytoin	164	3.9	4.0	0.3	16.8	1.1	2.4	5.3
	Topiramate	306	3.1	3.3	0.3	16.7	0.9	1.7	4.2
	Oxcarbazepine	273	2.9	2.8	0.3	15.5	0.9	1.9	4.1
	Valproate	113	3.7	3.8	0.3	16.1	1.1	2.1	4.8
	Zonisamide	161	2.7	2.7	0.3	12.9	0.9	1.7	3.3
	Phenobarbital	70	4.6	4.3	0.3	17.9	1.5	3.1	7.0
	Lacosamide	69	2.4	2.1	0.3	9.3	0.8	1.6	3.7

B) Number of Children Remaining Under Follow-up at Specific Age, Stratified by Database.

Age (Years)	N Children in Medicaid	N Children in MarketScan
0	17590	6290
1	14327	4505
2	9669	3093
3	6923	2257
4	5377	1622
5	4249	1256
6	3305	920
7	2572	687
8	1982	534
9	1521	384
10	1136	263
11	808	195
12	554	144
13	375	95
14	263	63
15	153	41

Age (Years)	N Children in Medicaid	N Children in MarketScan
16	75	21
17	33	8
18	<11	0
19	0	0

Abbreviations: MarketScan, Merative MarketScan Commercial Claims and Encounters Database; MAX/TAF, Medicaid Analytic eXtract/Transformed Medicaid Statistical Information System Analytic Files; N, number; SD, standard deviation.

eTable 7. Cumulative Incidence in % at Age 8 Years and Hazard Ratios (95% Confidence Intervals) of Any Neurodevelopmental Disorder and Individual Neurodevelopmental Disorders in Children Prenatally Exposed to the Specific Antiseizure Medication of Interest. Results from Crude and Adjusted Main Analyses and from Adjusted Sensitivity Analyses are Presented for Publicly and Commercially Insured Children Combined.

Outcome	Exposure	Analysis	N Cases/ Total		Cum. Incidence in % (95% CI) at Age 8 Years		HR (95% CI)
			Among Exposed	Among Unexposed	Among Exposed	Among Unexposed	
Any NDD	levetiracetam	Main (Crude)	413 / 5261	872 / 8887	28.76 (25.63 - 32.18)	34.31 (32.00 - 36.73)	0.97 (0.87 - 1.09)
Any NDD	levetiracetam	Main (Adjusted)	413 / 5261	872 / 8887	29.46 (26.14 - 33.09)	33.78 (31.09 - 36.62)	0.93 (0.82 - 1.06)
Any NDD	levetiracetam	Monotherapy	280 / 3852	872 / 8887	28.42 (24.61 - 32.68)	33.48 (30.67 - 36.48)	0.91 (0.79 - 1.06)
Any NDD	levetiracetam	Polytherapy (no Valproate)	122 / 1343	872 / 8887	28.07 (22.66 - 34.45)	33.99 (30.80 - 37.41)	0.95 (0.78 - 1.17)
Any NDD	levetiracetam	Strict Monotherapy	232 / 3360	872 / 8887	27.65 (23.44 - 32.44)	32.84 (29.96 - 35.92)	0.91 (0.78 - 1.07)
Any NDD	levetiracetam	>=2 Rx	320 / 4067	872 / 8887	30.03 (26.21 - 34.25)	33.45 (30.68 - 36.40)	0.93 (0.80 - 1.07)
Any NDD	levetiracetam	Active Comparator	431 / 5487	701 / 7381	28.89 (25.54 - 32.58)	31.53 (28.04 - 35.34)	0.97 (0.84 - 1.13)
Any NDD	levetiracetam	High Dose	248 / 3036	872 / 8887	30.66 (26.44 - 35.38)	33.73 (30.83 - 36.83)	0.99 (0.85 - 1.15)
Any NDD	levetiracetam	Low Dose	137 / 1879	872 / 8887	25.97 (21.14 - 31.66)	33.90 (31.09 - 36.90)	0.83 (0.69 - 1.01)
Any NDD	levetiracetam	No Child Epilepsy	325 / 5,261	705 / 8,887	21.71 (18.85 - 24.93)	28.09 (25.61 - 30.76)	0.85 (0.73 - 0.98)
Any NDD	levetiracetam	Accounting for Censoring	413 / 5261	872 / 8887	**	**	0.94 (0.82 - 1.08)
Any NDD	lamotrigine	Main (Crude)	404 / 4299	872 / 8887	30.89 (27.79 - 34.25)	34.31 (32.00 - 36.73)	0.95 (0.85 - 1.07)
Any NDD	lamotrigine	Main (Adjusted)	404 / 4299	872 / 8887	34.49 (30.98 - 38.27)	32.67 (29.98 - 35.54)	1.05 (0.92 - 1.19)
Any NDD	lamotrigine	Monotherapy	289 / 3200	872 / 8887	33.00 (29.10 - 37.27)	31.87 (29.05 - 34.89)	1.02 (0.88 - 1.19)
Any NDD	lamotrigine	Polytherapy (no Valproate)	106 / 1043	872 / 8887	38.31 (31.17 - 46.45)	34.37 (31.18 - 37.80)	1.08 (0.87 - 1.34)
Any NDD	lamotrigine	Strict Monotherapy	248 / 2864	872 / 8887	32.58 (28.38 - 37.22)	31.02 (28.12 - 34.14)	1.01 (0.86 - 1.18)
Any NDD	lamotrigine	>=2 Rx	330 / 3606	872 / 8887	32.50 (28.77 - 36.57)	32.21 (29.41 - 35.20)	1.01 (0.88 - 1.17)
Any NDD	lamotrigine	High Dose	226 / 2542	872 / 8887	29.79 (25.67 - 34.41)	31.32 (28.32 - 34.56)	0.98 (0.83 - 1.16)
Any NDD	lamotrigine	Low Dose	159 / 1489	872 / 8887	38.96 (33.19 - 45.35)	34.21 (31.40 - 37.20)	1.10 (0.93 - 1.32)
Any NDD	lamotrigine	No Child Epilepsy	325 / 4,299	705 / 8,887	27.11 (23.90 - 30.65)	26.83 (24.36 - 29.50)	0.99 (0.85 - 1.14)
Any NDD	lamotrigine	Accounting for Censoring	404 / 4299	872 / 8887	**	**	1.01 (0.88 - 1.17)
Any NDD	carbamazepine	Main (Crude)	275 / 1924	872 / 8887	35.65 (31.76 - 39.86)	34.31 (32.00 - 36.73)	1.03 (0.90 - 1.18)
Any NDD	carbamazepine	Main (Adjusted)	275 / 1924	872 / 8887	37.00 (32.88 - 41.45)	31.61 (28.81 - 34.61)	1.16 (1.00 - 1.35)
Any NDD	carbamazepine	Monotherapy	205 / 1456	872 / 8887	37.51 (32.82 - 42.64)	31.14 (28.26 - 34.23)	1.19 (1.00 - 1.41)
Any NDD	carbamazepine	Polytherapy (no Valproate)	63 / 435	872 / 8887	34.14 (26.48 - 43.27)	31.33 (27.95 - 35.01)	1.11 (0.85 - 1.46)
Any NDD	carbamazepine	Strict Monotherapy	184 / 1333	872 / 8887	37.48 (32.58 - 42.86)	30.69 (27.78 - 33.84)	1.21 (1.02 - 1.45)
Any NDD	carbamazepine	>=2 Rx	222 / 1545	872 / 8887	37.99 (33.33 - 43.07)	31.07 (28.17 - 34.19)	1.18 (1.00 - 1.39)
Any NDD	carbamazepine	Active Comparator	312 / 2211	701 / 7381	35.77 (31.57 - 40.36)	31.50 (27.94 - 35.39)	1.19 (1.00 - 1.41)
Any NDD	carbamazepine	High Dose	196 / 1200	872 / 8887	38.17 (33.30 - 43.49)	31.13 (28.14 - 34.36)	1.27 (1.07 - 1.51)
Any NDD	carbamazepine	Low Dose	63 / 508	872 / 8887	31.81 (24.87 - 40.10)	31.07 (28.03 - 34.36)	0.97 (0.74 - 1.26)
Any NDD	carbamazepine	No Child Epilepsy	201 / 1,924	705 / 8,887	27.77 (24.01 - 31.99)	25.23 (22.69 - 28.00)	1.05 (0.88 - 1.25)
Any NDD	carbamazepine	Accounting for Censoring	275 / 1924	872 / 8887	**	**	1.15 (0.98 - 1.34)
Any NDD	phenytoin	Main (Crude)	237 / 1595	872 / 8887	30.90 (27.22 - 34.95)	34.31 (32.00 - 36.73)	0.89 (0.77 - 1.03)
Any NDD	phenytoin	Main (Adjusted)	237 / 1595	872 / 8887	31.33 (27.49 - 35.56)	34.35 (31.50 - 37.38)	0.91 (0.78 - 1.07)
Any NDD	phenytoin	Monotherapy	171 / 1177	872 / 8887	30.46 (26.20 - 35.24)	33.96 (31.12 - 36.98)	0.87 (0.73 - 1.04)
Any NDD	phenytoin	Polytherapy (no Valproate)	59 / 382	872 / 8887	33.69 (25.66 - 43.40)	34.51 (30.96 - 38.35)	1.07 (0.81 - 1.41)
Any NDD	phenytoin	Strict Monotherapy	160 / 1060	872 / 8887	31.21 (26.73 - 36.23)	33.58 (30.67 - 36.68)	0.91 (0.76 - 1.10)
Any NDD	phenytoin	>=2 Rx	174 / 1172	872 / 8887	30.68 (26.36 - 35.52)	33.67 (30.72 - 36.83)	0.91 (0.76 - 1.09)
Any NDD	phenytoin	Active Comparator	253 / 1702	701 / 7381	30.13 (25.75 - 35.06)	34.33 (29.77 - 39.37)	0.90 (0.73 - 1.11)
Any NDD	phenytoin	High Dose	123 / 789	872 / 8887	32.21 (27.00 - 38.14)	33.36 (30.28 - 36.66)	0.93 (0.76 - 1.13)
Any NDD	phenytoin	Low Dose	97 / 642	872 / 8887	30.32 (24.72 - 36.84)	34.74 (31.68 - 38.00)	0.92 (0.74 - 1.15)
Any NDD	phenytoin	No Child Epilepsy	182 / 1,595	705 / 8,887	24.31 (20.84 - 28.26)	27.32 (24.71 - 30.15)	0.90 (0.75 - 1.07)
Any NDD	phenytoin	Accounting for Censoring	237 / 1595	872 / 8887	**	**	0.90 (0.77 - 1.06)
Any NDD	topiramate	Main (Crude)	145 / 1036	872 / 8887	37.98 (32.30 - 44.29)	34.31 (32.00 - 36.73)	1.22 (1.02 - 1.45)

Outcome	Exposure	Analysis	N Cases/ Total		Cum. Incidence in % (95% CI) at Age 8 Years		HR (95% CI)
			Among Exposed	Among Unexposed	Among Exposed	Among Unexposed	
Any NDD	topiramate	Main (Adjusted)	145 / 1036	872 / 8887	37.97 (32.21 - 44.39)	36.29 (33.30 - 39.45)	1.13 (0.93 - 1.36)
Any NDD	topiramate	Monotherapy	85 / 626	872 / 8887	33.90 (27.30 - 41.58)	36.58 (33.46 - 39.91)	1.05 (0.83 - 1.32)
Any NDD	topiramate	Polytherapy (no Valproate)	55 / 383	872 / 8887	48.32 (36.89 - 61.21)	36.07 (32.47 - 39.95)	1.23 (0.93 - 1.64)
Any NDD	topiramate	Strict Monotherapy	73 / 565	872 / 8887	32.73 (25.88 - 40.84)	36.11 (32.97 - 39.45)	1.05 (0.82 - 1.34)
Any NDD	topiramate	>=2 Rx	102 / 781	872 / 8887	33.61 (27.41 - 40.78)	35.57 (32.44 - 38.90)	1.03 (0.83 - 1.28)
Any NDD	topiramate	Active Comparator	314 / 2494	701 / 7381	36.73 (32.41 - 41.43)	36.45 (33.00 - 40.14)	1.15 (0.98 - 1.34)
Any NDD	topiramate	High Dose	97 / 617	872 / 8887	40.43 (33.28 - 48.48)	35.27 (31.99 - 38.78)	1.18 (0.95 - 1.48)
Any NDD	topiramate	Low Dose	46 / 363	872 / 8887	35.82 (26.53 - 47.16)	37.82 (34.47 - 41.38)	1.11 (0.82 - 1.50)
Any NDD	topiramate	No Child Epilepsy	115 / 1,036	705 / 8,887	29.66 (24.41 - 35.75)	29.70 (26.92 - 32.71)	1.08 (0.88 - 1.34)
Any NDD	topiramate	Accounting for Censoring	145 / 1036	872 / 8887	**	**	1.14 (0.93 - 1.39)
Any NDD	oxcarbazepine	Main (Crude)	94 / 845	872 / 8887	35.64 (29.09 - 43.16)	34.31 (32.00 - 36.73)	1.05 (0.85 - 1.30)
Any NDD	oxcarbazepine	Main (Adjusted)	94 / 845	872 / 8887	36.57 (29.82 - 44.31)	34.42 (31.28 - 37.78)	1.09 (0.87 - 1.36)
Any NDD	oxcarbazepine	Monotherapy	61 / 579	872 / 8887	31.30 (24.24 - 39.81)	33.95 (30.68 - 37.48)	1.03 (0.79 - 1.34)
Any NDD	oxcarbazepine	Polytherapy (no Valproate)	28 / 254	872 / 8887	45.67 (31.47 - 62.66)	35.59 (31.52 - 40.02)	1.09 (0.74 - 1.59)
Any NDD	oxcarbazepine	Strict Monotherapy	51 / 522	872 / 8887	31.32 (23.57 - 40.86)	33.14 (29.77 - 36.78)	1.04 (0.78 - 1.39)
Any NDD	oxcarbazepine	>=2 Rx	70 / 685	872 / 8887	34.59 (27.03 - 43.54)	33.95 (30.67 - 37.49)	1.02 (0.79 - 1.31)
Any NDD	oxcarbazepine	Active Comparator	150 / 1355	701 / 7381	33.46 (28.10 - 39.52)	35.08 (31.91 - 38.47)	0.97 (0.80 - 1.17)
Any NDD	oxcarbazepine	High Dose	52 / 454	872 / 8887	41.67 (31.62 - 53.44)	33.44 (29.84 - 37.34)	1.18 (0.89 - 1.58)
Any NDD	oxcarbazepine	Low Dose	39 / 332	872 / 8887	31.07 (22.65 - 41.67)	35.10 (31.63 - 38.85)	0.95 (0.69 - 1.32)
Any NDD	oxcarbazepine	No Child Epilepsy	77 / 845	705 / 8,887	29.22 (23.09 - 36.56)	28.25 (25.37 - 31.39)	1.08 (0.84 - 1.38)
Any NDD	oxcarbazepine	Accounting for Censoring	94 / 845	872 / 8887	**	**	1.17 (0.92 - 1.48)
Any NDD	valproate	Main (Crude)	144 / 800	872 / 8887	41.41 (35.58 - 47.78)	34.31 (32.00 - 36.73)	1.32 (1.11 - 1.58)
Any NDD	valproate	Main (Adjusted)	144 / 800	872 / 8887	41.19 (35.28 - 47.68)	35.08 (32.00 - 38.36)	1.29 (1.06 - 1.56)
Any NDD	valproate	Monotherapy	98 / 561	872 / 8887	39.48 (32.70 - 47.11)	35.11 (31.87 - 38.58)	1.24 (0.99 - 1.56)
Any NDD	valproate	Strict Monotherapy	90 / 515	872 / 8887	39.55 (32.57 - 47.41)	34.36 (31.10 - 37.85)	1.25 (0.99 - 1.58)
Any NDD	valproate	>=2 Rx	117 / 568	872 / 8887	46.00 (38.94 - 53.67)	35.20 (31.89 - 38.74)	1.54 (1.25 - 1.89)
Any NDD	valproate	Active Comparator	227 / 1392	701 / 7381	37.79 (32.92 - 43.12)	37.37 (33.58 - 41.45)	1.18 (0.99 - 1.42)
Any NDD	valproate	High Dose	102 / 509	872 / 8887	41.82 (34.90 - 49.52)	34.96 (31.68 - 38.47)	1.37 (1.09 - 1.70)
Any NDD	valproate	Low Dose	33 / 209	872 / 8887	38.01 (27.06 - 51.56)	34.00 (30.59 - 37.69)	1.14 (0.80 - 1.63)
Any NDD	valproate	No Child Epilepsy	97 / 800	705 / 8,887	27.51 (22.40 - 33.51)	27.76 (24.95 - 30.81)	1.09 (0.87 - 1.37)
Any NDD	valproate	Accounting for Censoring	144 / 800	872 / 8887	**	**	1.24 (1.02 - 1.51)
Any NDD	zonisamide	Main (Crude)	55 / 446	872 / 8887	42.64 (31.86 - 55.31)	34.31 (32.00 - 36.73)	1.33 (1.01 - 1.74)
Any NDD	zonisamide	Main (Adjusted)	55 / 446	872 / 8887	43.03 (32.18 - 55.74)	33.02 (29.11 - 37.29)	1.41 (1.06 - 1.88)
Any NDD	zonisamide	Monotherapy	26 / 225	840 / 8593	38.59 (24.25 - 57.51)	32.01 (27.89 - 36.56)	1.31 (0.88 - 1.95)
Any NDD	zonisamide	Polytherapy (no Valproate)	28 / 211	769 / 8007	47.74 (32.53 - 65.71)	34.26 (29.16 - 39.98)	1.56 (1.05 - 2.30)
Any NDD	zonisamide	Strict Monotherapy	20 / 207	840 / 8593	27.95 (15.95 - 46.14)	30.99 (26.66 - 35.84)	1.15 (0.73 - 1.81)
Any NDD	zonisamide	>=2 Rx	47 / 355	872 / 8887	43.69 (31.96 - 57.54)	32.93 (28.65 - 37.66)	1.55 (1.14 - 2.11)
Any NDD	zonisamide	Active Comparator	59 / 508	701 / 7381	40.58 (30.41 - 52.63)	33.13 (29.16 - 37.49)	1.24 (0.93 - 1.65)
Any NDD	zonisamide	High Dose	33 / 276	872 / 8887	41.20 (27.72 - 58.04)	33.05 (28.88 - 37.66)	1.31 (0.91 - 1.88)
Any NDD	zonisamide	Low Dose	****	****	****	****	****
Any NDD	zonisamide	No Child Epilepsy	44 / 446	705 / 8,887	33.93 (24.27 - 46.09)	26.45 (22.94 - 30.39)	1.35 (0.98 - 1.85)
Any NDD	zonisamide	Accounting for Censoring	55 / 446	872 / 8887	**	**	1.32 (0.98 - 1.79)
Any NDD	phenobarbital	Main (Crude)	60 / 416	872 / 8887	27.13 (20.80 - 34.90)	34.31 (32.00 - 36.73)	0.83 (0.64 - 1.08)
Any NDD	phenobarbital	Main (Adjusted)	60 / 416	872 / 8887	27.56 (18.98 - 38.99)	29.10 (22.30 - 37.41)	0.92 (0.61 - 1.39)
Any NDD	phenobarbital	Monotherapy	41 / 255	836 / 8148	30.18 (19.64 - 44.59)	28.98 (20.82 - 39.45)	0.95 (0.59 - 1.54)
Any NDD	phenobarbital	Polytherapy (no Valproate)	****	****	****	****	****
Any NDD	phenobarbital	Strict Monotherapy	****	****	****	****	****
Any NDD	phenobarbital	>=2 Rx	47 / 319	872 / 8887	26.66 (16.10 - 42.15)	24.44 (15.96 - 36.34)	1.00 (0.59 - 1.68)

Outcome	Exposure	Analysis	N Cases/ Total		Cum. Incidence in % (95% CI) at Age 8 Years		HR (95% CI)
			Among Exposed	Among Unexposed	Among Exposed	Among Unexposed	
Any NDD	phenobarbital	Active Comparator	131 / 934	701 / 7381	32.22 (25.60 - 40.04)	39.66 (33.46 - 46.55)	0.87 (0.64 - 1.17)
Any NDD	phenobarbital	High Dose	33 / 221	872 / 8887	20.54 (11.40 - 35.39)	26.13 (16.68 - 39.52)	0.85 (0.47 - 1.54)
Any NDD	phenobarbital	Low Dose	****	****	****	****	****
Any NDD	phenobarbital	No Child Epilepsy	42 / 416	705 / 8,887	17.22 (10.60 - 27.31)	23.01 (17.03 - 30.66)	0.79 (0.48 - 1.28)
Any NDD	phenobarbital	Accounting for Censoring	60 / 416	872 / 8887	**	**	0.95 (0.63 - 1.41)
Any NDD	lacosamide	Main (Crude)	13 / 219	872 / 8887	22.70 (12.23 - 39.84)	34.31 (32.00 - 36.73)	1.16 (0.67 - 2.02)
Any NDD	lacosamide	Main (Adjusted)	13 / 219	462 / 6175	24.28 (13.26 - 41.94)	25.22 (18.19 - 34.34)	1.03 (0.57 - 1.84)
Any NDD	lacosamide	Monotherapy	****	****	****	****	****
Any NDD	lacosamide	Polytherapy (no Valproate)	****	****	****	****	****
Any NDD	lacosamide	Strict Monotherapy	****	****	****	****	****
Any NDD	lacosamide	>=2 Rx	****	****	****	****	****
Any NDD	lacosamide	Active Comparator	14 / 226	701 / 7381	25.98 (14.52 - 43.83)	37.28 (31.27 - 44.04)	0.97 (0.55 - 1.71)
Any NDD	lacosamide	High Dose	****	****	****	****	****
Any NDD	lacosamide	Low Dose	****	****	****	****	****
Any NDD	lacosamide	No Child Epilepsy	*** / 219	391 / 6,175	18.06 (9.02 - 34.27)	22.70 (15.96 - 31.70)	0.88 (0.46 - 1.70)
Any NDD	lacosamide	Accounting for Censoring	13 / 219	462 / 6175	**	**	1.17 (0.65 - 2.09)
Speech/Language Disorder	levetiracetam	Main (Crude)	261 / 5261	456 / 8887	15.33 (13.31 - 17.62)	14.46 (13.04 - 16.02)	1.14 (0.98 - 1.33)
Speech/Language Disorder	levetiracetam	Main (Adjusted)	261 / 5261	456 / 8887	14.99 (12.83 - 17.48)	15.80 (14.07 - 17.71)	0.97 (0.82 - 1.15)
Speech/Language Disorder	levetiracetam	Monotherapy	179 / 3852	456 / 8887	14.93 (12.36 - 17.98)	15.94 (14.14 - 17.95)	0.95 (0.78 - 1.14)
Speech/Language Disorder	levetiracetam	Polytherapy (no Valproate)	76 / 1343	456 / 8887	14.79 (11.37 - 19.12)	15.77 (13.73 - 18.07)	1.02 (0.78 - 1.32)
Speech/Language Disorder	levetiracetam	Strict Monotherapy	151 / 3360	456 / 8887	14.13 (11.56 - 17.22)	15.65 (13.82 - 17.70)	0.96 (0.78 - 1.17)
Speech/Language Disorder	levetiracetam	>=2 Rx	202 / 4067	456 / 8887	15.24 (12.77 - 18.15)	15.88 (14.09 - 17.87)	0.95 (0.79 - 1.15)
Speech/Language Disorder	levetiracetam	Active Comparator	270 / 5487	392 / 7381	15.10 (12.93 - 17.60)	15.12 (12.91 - 17.67)	0.99 (0.82 - 1.20)
Speech/Language Disorder	levetiracetam	High Dose	154 / 3036	456 / 8887	16.01 (13.17 - 19.40)	16.16 (14.28 - 18.25)	1.01 (0.82 - 1.23)
Speech/Language Disorder	levetiracetam	Low Dose	89 / 1879	456 / 8887	14.07 (10.95 - 17.98)	15.89 (14.08 - 17.91)	0.93 (0.73 - 1.18)
Speech/Language Disorder	levetiracetam	No Child Epilepsy	209 / 5,261	365 / 8,887	10.94 (9.15 - 13.05)	13.05 (11.50 - 14.80)	0.89 (0.74 - 1.07)
Speech/Language Disorder	levetiracetam	Accounting for Censoring	261 / 5261	456 / 8887	**	**	0.95 (0.80 - 1.14)
Speech/Language Disorder	lamotrigine	Main (Crude)	246 / 4299	456 / 8887	15.33 (13.35 - 17.57)	14.46 (13.04 - 16.02)	1.12 (0.96 - 1.31)
Speech/Language Disorder	lamotrigine	Main (Adjusted)	246 / 4299	456 / 8887	16.20 (13.97 - 18.74)	14.78 (13.08 - 16.67)	1.11 (0.93 - 1.32)
Speech/Language Disorder	lamotrigine	Monotherapy	175 / 3200	456 / 8887	14.93 (12.51 - 17.77)	14.41 (12.65 - 16.38)	1.05 (0.87 - 1.28)
Speech/Language Disorder	lamotrigine	Polytherapy (no Valproate)	64 / 1043	456 / 8887	18.74 (14.34 - 24.29)	15.92 (13.85 - 18.25)	1.17 (0.88 - 1.54)
Speech/Language Disorder	lamotrigine	Strict Monotherapy	152 / 2864	456 / 8887	14.41 (11.86 - 17.47)	13.98 (12.20 - 15.99)	1.04 (0.84 - 1.28)
Speech/Language Disorder	lamotrigine	>=2 Rx	205 / 3606	456 / 8887	15.71 (13.37 - 18.41)	14.65 (12.89 - 16.62)	1.10 (0.92 - 1.33)
Speech/Language Disorder	lamotrigine	High Dose	144 / 2542	456 / 8887	16.05 (13.25 - 19.37)	14.43 (12.57 - 16.55)	1.12 (0.90 - 1.39)
Speech/Language Disorder	lamotrigine	Low Dose	88 / 1489	456 / 8887	16.04 (12.67 - 20.18)	15.23 (13.43 - 17.24)	1.08 (0.85 - 1.37)
Speech/Language Disorder	lamotrigine	No Child Epilepsy	193 / 4,299	365 / 8,887	12.17 (10.21 - 14.47)	12.14 (10.61 - 13.86)	1.00 (0.82 - 1.22)
Speech/Language Disorder	lamotrigine	Accounting for Censoring	246 / 4299	456 / 8887	**	**	1.08 (0.90 - 1.31)
Speech/Language Disorder	carbamazepine	Main (Crude)	124 / 1924	456 / 8887	13.07 (10.89 - 15.64)	14.46 (13.04 - 16.02)	0.94 (0.77 - 1.15)
Speech/Language Disorder	carbamazepine	Main (Adjusted)	124 / 1924	456 / 8887	13.22 (10.94 - 15.94)	13.24 (11.50 - 15.22)	1.07 (0.86 - 1.34)
Speech/Language Disorder	carbamazepine	Monotherapy	89 / 1456	456 / 8887	12.43 (9.94 - 15.49)	13.01 (11.23 - 15.05)	1.04 (0.81 - 1.34)
Speech/Language Disorder	carbamazepine	Polytherapy (no Valproate)	31 / 435	456 / 8887	14.39 (9.95 - 20.56)	13.10 (11.06 - 15.48)	1.11 (0.75 - 1.64)
Speech/Language Disorder	carbamazepine	Strict Monotherapy	83 / 1333	456 / 8887	13.39 (10.67 - 16.74)	12.75 (10.95 - 14.81)	1.11 (0.86 - 1.44)
Speech/Language Disorder	carbamazepine	>=2 Rx	97 / 1545	456 / 8887	12.78 (10.28 - 15.84)	12.98 (11.19 - 15.02)	1.05 (0.82 - 1.34)
Speech/Language Disorder	carbamazepine	Active Comparator	141 / 2211	392 / 7381	12.88 (10.61 - 15.59)	13.57 (11.47 - 16.03)	1.03 (0.81 - 1.32)
Speech/Language Disorder	carbamazepine	High Dose	83 / 1200	456 / 8887	13.58 (10.78 - 17.02)	13.05 (11.21 - 15.18)	1.10 (0.85 - 1.43)
Speech/Language Disorder	carbamazepine	Low Dose	32 / 508	456 / 8887	12.32 (8.65 - 17.41)	13.55 (11.61 - 15.78)	1.01 (0.69 - 1.46)
Speech/Language Disorder	carbamazepine	No Child Epilepsy	82 / 1,924	365 / 8,887	8.80 (6.91 - 11.19)	10.54 (9.00 - 12.33)	0.89 (0.68 - 1.16)
Speech/Language Disorder	carbamazepine	Accounting for Censoring	124 / 1924	456 / 8887	**	**	1.03 (0.82 - 1.30)

Outcome	Exposure	Analysis	N Cases/ Total		Cum. Incidence in % (95% CI) at Age 8 Years		HR (95% CI)
			Among Exposed	Among Unexposed	Among Exposed	Among Unexposed	
Speech/Language Disorder	phenytoin	Main (Crude)	98 / 1595	456 / 8887	12.34 (10.10 - 15.02)	14.46 (13.04 - 16.02)	0.80 (0.64 - 1.00)
Speech/Language Disorder	phenytoin	Main (Adjusted)	98 / 1595	456 / 8887	11.93 (9.68 - 14.67)	13.68 (11.92 - 15.69)	0.85 (0.66 - 1.08)
Speech/Language Disorder	phenytoin	Monotherapy	65 / 1177	456 / 8887	11.22 (8.73 - 14.35)	13.37 (11.62 - 15.35)	0.77 (0.58 - 1.02)
Speech/Language Disorder	phenytoin	Polytherapy (no Valproate)	30 / 382	456 / 8887	14.94 (10.20 - 21.60)	14.02 (11.77 - 16.65)	1.16 (0.78 - 1.74)
Speech/Language Disorder	phenytoin	Strict Monotherapy	62 / 1060	456 / 8887	11.82 (9.16 - 15.20)	13.19 (11.40 - 15.23)	0.83 (0.62 - 1.11)
Speech/Language Disorder	phenytoin	>=2 Rx	75 / 1172	456 / 8887	12.34 (9.74 - 15.57)	13.51 (11.68 - 15.61)	0.87 (0.66 - 1.13)
Speech/Language Disorder	phenytoin	Active Comparator	103 / 1702	392 / 7381	10.94 (8.49 - 14.04)	14.24 (11.51 - 17.56)	0.79 (0.57 - 1.08)
Speech/Language Disorder	phenytoin	High Dose	45 / 789	456 / 8887	10.53 (7.74 - 14.24)	13.73 (11.79 - 15.95)	0.74 (0.53 - 1.03)
Speech/Language Disorder	phenytoin	Low Dose	42 / 642	456 / 8887	13.10 (9.61 - 17.74)	13.25 (11.37 - 15.42)	0.96 (0.68 - 1.34)
Speech/Language Disorder	phenytoin	No Child Epilepsy	69 / 1,595	365 / 8,887	8.51 (6.62 - 10.91)	10.51 (8.95 - 12.31)	0.79 (0.60 - 1.05)
Speech/Language Disorder	phenytoin	Accounting for Censoring	98 / 1595	456 / 8887	**	**	0.83 (0.65 - 1.07)
Speech/Language Disorder	topiramate	Main (Crude)	75 / 1036	456 / 8887	17.19 (13.56 - 21.67)	14.46 (13.04 - 16.02)	1.23 (0.96 - 1.57)
Speech/Language Disorder	topiramate	Main (Adjusted)	75 / 1036	456 / 8887	17.13 (13.43 - 21.71)	15.88 (13.99 - 18.00)	1.10 (0.85 - 1.43)
Speech/Language Disorder	topiramate	Monotherapy	43 / 626	456 / 8887	15.86 (11.57 - 21.54)	15.95 (13.98 - 18.17)	1.04 (0.75 - 1.44)
Speech/Language Disorder	topiramate	Polytherapy (no Valproate)	31 / 383	456 / 8887	19.99 (13.57 - 28.89)	15.99 (13.73 - 18.58)	1.23 (0.84 - 1.81)
Speech/Language Disorder	topiramate	Strict Monotherapy	38 / 565	456 / 8887	15.63 (11.28 - 21.44)	15.93 (13.95 - 18.17)	1.04 (0.73 - 1.47)
Speech/Language Disorder	topiramate	>=2 Rx	50 / 781	456 / 8887	14.16 (10.61 - 18.75)	15.25 (13.30 - 17.46)	0.98 (0.72 - 1.34)
Speech/Language Disorder	topiramate	Active Comparator	155 / 2494	392 / 7381	15.91 (13.31 - 18.95)	13.35 (11.54 - 15.41)	1.18 (0.95 - 1.47)
Speech/Language Disorder	topiramate	High Dose	48 / 617	456 / 8887	16.71 (12.51 - 22.14)	15.02 (13.03 - 17.29)	1.14 (0.83 - 1.57)
Speech/Language Disorder	topiramate	Low Dose	27 / 363	456 / 8887	20.90 (13.42 - 31.71)	17.47 (15.28 - 19.95)	1.18 (0.80 - 1.76)
Speech/Language Disorder	topiramate	No Child Epilepsy	64 / 1,036	365 / 8,887	14.25 (10.97 - 18.42)	12.74 (11.06 - 14.65)	1.15 (0.87 - 1.54)
Speech/Language Disorder	topiramate	Accounting for Censoring	75 / 1036	456 / 8887	**	**	1.03 (0.78 - 1.36)
Speech/Language Disorder	oxcarbazepine	Main (Crude)	44 / 845	456 / 8887	13.91 (10.02 - 19.15)	14.46 (13.04 - 16.02)	0.92 (0.68 - 1.26)
Speech/Language Disorder	oxcarbazepine	Main (Adjusted)	44 / 845	456 / 8887	14.42 (10.36 - 19.88)	15.24 (13.24 - 17.51)	0.91 (0.66 - 1.26)
Speech/Language Disorder	oxcarbazepine	Monotherapy	27 / 579	456 / 8887	12.25 (8.00 - 18.52)	14.90 (12.83 - 17.28)	0.83 (0.56 - 1.23)
Speech/Language Disorder	oxcarbazepine	Polytherapy (no Valproate)	14 / 254	456 / 8887	18.54 (10.52 - 31.49)	15.64 (13.09 - 18.63)	0.99 (0.57 - 1.72)
Speech/Language Disorder	oxcarbazepine	Strict Monotherapy	25 / 522	456 / 8887	13.19 (8.25 - 20.72)	14.69 (12.56 - 17.13)	0.92 (0.61 - 1.39)
Speech/Language Disorder	oxcarbazepine	>=2 Rx	34 / 685	456 / 8887	13.81 (9.51 - 19.82)	15.26 (13.16 - 17.65)	0.86 (0.60 - 1.24)
Speech/Language Disorder	oxcarbazepine	Active Comparator	70 / 1355	392 / 7381	13.27 (10.18 - 17.20)	14.50 (12.69 - 16.55)	0.87 (0.67 - 1.13)
Speech/Language Disorder	oxcarbazepine	High Dose	26 / 454	456 / 8887	16.02 (10.54 - 23.93)	16.13 (13.72 - 18.92)	0.98 (0.65 - 1.47)
Speech/Language Disorder	oxcarbazepine	Low Dose	17 / 332	456 / 8887	12.98 (7.54 - 21.84)	14.57 (12.44 - 17.03)	0.84 (0.51 - 1.37)
Speech/Language Disorder	oxcarbazepine	No Child Epilepsy	36 / 845	365 / 8,887	12.08 (8.34 - 17.33)	12.45 (10.65 - 14.52)	0.93 (0.65 - 1.33)
Speech/Language Disorder	oxcarbazepine	Accounting for Censoring	44 / 845	456 / 8887	**	**	0.95 (0.67 - 1.33)
Speech/Language Disorder	valproate	Main (Crude)	79 / 800	456 / 8887	21.64 (17.37 - 26.79)	14.46 (13.04 - 16.02)	1.45 (1.14 - 1.84)
Speech/Language Disorder	valproate	Main (Adjusted)	79 / 800	456 / 8887	21.48 (17.15 - 26.71)	14.07 (12.15 - 16.27)	1.52 (1.17 - 1.98)
Speech/Language Disorder	valproate	Monotherapy	57 / 561	456 / 8887	21.43 (16.40 - 27.74)	13.65 (11.65 - 15.97)	1.60 (1.18 - 2.16)
Speech/Language Disorder	valproate	Strict Monotherapy	52 / 515	456 / 8887	20.70 (15.66 - 27.09)	13.37 (11.36 - 15.70)	1.60 (1.17 - 2.19)
Speech/Language Disorder	valproate	>=2 Rx	67 / 568	456 / 8887	26.03 (20.51 - 32.70)	14.24 (12.18 - 16.62)	1.82 (1.37 - 2.41)
Speech/Language Disorder	valproate	Active Comparator	122 / 1392	392 / 7381	19.33 (15.83 - 23.48)	13.85 (11.77 - 16.25)	1.41 (1.10 - 1.79)
Speech/Language Disorder	valproate	High Dose	57 / 509	456 / 8887	22.36 (17.27 - 28.69)	14.40 (12.33 - 16.79)	1.63 (1.21 - 2.20)
Speech/Language Disorder	valproate	Low Dose	17 / 209	456 / 8887	18.26 (11.22 - 28.92)	13.39 (11.31 - 15.82)	1.33 (0.81 - 2.18)
Speech/Language Disorder	valproate	No Child Epilepsy	56 / 800	365 / 8,887	15.47 (11.80 - 20.15)	10.78 (9.11 - 12.74)	1.44 (1.06 - 1.96)
Speech/Language Disorder	valproate	Accounting for Censoring	79 / 800	456 / 8887	**	**	1.48 (1.12 - 1.94)
Speech/Language Disorder	zonisamide	Main (Crude)	30 / 446	456 / 8887	15.57 (10.59 - 22.59)	14.46 (13.04 - 16.02)	1.36 (0.94 - 1.96)
Speech/Language Disorder	zonisamide	Main (Adjusted)	30 / 446	456 / 8887	15.72 (10.66 - 22.84)	14.52 (12.24 - 17.18)	1.33 (0.90 - 1.96)
Speech/Language Disorder	zonisamide	Monotherapy	14 / 225	443 / 8593	12.74 (7.54 - 21.09)	12.97 (10.74 - 15.61)	1.24 (0.72 - 2.15)
Speech/Language Disorder	zonisamide	Polytherapy (no Valproate)	16 / 211	419 / 8007	20.01 (11.75 - 32.88)	16.42 (13.30 - 20.19)	1.50 (0.89 - 2.52)
Speech/Language Disorder	zonisamide	Strict Monotherapy	12 / 207	443 / 8593	12.43 (7.02 - 21.49)	12.66 (10.35 - 15.44)	1.21 (0.67 - 2.19)

Outcome	Exposure	Analysis	N Cases/ Total		Cum. Incidence in % (95% CI) at Age 8 Years		HR (95% CI)
			Among Exposed	Among Unexposed	Among Exposed	Among Unexposed	
Speech/Language Disorder	zonisamide	>=2 Rx	25 / 355	456 / 8887	16.54 (10.80 - 24.89)	14.30 (11.85 - 17.21)	1.42 (0.93 - 2.16)
Speech/Language Disorder	zonisamide	Active Comparator	32 / 508	392 / 7381	14.49 (9.82 - 21.12)	14.42 (11.98 - 17.30)	1.20 (0.82 - 1.76)
Speech/Language Disorder	zonisamide	High Dose	19 / 276	456 / 8887	15.93 (9.67 - 25.63)	14.84 (12.31 - 17.84)	1.28 (0.79 - 2.08)
Speech/Language Disorder	zonisamide	Low Dose	****	****	****	****	****
Speech/Language Disorder	zonisamide	No Child Epilepsy	24 / 446	365 / 8,887	12.71 (8.16 - 19.50)	11.69 (9.69 - 14.07)	1.26 (0.82 - 1.94)
Speech/Language Disorder	zonisamide	Accounting for Censoring	30 / 446	456 / 8887	**	**	1.24 (0.82 - 1.86)
Speech/Language Disorder	phenobarbital	Main (Crude)	25 / 416	456 / 8887	11.38 (7.68 - 16.69)	14.46 (13.04 - 16.02)	0.78 (0.52 - 1.16)
Speech/Language Disorder	phenobarbital	Main (Adjusted)	25 / 416	456 / 8887	11.23 (6.54 - 18.93)	12.51 (8.59 - 18.04)	0.89 (0.48 - 1.66)
Speech/Language Disorder	phenobarbital	Monotherapy	15 / 255	430 / 8148	11.08 (5.51 - 21.59)	11.15 (6.85 - 17.88)	0.87 (0.42 - 1.80)
Speech/Language Disorder	phenobarbital	Polytherapy (no Valproate)	****	****	****	****	****
Speech/Language Disorder	phenobarbital	Strict Monotherapy	****	****	****	****	****
Speech/Language Disorder	phenobarbital	>=2 Rx	19 / 319	456 / 8887	7.10 (2.84 - 17.13)	11.33 (6.51 - 19.32)	0.51 (0.24 - 1.11)
Speech/Language Disorder	phenobarbital	Active Comparator	53 / 934	392 / 7381	13.06 (9.36 - 18.08)	13.97 (10.72 - 18.09)	1.01 (0.65 - 1.57)
Speech/Language Disorder	phenobarbital	High Dose	15 / 221	456 / 8887	9.19 (3.92 - 20.73)	11.97 (6.35 - 21.96)	0.75 (0.32 - 1.76)
Speech/Language Disorder	phenobarbital	Low Dose	****	****	****	****	****
Speech/Language Disorder	phenobarbital	No Child Epilepsy	14 / 416	365 / 8,887	7.66 (3.93 - 14.63)	9.97 (6.50 - 15.15)	0.76 (0.36 - 1.59)
Speech/Language Disorder	phenobarbital	Accounting for Censoring	25 / 416	456 / 8887	**	**	0.85 (0.46 - 1.56)
Speech/Language Disorder	lacosamide	Main (Crude)	*** / 219	456 / 8887	10.67 (5.04 - 21.83)	14.46 (13.04 - 16.02)	1.14 (0.56 - 2.29)
Speech/Language Disorder	lacosamide	Main (Adjusted)	*** / 219	283 / 6175	11.06 (5.20 - 22.70)	14.86 (11.11 - 19.73)	0.77 (0.37 - 1.63)
Speech/Language Disorder	lacosamide	Monotherapy	****	****	****	****	****
Speech/Language Disorder	lacosamide	Polytherapy (no Valproate)	****	****	****	****	****
Speech/Language Disorder	lacosamide	Strict Monotherapy	****	****	****	****	****
Speech/Language Disorder	lacosamide	>=2 Rx	****	****	****	****	****
Speech/Language Disorder	lacosamide	Active Comparator	*** / 226	392 / 7381	9.86 (4.52 - 20.78)	14.61 (11.17 - 18.99)	0.79 (0.37 - 1.67)
Speech/Language Disorder	lacosamide	High Dose	****	****	****	****	****
Speech/Language Disorder	lacosamide	Low Dose	****	****	****	****	****
Speech/Language Disorder	lacosamide	No Child Epilepsy	*** / 219	238 / 6,175	9.11 (3.82 - 20.90)	12.99 (9.55 - 17.56)	0.66 (0.28 - 1.54)
Speech/Language Disorder	lacosamide	Accounting for Censoring	*** / 219	283 / 6175	**	**	0.90 (0.43 - 1.91)
ADHD	levetiracetam	Main (Crude)	106 / 5261	366 / 8887	13.55 (10.94 - 16.73)	20.01 (17.93 - 22.29)	0.68 (0.54 - 0.84)
ADHD	levetiracetam	Main (Adjusted)	106 / 5261	366 / 8887	14.48 (11.71 - 17.83)	17.94 (15.58 - 20.61)	0.81 (0.64 - 1.02)
ADHD	levetiracetam	Monotherapy	74 / 3852	366 / 8887	14.43 (11.19 - 18.51)	17.35 (14.89 - 20.17)	0.89 (0.68 - 1.16)
ADHD	levetiracetam	Polytherapy (no Valproate)	27 / 1343	366 / 8887	11.38 (7.40 - 17.30)	18.37 (15.61 - 21.56)	0.60 (0.40 - 0.91)
ADHD	levetiracetam	Strict Monotherapy	57 / 3360	366 / 8887	14.15 (10.55 - 18.85)	16.97 (14.45 - 19.86)	0.87 (0.64 - 1.17)
ADHD	levetiracetam	>=2 Rx	79 / 4067	366 / 8887	14.02 (10.94 - 17.87)	17.43 (15.02 - 20.18)	0.78 (0.60 - 1.02)
ADHD	levetiracetam	Active Comparator	113 / 5487	250 / 7381	13.65 (10.85 - 17.10)	18.07 (14.94 - 21.77)	0.82 (0.62 - 1.07)
ADHD	levetiracetam	High Dose	61 / 3036	366 / 8887	14.23 (10.83 - 18.57)	17.58 (15.05 - 20.48)	0.83 (0.62 - 1.12)
ADHD	levetiracetam	Low Dose	35 / 1879	366 / 8887	11.56 (7.83 - 16.90)	18.14 (15.66 - 20.96)	0.67 (0.47 - 0.95)
ADHD	levetiracetam	No Child Epilepsy	78 / 5,261	275 / 8,887	10.75 (8.39 - 13.73)	14.05 (11.93 - 16.50)	0.81 (0.61 - 1.06)
ADHD	levetiracetam	Accounting for Censoring	106 / 5261	366 / 8887	**	**	0.84 (0.65 - 1.07)
ADHD	lamotrigine	Main (Crude)	136 / 4299	366 / 8887	17.72 (14.93 - 20.97)	20.01 (17.93 - 22.29)	0.77 (0.63 - 0.93)
ADHD	lamotrigine	Main (Adjusted)	136 / 4299	366 / 8887	20.33 (17.16 - 24.01)	17.52 (15.19 - 20.17)	1.04 (0.84 - 1.29)
ADHD	lamotrigine	Monotherapy	97 / 3200	366 / 8887	19.50 (16.01 - 23.64)	17.08 (14.65 - 19.87)	1.06 (0.83 - 1.36)
ADHD	lamotrigine	Polytherapy (no Valproate)	34 / 1043	366 / 8887	22.11 (15.84 - 30.38)	17.89 (15.15 - 21.07)	0.97 (0.67 - 1.40)
ADHD	lamotrigine	Strict Monotherapy	84 / 2864	366 / 8887	19.82 (16.03 - 24.37)	16.58 (14.08 - 19.46)	1.09 (0.84 - 1.42)
ADHD	lamotrigine	>=2 Rx	106 / 3606	366 / 8887	18.65 (15.34 - 22.57)	17.14 (14.73 - 19.90)	0.96 (0.75 - 1.22)
ADHD	lamotrigine	High Dose	66 / 2542	366 / 8887	15.03 (11.57 - 19.40)	16.42 (13.86 - 19.39)	0.82 (0.61 - 1.11)
ADHD	lamotrigine	Low Dose	66 / 1489	366 / 8887	25.64 (20.29 - 32.08)	18.78 (16.32 - 21.57)	1.25 (0.95 - 1.65)
ADHD	lamotrigine	No Child Epilepsy	100 / 4,299	275 / 8,887	15.31 (12.51 - 18.67)	13.39 (11.32 - 15.81)	1.03 (0.80 - 1.32)

Outcome	Exposure	Analysis	N Cases/ Total		Cum. Incidence in % (95% CI) at Age 8 Years		HR (95% CI)
			Among Exposed	Among Unexposed	Among Exposed	Among Unexposed	
ADHD	lamotrigine	Accounting for Censoring	136 / 4299	366 / 8887	**	**	0.99 (0.78 - 1.24)
ADHD	carbamazepine	Main (Crude)	144 / 1924	366 / 8887	22.32 (18.82 - 26.35)	20.01 (17.93 - 22.29)	1.11 (0.91 - 1.34)
ADHD	carbamazepine	Main (Adjusted)	144 / 1924	366 / 8887	23.06 (19.35 - 27.36)	18.56 (16.12 - 21.33)	1.23 (0.99 - 1.52)
ADHD	carbamazepine	Monotherapy	108 / 1456	366 / 8887	24.00 (19.77 - 28.95)	18.34 (15.83 - 21.18)	1.27 (1.00 - 1.60)
ADHD	carbamazepine	Polytherapy (no Valproate)	30 / 435	366 / 8887	18.42 (12.13 - 27.42)	18.66 (15.69 - 22.10)	1.07 (0.73 - 1.58)
ADHD	carbamazepine	Strict Monotherapy	95 / 1333	366 / 8887	23.70 (19.29 - 28.91)	18.13 (15.60 - 21.02)	1.26 (0.98 - 1.62)
ADHD	carbamazepine	>=2 Rx	116 / 1545	366 / 8887	23.53 (19.39 - 28.39)	18.36 (15.83 - 21.25)	1.24 (0.98 - 1.56)
ADHD	carbamazepine	Active Comparator	162 / 2211	250 / 7381	21.16 (17.41 - 25.59)	19.00 (15.83 - 22.72)	1.26 (0.97 - 1.63)
ADHD	carbamazepine	High Dose	107 / 1200	366 / 8887	23.73 (19.39 - 28.84)	18.46 (15.85 - 21.44)	1.35 (1.06 - 1.72)
ADHD	carbamazepine	Low Dose	29 / 508	366 / 8887	19.71 (13.57 - 28.13)	17.78 (15.17 - 20.78)	0.93 (0.63 - 1.38)
ADHD	carbamazepine	No Child Epilepsy	106 / 1,924	275 / 8,887	16.73 (13.50 - 20.63)	13.69 (11.56 - 16.17)	1.22 (0.95 - 1.56)
ADHD	carbamazepine	Accounting for Censoring	144 / 1924	366 / 8887	**	**	1.22 (0.98 - 1.52)
ADHD	phenytoin	Main (Crude)	144 / 1595	366 / 8887	21.22 (17.82 - 25.16)	20.01 (17.93 - 22.29)	1.06 (0.87 - 1.28)
ADHD	phenytoin	Main (Adjusted)	144 / 1595	366 / 8887	20.90 (17.41 - 24.98)	21.14 (18.58 - 23.99)	1.01 (0.82 - 1.24)
ADHD	phenytoin	Monotherapy	103 / 1177	366 / 8887	19.58 (15.81 - 24.13)	21.01 (18.47 - 23.86)	0.96 (0.76 - 1.21)
ADHD	phenytoin	Polytherapy (no Valproate)	36 / 382	366 / 8887	24.62 (17.11 - 34.67)	21.21 (18.02 - 24.88)	1.18 (0.82 - 1.70)
ADHD	phenytoin	Strict Monotherapy	98 / 1060	366 / 8887	19.99 (16.04 - 24.76)	20.84 (18.25 - 23.74)	1.00 (0.79 - 1.28)
ADHD	phenytoin	>=2 Rx	108 / 1172	366 / 8887	21.42 (17.39 - 26.23)	20.55 (17.92 - 23.52)	1.03 (0.82 - 1.31)
ADHD	phenytoin	Active Comparator	155 / 1702	250 / 7381	20.44 (16.44 - 25.25)	21.01 (17.03 - 25.76)	1.01 (0.75 - 1.36)
ADHD	phenytoin	High Dose	77 / 789	366 / 8887	23.00 (18.14 - 28.91)	20.10 (17.38 - 23.18)	1.09 (0.84 - 1.42)
ADHD	phenytoin	Low Dose	60 / 642	366 / 8887	19.81 (14.89 - 26.09)	21.66 (18.92 - 24.74)	1.00 (0.75 - 1.34)
ADHD	phenytoin	No Child Epilepsy	111 / 1,595	275 / 8,887	16.50 (13.38 - 20.26)	15.62 (13.38 - 18.20)	1.08 (0.85 - 1.37)
ADHD	phenytoin	Accounting for Censoring	144 / 1595	366 / 8887	**	**	1.00 (0.80 - 1.24)
ADHD	topiramate	Main (Crude)	63 / 1036	366 / 8887	21.65 (16.66 - 27.86)	20.01 (17.93 - 22.29)	1.15 (0.88 - 1.51)
ADHD	topiramate	Main (Adjusted)	63 / 1036	366 / 8887	21.90 (16.82 - 28.24)	20.36 (17.69 - 23.36)	1.13 (0.85 - 1.50)
ADHD	topiramate	Monotherapy	38 / 626	366 / 8887	19.66 (13.93 - 27.34)	20.27 (17.51 - 23.39)	1.01 (0.71 - 1.43)
ADHD	topiramate	Polytherapy (no Valproate)	21 / 383	366 / 8887	26.50 (16.89 - 40.10)	19.83 (16.61 - 23.58)	1.29 (0.82 - 2.03)
ADHD	topiramate	Strict Monotherapy	31 / 565	366 / 8887	18.03 (12.26 - 26.08)	19.89 (17.14 - 23.02)	0.98 (0.67 - 1.44)
ADHD	topiramate	>=2 Rx	42 / 781	366 / 8887	17.97 (12.80 - 24.91)	19.99 (17.22 - 23.13)	0.96 (0.69 - 1.35)
ADHD	topiramate	Active Comparator	134 / 2494	250 / 7381	20.93 (17.06 - 25.54)	25.13 (21.81 - 28.86)	0.97 (0.76 - 1.24)
ADHD	topiramate	High Dose	42 / 617	366 / 8887	22.16 (16.06 - 30.13)	19.83 (16.95 - 23.12)	1.13 (0.81 - 1.59)
ADHD	topiramate	Low Dose	21 / 363	366 / 8887	22.90 (14.31 - 35.45)	20.74 (17.69 - 24.25)	1.26 (0.81 - 1.98)
ADHD	topiramate	No Child Epilepsy	44 / 1,036	275 / 8,887	14.90 (10.67 - 20.59)	15.77 (13.36 - 18.55)	1.02 (0.73 - 1.43)
ADHD	topiramate	Accounting for Censoring	63 / 1036	366 / 8887	**	**	1.13 (0.84 - 1.52)
ADHD	oxcarbazepine	Main (Crude)	48 / 845	366 / 8887	25.01 (18.84 - 32.76)	20.01 (17.93 - 22.29)	1.24 (0.92 - 1.68)
ADHD	oxcarbazepine	Main (Adjusted)	48 / 845	366 / 8887	24.99 (18.66 - 32.99)	19.60 (16.87 - 22.70)	1.28 (0.93 - 1.77)
ADHD	oxcarbazepine	Monotherapy	37 / 579	366 / 8887	26.35 (18.95 - 35.92)	19.64 (16.82 - 22.86)	1.43 (1.00 - 2.04)
ADHD	oxcarbazepine	Polytherapy (no Valproate)	*** / 254	366 / 8887	22.74 (12.11 - 40.31)	20.21 (16.65 - 24.41)	1.01 (0.54 - 1.89)
ADHD	oxcarbazepine	Strict Monotherapy	31 / 522	366 / 8887	28.27 (19.76 - 39.44)	19.15 (16.27 - 22.46)	1.53 (1.04 - 2.26)
ADHD	oxcarbazepine	>=2 Rx	34 / 685	366 / 8887	22.32 (15.64 - 31.27)	19.17 (16.33 - 22.44)	1.20 (0.83 - 1.74)
ADHD	oxcarbazepine	Active Comparator	76 / 1355	250 / 7381	23.55 (18.36 - 29.93)	22.52 (19.53 - 25.89)	1.14 (0.87 - 1.51)
ADHD	oxcarbazepine	High Dose	24 / 454	366 / 8887	25.44 (16.73 - 37.56)	17.34 (14.34 - 20.87)	1.47 (0.95 - 2.28)
ADHD	oxcarbazepine	Low Dose	22 / 332	366 / 8887	23.37 (15.16 - 35.02)	21.72 (18.63 - 25.24)	1.09 (0.70 - 1.71)
ADHD	oxcarbazepine	No Child Epilepsy	39 / 845	275 / 8,887	19.57 (13.99 - 27.01)	15.02 (12.61 - 17.86)	1.37 (0.95 - 1.96)
ADHD	oxcarbazepine	Accounting for Censoring	48 / 845	366 / 8887	**	**	1.36 (0.97 - 1.91)
ADHD	valproate	Main (Crude)	78 / 800	366 / 8887	27.23 (21.87 - 33.58)	20.01 (17.93 - 22.29)	1.36 (1.06 - 1.74)
ADHD	valproate	Main (Adjusted)	78 / 800	366 / 8887	26.93 (21.53 - 33.38)	21.76 (19.00 - 24.86)	1.26 (0.97 - 1.64)
ADHD	valproate	Monotherapy	50 / 561	366 / 8887	24.49 (18.46 - 32.07)	22.18 (19.29 - 25.44)	1.12 (0.82 - 1.54)

Outcome	Exposure	Analysis	N Cases/ Total		Cum. Incidence in % (95% CI) at Age 8 Years		HR (95% CI)
			**Among Exposed**	**Among Unexposed**	**Among Exposed**	**Among Unexposed**	
ADHD	valproate	Strict Monotherapy	48 / 515	366 / 8887	25.80 (19.44 - 33.75)	21.44 (18.54 - 24.71)	1.22 (0.88 - 1.68)
ADHD	valproate	>=2 Rx	61 / 568	366 / 8887	28.70 (22.27 - 36.51)	21.98 (19.01 - 25.35)	1.38 (1.03 - 1.85)
ADHD	valproate	Active Comparator	120 / 1392	250 / 7381	25.23 (20.61 - 30.68)	25.35 (21.78 - 29.39)	1.08 (0.83 - 1.39)
ADHD	valproate	High Dose	58 / 509	366 / 8887	28.45 (22.07 - 36.19)	21.49 (18.57 - 24.80)	1.37 (1.02 - 1.85)
ADHD	valproate	Low Dose	15 / 209	366 / 8887	21.55 (12.42 - 35.86)	20.96 (17.92 - 24.42)	0.95 (0.56 - 1.61)
ADHD	valproate	No Child Epilepsy	47 / 800	275 / 8,887	15.18 (11.02 - 20.72)	16.11 (13.68 - 18.92)	1.02 (0.73 - 1.41)
ADHD	valproate	Accounting for Censoring	78 / 800	366 / 8887	**	**	1.23 (0.94 - 1.62)
ADHD	zonisamide	Main (Crude)	23 / 446	366 / 8887	23.20 (14.72 - 35.45)	20.01 (17.93 - 22.29)	1.19 (0.78 - 1.81)
ADHD	zonisamide	Main (Adjusted)	23 / 446	366 / 8887	23.05 (14.57 - 35.35)	18.99 (15.61 - 23.00)	1.33 (0.85 - 2.06)
ADHD	zonisamide	Monotherapy	*** / 225	346 / 8593	19.93 (9.48 - 39.09)	19.37 (15.77 - 23.66)	1.24 (0.66 - 2.34)
ADHD	zonisamide	Polytherapy (no Valproate)	12 / 211	310 / 8007	25.22 (13.50 - 44.16)	19.20 (14.87 - 24.59)	1.29 (0.71 - 2.32)
ADHD	zonisamide	Strict Monotherapy	*** / 207	346 / 8593	9.81 (3.24 - 27.65)	19.07 (15.26 - 23.69)	0.84 (0.38 - 1.86)
ADHD	zonisamide	>=2 Rx	20 / 355	366 / 8887	25.57 (15.78 - 39.81)	19.87 (16.12 - 24.36)	1.40 (0.87 - 2.24)
ADHD	zonisamide	Active Comparator	24 / 508	250 / 7381	21.78 (13.89 - 33.21)	20.15 (16.49 - 24.51)	1.16 (0.74 - 1.83)
ADHD	zonisamide	High Dose	12 / 276	366 / 8887	18.08 (9.07 - 34.18)	19.27 (15.59 - 23.68)	1.04 (0.59 - 1.82)
ADHD	zonisamide	Low Dose	****	****	****	****	****
ADHD	zonisamide	No Child Epilepsy	18 / 446	275 / 8,887	20.63 (12.44 - 33.08)	13.92 (10.99 - 17.54)	1.50 (0.91 - 2.47)
ADHD	zonisamide	Accounting for Censoring	23 / 446	366 / 8887	**	**	1.30 (0.80 - 2.11)
ADHD	phenobarbital	Main (Crude)	39 / 416	366 / 8887	19.53 (13.71 - 27.41)	20.01 (17.93 - 22.29)	1.00 (0.71 - 1.39)
ADHD	phenobarbital	Main (Adjusted)	39 / 416	366 / 8887	22.38 (14.00 - 34.65)	15.30 (9.85 - 23.35)	1.11 (0.66 - 1.88)
ADHD	phenobarbital	Monotherapy	27 / 255	362 / 8148	26.89 (16.42 - 42.13)	14.24 (7.97 - 24.71)	1.32 (0.69 - 2.52)
ADHD	phenobarbital	Polytherapy (no Valproate)	****	****	****	****	****
ADHD	phenobarbital	Strict Monotherapy	****	****	****	****	****
ADHD	phenobarbital	>=2 Rx	30 / 319	366 / 8887	21.82 (11.97 - 37.82)	12.78 (6.11 - 25.64)	1.59 (0.77 - 3.27)
ADHD	phenobarbital	Active Comparator	77 / 934	250 / 7381	21.25 (15.12 - 29.39)	28.40 (22.61 - 35.31)	0.69 (0.46 - 1.03)
ADHD	phenobarbital	High Dose	19 / 221	366 / 8887	15.53 (7.28 - 31.41)	13.34 (6.37 - 26.76)	1.00 (0.44 - 2.29)
ADHD	phenobarbital	Low Dose	****	****	****	****	****
ADHD	phenobarbital	No Child Epilepsy	30 / 416	275 / 8,887	15.66 (8.83 - 26.94)	9.21 (5.20 - 16.05)	1.39 (0.76 - 2.52)
ADHD	phenobarbital	Accounting for Censoring	39 / 416	366 / 8887	**	**	1.12 (0.67 - 1.86)
ADHD	lacosamide	Main (Crude)	*** / 219	366 / 8887	37.90 (8.40 - 92.48)	20.01 (17.93 - 22.29)	1.27 (0.41 - 3.97)
ADHD	lacosamide	Main (Adjusted)	*** / 219	116 / 6175	38.27 (8.71 - 92.23)	10.30 (4.80 - 21.35)	2.66 (0.77 - 9.21)
ADHD	lacosamide	Monotherapy	****	****	****	****	****
ADHD	lacosamide	Polytherapy (no Valproate)	****	****	****	****	****
ADHD	lacosamide	Strict Monotherapy	****	****	****	****	****
ADHD	lacosamide	>=2 Rx	****	****	****	****	****
ADHD	lacosamide	Active Comparator	*** / 226	250 / 7381	38.60 (9.71 - 90.26)	25.27 (19.64 - 32.17)	1.65 (0.59 - 4.63)
ADHD	lacosamide	High Dose	****	****	****	****	****
ADHD	lacosamide	Low Dose	****	****	****	****	****
ADHD	lacosamide	No Child Epilepsy	*** / 219	90 / 6,175	35.56 (6.87 - 93.37)	9.36 (4.13 - 20.45)	2.21 (0.48 - 10.28)
ADHD	lacosamide	Accounting for Censoring	*** / 219	116 / 6175	**	**	2.48 (0.76 - 8.09)
Behavioral Disorder	levetiracetam	Main (Crude)	89 / 5261	239 / 8887	8.54 (6.64 - 10.93)	11.21 (9.72 - 12.90)	0.86 (0.67 - 1.09)
Behavioral Disorder	levetiracetam	Main (Adjusted)	89 / 5261	239 / 8887	9.49 (7.39 - 12.14)	9.66 (8.06 - 11.55)	1.04 (0.80 - 1.36)
Behavioral Disorder	levetiracetam	Monotherapy	65 / 3852	239 / 8887	9.13 (6.72 - 12.35)	9.16 (7.54 - 11.11)	1.15 (0.85 - 1.54)
Behavioral Disorder	levetiracetam	Polytherapy (no Valproate)	21 / 1343	239 / 8887	8.05 (5.03 - 12.76)	10.10 (8.18 - 12.43)	0.78 (0.49 - 1.25)
Behavioral Disorder	levetiracetam	Strict Monotherapy	53 / 3360	239 / 8887	8.88 (6.23 - 12.58)	8.93 (7.28 - 10.92)	1.17 (0.85 - 1.61)
Behavioral Disorder	levetiracetam	>=2 Rx	72 / 4067	239 / 8887	9.55 (7.26 - 12.53)	9.52 (7.88 - 11.49)	1.09 (0.82 - 1.46)
Behavioral Disorder	levetiracetam	Active Comparator	93 / 5487	159 / 7381	7.90 (6.04 - 10.31)	9.57 (7.41 - 12.33)	1.00 (0.72 - 1.37)
Behavioral Disorder	levetiracetam	High Dose	60 / 3036	239 / 8887	10.92 (8.13 - 14.59)	9.40 (7.71 - 11.44)	1.19 (0.88 - 1.63)

Outcome	Exposure	Analysis	N Cases/ Total		Cum. Incidence in % (95% CI) at Age 8 Years		HR (95% CI)
			Among Exposed	Among Unexposed	Among Exposed	Among Unexposed	
Behavioral Disorder	levetiracetam	Low Dose	25 / 1879	239 / 8887	6.27 (3.73 - 10.43)	9.57 (7.91 - 11.55)	0.76 (0.50 - 1.16)
Behavioral Disorder	levetiracetam	No Child Epilepsy	66 / 5,261	185 / 8,887	6.91 (5.15 - 9.24)	7.74 (6.31 - 9.48)	0.97 (0.72 - 1.32)
Behavioral Disorder	levetiracetam	Accounting for Censoring	89 / 5261	239 / 8887	**	**	1.08 (0.81 - 1.44)
Behavioral Disorder	lamotrigine	Main (Crude)	84 / 4299	239 / 8887	8.26 (6.46 - 10.53)	11.21 (9.72 - 12.90)	0.73 (0.57 - 0.93)
Behavioral Disorder	lamotrigine	Main (Adjusted)	84 / 4299	239 / 8887	10.12 (7.97 - 12.81)	10.06 (8.38 - 12.04)	1.03 (0.79 - 1.35)
Behavioral Disorder	lamotrigine	Monotherapy	65 / 3200	239 / 8887	10.70 (8.22 - 13.87)	9.68 (7.95 - 11.76)	1.13 (0.84 - 1.52)
Behavioral Disorder	lamotrigine	Polytherapy (no Valproate)	18 / 1043	239 / 8887	8.48 (4.90 - 14.45)	10.77 (8.78 - 13.16)	0.76 (0.46 - 1.26)
Behavioral Disorder	lamotrigine	Strict Monotherapy	56 / 2864	239 / 8887	10.30 (7.76 - 13.61)	9.30 (7.54 - 11.45)	1.19 (0.87 - 1.62)
Behavioral Disorder	lamotrigine	>=2 Rx	64 / 3606	239 / 8887	9.01 (6.83 - 11.85)	9.80 (8.08 - 11.87)	0.94 (0.69 - 1.27)
Behavioral Disorder	lamotrigine	High Dose	38 / 2542	239 / 8887	7.73 (5.36 - 11.09)	9.51 (7.67 - 11.75)	0.82 (0.56 - 1.19)
Behavioral Disorder	lamotrigine	Low Dose	45 / 1489	239 / 8887	13.08 (9.48 - 17.90)	10.46 (8.72 - 12.51)	1.29 (0.92 - 1.80)
Behavioral Disorder	lamotrigine	No Child Epilepsy	65 / 4,299	185 / 8,887	7.74 (5.89 - 10.14)	8.04 (6.54 - 9.86)	1.02 (0.75 - 1.38)
Behavioral Disorder	lamotrigine	Accounting for Censoring	84 / 4299	239 / 8887	**	**	1.02 (0.76 - 1.36)
Behavioral Disorder	carbamazepine	Main (Crude)	93 / 1924	239 / 8887	12.08 (9.61 - 15.13)	11.21 (9.72 - 12.90)	1.12 (0.88 - 1.43)
Behavioral Disorder	carbamazepine	Main (Adjusted)	93 / 1924	239 / 8887	13.05 (10.40 - 16.33)	10.39 (8.63 - 12.48)	1.35 (1.04 - 1.77)
Behavioral Disorder	carbamazepine	Monotherapy	75 / 1456	239 / 8887	13.75 (10.72 - 17.56)	10.38 (8.57 - 12.56)	1.46 (1.09 - 1.94)
Behavioral Disorder	carbamazepine	Polytherapy (no Valproate)	14 / 435	239 / 8887	8.79 (4.75 - 15.98)	10.04 (7.99 - 12.59)	0.90 (0.52 - 1.55)
Behavioral Disorder	carbamazepine	Strict Monotherapy	66 / 1333	239 / 8887	13.65 (10.46 - 17.72)	10.27 (8.44 - 12.48)	1.46 (1.08 - 1.98)
Behavioral Disorder	carbamazepine	>=2 Rx	76 / 1545	239 / 8887	12.42 (9.59 - 16.01)	10.25 (8.43 - 12.43)	1.38 (1.03 - 1.84)
Behavioral Disorder	carbamazepine	Active Comparator	101 / 2211	159 / 7381	11.44 (8.78 - 14.83)	9.93 (7.74 - 12.68)	1.13 (0.82 - 1.56)
Behavioral Disorder	carbamazepine	High Dose	71 / 1200	239 / 8887	13.41 (10.28 - 17.41)	10.47 (8.58 - 12.76)	1.49 (1.10 - 2.00)
Behavioral Disorder	carbamazepine	Low Dose	19 / 508	239 / 8887	11.67 (7.33 - 18.31)	9.72 (7.87 - 11.97)	1.06 (0.65 - 1.72)
Behavioral Disorder	carbamazepine	No Child Epilepsy	64 / 1,924	185 / 8,887	8.66 (6.51 - 11.50)	8.05 (6.50 - 9.95)	1.17 (0.86 - 1.61)
Behavioral Disorder	carbamazepine	Accounting for Censoring	93 / 1924	239 / 8887	**	**	1.33 (1.01 - 1.75)
Behavioral Disorder	phenytoin	Main (Crude)	99 / 1595	239 / 8887	12.28 (9.78 - 15.36)	11.21 (9.72 - 12.90)	1.19 (0.94 - 1.50)
Behavioral Disorder	phenytoin	Main (Adjusted)	99 / 1595	239 / 8887	12.43 (9.83 - 15.67)	11.40 (9.58 - 13.54)	1.19 (0.92 - 1.54)
Behavioral Disorder	phenytoin	Monotherapy	73 / 1177	239 / 8887	12.20 (9.34 - 15.87)	11.31 (9.50 - 13.44)	1.18 (0.89 - 1.56)
Behavioral Disorder	phenytoin	Polytherapy (no Valproate)	23 / 382	239 / 8887	13.08 (7.89 - 21.25)	11.13 (8.90 - 13.87)	1.34 (0.85 - 2.13)
Behavioral Disorder	phenytoin	Strict Monotherapy	68 / 1060	239 / 8887	12.13 (9.18 - 15.94)	10.96 (9.14 - 13.12)	1.24 (0.92 - 1.66)
Behavioral Disorder	phenytoin	>=2 Rx	66 / 1172	239 / 8887	11.44 (8.64 - 15.07)	11.09 (9.22 - 13.32)	1.09 (0.81 - 1.47)
Behavioral Disorder	phenytoin	Active Comparator	102 / 1702	159 / 7381	11.16 (8.37 - 14.82)	11.54 (8.68 - 15.25)	0.96 (0.66 - 1.38)
Behavioral Disorder	phenytoin	High Dose	56 / 789	239 / 8887	13.54 (9.92 - 18.32)	10.88 (8.95 - 13.21)	1.38 (1.00 - 1.90)
Behavioral Disorder	phenytoin	Low Dose	36 / 642	239 / 8887	10.84 (7.42 - 15.70)	11.66 (9.71 - 13.98)	1.06 (0.73 - 1.54)
Behavioral Disorder	phenytoin	No Child Epilepsy	77 / 1,595	185 / 8,887	9.50 (7.21 - 12.45)	8.89 (7.28 - 10.84)	1.20 (0.89 - 1.61)
Behavioral Disorder	phenytoin	Accounting for Censoring	99 / 1595	239 / 8887	**	**	1.15 (0.88 - 1.51)
Behavioral Disorder	topiramate	Main (Crude)	35 / 1036	239 / 8887	9.44 (6.48 - 13.66)	11.21 (9.72 - 12.90)	1.01 (0.70 - 1.43)
Behavioral Disorder	topiramate	Main (Adjusted)	35 / 1036	239 / 8887	9.54 (6.54 - 13.82)	11.40 (9.48 - 13.68)	1.03 (0.70 - 1.49)
Behavioral Disorder	topiramate	Monotherapy	20 / 626	239 / 8887	7.76 (4.65 - 12.81)	11.14 (9.15 - 13.53)	0.94 (0.58 - 1.52)
Behavioral Disorder	topiramate	Polytherapy (no Valproate)	14 / 383	239 / 8887	13.52 (7.52 - 23.68)	11.64 (9.32 - 14.48)	1.23 (0.70 - 2.16)
Behavioral Disorder	topiramate	Strict Monotherapy	19 / 565	239 / 8887	8.96 (5.38 - 14.73)	10.87 (8.89 - 13.26)	1.08 (0.66 - 1.76)
Behavioral Disorder	topiramate	>=2 Rx	26 / 781	239 / 8887	8.48 (5.41 - 13.18)	11.47 (9.45 - 13.88)	0.98 (0.64 - 1.51)
Behavioral Disorder	topiramate	Active Comparator	79 / 2494	159 / 7381	9.74 (7.25 - 13.03)	11.36 (9.15 - 14.05)	0.94 (0.69 - 1.30)
Behavioral Disorder	topiramate	High Dose	25 / 617	239 / 8887	10.52 (6.81 - 16.07)	11.26 (9.15 - 13.82)	1.14 (0.74 - 1.77)
Behavioral Disorder	topiramate	Low Dose	*** / 363	239 / 8887	8.13 (3.73 - 17.26)	11.64 (9.52 - 14.20)	0.74 (0.36 - 1.52)
Behavioral Disorder	topiramate	No Child Epilepsy	23 / 1,036	185 / 8,887	6.13 (3.78 - 9.88)	9.23 (7.50 - 11.35)	0.83 (0.52 - 1.31)
Behavioral Disorder	topiramate	Accounting for Censoring	35 / 1036	239 / 8887	**	**	1.05 (0.71 - 1.56)
Behavioral Disorder	oxcarbazepine	Main (Crude)	32 / 845	239 / 8887	13.19 (8.89 - 19.32)	11.21 (9.72 - 12.90)	1.26 (0.87 - 1.82)
Behavioral Disorder	oxcarbazepine	Main (Adjusted)	32 / 845	239 / 8887	13.94 (9.39 - 20.44)	10.96 (9.01 - 13.31)	1.40 (0.94 - 2.07)

Outcome	Exposure	Analysis	N Cases/ Total		Cum. Incidence in % (95% CI) at Age 8 Years		HR (95% CI)
			Among Exposed	Among Unexposed	Among Exposed	Among Unexposed	
Behavioral Disorder	oxcarbazepine	Monotherapy	20 / 579	239 / 8887	11.14 (6.67 - 18.29)	11.02 (8.97 - 13.52)	1.24 (0.77 - 2.01)
Behavioral Disorder	oxcarbazepine	Polytherapy (no Valproate)	12 / 254	239 / 8887	20.51 (10.88 - 36.72)	10.89 (8.45 - 13.97)	1.86 (1.00 - 3.44)
Behavioral Disorder	oxcarbazepine	Strict Monotherapy	14 / 522	239 / 8887	8.80 (4.93 - 15.44)	10.65 (8.56 - 13.20)	1.07 (0.61 - 1.88)
Behavioral Disorder	oxcarbazepine	>=2 Rx	23 / 685	239 / 8887	12.14 (7.58 - 19.13)	10.83 (8.79 - 13.31)	1.34 (0.86 - 2.10)
Behavioral Disorder	oxcarbazepine	Active Comparator	54 / 1355	159 / 7381	14.70 (10.81 - 19.82)	11.24 (9.22 - 13.67)	1.30 (0.94 - 1.82)
Behavioral Disorder	oxcarbazepine	High Dose	15 / 454	239 / 8887	13.00 (7.20 - 22.88)	10.31 (8.15 - 12.99)	1.36 (0.78 - 2.35)
Behavioral Disorder	oxcarbazepine	Low Dose	16 / 332	239 / 8887	14.68 (8.46 - 24.80)	11.80 (9.58 - 14.48)	1.34 (0.79 - 2.28)
Behavioral Disorder	oxcarbazepine	No Child Epilepsy	25 / 845	185 / 8,887	9.98 (6.21 - 15.83)	8.89 (7.14 - 11.06)	1.33 (0.85 - 2.09)
Behavioral Disorder	oxcarbazepine	Accounting for Censoring	32 / 845	239 / 8887	**	**	1.45 (0.96 - 2.20)
Behavioral Disorder	valproate	Main (Crude)	49 / 800	239 / 8887	15.66 (11.68 - 20.84)	11.21 (9.72 - 12.90)	1.38 (1.01 - 1.87)
Behavioral Disorder	valproate	Main (Adjusted)	49 / 800	239 / 8887	16.01 (11.91 - 21.33)	12.02 (10.04 - 14.37)	1.31 (0.94 - 1.82)
Behavioral Disorder	valproate	Monotherapy	38 / 561	239 / 8887	17.60 (12.59 - 24.30)	12.52 (10.40 - 15.04)	1.38 (0.95 - 2.00)
Behavioral Disorder	valproate	Strict Monotherapy	36 / 515	239 / 8887	18.12 (12.91 - 25.10)	12.29 (10.16 - 14.83)	1.44 (0.99 - 2.10)
Behavioral Disorder	valproate	>=2 Rx	39 / 568	239 / 8887	16.04 (11.39 - 22.33)	11.93 (9.81 - 14.48)	1.46 (1.01 - 2.11)
Behavioral Disorder	valproate	Active Comparator	84 / 1392	159 / 7381	15.90 (12.31 - 20.40)	12.60 (10.14 - 15.61)	1.23 (0.90 - 1.68)
Behavioral Disorder	valproate	High Dose	37 / 509	239 / 8887	16.08 (11.34 - 22.53)	11.94 (9.84 - 14.46)	1.39 (0.96 - 2.03)
Behavioral Disorder	valproate	Low Dose	*** / 209	239 / 8887	13.58 (7.13 - 25.01)	11.39 (9.27 - 13.96)	1.02 (0.52 - 2.02)
Behavioral Disorder	valproate	No Child Epilepsy	30 / 800	185 / 8,887	9.65 (6.53 - 14.12)	9.28 (7.53 - 11.41)	1.07 (0.71 - 1.61)
Behavioral Disorder	valproate	Accounting for Censoring	49 / 800	239 / 8887	**	**	1.26 (0.90 - 1.77)
Behavioral Disorder	zonisamide	Main (Crude)	16 / 446	239 / 8887	16.13 (9.19 - 27.45)	11.21 (9.72 - 12.90)	1.29 (0.78 - 2.15)
Behavioral Disorder	zonisamide	Main (Adjusted)	16 / 446	239 / 8887	16.73 (9.60 - 28.26)	9.03 (6.84 - 11.88)	1.79 (1.04 - 3.06)
Behavioral Disorder	zonisamide	Monotherapy	*** / 225	228 / 8593	14.39 (5.75 - 33.49)	8.78 (6.46 - 11.89)	1.42 (0.62 - 3.23)
Behavioral Disorder	zonisamide	Polytherapy (no Valproate)	*** / 211	203 / 8007	19.45 (9.73 - 36.68)	9.14 (6.46 - 12.86)	2.08 (1.06 - 4.07)
Behavioral Disorder	zonisamide	Strict Monotherapy	*** / 207	228 / 8593	15.55 (5.83 - 37.82)	8.13 (5.82 - 11.30)	1.42 (0.58 - 3.48)
Behavioral Disorder	zonisamide	>=2 Rx	12 / 355	239 / 8887	12.83 (6.54 - 24.32)	9.81 (7.34 - 13.06)	1.47 (0.79 - 2.74)
Behavioral Disorder	zonisamide	Active Comparator	19 / 508	159 / 7381	14.70 (8.41 - 25.02)	10.77 (8.22 - 14.05)	1.48 (0.87 - 2.51)
Behavioral Disorder	zonisamide	High Dose	12 / 276	239 / 8887	18.19 (9.46 - 33.33)	9.43 (7.03 - 12.58)	1.93 (1.05 - 3.56)
Behavioral Disorder	zonisamide	Low Dose	****	****	****	****	****
Behavioral Disorder	zonisamide	No Child Epilepsy	11 / 446	185 / 8,887	10.45 (5.22 - 20.34)	7.24 (5.29 - 9.87)	1.60 (0.85 - 3.03)
Behavioral Disorder	zonisamide	Accounting for Censoring	16 / 446	239 / 8887	**	**	1.63 (0.91 - 2.91)
Behavioral Disorder	phenobarbital	Main (Crude)	24 / 416	239 / 8887	10.86 (6.78 - 17.16)	11.21 (9.72 - 12.90)	1.03 (0.67 - 1.57)
Behavioral Disorder	phenobarbital	Main (Adjusted)	24 / 416	239 / 8887	11.98 (6.42 - 21.77)	9.66 (5.70 - 16.12)	1.56 (0.83 - 2.92)
Behavioral Disorder	phenobarbital	Monotherapy	19 / 255	237 / 8148	15.89 (8.48 - 28.67)	10.07 (5.26 - 18.82)	2.24 (1.04 - 4.84)
Behavioral Disorder	phenobarbital	Polytherapy (no Valproate)	****	****	****	****	****
Behavioral Disorder	phenobarbital	Strict Monotherapy	****	****	****	****	****
Behavioral Disorder	phenobarbital	>=2 Rx	19 / 319	239 / 8887	12.33 (5.58 - 26.05)	7.83 (3.33 - 17.83)	2.21 (1.06 - 4.62)
Behavioral Disorder	phenobarbital	Active Comparator	51 / 934	159 / 7381	12.27 (7.84 - 18.92)	11.71 (7.98 - 17.02)	0.90 (0.53 - 1.51)
Behavioral Disorder	phenobarbital	High Dose	14 / 221	239 / 8887	14.58 (6.92 - 29.25)	8.52 (3.59 - 19.51)	2.34 (1.01 - 5.42)
Behavioral Disorder	phenobarbital	Low Dose	****	****	****	****	****
Behavioral Disorder	phenobarbital	No Child Epilepsy	20 / 416	185 / 8,887	10.74 (5.56 - 20.18)	7.75 (4.32 - 13.70)	1.63 (0.82 - 3.28)
Behavioral Disorder	phenobarbital	Accounting for Censoring	24 / 416	239 / 8887	**	**	1.63 (0.87 - 3.04)
Behavioral Disorder	lacosamide	Main (Crude)	*** / 219	239 / 8887	3.70 (0.53 - 23.51)	11.21 (9.72 - 12.90)	0.49 (0.07 - 3.52)
Behavioral Disorder	lacosamide	Main (Adjusted)	*** / 219	95 / 6175	3.99 (0.60 - 24.11)	5.31 (2.51 - 11.07)	0.65 (0.09 - 4.72)
Behavioral Disorder	lacosamide	Monotherapy	****	****	****	****	****
Behavioral Disorder	lacosamide	Polytherapy (no Valproate)	****	****	****	****	****
Behavioral Disorder	lacosamide	Strict Monotherapy	****	****	****	****	****
Behavioral Disorder	lacosamide	>=2 Rx	****	****	****	****	****
Behavioral Disorder	lacosamide	Active Comparator	*** / 226	159 / 7381	3.40 (0.47 - 22.49)	8.66 (5.37 - 13.83)	0.45 (0.06 - 3.32)

Outcome	Exposure	Analysis	N Cases/ Total		Cum. Incidence in % (95% CI) at Age 8 Years		HR (95% CI)
			Among Exposed	Among Unexposed	Among Exposed	Among Unexposed	
Behavioral Disorder	lacosamide	High Dose	****	****	****	****	****
Behavioral Disorder	lacosamide	Low Dose	****	****	****	****	****
Behavioral Disorder	lacosamide	No Child Epilepsy	*** / 219	75 / 6,175	3.99 (0.60 - 24.11)	4.80 (2.17 - 10.45)	0.69 (0.09 - 5.09)
Behavioral Disorder	lacosamide	Accounting for Censoring	*** / 219	95 / 6175	**	**	0.78 (0.10 - 6.20)
Coordination Disorder	levetiracetam	Main (Crude)	59 / 5261	91 / 8887	2.84 (1.96 - 4.10)	2.04 (1.56 - 2.66)	1.23 (0.88 - 1.71)
Coordination Disorder	levetiracetam	Main (Adjusted)	59 / 5261	91 / 8887	2.81 (1.85 - 4.26)	2.29 (1.72 - 3.06)	0.96 (0.67 - 1.38)
Coordination Disorder	levetiracetam	Monotherapy	36 / 3852	91 / 8887	2.91 (1.70 - 4.95)	2.36 (1.75 - 3.17)	0.87 (0.57 - 1.32)
Coordination Disorder	levetiracetam	Polytherapy (no Valproate)	20 / 1343	91 / 8887	2.71 (1.46 - 5.00)	2.05 (1.45 - 2.91)	1.25 (0.74 - 2.13)
Coordination Disorder	levetiracetam	Strict Monotherapy	32 / 3360	91 / 8887	3.22 (1.78 - 5.79)	2.36 (1.74 - 3.20)	0.90 (0.58 - 1.41)
Coordination Disorder	levetiracetam	>=2 Rx	49 / 4067	91 / 8887	3.09 (1.95 - 4.88)	2.27 (1.69 - 3.05)	1.03 (0.70 - 1.52)
Coordination Disorder	levetiracetam	Active Comparator	62 / 5487	112 / 7381	2.99 (2.04 - 4.39)	2.63 (1.68 - 4.08)	1.09 (0.73 - 1.64)
Coordination Disorder	levetiracetam	High Dose	41 / 3036	91 / 8887	3.64 (2.26 - 5.85)	2.29 (1.69 - 3.09)	1.15 (0.76 - 1.73)
Coordination Disorder	levetiracetam	Low Dose	15 / 1879	91 / 8887	1.81 (0.81 - 4.03)	2.32 (1.71 - 3.16)	0.73 (0.41 - 1.28)
Coordination Disorder	levetiracetam	No Child Epilepsy	42 / 5,261	71 / 8,887	1.94 (1.13 - 3.32)	1.94 (1.41 - 2.67)	0.82 (0.54 - 1.26)
Coordination Disorder	levetiracetam	Accounting for Censoring	59 / 5261	91 / 8887	**	**	1.16 (0.78 - 1.74)
Coordination Disorder	lamotrigine	Main (Crude)	56 / 4299	91 / 8887	2.75 (1.93 - 3.91)	2.04 (1.56 - 2.66)	1.29 (0.93 - 1.81)
Coordination Disorder	lamotrigine	Main (Adjusted)	56 / 4299	91 / 8887	2.62 (1.71 - 4.01)	2.01 (1.48 - 2.72)	1.06 (0.72 - 1.55)
Coordination Disorder	lamotrigine	Monotherapy	37 / 3200	91 / 8887	2.57 (1.51 - 4.35)	1.98 (1.44 - 2.72)	0.97 (0.62 - 1.50)
Coordination Disorder	lamotrigine	Polytherapy (no Valproate)	18 / 1043	91 / 8887	3.36 (1.80 - 6.23)	1.97 (1.37 - 2.83)	1.54 (0.89 - 2.66)
Coordination Disorder	lamotrigine	Strict Monotherapy	27 / 2864	91 / 8887	1.33 (0.75 - 2.35)	2.00 (1.44 - 2.79)	0.74 (0.45 - 1.22)
Coordination Disorder	lamotrigine	>=2 Rx	49 / 3606	91 / 8887	2.87 (1.82 - 4.52)	2.00 (1.45 - 2.74)	1.10 (0.74 - 1.66)
Coordination Disorder	lamotrigine	High Dose	29 / 2542	91 / 8887	1.77 (1.03 - 3.03)	2.00 (1.42 - 2.81)	0.90 (0.55 - 1.47)
Coordination Disorder	lamotrigine	Low Dose	22 / 1489	91 / 8887	3.77 (2.08 - 6.77)	1.93 (1.40 - 2.65)	1.36 (0.83 - 2.23)
Coordination Disorder	lamotrigine	No Child Epilepsy	45 / 4,299	71 / 8,887	1.52 (0.96 - 2.41)	1.72 (1.22 - 2.40)	1.00 (0.65 - 1.53)
Coordination Disorder	lamotrigine	Accounting for Censoring	56 / 4299	91 / 8887	**	**	1.12 (0.74 - 1.69)
Coordination Disorder	carbamazepine	Main (Crude)	22 / 1924	91 / 8887	2.63 (1.63 - 4.24)	2.04 (1.56 - 2.66)	0.91 (0.57 - 1.46)
Coordination Disorder	carbamazepine	Main (Adjusted)	22 / 1924	91 / 8887	2.80 (1.71 - 4.56)	1.50 (1.01 - 2.22)	1.20 (0.72 - 2.00)
Coordination Disorder	carbamazepine	Monotherapy	16 / 1456	91 / 8887	2.90 (1.64 - 5.13)	1.41 (0.93 - 2.15)	1.25 (0.70 - 2.22)
Coordination Disorder	carbamazepine	Polytherapy (no Valproate)	*** / 435	91 / 8887	2.60 (1.11 - 6.06)	1.51 (0.95 - 2.40)	1.32 (0.56 - 3.12)
Coordination Disorder	carbamazepine	Strict Monotherapy	15 / 1333	91 / 8887	3.11 (1.73 - 5.56)	1.38 (0.90 - 2.12)	1.30 (0.72 - 2.37)
Coordination Disorder	carbamazepine	>=2 Rx	17 / 1545	91 / 8887	2.52 (1.39 - 4.57)	1.44 (0.95 - 2.18)	1.09 (0.62 - 1.93)
Coordination Disorder	carbamazepine	Active Comparator	30 / 2211	112 / 7381	3.91 (2.62 - 5.83)	2.25 (1.34 - 3.77)	1.82 (1.11 - 2.98)
Coordination Disorder	carbamazepine	High Dose	14 / 1200	91 / 8887	2.52 (1.35 - 4.66)	1.52 (1.00 - 2.31)	1.05 (0.57 - 1.92)
Coordination Disorder	carbamazepine	Low Dose	*** / 508	91 / 8887	3.46 (1.55 - 7.67)	1.42 (0.89 - 2.25)	1.83 (0.86 - 3.90)
Coordination Disorder	carbamazepine	No Child Epilepsy	14 / 1,924	71 / 8,887	1.87 (0.98 - 3.55)	1.24 (0.79 - 1.94)	0.91 (0.49 - 1.71)
Coordination Disorder	carbamazepine	Accounting for Censoring	22 / 1924	91 / 8887	**	**	1.23 (0.71 - 2.13)
Coordination Disorder	phenytoin	Main (Crude)	20 / 1595	91 / 8887	2.85 (1.76 - 4.59)	2.04 (1.56 - 2.66)	0.91 (0.56 - 1.47)
Coordination Disorder	phenytoin	Main (Adjusted)	20 / 1595	91 / 8887	2.79 (1.67 - 4.64)	1.64 (1.09 - 2.44)	1.08 (0.63 - 1.86)
Coordination Disorder	phenytoin	Monotherapy	14 / 1177	91 / 8887	2.49 (1.34 - 4.60)	1.58 (1.05 - 2.38)	1.00 (0.54 - 1.84)
Coordination Disorder	phenytoin	Polytherapy (no Valproate)	*** / 382	91 / 8887	4.03 (1.64 - 9.70)	1.69 (1.03 - 2.78)	1.51 (0.64 - 3.57)
Coordination Disorder	phenytoin	Strict Monotherapy	12 / 1060	91 / 8887	2.43 (1.25 - 4.69)	1.58 (1.03 - 2.42)	0.97 (0.50 - 1.88)
Coordination Disorder	phenytoin	>=2 Rx	16 / 1172	91 / 8887	3.36 (1.94 - 5.79)	1.50 (0.98 - 2.29)	1.24 (0.69 - 2.23)
Coordination Disorder	phenytoin	Active Comparator	21 / 1702	112 / 7381	3.10 (1.74 - 5.48)	2.06 (0.99 - 4.28)	1.30 (0.63 - 2.67)
Coordination Disorder	phenytoin	High Dose	*** / 789	91 / 8887	1.14 (0.43 - 3.02)	1.64 (1.05 - 2.54)	0.58 (0.26 - 1.32)
Coordination Disorder	phenytoin	Low Dose	12 / 642	91 / 8887	4.59 (2.48 - 8.43)	1.65 (1.06 - 2.55)	1.80 (0.94 - 3.47)
Coordination Disorder	phenytoin	No Child Epilepsy	12 / 1,595	71 / 8,887	1.28 (0.63 - 2.58)	1.27 (0.79 - 2.04)	0.89 (0.45 - 1.77)
Coordination Disorder	phenytoin	Accounting for Censoring	20 / 1595	91 / 8887	**	**	1.19 (0.66 - 2.13)
Coordination Disorder	topiramate	Main (Crude)	*** / 1036	91 / 8887	2.37 (1.03 - 5.38)	2.04 (1.56 - 2.66)	0.69 (0.33 - 1.42)

Outcome	Exposure	Analysis	N Cases/ Total		Cum. Incidence in % (95% CI) at Age 8 Years		HR (95% CI)
			Among Exposed	Among Unexposed	Among Exposed	Among Unexposed	
Coordination Disorder	topiramate	Main (Adjusted)	*** / 1036	91 / 8887	2.26 (0.94 - 5.40)	1.90 (1.36 - 2.65)	0.63 (0.30 - 1.34)
Coordination Disorder	topiramate	Monotherapy	*** / 626	91 / 8887	2.94 (1.14 - 7.43)	1.89 (1.34 - 2.68)	0.82 (0.35 - 1.91)
Coordination Disorder	topiramate	Polytherapy (no Valproate)	*** / 383	91 / 8887	1.10 (0.23 - 5.29)	1.92 (1.30 - 2.85)	0.41 (0.10 - 1.70)
Coordination Disorder	topiramate	Strict Monotherapy	*** / 565	91 / 8887	1.45 (0.51 - 4.12)	1.90 (1.34 - 2.70)	0.65 (0.23 - 1.81)
Coordination Disorder	topiramate	>=2 Rx	*** / 781	91 / 8887	1.51 (0.59 - 3.83)	1.66 (1.15 - 2.41)	0.69 (0.29 - 1.65)
Coordination Disorder	topiramate	Active Comparator	28 / 2494	112 / 7381	2.47 (1.48 - 4.12)	3.19 (2.14 - 4.74)	0.96 (0.58 - 1.58)
Coordination Disorder	topiramate	High Dose	*** / 617	91 / 8887	1.30 (0.42 - 4.01)	1.83 (1.26 - 2.65)	0.51 (0.18 - 1.46)
Coordination Disorder	topiramate	Low Dose	*** / 363	91 / 8887	3.83 (0.77 - 17.85)	2.20 (1.54 - 3.13)	0.64 (0.20 - 2.09)
Coordination Disorder	topiramate	No Child Epilepsy	*** / 1,036	71 / 8,887	0.40 (0.12 - 1.40)	1.52 (1.03 - 2.23)	0.29 (0.09 - 0.99)
Coordination Disorder	topiramate	Accounting for Censoring	*** / 1036	91 / 8887	**	**	0.84 (0.38 - 1.86)
Coordination Disorder	oxcarbazepine	Main (Crude)	*** / 845	91 / 8887	2.50 (1.06 - 5.83)	2.04 (1.56 - 2.66)	0.76 (0.35 - 1.64)
Coordination Disorder	oxcarbazepine	Main (Adjusted)	*** / 845	91 / 8887	2.31 (0.92 - 5.73)	1.55 (1.06 - 2.27)	0.82 (0.36 - 1.88)
Coordination Disorder	oxcarbazepine	Monotherapy	*** / 579	91 / 8887	2.85 (1.05 - 7.61)	1.47 (0.98 - 2.22)	0.94 (0.37 - 2.39)
Coordination Disorder	oxcarbazepine	Polytherapy (no Valproate)	*	*	*	*	*
Coordination Disorder	oxcarbazepine	Strict Monotherapy	*** / 522	91 / 8887	3.47 (1.28 - 9.27)	1.44 (0.93 - 2.23)	1.14 (0.45 - 2.91)
Coordination Disorder	oxcarbazepine	>=2 Rx	*** / 685	91 / 8887	0.51 (0.14 - 1.83)	1.62 (1.09 - 2.42)	0.41 (0.12 - 1.43)
Coordination Disorder	oxcarbazepine	Active Comparator	*** / 1355	112 / 7381	2.00 (0.94 - 4.23)	3.09 (2.15 - 4.43)	0.59 (0.30 - 1.15)
Coordination Disorder	oxcarbazepine	High Dose	*** / 454	91 / 8887	2.86 (0.90 - 8.87)	1.60 (1.03 - 2.48)	1.07 (0.41 - 2.80)
Coordination Disorder	oxcarbazepine	Low Dose	*** / 332	91 / 8887	1.41 (0.21 - 9.06)	1.29 (0.83 - 2.02)	0.33 (0.05 - 2.33)
Coordination Disorder	oxcarbazepine	No Child Epilepsy	*** / 845	71 / 8,887	1.37 (0.42 - 4.45)	1.30 (0.84 - 1.99)	0.64 (0.22 - 1.85)
Coordination Disorder	oxcarbazepine	Accounting for Censoring	*** / 845	91 / 8887	**	**	0.77 (0.34 - 1.76)
Coordination Disorder	valproate	Main (Crude)	14 / 800	91 / 8887	2.87 (1.64 - 5.01)	2.04 (1.56 - 2.66)	1.42 (0.81 - 2.50)
Coordination Disorder	valproate	Main (Adjusted)	14 / 800	91 / 8887	2.68 (1.47 - 4.86)	1.51 (0.99 - 2.31)	1.67 (0.89 - 3.13)
Coordination Disorder	valproate	Monotherapy	*** / 561	91 / 8887	2.38 (1.07 - 5.27)	1.55 (0.98 - 2.43)	1.30 (0.59 - 2.86)
Coordination Disorder	valproate	Strict Monotherapy	*** / 515	91 / 8887	2.36 (1.00 - 5.53)	1.49 (0.94 - 2.35)	1.19 (0.52 - 2.75)
Coordination Disorder	valproate	>=2 Rx	12 / 568	91 / 8887	2.93 (1.56 - 5.45)	1.45 (0.92 - 2.27)	2.04 (1.03 - 4.04)
Coordination Disorder	valproate	Active Comparator	17 / 1392	112 / 7381	1.98 (1.14 - 3.43)	2.48 (1.51 - 4.07)	1.22 (0.67 - 2.24)
Coordination Disorder	valproate	High Dose	12 / 509	91 / 8887	3.69 (1.97 - 6.85)	1.43 (0.91 - 2.25)	2.24 (1.15 - 4.38)
Coordination Disorder	valproate	Low Dose	*** / 209	91 / 8887	1.01 (0.25 - 4.08)	1.68 (1.05 - 2.69)	1.01 (0.23 - 4.35)
Coordination Disorder	valproate	No Child Epilepsy	*** / 800	71 / 8,887	1.59 (0.70 - 3.62)	1.15 (0.70 - 1.89)	1.20 (0.52 - 2.79)
Coordination Disorder	valproate	Accounting for Censoring	14 / 800	91 / 8887	**	**	1.56 (0.84 - 2.91)
Coordination Disorder	zonisamide	Main (Crude)	*** / 446	91 / 8887	5.21 (1.92 - 13.75)	2.04 (1.56 - 2.66)	1.57 (0.73 - 3.39)
Coordination Disorder	zonisamide	Main (Adjusted)	*** / 446	91 / 8887	5.27 (1.90 - 14.15)	1.86 (1.21 - 2.85)	1.43 (0.65 - 3.16)
Coordination Disorder	zonisamide	Monotherapy	*** / 225	89 / 8593	1.31 (0.29 - 5.86)	1.80 (1.16 - 2.78)	0.70 (0.17 - 2.96)
Coordination Disorder	zonisamide	Polytherapy (no Valproate)	*** / 211	82 / 8007	9.85 (3.37 - 26.89)	1.77 (0.96 - 3.25)	2.83 (1.12 - 7.16)
Coordination Disorder	zonisamide	Strict Monotherapy	*** / 207	89 / 8593	0.43 (0.04 - 4.16)	1.63 (0.98 - 2.69)	0.37 (0.05 - 2.74)
Coordination Disorder	zonisamide	>=2 Rx	*** / 355	91 / 8887	3.34 (1.37 - 7.99)	1.67 (1.04 - 2.68)	1.63 (0.70 - 3.82)
Coordination Disorder	zonisamide	Active Comparator	*** / 508	112 / 7381	5.00 (1.78 - 13.65)	2.41 (1.42 - 4.08)	1.28 (0.57 - 2.89)
Coordination Disorder	zonisamide	High Dose	*** / 276	91 / 8887	6.77 (2.19 - 19.89)	1.65 (1.00 - 2.71)	1.91 (0.77 - 4.76)
Coordination Disorder	zonisamide	Low Dose	****	****	****	****	****
Coordination Disorder	zonisamide	No Child Epilepsy	*** / 446	71 / 8,887	3.85 (1.04 - 13.75)	1.56 (0.96 - 2.52)	1.27 (0.50 - 3.24)
Coordination Disorder	zonisamide	Accounting for Censoring	*** / 446	91 / 8887	**	**	1.72 (0.75 - 3.96)
Coordination Disorder	phenobarbital	Main (Crude)	*** / 416	91 / 8887	1.35 (0.44 - 4.14)	2.04 (1.56 - 2.66)	0.53 (0.17 - 1.68)
Coordination Disorder	phenobarbital	Main (Adjusted)	*** / 416	91 / 8887	2.50 (0.80 - 7.69)	0.98 (0.23 - 4.15)	2.30 (0.54 - 9.84)
Coordination Disorder	phenobarbital	Monotherapy	*** / 255	82 / 8148	1.94 (0.39 - 9.30)	0.62 (0.06 - 5.98)	2.91 (0.39 - 21.82)
Coordination Disorder	phenobarbital	Polytherapy (no Valproate)	****	****	****	****	****
Coordination Disorder	phenobarbital	Strict Monotherapy	****	****	****	****	****
Coordination Disorder	phenobarbital	>=2 Rx	*** / 319	91 / 8887	0.03 (0.00 - 100.00)	0.49 (0.04 - 6.60)	0.04 (0.00 - 0.31)

Outcome	Exposure	Analysis	N Cases/ Total		Cum. Incidence in % (95% CI) at Age 8 Years		HR (95% CI)
			Among Exposed	Among Unexposed	Among Exposed	Among Unexposed	
Coordination Disorder	phenobarbital	Active Comparator	*** / 934	112 / 7381	1.34 (0.46 - 3.84)	2.88 (1.27 - 6.43)	0.60 (0.16 - 2.32)
Coordination Disorder	phenobarbital	High Dose	*** / 221	91 / 8887	2.50 (0.53 - 11.32)	0.40 (0.01 - 11.05)	5.69 (0.83 - 38.94)
Coordination Disorder	phenobarbital	Low Dose	****	****	****	****	****
Coordination Disorder	phenobarbital	No Child Epilepsy	*** / 416	71 / 8,887	1.22 (0.23 - 6.30)	0.85 (0.17 - 4.24)	1.38 (0.17 - 10.93)
Coordination Disorder	phenobarbital	Accounting for Censoring	*** / 416	91 / 8887	**	**	2.47 (0.57 - 10.76)
Coordination Disorder	lacosamide	Main (Crude)	*** / 219	91 / 8887	0.52 (0.07 - 3.66)	2.04 (1.56 - 2.66)	0.59 (0.08 - 4.25)
Coordination Disorder	lacosamide	Main (Adjusted)	*** / 219	68 / 6175	0.60 (0.09 - 3.80)	2.38 (1.17 - 4.82)	0.48 (0.06 - 3.60)
Coordination Disorder	lacosamide	Monotherapy	****	****	****	****	****
Coordination Disorder	lacosamide	Polytherapy (no Valproate)	****	****	****	****	****
Coordination Disorder	lacosamide	Strict Monotherapy	****	****	****	****	****
Coordination Disorder	lacosamide	>=2 Rx	****	****	****	****	****
Coordination Disorder	lacosamide	Active Comparator	*** / 226	112 / 7381	0.60 (0.09 - 3.75)	2.25 (1.14 - 4.42)	0.49 (0.07 - 3.62)
Coordination Disorder	lacosamide	High Dose	****	****	****	****	****
Coordination Disorder	lacosamide	Low Dose	****	****	****	****	****
Coordination Disorder	lacosamide	No Child Epilepsy	*** / 219	58 / 6,175	0.60 (0.09 - 3.80)	2.06 (0.93 - 4.53)	0.56 (0.07 - 4.28)
Coordination Disorder	lacosamide	Accounting for Censoring	*** / 219	68 / 6175	**	**	0.46 (0.06 - 3.36)
Autism Spectrum Disorder	levetiracetam	Main (Crude)	40 / 5261	106 / 8887	3.17 (2.14 - 4.68)	4.07 (3.27 - 5.07)	0.78 (0.54 - 1.13)
Autism Spectrum Disorder	levetiracetam	Main (Adjusted)	40 / 5261	106 / 8887	3.10 (2.02 - 4.74)	4.22 (3.29 - 5.40)	0.67 (0.45 - 1.00)
Autism Spectrum Disorder	levetiracetam	Monotherapy	26 / 3852	106 / 8887	3.03 (1.80 - 5.10)	4.23 (3.25 - 5.49)	0.61 (0.38 - 0.98)
Autism Spectrum Disorder	levetiracetam	Polytherapy (no Valproate)	12 / 1343	106 / 8887	3.38 (1.66 - 6.82)	4.13 (3.10 - 5.48)	0.75 (0.40 - 1.40)
Autism Spectrum Disorder	levetiracetam	Strict Monotherapy	21 / 3360	106 / 8887	3.33 (1.86 - 5.92)	4.22 (3.23 - 5.50)	0.58 (0.35 - 0.97)
Autism Spectrum Disorder	levetiracetam	>=2 Rx	33 / 4067	106 / 8887	3.29 (2.06 - 5.24)	4.24 (3.29 - 5.46)	0.70 (0.46 - 1.09)
Autism Spectrum Disorder	levetiracetam	Active Comparator	41 / 5487	83 / 7381	3.65 (2.41 - 5.51)	4.48 (3.11 - 6.44)	0.79 (0.50 - 1.25)
Autism Spectrum Disorder	levetiracetam	High Dose	29 / 3036	106 / 8887	3.54 (2.19 - 5.69)	4.31 (3.32 - 5.59)	0.80 (0.51 - 1.26)
Autism Spectrum Disorder	levetiracetam	Low Dose	*** / 1879	106 / 8887	2.77 (1.25 - 6.10)	4.21 (3.22 - 5.49)	0.51 (0.27 - 0.99)
Autism Spectrum Disorder	levetiracetam	No Child Epilepsy	36 / 5,261	70 / 8,887	2.71 (1.71 - 4.29)	2.93 (2.19 - 3.91)	0.81 (0.52 - 1.26)
Autism Spectrum Disorder	levetiracetam	Accounting for Censoring	40 / 5261	106 / 8887	**	**	0.79 (0.50 - 1.25)
Autism Spectrum Disorder	lamotrigine	Main (Crude)	55 / 4299	106 / 8887	4.32 (3.16 - 5.89)	4.07 (3.27 - 5.07)	1.07 (0.78 - 1.49)
Autism Spectrum Disorder	lamotrigine	Main (Adjusted)	55 / 4299	106 / 8887	4.84 (3.50 - 6.66)	4.29 (3.33 - 5.51)	1.10 (0.77 - 1.58)
Autism Spectrum Disorder	lamotrigine	Monotherapy	36 / 3200	106 / 8887	5.15 (3.52 - 7.49)	4.35 (3.33 - 5.67)	1.05 (0.69 - 1.58)
Autism Spectrum Disorder	lamotrigine	Polytherapy (no Valproate)	16 / 1043	106 / 8887	4.04 (2.35 - 6.90)	4.25 (3.19 - 5.65)	1.17 (0.66 - 2.08)
Autism Spectrum Disorder	lamotrigine	Strict Monotherapy	28 / 2864	106 / 8887	4.98 (3.29 - 7.49)	4.34 (3.30 - 5.70)	0.93 (0.59 - 1.48)
Autism Spectrum Disorder	lamotrigine	>=2 Rx	42 / 3606	106 / 8887	4.36 (3.02 - 6.27)	4.24 (3.26 - 5.50)	1.01 (0.68 - 1.51)
Autism Spectrum Disorder	lamotrigine	High Dose	32 / 2542	106 / 8887	4.80 (3.13 - 7.31)	4.19 (3.16 - 5.55)	1.04 (0.66 - 1.63)
Autism Spectrum Disorder	lamotrigine	Low Dose	22 / 1489	106 / 8887	4.92 (3.01 - 7.99)	4.62 (3.56 - 5.99)	1.16 (0.71 - 1.88)
Autism Spectrum Disorder	lamotrigine	No Child Epilepsy	33 / 4,299	70 / 8,887	2.64 (1.74 - 4.00)	2.88 (2.13 - 3.88)	0.93 (0.59 - 1.46)
Autism Spectrum Disorder	lamotrigine	Accounting for Censoring	55 / 4299	106 / 8887	**	**	1.25 (0.84 - 1.86)
Autism Spectrum Disorder	carbamazepine	Main (Crude)	30 / 1924	106 / 8887	3.66 (2.44 - 5.47)	4.07 (3.27 - 5.07)	0.90 (0.60 - 1.35)
Autism Spectrum Disorder	carbamazepine	Main (Adjusted)	30 / 1924	106 / 8887	3.80 (2.48 - 5.79)	3.79 (2.87 - 5.01)	0.94 (0.60 - 1.48)
Autism Spectrum Disorder	carbamazepine	Monotherapy	23 / 1456	106 / 8887	3.66 (2.28 - 5.85)	3.86 (2.89 - 5.14)	0.98 (0.60 - 1.61)
Autism Spectrum Disorder	carbamazepine	Polytherapy (no Valproate)	*** / 435	106 / 8887	4.24 (1.77 - 9.98)	3.69 (2.65 - 5.14)	0.90 (0.41 - 1.99)
Autism Spectrum Disorder	carbamazepine	Strict Monotherapy	21 / 1333	106 / 8887	3.40 (2.08 - 5.53)	3.87 (2.88 - 5.18)	1.00 (0.60 - 1.67)
Autism Spectrum Disorder	carbamazepine	>=2 Rx	27 / 1545	106 / 8887	4.08 (2.59 - 6.39)	3.78 (2.82 - 5.05)	1.04 (0.65 - 1.66)
Autism Spectrum Disorder	carbamazepine	Active Comparator	33 / 2211	83 / 7381	4.15 (2.73 - 6.29)	4.60 (3.19 - 6.61)	1.03 (0.61 - 1.73)
Autism Spectrum Disorder	carbamazepine	High Dose	23 / 1200	106 / 8887	4.72 (2.96 - 7.48)	3.82 (2.84 - 5.13)	1.06 (0.65 - 1.73)
Autism Spectrum Disorder	carbamazepine	Low Dose	*** / 508	106 / 8887	2.03 (0.80 - 5.09)	3.99 (2.94 - 5.42)	0.70 (0.30 - 1.62)
Autism Spectrum Disorder	carbamazepine	No Child Epilepsy	15 / 1,924	70 / 8,887	2.06 (1.13 - 3.75)	2.42 (1.72 - 3.39)	0.75 (0.41 - 1.38)
Autism Spectrum Disorder	carbamazepine	Accounting for Censoring	30 / 1924	106 / 8887	**	**	0.96 (0.60 - 1.55)

Outcome	Exposure	Analysis	N Cases/ Total		Cum. Incidence in % (95% CI) at Age 8 Years		HR (95% CI)
			**Among Exposed**	**Among Unexposed**	**Among Exposed**	**Among Unexposed**	
Autism Spectrum Disorder	phenytoin	Main (Crude)	21 / 1595	106 / 8887	2.89 (1.83 - 4.55)	4.07 (3.27 - 5.07)	0.67 (0.42 - 1.08)
Autism Spectrum Disorder	phenytoin	Main (Adjusted)	21 / 1595	106 / 8887	2.79 (1.75 - 4.44)	3.54 (2.64 - 4.74)	0.78 (0.47 - 1.31)
Autism Spectrum Disorder	phenytoin	Monotherapy	11 / 1177	106 / 8887	2.07 (1.08 - 3.96)	3.52 (2.63 - 4.71)	0.54 (0.28 - 1.03)
Autism Spectrum Disorder	phenytoin	Polytherapy (no Valproate)	*** / 382	106 / 8887	4.95 (2.51 - 9.64)	3.37 (2.34 - 4.86)	1.59 (0.75 - 3.36)
Autism Spectrum Disorder	phenytoin	Strict Monotherapy	11 / 1060	106 / 8887	2.29 (1.20 - 4.34)	3.56 (2.64 - 4.79)	0.59 (0.31 - 1.13)
Autism Spectrum Disorder	phenytoin	>=2 Rx	15 / 1172	106 / 8887	2.27 (1.31 - 3.93)	3.45 (2.54 - 4.69)	0.75 (0.41 - 1.35)
Autism Spectrum Disorder	phenytoin	Active Comparator	21 / 1702	83 / 7381	2.48 (1.42 - 4.31)	5.30 (3.48 - 8.04)	0.58 (0.30 - 1.11)
Autism Spectrum Disorder	phenytoin	High Dose	*** / 789	106 / 8887	2.84 (1.45 - 5.51)	3.57 (2.60 - 4.87)	0.69 (0.35 - 1.40)
Autism Spectrum Disorder	phenytoin	Low Dose	*** / 642	106 / 8887	2.79 (1.41 - 5.45)	3.58 (2.60 - 4.91)	0.87 (0.44 - 1.72)
Autism Spectrum Disorder	phenytoin	No Child Epilepsy	16 / 1,595	70 / 8,887	2.09 (1.20 - 3.62)	2.05 (1.41 - 2.97)	0.99 (0.54 - 1.81)
Autism Spectrum Disorder	phenytoin	Accounting for Censoring	21 / 1595	106 / 8887	**	**	0.73 (0.44 - 1.24)
Autism Spectrum Disorder	topiramate	Main (Crude)	18 / 1036	106 / 8887	5.98 (3.60 - 9.84)	4.07 (3.27 - 5.07)	1.22 (0.74 - 2.01)
Autism Spectrum Disorder	topiramate	Main (Adjusted)	18 / 1036	106 / 8887	5.65 (3.32 - 9.52)	4.57 (3.51 - 5.94)	1.02 (0.60 - 1.74)
Autism Spectrum Disorder	topiramate	Monotherapy	*** / 626	106 / 8887	4.91 (2.41 - 9.85)	4.63 (3.50 - 6.10)	0.86 (0.44 - 1.69)
Autism Spectrum Disorder	topiramate	Polytherapy (no Valproate)	*** / 383	106 / 8887	7.13 (2.94 - 16.77)	4.94 (3.63 - 6.72)	1.09 (0.49 - 2.45)
Autism Spectrum Disorder	topiramate	Strict Monotherapy	*** / 565	106 / 8887	5.72 (2.81 - 11.47)	4.67 (3.53 - 6.16)	0.99 (0.50 - 1.94)
Autism Spectrum Disorder	topiramate	>=2 Rx	14 / 781	106 / 8887	6.14 (3.42 - 10.88)	4.50 (3.40 - 5.94)	1.09 (0.60 - 1.97)
Autism Spectrum Disorder	topiramate	Active Comparator	33 / 2494	83 / 7381	5.00 (3.32 - 7.50)	4.24 (2.99 - 6.00)	1.17 (0.73 - 1.87)
Autism Spectrum Disorder	topiramate	High Dose	11 / 617	106 / 8887	5.49 (2.87 - 10.37)	4.66 (3.50 - 6.18)	0.90 (0.47 - 1.73)
Autism Spectrum Disorder	topiramate	Low Dose	*** / 363	106 / 8887	7.30 (2.84 - 18.12)	4.72 (3.51 - 6.35)	1.35 (0.61 - 2.98)
Autism Spectrum Disorder	topiramate	No Child Epilepsy	11 / 1,036	70 / 8,887	3.31 (1.67 - 6.50)	2.80 (1.99 - 3.92)	0.99 (0.51 - 1.92)
Autism Spectrum Disorder	topiramate	Accounting for Censoring	18 / 1036	106 / 8887	**	**	1.05 (0.60 - 1.85)
Autism Spectrum Disorder	oxcarbazepine	Main (Crude)	*** / 845	106 / 8887	3.62 (1.75 - 7.40)	4.07 (3.27 - 5.07)	0.90 (0.47 - 1.72)
Autism Spectrum Disorder	oxcarbazepine	Main (Adjusted)	*** / 845	106 / 8887	3.35 (1.55 - 7.16)	4.12 (3.08 - 5.50)	0.82 (0.41 - 1.64)
Autism Spectrum Disorder	oxcarbazepine	Monotherapy	*** / 579	106 / 8887	4.17 (1.76 - 9.69)	3.92 (2.87 - 5.35)	0.95 (0.43 - 2.09)
Autism Spectrum Disorder	oxcarbazepine	Polytherapy (no Valproate)	*** / 254	106 / 8887	1.60 (0.39 - 6.51)	4.66 (3.26 - 6.63)	0.54 (0.13 - 2.30)
Autism Spectrum Disorder	oxcarbazepine	Strict Monotherapy	*** / 522	106 / 8887	3.48 (1.19 - 9.98)	3.84 (2.78 - 5.30)	0.64 (0.23 - 1.77)
Autism Spectrum Disorder	oxcarbazepine	>=2 Rx	*** / 685	106 / 8887	2.51 (0.91 - 6.82)	4.17 (3.09 - 5.63)	0.55 (0.23 - 1.31)
Autism Spectrum Disorder	oxcarbazepine	Active Comparator	15 / 1355	83 / 7381	4.00 (2.14 - 7.40)	4.56 (3.35 - 6.20)	0.79 (0.44 - 1.40)
Autism Spectrum Disorder	oxcarbazepine	High Dose	*** / 454	106 / 8887	3.18 (1.01 - 9.83)	4.04 (2.90 - 5.62)	0.70 (0.27 - 1.79)
Autism Spectrum Disorder	oxcarbazepine	Low Dose	*** / 332	106 / 8887	3.30 (1.05 - 10.15)	4.35 (3.17 - 5.95)	0.72 (0.27 - 1.93)
Autism Spectrum Disorder	oxcarbazepine	No Child Epilepsy	*** / 845	70 / 8,887	1.63 (0.55 - 4.73)	2.67 (1.88 - 3.80)	0.77 (0.32 - 1.84)
Autism Spectrum Disorder	oxcarbazepine	Accounting for Censoring	*** / 845	106 / 8887	**	**	1.07 (0.51 - 2.26)
Autism Spectrum Disorder	valproate	Main (Crude)	34 / 800	106 / 8887	10.22 (7.24 - 14.32)	4.07 (3.27 - 5.07)	2.53 (1.72 - 3.73)
Autism Spectrum Disorder	valproate	Main (Adjusted)	34 / 800	106 / 8887	9.95 (6.96 - 14.12)	3.75 (2.76 - 5.09)	2.60 (1.67 - 4.04)
Autism Spectrum Disorder	valproate	Monotherapy	26 / 561	106 / 8887	11.61 (7.80 - 17.12)	3.77 (2.73 - 5.20)	2.90 (1.78 - 4.71)
Autism Spectrum Disorder	valproate	Strict Monotherapy	24 / 515	106 / 8887	11.53 (7.61 - 17.26)	3.76 (2.71 - 5.22)	2.89 (1.75 - 4.75)
Autism Spectrum Disorder	valproate	>=2 Rx	32 / 568	106 / 8887	13.25 (9.21 - 18.87)	3.76 (2.71 - 5.22)	3.57 (2.26 - 5.65)
Autism Spectrum Disorder	valproate	Active Comparator	41 / 1392	83 / 7381	7.17 (5.02 - 10.20)	4.99 (3.53 - 7.02)	1.70 (1.08 - 2.67)
Autism Spectrum Disorder	valproate	High Dose	23 / 509	106 / 8887	9.31 (5.99 - 14.33)	3.88 (2.81 - 5.33)	2.46 (1.48 - 4.08)
Autism Spectrum Disorder	valproate	Low Dose	*** / 209	106 / 8887	9.70 (4.62 - 19.75)	3.69 (2.59 - 5.25)	2.43 (1.10 - 5.39)
Autism Spectrum Disorder	valproate	No Child Epilepsy	18 / 800	70 / 8,887	5.46 (3.31 - 8.94)	2.29 (1.56 - 3.35)	2.25 (1.24 - 4.08)
Autism Spectrum Disorder	valproate	Accounting for Censoring	34 / 800	106 / 8887	**	**	2.60 (1.63 - 4.13)
Autism Spectrum Disorder	zonisamide	Main (Crude)	*** / 446	106 / 8887	4.93 (2.02 - 11.78)	4.07 (3.27 - 5.07)	1.13 (0.50 - 2.58)
Autism Spectrum Disorder	zonisamide	Main (Adjusted)	*** / 446	106 / 8887	4.95 (2.02 - 11.88)	4.04 (2.84 - 5.73)	1.08 (0.46 - 2.53)
Autism Spectrum Disorder	zonisamide	Monotherapy	*** / 225	102 / 8593	2.33 (0.60 - 8.82)	3.91 (2.65 - 5.74)	0.73 (0.18 - 3.01)
Autism Spectrum Disorder	zonisamide	Polytherapy (no Valproate)	*** / 211	96 / 8007	7.76 (2.73 - 20.99)	4.20 (2.73 - 6.43)	1.49 (0.53 - 4.17)
Autism Spectrum Disorder	zonisamide	Strict Monotherapy	*** / 207	102 / 8593	2.58 (0.66 - 9.78)	3.80 (2.50 - 5.76)	0.83 (0.20 - 3.44)

Outcome	Exposure	Analysis	N Cases/ Total		Cum. Incidence in % (95% CI) at Age 8 Years		HR (95% CI)
			Among Exposed	Among Unexposed	Among Exposed	Among Unexposed	
Autism Spectrum Disorder	zonisamide	>=2 Rx	*** / 355	106 / 8887	5.91 (2.42 - 14.06)	4.19 (2.88 - 6.08)	1.29 (0.54 - 3.03)
Autism Spectrum Disorder	zonisamide	Active Comparator	*** / 508	83 / 7381	4.13 (1.63 - 10.25)	4.95 (3.39 - 7.19)	0.85 (0.34 - 2.09)
Autism Spectrum Disorder	zonisamide	High Dose	*** / 276	106 / 8887	5.04 (1.61 - 15.17)	4.63 (3.23 - 6.62)	0.97 (0.34 - 2.74)
Autism Spectrum Disorder	zonisamide	Low Dose	****	****	****	****	****
Autism Spectrum Disorder	zonisamide	No Child Epilepsy	*** / 446	70 / 8,887	4.95 (2.02 - 11.88)	2.60 (1.71 - 3.96)	1.58 (0.66 - 3.76)
Autism Spectrum Disorder	zonisamide	Accounting for Censoring	*** / 446	106 / 8887	**	**	1.24 (0.52 - 2.97)
Autism Spectrum Disorder	phenobarbital	Main (Crude)	*** / 416	106 / 8887	4.03 (1.95 - 8.23)	4.07 (3.27 - 5.07)	0.99 (0.48 - 2.03)
Autism Spectrum Disorder	phenobarbital	Main (Adjusted)	*** / 416	106 / 8887	3.93 (1.38 - 10.94)	2.18 (0.84 - 5.58)	1.60 (0.49 - 5.24)
Autism Spectrum Disorder	phenobarbital	Monotherapy	*** / 255	101 / 8148	5.81 (2.07 - 15.70)	2.43 (0.81 - 7.21)	2.07 (0.48 - 8.99)
Autism Spectrum Disorder	phenobarbital	Polytherapy (no Valproate)	****	****	****	****	****
Autism Spectrum Disorder	phenobarbital	Strict Monotherapy	****	****	****	****	****
Autism Spectrum Disorder	phenobarbital	>=2 Rx	*** / 319	106 / 8887	1.67 (0.26 - 10.19)	1.88 (0.43 - 7.94)	1.06 (0.21 - 5.33)
Autism Spectrum Disorder	phenobarbital	Active Comparator	16 / 934	83 / 7381	5.18 (2.89 - 9.19)	7.62 (4.82 - 11.95)	0.91 (0.39 - 2.10)
Autism Spectrum Disorder	phenobarbital	High Dose	*** / 221	106 / 8887	5.61 (1.67 - 17.93)	1.85 (0.39 - 8.49)	2.29 (0.42 - 12.41)
Autism Spectrum Disorder	phenobarbital	Low Dose	****	****	****	****	****
Autism Spectrum Disorder	phenobarbital	No Child Epilepsy	*** / 416	70 / 8,887	2.99 (0.84 - 10.33)	1.62 (0.56 - 4.61)	1.36 (0.29 - 6.37)
Autism Spectrum Disorder	phenobarbital	Accounting for Censoring	*** / 416	106 / 8887	**	**	1.58 (0.51 - 4.90)
Autism Spectrum Disorder	lacosamide	Main (Crude)	*** / 219	106 / 8887	0.68 (0.10 - 4.76)	4.07 (3.27 - 5.07)	0.71 (0.10 - 5.07)
Autism Spectrum Disorder	lacosamide	Main (Adjusted)	*** / 219	65 / 6175	0.77 (0.12 - 4.96)	3.50 (1.52 - 7.97)	0.69 (0.09 - 5.23)
Autism Spectrum Disorder	lacosamide	Monotherapy	****	****	****	****	****
Autism Spectrum Disorder	lacosamide	Polytherapy (no Valproate)	****	****	****	****	****
Autism Spectrum Disorder	lacosamide	Strict Monotherapy	****	****	****	****	****
Autism Spectrum Disorder	lacosamide	>=2 Rx	****	****	****	****	****
Autism Spectrum Disorder	lacosamide	Active Comparator	*** / 226	83 / 7381	0.62 (0.08 - 4.91)	4.94 (2.78 - 8.69)	0.53 (0.07 - 3.97)
Autism Spectrum Disorder	lacosamide	High Dose	****	****	****	****	****
Autism Spectrum Disorder	lacosamide	Low Dose	****	****	****	****	****
Autism Spectrum Disorder	lacosamide	No Child Epilepsy	*** / 219	52 / 6,175	0.77 (0.12 - 4.96)	3.15 (1.29 - 7.59)	0.78 (0.10 - 5.92)
Autism Spectrum Disorder	lacosamide	Accounting for Censoring	*** / 219	65 / 6175	**	**	0.72 (0.10 - 5.07)
Learning Difficulty	levetiracetam	Main (Crude)	15 / 5261	43 / 8887	2.10 (1.09 - 4.01)	1.50 (0.97 - 2.31)	0.96 (0.53 - 1.74)
Learning Difficulty	levetiracetam	Main (Adjusted)	15 / 5261	43 / 8887	2.34 (1.21 - 4.49)	1.32 (0.76 - 2.28)	1.01 (0.52 - 1.94)
Learning Difficulty	levetiracetam	Monotherapy	*** / 3852	43 / 8887	2.52 (1.16 - 5.44)	1.31 (0.73 - 2.35)	0.93 (0.42 - 2.07)
Learning Difficulty	levetiracetam	Polytherapy (no Valproate)	*** / 1343	43 / 8887	1.00 (0.22 - 4.42)	1.31 (0.68 - 2.50)	0.86 (0.30 - 2.51)
Learning Difficulty	levetiracetam	Strict Monotherapy	*** / 3360	43 / 8887	3.18 (1.46 - 6.87)	1.30 (0.71 - 2.36)	1.03 (0.44 - 2.39)
Learning Difficulty	levetiracetam	>=2 Rx	12 / 4067	43 / 8887	2.42 (1.15 - 5.07)	1.31 (0.74 - 2.33)	1.04 (0.51 - 2.12)
Learning Difficulty	levetiracetam	Active Comparator	18 / 5487	21 / 7381	2.92 (1.65 - 5.13)	2.29 (1.18 - 4.41)	1.42 (0.68 - 3.00)
Learning Difficulty	levetiracetam	High Dose	*** / 3036	43 / 8887	1.98 (0.83 - 4.69)	1.33 (0.73 - 2.40)	0.99 (0.43 - 2.24)
Learning Difficulty	levetiracetam	Low Dose	*** / 1879	43 / 8887	3.03 (1.17 - 7.71)	1.30 (0.72 - 2.34)	0.97 (0.39 - 2.42)
Learning Difficulty	levetiracetam	No Child Epilepsy	*** / 5,261	31 / 8,887	1.51 (0.64 - 3.50)	0.93 (0.50 - 1.74)	0.93 (0.42 - 2.05)
Learning Difficulty	levetiracetam	Accounting for Censoring	15 / 5261	43 / 8887	**	**	1.27 (0.60 - 2.69)
Learning Difficulty	lamotrigine	Main (Crude)	18 / 4299	43 / 8887	2.03 (1.11 - 3.72)	1.50 (0.97 - 2.31)	0.94 (0.54 - 1.62)
Learning Difficulty	lamotrigine	Main (Adjusted)	18 / 4299	43 / 8887	2.27 (1.20 - 4.29)	1.31 (0.75 - 2.29)	1.10 (0.59 - 2.05)
Learning Difficulty	lamotrigine	Monotherapy	12 / 3200	43 / 8887	2.51 (1.25 - 5.02)	1.26 (0.69 - 2.30)	0.99 (0.48 - 2.05)
Learning Difficulty	lamotrigine	Polytherapy (no Valproate)	*** / 1043	43 / 8887	1.86 (0.48 - 7.03)	1.53 (0.84 - 2.80)	1.27 (0.45 - 3.61)
Learning Difficulty	lamotrigine	Strict Monotherapy	*** / 2864	43 / 8887	2.39 (1.12 - 5.03)	1.22 (0.65 - 2.30)	0.91 (0.42 - 1.97)
Learning Difficulty	lamotrigine	>=2 Rx	17 / 3606	43 / 8887	2.38 (1.22 - 4.63)	1.35 (0.76 - 2.40)	1.23 (0.64 - 2.36)
Learning Difficulty	lamotrigine	High Dose	14 / 2542	43 / 8887	2.82 (1.37 - 5.77)	1.35 (0.72 - 2.52)	1.38 (0.68 - 2.80)
Learning Difficulty	lamotrigine	Low Dose	*** / 1489	43 / 8887	1.34 (0.34 - 5.20)	1.29 (0.72 - 2.31)	0.63 (0.22 - 1.86)
Learning Difficulty	lamotrigine	No Child Epilepsy	13 / 4,299	31 / 8,887	1.33 (0.58 - 3.01)	0.92 (0.49 - 1.73)	1.08 (0.51 - 2.28)

Outcome	Exposure	Analysis	N Cases/ Total		Cum. Incidence in % (95% CI) at Age 8 Years		HR (95% CI)
			Among Exposed	Among Unexposed	Among Exposed	Among Unexposed	
Learning Difficulty	lamotrigine	Accounting for Censoring	18 / 4299	43 / 8887	**	**	1.26 (0.64 - 2.48)
Learning Difficulty	carbamazepine	Main (Crude)	19 / 1924	43 / 8887	1.65 (0.83 - 3.25)	1.50 (0.97 - 2.31)	1.12 (0.65 - 1.93)
Learning Difficulty	carbamazepine	Main (Adjusted)	19 / 1924	43 / 8887	1.78 (0.89 - 3.55)	1.32 (0.76 - 2.28)	1.16 (0.64 - 2.10)
Learning Difficulty	carbamazepine	Monotherapy	17 / 1456	43 / 8887	2.21 (1.07 - 4.52)	1.29 (0.73 - 2.29)	1.43 (0.77 - 2.67)
Learning Difficulty	carbamazepine	Polytherapy (no Valproate)	*** / 435	43 / 8887	0.59 (0.08 - 4.34)	1.33 (0.68 - 2.59)	0.52 (0.12 - 2.17)
Learning Difficulty	carbamazepine	Strict Monotherapy	13 / 1333	43 / 8887	2.45 (1.19 - 5.01)	1.31 (0.74 - 2.33)	1.15 (0.58 - 2.29)
Learning Difficulty	carbamazepine	>=2 Rx	15 / 1545	43 / 8887	1.89 (0.87 - 4.07)	1.34 (0.76 - 2.36)	1.15 (0.60 - 2.20)
Learning Difficulty	carbamazepine	Active Comparator	19 / 2211	21 / 7381	1.28 (0.52 - 3.13)	2.12 (1.09 - 4.10)	0.89 (0.40 - 2.00)
Learning Difficulty	carbamazepine	High Dose	13 / 1200	43 / 8887	1.96 (0.86 - 4.45)	1.28 (0.71 - 2.31)	1.17 (0.60 - 2.28)
Learning Difficulty	carbamazepine	Low Dose	*** / 508	43 / 8887	1.37 (0.44 - 4.24)	1.16 (0.61 - 2.21)	1.72 (0.71 - 4.16)
Learning Difficulty	carbamazepine	No Child Epilepsy	11 / 1,924	31 / 8,887	1.05 (0.41 - 2.68)	0.92 (0.50 - 1.71)	0.98 (0.46 - 2.08)
Learning Difficulty	carbamazepine	Accounting for Censoring	19 / 1924	43 / 8887	**	**	1.27 (0.69 - 2.32)
Learning Difficulty	phenytoin	Main (Crude)	15 / 1595	43 / 8887	2.01 (1.07 - 3.77)	1.50 (0.97 - 2.31)	0.84 (0.47 - 1.52)
Learning Difficulty	phenytoin	Main (Adjusted)	15 / 1595	43 / 8887	1.84 (0.93 - 3.64)	1.58 (0.97 - 2.58)	0.73 (0.39 - 1.37)
Learning Difficulty	phenytoin	Monotherapy	*** / 1177	43 / 8887	1.87 (0.86 - 4.08)	1.58 (0.96 - 2.59)	0.67 (0.33 - 1.39)
Learning Difficulty	phenytoin	Polytherapy (no Valproate)	*** / 382	43 / 8887	2.36 (0.69 - 7.85)	1.48 (0.81 - 2.70)	0.94 (0.31 - 2.80)
Learning Difficulty	phenytoin	Strict Monotherapy	*** / 1060	43 / 8887	2.00 (0.91 - 4.37)	1.66 (1.01 - 2.71)	0.66 (0.31 - 1.41)
Learning Difficulty	phenytoin	>=2 Rx	11 / 1172	43 / 8887	1.52 (0.64 - 3.59)	1.49 (0.88 - 2.51)	0.68 (0.33 - 1.38)
Learning Difficulty	phenytoin	Active Comparator	16 / 1702	21 / 7381	1.21 (0.46 - 3.19)	2.28 (1.04 - 4.97)	0.83 (0.34 - 2.06)
Learning Difficulty	phenytoin	High Dose	*** / 789	43 / 8887	1.72 (0.67 - 4.38)	1.41 (0.81 - 2.45)	0.72 (0.32 - 1.63)
Learning Difficulty	phenytoin	Low Dose	*** / 642	43 / 8887	2.11 (0.78 - 5.66)	1.58 (0.94 - 2.66)	0.88 (0.38 - 2.01)
Learning Difficulty	phenytoin	No Child Epilepsy	13 / 1,595	31 / 8,887	1.52 (0.70 - 3.27)	1.21 (0.69 - 2.09)	0.84 (0.42 - 1.69)
Learning Difficulty	phenytoin	Accounting for Censoring	15 / 1595	43 / 8887	**	**	0.76 (0.40 - 1.43)
Learning Difficulty	topiramate	Main (Crude)	*** / 1036	43 / 8887	2.73 (1.16 - 6.36)	1.50 (0.97 - 2.31)	1.25 (0.59 - 2.66)
Learning Difficulty	topiramate	Main (Adjusted)	*** / 1036	43 / 8887	2.60 (1.05 - 6.34)	1.29 (0.71 - 2.33)	1.23 (0.54 - 2.79)
Learning Difficulty	topiramate	Monotherapy	*** / 626	43 / 8887	1.60 (0.39 - 6.41)	1.45 (0.79 - 2.63)	0.76 (0.23 - 2.52)
Learning Difficulty	topiramate	Polytherapy (no Valproate)	*** / 383	43 / 8887	2.56 (0.70 - 9.13)	1.21 (0.58 - 2.52)	1.88 (0.63 - 5.64)
Learning Difficulty	topiramate	Strict Monotherapy	*** / 565	43 / 8887	1.09 (0.15 - 7.45)	1.49 (0.82 - 2.71)	0.58 (0.14 - 2.39)
Learning Difficulty	topiramate	>=2 Rx	*** / 781	43 / 8887	1.56 (0.52 - 4.62)	1.33 (0.72 - 2.46)	1.14 (0.45 - 2.87)
Learning Difficulty	topiramate	Active Comparator	11 / 2494	21 / 7381	2.03 (0.95 - 4.28)	1.76 (0.92 - 3.36)	1.21 (0.53 - 2.72)
Learning Difficulty	topiramate	High Dose	*** / 617	43 / 8887	2.37 (0.75 - 7.30)	1.41 (0.76 - 2.63)	1.24 (0.50 - 3.12)
Learning Difficulty	topiramate	Low Dose	*** / 363	43 / 8887	2.79 (0.53 - 13.93)	1.17 (0.57 - 2.40)	1.22 (0.28 - 5.31)
Learning Difficulty	topiramate	No Child Epilepsy	*** / 1,036	31 / 8,887	1.87 (0.62 - 5.55)	0.99 (0.52 - 1.89)	1.19 (0.46 - 3.09)
Learning Difficulty	topiramate	Accounting for Censoring	*** / 1036	43 / 8887	**	**	1.49 (0.64 - 3.49)
Learning Difficulty	oxcarbazepine	Main (Crude)	*** / 845	43 / 8887	1.69 (0.51 - 5.54)	1.50 (0.97 - 2.31)	1.01 (0.40 - 2.55)
Learning Difficulty	oxcarbazepine	Main (Adjusted)	*** / 845	43 / 8887	1.91 (0.60 - 5.99)	1.07 (0.53 - 2.14)	1.09 (0.39 - 3.05)
Learning Difficulty	oxcarbazepine	Monotherapy	*** / 579	43 / 8887	1.41 (0.35 - 5.53)	1.21 (0.60 - 2.41)	0.90 (0.26 - 3.20)
Learning Difficulty	oxcarbazepine	Polytherapy (no Valproate)	*** / 254	43 / 8887	7.48 (3.11 - 17.41)	0.75 (0.26 - 2.15)	0.61 (0.07 - 5.07)
Learning Difficulty	oxcarbazepine	Strict Monotherapy	*** / 522	43 / 8887	1.68 (0.42 - 6.60)	1.25 (0.61 - 2.56)	1.18 (0.35 - 4.01)
Learning Difficulty	oxcarbazepine	>=2 Rx	*** / 685	43 / 8887	1.54 (0.35 - 6.76)	1.10 (0.53 - 2.28)	1.10 (0.36 - 3.36)
Learning Difficulty	oxcarbazepine	Active Comparator	*** / 1355	21 / 7381	1.55 (0.49 - 4.88)	1.97 (1.10 - 3.51)	0.88 (0.34 - 2.27)
Learning Difficulty	oxcarbazepine	High Dose	*** / 454	43 / 8887	2.46 (0.55 - 10.61)	1.16 (0.54 - 2.50)	1.85 (0.57 - 5.98)
Learning Difficulty	oxcarbazepine	Low Dose	*** / 332	43 / 8887	1.35 (0.21 - 8.63)	0.95 (0.42 - 2.16)	0.48 (0.06 - 3.75)
Learning Difficulty	oxcarbazepine	No Child Epilepsy	*** / 845	31 / 8,887	1.22 (0.28 - 5.29)	0.75 (0.34 - 1.66)	1.08 (0.33 - 3.54)
Learning Difficulty	oxcarbazepine	Accounting for Censoring	*** / 845	43 / 8887	**	**	1.40 (0.48 - 4.10)
Learning Difficulty	valproate	Main (Crude)	15 / 800	43 / 8887	3.34 (1.56 - 7.06)	1.50 (0.97 - 2.31)	1.90 (1.05 - 3.44)
Learning Difficulty	valproate	Main (Adjusted)	15 / 800	43 / 8887	3.17 (1.42 - 7.03)	1.44 (0.83 - 2.51)	1.49 (0.77 - 2.88)
Learning Difficulty	valproate	Monotherapy	*** / 561	43 / 8887	1.38 (0.34 - 5.54)	1.46 (0.81 - 2.60)	1.09 (0.49 - 2.40)

Outcome	Exposure	Analysis	N Cases/ Total		Cum. Incidence in % (95% CI) at Age 8 Years		HR (95% CI)
			Among Exposed	Among Unexposed	Among Exposed	Among Unexposed	
Learning Difficulty	valproate	Strict Monotherapy	*** / 515	43 / 8887	0.98 (0.17 - 5.42)	1.47 (0.82 - 2.65)	1.11 (0.49 - 2.54)
Learning Difficulty	valproate	>=2 Rx	14 / 568	43 / 8887	4.60 (2.09 - 9.99)	1.37 (0.75 - 2.51)	2.13 (1.07 - 4.24)
Learning Difficulty	valproate	Active Comparator	20 / 1392	21 / 7381	2.16 (0.97 - 4.79)	1.72 (0.84 - 3.49)	1.79 (0.85 - 3.79)
Learning Difficulty	valproate	High Dose	13 / 509	43 / 8887	3.92 (1.66 - 9.09)	1.33 (0.73 - 2.43)	1.73 (0.85 - 3.49)
Learning Difficulty	valproate	Low Dose	*** / 209	43 / 8887	1.75 (0.21 - 13.78)	1.30 (0.67 - 2.53)	0.56 (0.08 - 4.07)
Learning Difficulty	valproate	No Child Epilepsy	*** / 800	31 / 8,887	2.03 (0.71 - 5.77)	1.09 (0.59 - 1.99)	0.93 (0.37 - 2.32)
Learning Difficulty	valproate	Accounting for Censoring	15 / 800	43 / 8887	**	**	1.53 (0.77 - 3.02)
Learning Difficulty	zonisamide	Main (Crude)	*	*	*	*	*
Learning Difficulty	zonisamide	Main (Adjusted)	*	*	*	*	*
Learning Difficulty	zonisamide	Monotherapy	*	*	*	*	*
Learning Difficulty	zonisamide	Polytherapy (no Valproate)	*	*	*	*	*
Learning Difficulty	zonisamide	Strict Monotherapy	*	*	*	*	*
Learning Difficulty	zonisamide	>=2 Rx	*	*	*	*	*
Learning Difficulty	zonisamide	Active Comparator	*	*	*	*	*
Learning Difficulty	zonisamide	High Dose	*	*	*	*	*
Learning Difficulty	zonisamide	Low Dose	****	****	****	****	****
Learning Difficulty	zonisamide	No Child Epilepsy	*	*	*	*	*
Learning Difficulty	zonisamide	Accounting for Censoring	*	*	*	*	*
Learning Difficulty	phenobarbital	Main (Crude)	*** / 416	43 / 8887	2.28 (0.74 - 6.95)	1.50 (0.97 - 2.31)	0.75 (0.27 - 2.11)
Learning Difficulty	phenobarbital	Main (Adjusted)	*** / 416	43 / 8887	0.76 (0.05 - 11.50)	1.22 (0.35 - 4.22)	0.27 (0.07 - 0.98)
Learning Difficulty	phenobarbital	Monotherapy	*** / 255	43 / 8148	0.92 (0.05 - 16.68)	1.46 (0.37 - 5.63)	0.31 (0.08 - 1.24)
Learning Difficulty	phenobarbital	Polytherapy (no Valproate)	****	****	****	****	****
Learning Difficulty	phenobarbital	Strict Monotherapy	****	****	****	****	****
Learning Difficulty	phenobarbital	>=2 Rx	*** / 319	43 / 8887	2.31 (0.29 - 17.22)	0.97 (0.13 - 6.97)	0.92 (0.21 - 3.96)
Learning Difficulty	phenobarbital	Active Comparator	11 / 934	21 / 7381	1.42 (0.36 - 5.44)	1.72 (0.56 - 5.20)	1.44 (0.45 - 4.57)
Learning Difficulty	phenobarbital	High Dose	*** / 221	43 / 8887	0.96 (0.04 - 19.91)	0.93 (0.13 - 6.47)	0.61 (0.13 - 2.85)
Learning Difficulty	phenobarbital	Low Dose	****	****	****	****	****
Learning Difficulty	phenobarbital	No Child Epilepsy	*** / 416	31 / 8,887	0.28 (0.00 - 28.43)	1.05 (0.30 - 3.62)	0.20 (0.04 - 1.07)
Learning Difficulty	phenobarbital	Accounting for Censoring	*** / 416	43 / 8887	**	**	0.35 (0.09 - 1.37)
Learning Difficulty	lacosamide	Main (Crude)	*	*	*	*	*
Learning Difficulty	lacosamide	Main (Adjusted)	*	*	*	*	*
Learning Difficulty	lacosamide	Monotherapy	****	****	****	****	****
Learning Difficulty	lacosamide	Polytherapy (no Valproate)	****	****	****	****	****
Learning Difficulty	lacosamide	Strict Monotherapy	****	****	****	****	****
Learning Difficulty	lacosamide	>=2 Rx	****	****	****	****	****
Learning Difficulty	lacosamide	Active Comparator	*	*	*	*	*
Learning Difficulty	lacosamide	High Dose	****	****	****	****	****
Learning Difficulty	lacosamide	Low Dose	****	****	****	****	****
Learning Difficulty	lacosamide	No Child Epilepsy	*	*	*	*	*
Learning Difficulty	lacosamide	Accounting for Censoring	*	*	*	*	*
Intellectual Disability	levetiracetam	Main (Crude)	*** / 5261	26 / 8887	0.70 (0.29 - 1.67)	0.96 (0.59 - 1.59)	0.83 (0.39 - 1.77)
Intellectual Disability	levetiracetam	Main (Adjusted)	*** / 5261	26 / 8887	0.73 (0.32 - 1.69)	0.81 (0.44 - 1.48)	1.14 (0.49 - 2.64)
Intellectual Disability	levetiracetam	Monotherapy	*** / 3852	26 / 8887	0.47 (0.15 - 1.50)	0.83 (0.45 - 1.55)	0.67 (0.21 - 2.16)
Intellectual Disability	levetiracetam	Polytherapy (no Valproate)	*** / 1343	26 / 8887	1.16 (0.33 - 4.10)	0.70 (0.33 - 1.46)	1.90 (0.64 - 5.61)
Intellectual Disability	levetiracetam	Strict Monotherapy	*** / 3360	26 / 8887	0.46 (0.11 - 1.85)	0.82 (0.44 - 1.55)	0.60 (0.16 - 2.25)
Intellectual Disability	levetiracetam	>=2 Rx	*** / 4067	26 / 8887	0.83 (0.34 - 2.05)	0.78 (0.42 - 1.46)	1.27 (0.52 - 3.10)
Intellectual Disability	levetiracetam	Active Comparator	*** / 5487	21 / 7381	0.66 (0.24 - 1.82)	2.21 (1.19 - 4.11)	0.42 (0.18 - 1.03)
Intellectual Disability	levetiracetam	High Dose	*** / 3036	26 / 8887	1.01 (0.36 - 2.80)	0.78 (0.41 - 1.50)	1.25 (0.46 - 3.39)

Outcome	Exposure	Analysis	N Cases/ Total		Cum. Incidence in % (95% CI) at Age 8 Years		HR (95% CI)
			Among Exposed	Among Unexposed	Among Exposed	Among Unexposed	
Intellectual Disability	levetiracetam	Low Dose	*** / 1879	26 / 8887	0.38 (0.10 - 1.41)	0.86 (0.47 - 1.57)	0.65 (0.14 - 2.90)
Intellectual Disability	levetiracetam	No Child Epilepsy	*** / 5,261	15 / 8,887	0.51 (0.16 - 1.57)	0.36 (0.14 - 0.92)	0.98 (0.29 - 3.34)
Intellectual Disability	levetiracetam	Accounting for Censoring	*** / 5261	26 / 8887	**	**	1.25 (0.52 - 2.99)
Intellectual Disability	lamotrigine	Main (Crude)	16 / 4299	26 / 8887	1.63 (0.89 - 3.01)	0.96 (0.59 - 1.59)	1.33 (0.71 - 2.47)
Intellectual Disability	lamotrigine	Main (Adjusted)	16 / 4299	26 / 8887	2.20 (1.24 - 3.88)	0.62 (0.31 - 1.22)	2.38 (1.20 - 4.73)
Intellectual Disability	lamotrigine	Monotherapy	14 / 3200	26 / 8887	2.71 (1.48 - 4.95)	0.58 (0.28 - 1.19)	2.86 (1.38 - 5.90)
Intellectual Disability	lamotrigine	Polytherapy (no Valproate)	*** / 1043	26 / 8887	0.67 (0.17 - 2.61)	0.60 (0.27 - 1.35)	1.21 (0.27 - 5.30)
Intellectual Disability	lamotrigine	Strict Monotherapy	12 / 2864	26 / 8887	2.40 (1.23 - 4.67)	0.54 (0.25 - 1.17)	2.96 (1.37 - 6.38)
Intellectual Disability	lamotrigine	>=2 Rx	12 / 3606	26 / 8887	2.26 (1.20 - 4.25)	0.58 (0.28 - 1.19)	2.15 (1.01 - 4.61)
Intellectual Disability	lamotrigine	High Dose	*** / 2542	26 / 8887	2.03 (0.99 - 4.18)	0.54 (0.24 - 1.19)	2.50 (1.06 - 5.89)
Intellectual Disability	lamotrigine	Low Dose	*** / 1489	26 / 8887	2.30 (0.91 - 5.73)	0.68 (0.35 - 1.32)	2.32 (0.94 - 5.70)
Intellectual Disability	lamotrigine	No Child Epilepsy	*** / 4,299	15 / 8,887	1.28 (0.63 - 2.61)	0.31 (0.12 - 0.83)	2.53 (1.07 - 6.01)
Intellectual Disability	lamotrigine	Accounting for Censoring	16 / 4299	26 / 8887	**	**	2.47 (1.20 - 5.08)
Intellectual Disability	carbamazepine	Main (Crude)	*** / 1924	26 / 8887	1.08 (0.46 - 2.51)	0.96 (0.59 - 1.59)	0.95 (0.44 - 2.03)
Intellectual Disability	carbamazepine	Main (Adjusted)	*** / 1924	26 / 8887	0.98 (0.39 - 2.46)	0.79 (0.41 - 1.52)	0.84 (0.36 - 1.95)
Intellectual Disability	carbamazepine	Monotherapy	*** / 1456	26 / 8887	0.74 (0.25 - 2.20)	0.80 (0.41 - 1.56)	0.80 (0.30 - 2.12)
Intellectual Disability	carbamazepine	Polytherapy (no Valproate)	*** / 435	26 / 8887	2.03 (0.49 - 8.28)	0.63 (0.27 - 1.51)	1.07 (0.25 - 4.61)
Intellectual Disability	carbamazepine	Strict Monotherapy	*** / 1333	26 / 8887	0.86 (0.29 - 2.51)	0.79 (0.40 - 1.54)	0.95 (0.36 - 2.53)
Intellectual Disability	carbamazepine	>=2 Rx	*** / 1545	26 / 8887	1.25 (0.50 - 3.08)	0.77 (0.39 - 1.53)	1.10 (0.47 - 2.59)
Intellectual Disability	carbamazepine	Active Comparator	*** / 2211	21 / 7381	0.92 (0.33 - 2.57)	2.45 (1.39 - 4.29)	0.31 (0.11 - 0.89)
Intellectual Disability	carbamazepine	High Dose	*** / 1200	26 / 8887	1.39 (0.54 - 3.56)	0.82 (0.41 - 1.64)	1.02 (0.42 - 2.48)
Intellectual Disability	carbamazepine	Low Dose	*** / 508	26 / 8887	0.34 (0.05 - 2.22)	0.71 (0.34 - 1.50)	0.53 (0.07 - 4.03)
Intellectual Disability	carbamazepine	No Child Epilepsy	*** / 1,924	15 / 8,887	0.42 (0.09 - 2.03)	0.55 (0.25 - 1.21)	0.31 (0.08 - 1.20)
Intellectual Disability	carbamazepine	Accounting for Censoring	*** / 1924	26 / 8887	**	**	0.86 (0.37 - 2.01)
Intellectual Disability	phenytoin	Main (Crude)	11 / 1595	26 / 8887	1.50 (0.76 - 2.94)	0.96 (0.59 - 1.59)	1.16 (0.57 - 2.37)
Intellectual Disability	phenytoin	Main (Adjusted)	11 / 1595	26 / 8887	1.57 (0.77 - 3.16)	1.06 (0.60 - 1.90)	1.05 (0.49 - 2.24)
Intellectual Disability	phenytoin	Monotherapy	*** / 1177	26 / 8887	1.73 (0.81 - 3.70)	1.02 (0.56 - 1.84)	1.24 (0.56 - 2.74)
Intellectual Disability	phenytoin	Polytherapy (no Valproate)	*** / 382	26 / 8887	0.11 (0.00 - 6.30)	1.16 (0.57 - 2.33)	0.11 (0.01 - 0.86)
Intellectual Disability	phenytoin	Strict Monotherapy	*** / 1060	26 / 8887	1.71 (0.76 - 3.84)	1.01 (0.55 - 1.85)	1.26 (0.54 - 2.90)
Intellectual Disability	phenytoin	>=2 Rx	*** / 1172	26 / 8887	1.24 (0.52 - 2.91)	1.02 (0.55 - 1.88)	0.91 (0.37 - 2.26)
Intellectual Disability	phenytoin	Active Comparator	13 / 1702	21 / 7381	1.34 (0.52 - 3.43)	3.16 (1.70 - 5.84)	0.48 (0.19 - 1.22)
Intellectual Disability	phenytoin	High Dose	*** / 789	26 / 8887	1.06 (0.35 - 3.16)	1.02 (0.53 - 1.93)	0.87 (0.31 - 2.46)
Intellectual Disability	phenytoin	Low Dose	*** / 642	26 / 8887	1.77 (0.63 - 4.91)	1.04 (0.55 - 1.95)	1.28 (0.48 - 3.42)
Intellectual Disability	phenytoin	No Child Epilepsy	*** / 1,595	15 / 8,887	1.37 (0.64 - 2.91)	0.63 (0.30 - 1.34)	1.35 (0.55 - 3.30)
Intellectual Disability	phenytoin	Accounting for Censoring	11 / 1595	26 / 8887	**	**	1.02 (0.46 - 2.25)
Intellectual Disability	topiramate	Main (Crude)	*** / 1036	26 / 8887	2.30 (1.04 - 5.04)	0.96 (0.59 - 1.59)	1.86 (0.81 - 4.29)
Intellectual Disability	topiramate	Main (Adjusted)	*** / 1036	26 / 8887	2.12 (0.93 - 4.79)	0.86 (0.46 - 1.63)	1.84 (0.75 - 4.51)
Intellectual Disability	topiramate	Monotherapy	*** / 626	26 / 8887	2.51 (0.99 - 6.31)	0.93 (0.49 - 1.75)	2.09 (0.75 - 5.83)
Intellectual Disability	topiramate	Polytherapy (no Valproate)	*** / 383	26 / 8887	1.75 (0.32 - 9.31)	0.71 (0.30 - 1.67)	1.58 (0.36 - 6.92)
Intellectual Disability	topiramate	Strict Monotherapy	*** / 565	26 / 8887	2.52 (0.90 - 6.96)	0.92 (0.48 - 1.77)	2.02 (0.67 - 6.13)
Intellectual Disability	topiramate	>=2 Rx	*** / 781	26 / 8887	1.84 (0.70 - 4.81)	0.82 (0.42 - 1.59)	1.75 (0.63 - 4.88)
Intellectual Disability	topiramate	Active Comparator	*** / 2494	21 / 7381	1.38 (0.60 - 3.15)	1.63 (0.87 - 3.06)	0.78 (0.31 - 1.96)
Intellectual Disability	topiramate	High Dose	*** / 617	26 / 8887	1.63 (0.46 - 5.69)	0.87 (0.44 - 1.74)	1.14 (0.33 - 4.00)
Intellectual Disability	topiramate	Low Dose	*** / 363	26 / 8887	3.61 (1.28 - 9.96)	0.86 (0.42 - 1.76)	3.88 (1.26 - 11.92)
Intellectual Disability	topiramate	No Child Epilepsy	*** / 1,036	15 / 8,887	0.82 (0.23 - 2.89)	0.43 (0.17 - 1.08)	1.56 (0.42 - 5.81)
Intellectual Disability	topiramate	Accounting for Censoring	*** / 1036	26 / 8887	**	**	1.81 (0.75 - 4.36)
Intellectual Disability	oxcarbazepine	Main (Crude)	*** / 845	26 / 8887	2.73 (1.05 - 6.98)	0.96 (0.59 - 1.59)	2.11 (0.87 - 5.13)
Intellectual Disability	oxcarbazepine	Main (Adjusted)	*** / 845	26 / 8887	2.62 (0.99 - 6.82)	0.57 (0.24 - 1.36)	3.30 (1.29 - 8.41)

Outcome	Exposure	Analysis	N Cases/ Total		Cum. Incidence in % (95% CI) at Age 8 Years		HR (95% CI)
			Among Exposed	Among Unexposed	Among Exposed	Among Unexposed	
Intellectual Disability	oxcarbazepine	Monotherapy	*** / 579	26 / 8887	2.40 (0.71 - 7.95)	0.59 (0.25 - 1.41)	2.27 (0.66 - 7.80)
Intellectual Disability	oxcarbazepine	Polytherapy (no Valproate)	*** / 254	26 / 8887	2.23 (0.25 - 18.75)	0.52 (0.14 - 1.85)	5.36 (1.20 - 23.96)
Intellectual Disability	oxcarbazepine	Strict Monotherapy	*** / 522	26 / 8887	3.01 (0.87 - 10.19)	0.57 (0.23 - 1.40)	2.93 (0.87 - 9.93)
Intellectual Disability	oxcarbazepine	>=2 Rx	*** / 685	26 / 8887	3.44 (1.31 - 8.86)	0.54 (0.22 - 1.35)	4.34 (1.69 - 11.14)
Intellectual Disability	oxcarbazepine	Active Comparator	*** / 1355	21 / 7381	2.08 (0.84 - 5.09)	1.96 (1.13 - 3.40)	1.04 (0.42 - 2.58)
Intellectual Disability	oxcarbazepine	High Dose	*** / 454	26 / 8887	3.04 (0.92 - 9.83)	0.56 (0.21 - 1.47)	4.21 (1.38 - 12.81)
Intellectual Disability	oxcarbazepine	Low Dose	*** / 332	26 / 8887	2.28 (0.45 - 11.11)	0.61 (0.24 - 1.54)	2.37 (0.52 - 10.72)
Intellectual Disability	oxcarbazepine	No Child Epilepsy	*** / 845	15 / 8,887	1.79 (0.64 - 4.99)	0.30 (0.08 - 1.07)	4.75 (1.50 - 15.06)
Intellectual Disability	oxcarbazepine	Accounting for Censoring	*** / 845	26 / 8887	**	**	3.26 (1.27 - 8.38)
Intellectual Disability	valproate	Main (Crude)	11 / 800	26 / 8887	2.16 (1.02 - 4.54)	0.96 (0.59 - 1.59)	2.68 (1.32 - 5.46)
Intellectual Disability	valproate	Main (Adjusted)	11 / 800	26 / 8887	1.86 (0.81 - 4.23)	1.05 (0.55 - 1.97)	1.72 (0.77 - 3.83)
Intellectual Disability	valproate	Monotherapy	*** / 561	26 / 8887	1.51 (0.52 - 4.33)	1.22 (0.64 - 2.30)	1.39 (0.54 - 3.57)
Intellectual Disability	valproate	Strict Monotherapy	*** / 515	26 / 8887	1.23 (0.34 - 4.31)	1.19 (0.63 - 2.25)	1.19 (0.44 - 3.25)
Intellectual Disability	valproate	>=2 Rx	11 / 568	26 / 8887	2.85 (1.30 - 6.21)	1.02 (0.51 - 2.03)	2.47 (1.11 - 5.49)
Intellectual Disability	valproate	Active Comparator	12 / 1392	21 / 7381	0.91 (0.33 - 2.53)	2.20 (1.22 - 3.94)	0.63 (0.25 - 1.60)
Intellectual Disability	valproate	High Dose	*** / 509	26 / 8887	1.58 (0.55 - 4.50)	0.99 (0.49 - 2.02)	1.96 (0.82 - 4.72)
Intellectual Disability	valproate	Low Dose	*** / 209	26 / 8887	1.70 (0.24 - 11.53)	1.08 (0.59 - 1.99)	0.72 (0.09 - 5.42)
Intellectual Disability	valproate	No Child Epilepsy	*** / 800	15 / 8,887	1.13 (0.39 - 3.28)	0.67 (0.31 - 1.45)	1.66 (0.60 - 4.56)
Intellectual Disability	valproate	Accounting for Censoring	11 / 800	26 / 8887	**	**	1.71 (0.75 - 3.89)
Intellectual Disability	zonisamide	Main (Crude)	*** / 446	26 / 8887	5.04 (1.51 - 16.11)	0.96 (0.59 - 1.59)	3.09 (1.08 - 8.88)
Intellectual Disability	zonisamide	Main (Adjusted)	*** / 446	26 / 8887	5.17 (1.56 - 16.38)	0.56 (0.21 - 1.50)	4.50 (1.43 - 14.18)
Intellectual Disability	zonisamide	Monotherapy	*** / 225	23 / 8593	5.91 (1.25 - 25.49)	0.56 (0.18 - 1.69)	4.99 (1.10 - 22.61)
Intellectual Disability	zonisamide	Polytherapy (no Valproate)	*** / 211	21 / 8007	4.45 (0.65 - 27.23)	0.55 (0.18 - 1.67)	3.92 (0.78 - 19.83)
Intellectual Disability	zonisamide	Strict Monotherapy	*** / 207	23 / 8593	6.89 (1.47 - 29.09)	0.55 (0.17 - 1.83)	6.02 (1.34 - 27.09)
Intellectual Disability	zonisamide	>=2 Rx	*** / 355	26 / 8887	6.21 (1.85 - 19.78)	0.51 (0.17 - 1.50)	5.54 (1.73 - 17.77)
Intellectual Disability	zonisamide	Active Comparator	*** / 508	21 / 7381	4.25 (1.20 - 14.43)	1.88 (0.89 - 3.94)	1.75 (0.54 - 5.64)
Intellectual Disability	zonisamide	High Dose	*** / 276	26 / 8887	4.28 (0.89 - 19.18)	0.62 (0.25 - 1.58)	3.68 (1.02 - 13.30)
Intellectual Disability	zonisamide	Low Dose	****	****	****	****	****
Intellectual Disability	zonisamide	No Child Epilepsy	*** / 446	15 / 8,887	2.86 (0.61 - 12.90)	0.26 (0.05 - 1.31)	4.88 (1.05 - 22.73)
Intellectual Disability	zonisamide	Accounting for Censoring	*** / 446	26 / 8887	**	**	5.05 (1.45 - 17.59)
Intellectual Disability	phenobarbital	Main (Crude)	*** / 416	26 / 8887	1.88 (0.68 - 5.09)	0.96 (0.59 - 1.59)	1.58 (0.55 - 4.58)
Intellectual Disability	phenobarbital	Main (Adjusted)	*** / 416	26 / 8887	2.20 (0.57 - 8.32)	0.43 (0.03 - 6.42)	3.70 (0.85 - 16.11)
Intellectual Disability	phenobarbital	Monotherapy	*** / 255	26 / 8148	2.43 (0.48 - 11.95)	0.32 (0.01 - 15.33)	4.47 (0.92 - 21.64)
Intellectual Disability	phenobarbital	Polytherapy (no Valproate)	****	****	****	****	****
Intellectual Disability	phenobarbital	Strict Monotherapy	****	****	****	****	****
Intellectual Disability	phenobarbital	>=2 Rx	*** / 319	26 / 8887	1.47 (0.26 - 7.98)	0.29 (0.00 - 40.86)	4.29 (0.52 - 35.69)
Intellectual Disability	phenobarbital	Active Comparator	*** / 934	21 / 7381	1.37 (0.38 - 4.91)	2.69 (1.19 - 6.03)	0.48 (0.11 - 2.14)
Intellectual Disability	phenobarbital	High Dose	*** / 221	26 / 8887	0.19 (0.00 - 32.21)	0.16 (0.00 - 62.83)	0.84 (0.07 - 9.37)
Intellectual Disability	phenobarbital	Low Dose	****	****	****	****	****
Intellectual Disability	phenobarbital	No Child Epilepsy	*** / 416	15 / 8,887	1.42 (0.23 - 8.66)	0.34 (0.01 - 7.88)	2.99 (0.57 - 15.61)
Intellectual Disability	phenobarbital	Accounting for Censoring	*** / 416	26 / 8887	**	**	2.38 (0.61 - 9.21)
Intellectual Disability	lacosamide	Main (Crude)	*	*	*	*	*
Intellectual Disability	lacosamide	Main (Adjusted)	*	*	*	*	*
Intellectual Disability	lacosamide	Monotherapy	****	****	****	****	****
Intellectual Disability	lacosamide	Polytherapy (no Valproate)	****	****	****	****	****
Intellectual Disability	lacosamide	Strict Monotherapy	****	****	****	****	****
Intellectual Disability	lacosamide	>=2 Rx	****	****	****	****	****
Intellectual Disability	lacosamide	Active Comparator	*	*	*	*	*

Outcome	Exposure	Analysis	N Cases/ Total		Cum. Incidence in % (95% CI) at Age 8 Years		HR (95% CI)
			Among Exposed	Among Unexposed	Among Exposed	Among Unexposed	
Intellectual Disability	lacosamide	High Dose	****	****	****	****	****
Intellectual Disability	lacosamide	Low Dose	****	****	****	****	****
Intellectual Disability	lacosamide	No Child Epilepsy	*	*	*	*	*
Intellectual Disability	lacosamide	Accounting for Censoring	*	*	*	*	*

Note: Cumulative incidences at other ages will be made available upon request.
Abbreviation: ADHD, attention deficit hyperactivity disorder; cum., cumulative; CI, confidence interval; HR, hazard ratio; N, number; NDD, neurodevelopmental disorder; Rx, prescription dispensing.
Individual medications are sorted by the number of exposed pregnancies in both cohorts combined; individual neurodevelopmental disorders are sorted based on the absolute risk observed in the overall population at 8 years of age.
The dose distribution of each medication of interest (based on the dispensing with the highest daily dose during the assessment period) is presented in eTable 8. High- and low-dose users were categorized using the median dose as the cut-off point.
*No results presented due to 0 exposed cases.
**Cumulative incidence not estimable in models accounting for censoring.
***Cell size <11 suppressed in accordance with CMS' cell suppression policy.
****Analyses not conducted as exposure count was <200 for respective comparison.

eTable 8. Dose Distribution (in mg) of Each Medication of Interest Based on the Dispensing with the Highest Daily Dose During the Second Half of Pregnancy.

Exposure	N Pregnancies Total	N Pregnancies Missing	Mean	SD	Minimum	Quartile 1	Median	Quartile 3	Maximum
Levetiracetam	5,261	346	1,838	1,086	1	1,000	1,500	2,250	30,000
Lamotrigine	4,299	268	382	393	0	200	300	500	18,000
Carbamazepine	1,924	216	799	1,441	1	400	600	800	48,000
Phenytoin	1,595	164	430	507	1	300	400	500	18,000
Topiramate	1,036	56	250	461	0	100	200	300	12,000
Oxcarbazepine	845	59	1,196	634	4	600	1,200	1,500	3,600
Valproate	800	82	1,207	734	8	750	1,000	1,500	6,000
Zonisamide	446	38	362	460	17	200	300	400	9,000
Phenobarbital	416	28	145	123	7	97	120	194	1,600
Lacosamide	219	<11	336	147	100	200	400	400	1,059

N pregnancies missing refers to the number of pregnancies with exposure to medication formulations other than tablets or capsules or with unrealistic days' supply (=0 or >365). Extreme outlier values reflect data entry errors in the source data.
Cell size <11 suppressed in accordance with CMS' cell suppression policy.

eAppendix 1. Internal Validation of Algorithm to Identify Epilepsy.
Pre-existing conditions are imperfectly recorded in healthcare databases. A natural strategy to capture most conditions is to ascertain them during a long interval. However, this approach reduces sample size when only individuals enrolled for the entire duration of the interval are considered eligible for analysis. We evaluated the impact of different claims-based algorithms to identify pregnant women with epilepsy.

To determine the optimal window before delivery from which to ascertain epilepsy we used the pregnancy cohort in the 2011-2014 MarketScan® Database (n=1,894,935 deliveries). We restricted to women continuously enrolled with prescription claims data for at least three years before delivery (n=111,780 [6%]). As women with epilepsy are advised to see a physician at least once a year, three years ensured that the majority of women would have at least 2-3 contacts with the medical system. From this cohort we ascertained epilepsy in increasing longer 90-day intervals before delivery, e.g. 90, 180… 1,080 days. Epilepsy was defined as any of the ICD-9-CM codes 345.0x-345.9x, 649.4x, on two separate days plus at least one prescription claim for the medications listed in Table A1. A strict definition was used to maximize the proportion of true cases selected (i.e., specificity).

Table A1. Anti-Epileptic Drugs used in the Pre-Delivery Definition of Epilepsy (2011-2014)

Anti-Epileptic Drugs
Brivaracetam, Carbamazepine, Clobazam, Clonazepam, Diazepam, Divalproex, Eslicarbazepine, Ethosuximide, Ezogabine, Felbamate, Gabapentin, Lacosamide, Lamotrigine, Levetiracetam, Lorazepam, Oxcarbazepine, Perampanel, Phenobarbital, Phenytoin, Primidone, Rufinamide, Tiagabine, Topiramate, Valproic Acid/ Valproate, Vigabatrin, Zonisamide.

The proportion of prevalent (pre-existing) cases ascertained in each 90-day interval were plotted using closed circles and squares in Figure A1. As expected, the proportion of women classified with the condition increased with length of the pre-delivery interval during which diagnostic criteria were assessed. For epilepsy, the proportion was 0.36% around 450 days before delivery and 0.46% at the 1,080-day mark. Similar patterns were observed when we considered Multiple Sclerosis.

Figure A1. Proportion of Identified Cases and Number Continuously Enrolled with Increasing Days of Ascertainment before Delivery (2011-2014)

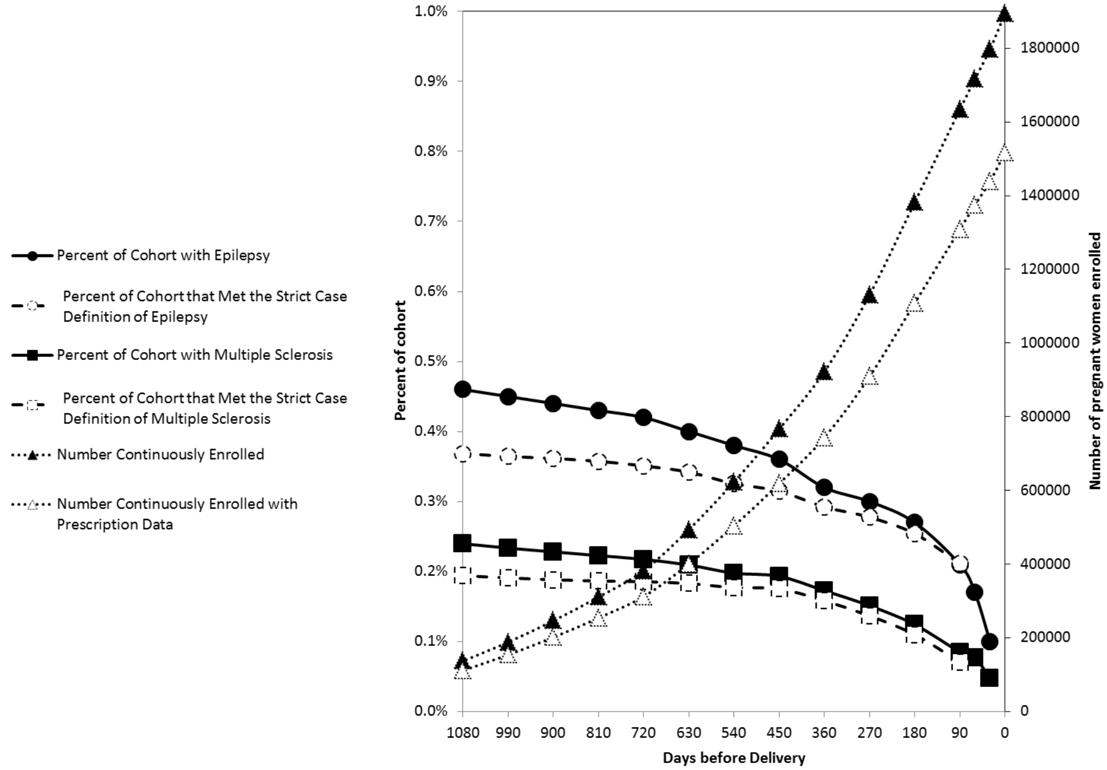

To confirm that the ascertained cases were true cases, we next performed a claims review on a sub-sample of them. We randomly sampled up to eight epilepsy cases newly identified in each 90-day interval for a full claims profile review (numbers chosen to represent the fewest number of new cases added in a single 90-day interval). Note that cases were

only eligible for selection during the interval in which they transitioned from a non-case to a case. Two reviewers classified cases as either "Met" or "Did Not Meet" a strict case definition defined in Table A2.

Table A2. Strict Case Definitions for Claims Review Validation

≥ 10 Epilepsy Codes[a]
OR
Lack of all the Following Exclusion Criteria:<br>Epilepsy codes[a] only co-existing with drug abuse codes<br>Epilepsy codes[a] only co-existing with preeclampsia/hypertension codes or codes only occurring around delivery<br>Epilepsy codes[a] only co-existing with other comorbidities that could lead to seizures (e.g. uncontrolled diabetes, PTSD, anorexia, anxiety, syncope)<br>Epilepsy codes[a] only clustered in a small window of time

[a] Epilepsy Codes = ICD-9-CM 345.0x-345.5x, 345.7x-345.9x, 649.4x

In the claims review, we classified 61% of the sampled epilepsy cases as meeting the strict case definitions. However, these percentages varied greatly by interval time (e.g., 71% met the strict epilepsy definitions in cases ascertained in the first 540 days before delivery vs. 51% in cases ascertained in the 541-1,080 day period before delivery).

We applied the percentage meeting the strict claims review definitions from our sub-sample to the full cohort of ascertained cases and plotted the percentages using open circles and squares in Figure A1. Although the absolute number of ascertained cases continued to climb over time, the proportion of cases meeting the strict case definitions in our sub-sample review tended to plateau around 450-630 days before delivery.

Finally, in Figure A1 we plotted how overall sample size declines as one requires increasingly longer continuous enrollment time before delivery (closed triangles). Additionally, in studies using prescription claim information, sample size is even further reduced as not all patients are covered by healthcare insurance with prescription claim information available in MarketScan (open triangles).

To determine the optimal window for case ascertainment in the pre-delivery interval we considered the following information collected up to this point: (1) Proportion of the cohort ascertained with the pre-existing conditions over time (2) Proportion of ascertained cases that met strict case definitions upon detailed claims reviews, and (3) Absolute sample size available for the selected interval. Given these criteria, we recommend an interval of 270-450 days before delivery for livebirths (or 0-180 days before the estimated last menstrual period), as (1) a large proportion of cases were ascertained in this interval, (2) most of the ascertained cases in this window also met the strict case definition applied upon detailed claims reviews, and (3) overall sample size was still reasonably large. (Studies evaluating early pregnancy events may want to modify the upper limit of the ascertainment window to a number of days before events are possible, or to the estimated LMP date.)

Then, we identified epilepsy using different combinations of diagnosis codes and anticonvulsant dispensations and estimated risk ratios of the association between epilepsy based on different definitions and selected pregnancy outcomes. Of 1,894,935 deliveries, 909,065 (48%) were to women continuously enrolled in a healthcare plan with prescription claim data available for 270 days before the delivery date. Of these, 2,248 (0.2%) were classified as having epilepsy. The number of cases identified varied depending on whether the delivery day was included, the required number of diagnoses codes in different days recorded and whether pharmacologic treatment was requested. Table A3.

Table A3. Sample Size for Varying Definitions of the Pre-Existing Epilepsy (2011-2014)

Pre-Existing Condition	Definition	n
Epilepsy	2+ Epilepsy Codes & 1+ Anti-epileptic Drug[a]	2,248
	2+ Epilepsy Codes & 1+ Anti-epileptic Drug (Excluding delivery admission)	2,035
	1+ Epilepsy Codes & 1+ Anti-epileptic Drug	2,612
	2+ Epilepsy Codes	2,904
	1+ Epilepsy Code (Excluding delivery admission)	3,618

[a] Original definition

Generally, the period of ascertainment did not meaningfully impact the estimated associations with obstetric outcomes. (Figure A2) However the risk ratio for preeclampsia associated with epilepsy was 1.67 [95% CI: 1.47, 1.90] when epilepsy was ascertained at delivery and 1.26 [95% CI: 1.07, 1.48] when epilepsy was ascertained in the pre-delivery interval (heterogeneity, p=0.007). Delivery-coded epilepsy was less likely confirmed in the pre-delivery interval in the presence of preeclampsia. The recording of epilepsy codes at delivery may reflect neurologic symptoms associated with preeclampsia, including eclamptic seizures, being misclassified as epilepsy. Therefore, the ascertainment window should stop before delivery.

Figure A2. Adjusted Risk Ratios of Obstetric Outcomes associated with Epilepsy Defined using Varying Pre-Delivery Definitions of the Condition (2011-2014)

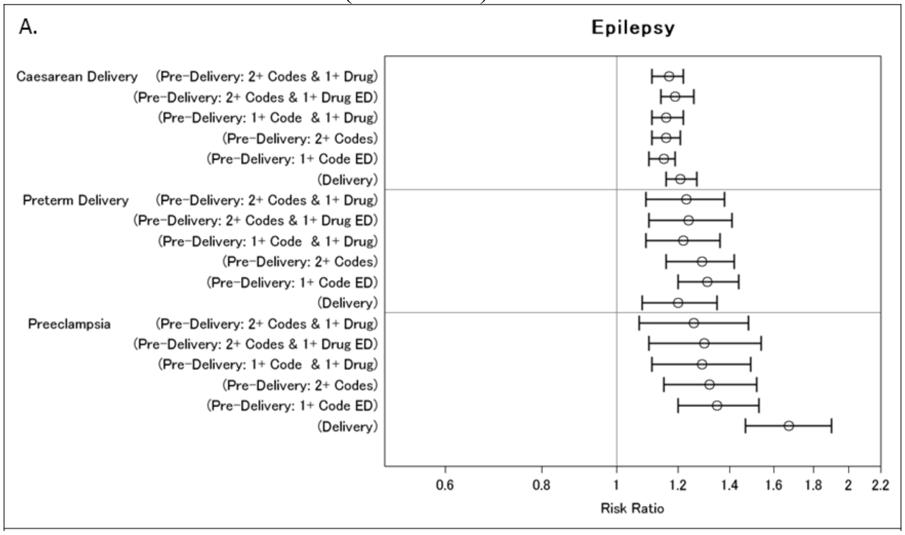

ED = excluding delivery admission

Conclusions:
In conclusion, we recommend identifying "epilepsy" using the interval from "LMP-180" until "delivery-1" defined as either (>=2 epilepsy ICD-9 and ICD-10 codes in two different days) OR (1 code an >=1 prescription an anticonvulsant).

eAppendix 2. STROBE Statement—Checklist Of Items That Should be Included in Reports of Cohort Studies

	Item No	Recommendation	Page No
**Title and abstract**	1	(*a*) Indicate the study's design with a commonly used term in the title or the abstract<br>(*b*) Provide in the abstract an informative and balanced summary of what was done and what was found	3-4
**Introduction**			
Background/rationale	2	Explain the scientific background and rationale for the investigation being reported	5-7
Objectives	3	State specific objectives, including any prespecified hypotheses	7
**Methods**			
Study design	4	Present key elements of study design early in the paper	7
Setting	5	Describe the setting, locations, and relevant dates, including periods of recruitment, exposure, follow-up, and data collection	7-8
Participants	6	(*a*) Give the eligibility criteria, and the sources and methods of selection of participants. Describe methods of follow-up<br>(*b*) For matched studies, give matching criteria and number of exposed and unexposed	7-8
Variables	7	Clearly define all outcomes, exposures, predictors, potential confounders, and effect modifiers. Give diagnostic criteria, if applicable	8-9
Data sources/ measurement	8*	For each variable of interest, give sources of data and details of methods of assessment (measurement). Describe comparability of assessment methods if there is more than one group	7-9
Bias	9	Describe any efforts to address potential sources of bias	7-11
Study size	10	Explain how the study size was arrived at	8
Quantitative variables	11	Explain how quantitative variables were handled in the analyses. If applicable, describe which groupings were chosen and why	8-9
Statistical methods	12	(*a*) Describe all statistical methods, including those used to control for confounding<br>(*b*) Describe any methods used to examine subgroups and interactions<br>(*c*) Explain how missing data were addressed<br>(*d*) If applicable, explain how loss to follow-up was addressed<br>(*e*) Describe any sensitivity analyses	9-11
**Results**			
Participants	13*	(a) Report numbers of individuals at each stage of study—eg numbers potentially eligible, examined for eligibility, confirmed eligible, included in the study, completing follow-up, and analysed<br>(b) Give reasons for non-participation at each stage<br>(c) Consider use of a flow diagram	11
Descriptive data	14*	(a) Give characteristics of study participants (eg demographic, clinical, social) and information on exposures and potential confounders<br>(b) Indicate number of participants with missing data for each variable of interest<br>(c) Summarise follow-up time (eg, average and total amount)	11-12
Outcome data	15*	Report numbers of outcome events or summary measures over time	12

Main results	16	(*a*) Give unadjusted estimates and, if applicable, confounder-adjusted estimates and their precision (eg, 95% confidence interval). Make clear which confounders were adjusted for and why they were included<br>(*b*) Report category boundaries when continuous variables were categorized<br>(*c*) If relevant, consider translating estimates of relative risk into absolute risk for a meaningful time period	12-13
Other analyses	17	Report other analyses done—eg analyses of subgroups and interactions, and sensitivity analyses	13-14
**Discussion**			
Key results	18	Summarise key results with reference to study objectives	14-15
Limitations	19	Discuss limitations of the study, taking into account sources of potential bias or imprecision. Discuss both direction and magnitude of any potential bias	17-18
Interpretation	20	Give a cautious overall interpretation of results considering objectives, limitations, multiplicity of analyses, results from similar studies, and other relevant evidence	15-17, 19
Generalisability	21	Discuss the generalisability (external validity) of the study results	15-17
**Other information**			
Funding	22	Give the source of funding and the role of the funders for the present study and, if applicable, for the original study on which the present article is based	20

*Give information separately for exposed and unexposed groups.

Note: An Explanation and Elaboration article discusses each checklist item and gives methodological background and published examples of transparent reporting. The STROBE checklist is best used in conjunction with this article (freely available on the Web sites of PLoS Medicine at http://www.plosmedicine.org/, Annals of Internal Medicine at http://www.annals.org/, and Epidemiology at http://www.epidem.com/). Information on the STROBE Initiative is available at http://www.strobe-statement.org.
